# Spatiotemporal and Species-Crossing Transmission Dynamics of Subclade 2.3.4.4b H5Nx HPAIVs

**DOI:** 10.1155/2024/2862053

**Published:** 2024-07-10

**Authors:** Minghui Li, Jingman Tian, Xiaoli Bai, Xingdong Song, Zhiguo Zhao, Jianzhong Shi, Guohua Deng, Xianying Zeng, Guobin Tian, Huihui Kong, Jinxiong Liu, Chengjun Li, Yanbing Li

**Affiliations:** State Key Laboratory for Animal Disease Control and Prevention Harbin Veterinary Research Institute Chinese Academy of Agricultural Sciences, Harbin, Heilongjiang province, China

## Abstract

Subclade 2.3.4.4b H5Nx highly pathogenic avian influenza (HPAI) viruses, emerged in 2013 with multiple subtypes of H5N8, H5N1, and H5N6, had unprecedently caused a global epizootic by H5N1 since 2021, which had devasted multiple species of wild birds, poultry, and wild mammals (terrestrial and marine) with a high mortality, causing severe ecological damage. The infected wild mammals may become new “mixers” for influenza viruses, posing the potential transmission to human. Frequent outbreaks of subclade 2.3.4.4b H5Nx viruses among wild birds and poultry had exposed major gaps in our knowledge on their evolution, spatiotemporal diffusion, and species-crossing transmission. Here, we integrated the phylogenetic and epidemiological data of subclade 2.3.4.4b H5Nx viruses in public database and used Bayesian phylodynamic analysis to reveal the pattern of the global large-scale transmission. Phylogenic analysis demonstrated that the HA gene of these viruses diverged into two dominant clusters around 2015 and 2016. The Bayesian phylodynamic analysis illustrated that the viruses presented spatiotemporally complex transmission network with geographical and host relative expansion and recombination with different subtypes of NA segment. Spatially, the Russian Federation (Siberia) was identified as the primary hub for virus transmission, which was further facilitated by the establishment of strong epidemiological linkages between West Europe and broader regions, such as North America. As for hosts, wild Anseriformes were the primary species for the virus spillover, contributing to the spatial expansion and rapid diffusion globally of subclade 2.3.4.4b viruses. We investigated the phylogeny of subclade 2.3.4.4b H5Nx viruses and the spatiotemporal pattern of transmission with initial location and the primary host, which could provide comprehensive insights for subclade 2.3.4.4b H5Nx viruses. Due to the wild birds involved the widespread of subclade 2.3.4.4b H5Nx viruses, the epizootics in poultry are inevitable, so we highly recommend to apply the policy of culling plus with vaccination to protect the poultry industry and potentially protect the public health.

## 1. Introduction

Wild waterfowl in Anseriformes and Charadriiformes are considered to be the natural hosts for avian influenza viruses (AIVs) and play an important role in the ecology and evolution of AIVs. Majority of wild waterfowl are highly active, migrating tens to thousands of kilometers between their breeding and wintering grounds each spring and autumn. Large-scale aggregations of migratory birds at stopover sites provide optimal conditions for the evolution, long-distance transmission, and spillover to other species of AIVs [1, 2, 3].

According to WOAH criteria for the classification of HA genes of H5 HPAI viruses, the clade 2.3.4.4 H5 viruses have evolved into eight subclades as a–h since they were isolated in 2008 [4] and have already caused three major intercontinental outbreaks since 2014 [5]. In early 2014, H5N8 viruses bearing the HA genes of subclade 2.3.4.4b (H5N8B, Gochang-like) and subclade 2.3.4.4c (H5N8A, Buan-like) caused outbreaks in poultry and infections in migratory birds in Korea; subsequently, the H5N8 of subclade 2.3.4.4c viruses were disseminated to Europe and North America in autumn 2014 [6, 7, 8]. Notably, the pathobiological features suggested that the H5N8 of subclade 2.3.4.4c viruses were highly adapted to waterfowl but poorly adapted to chickens and turkeys, thereby causing significant morbidity and mortality in domestic poultry but varied disease severities, including subclinical infection, in wild birds and domestic waterfowl [9, 10, 11, 12]. During 2016–2017, the H5N8 of subclade 2.3.4.4b viruses remerged and caused outbreaks in wild birds and domestic poultry across Europe and many countries in the Asia and Africa [13, 14, 15, 16, 17, 18]. In contrast to the H5N8 of subclade 2.3.4.4c viruses during 2014–2015, H5N8 of subclade 2.3.4.4 b viruses during 2016–2017 expressed augmented virulence for waterfowl but still low zoonotic potential [12, 19, 20]. During 2020–2021, there was a resurgence of outbreaks of the H5N8 of subclade 2.3.4.4b viruses in Europe, followed by extensive outbreaks in wild birds and domestic poultry in many countries in the Asia and Africa [21, 22, 23, 24, 25]. Remarkably, the H5N8 of subclade 2.3.4.4b viruses during 2020–2021 had caused some wild mammalian infection, such as seals and foxes in the United Kingdom [26], even the human infection in Russia [27], demonstrating a higher public health risk. A novel recombinant H5N1 of subclade 2.3.4.4b viruses, from H5N8 reassorted with H1N1, was first detected in Netherlands in October 2020 [28] and rapidly initiated a new wave of intercontinental transmission that even extended to North America, South America, and Antarctica [29, 30, 31, 32]. The World Animal Health Information System (WAHIS) reported on March 13, 2024, that there were 485 species from over 25 avian orders and 37 new mammal species (including both marine and terrestrial animals) infected with the virus since 2021, which poses a serious public risk.

The ongoing subclade 2.3.4.4b H5Nx HPAI (hereafter, subC2344b H5Nx) viruses pose an increasing threat to public health, and a comprehensive understanding of how these viruses circulate in nature is vital to better counter the aggressive HPAI outbreaks in multiple species. In this study, we performed phylogenetic and epidemiological analysis of HA gene of subC2344b H5Nx viruses and used Bayesian phylodynamic analysis to reveal the pattern of global large-scale transmission of the subC2344b H5Nx viruses and to elucidate the contribution of different host taxa in virus transmission; thus, the molecular epidemiology and transmission dynamics of the subC2344b H5Nx viruses were determined.

## 2. Materials and Methods

### 2.1. Phylogenetic and Epidemiological Analysis of HA Gene of subC2344b H5Nx Viruses

The HA gene of all H5 sequences was downloaded that were available on EpiFlu database of the Global Initiative on Sharing All Influenza Data (GISAID, https://www.gisaid.org) (accessed on 1st January 2023) with information on their collection date, sampling location, host, and NA subtype. Sequences with duplicated, missing, significant shift errors and lengths less than 80% integrity were removed, and the remaining sequences were used for phylogenetic analysis. Sequences were aligned using MAFFT v7.475 with default parameters and manually adjusted using MEGA v7.0. The phylogenetic tree was constructed by maximum likelihood (ML) method using IQ-Tree v1.6.12 [33], with the best alternative model (GTR + F + I + G4) automatically selected by ModelFinder [34]. SubC2344b H5Nx viruses were selected based on the classification of the HA sequence that was made adhering to the nomenclature system recommended by FAO/WHO/WOAH H5N1 Evolution Working Group and previously published papers [4, 15].

We annotated and visualized the ML tree and epidemiological information (collection date, sampling location, host, and NA subtype) of the entire subC2344b H5Nx HA dataset using the R package ggtree v2.4.2 [35]. We roughly divided the epidemic of the viruses into four periods: early 2015 period, 2016–2017 period, 2018–2019 period, and 2020–2022 period. According to the virus isolation locations, all sequences were classified into 10 countries or regions: West Europe, East Europe, Russian Federation (Siberia), West Central Asia, Korea, China, Japan, Africa, North America, and South America. Hosts were divided into six subclasses based on the globally recognized authority on bird taxonomy website (eBird.org) and their living habits: wild Anseriformes (such as Anas, Anser, and Cygnus), domestic Galliformes (including chickens and quails), domestic Anseriformes (ducks and geese), Charadriiformes (such as gulls and curlews), other wild species, and mammals, and environmental samples were removed from the dataset. Subtypes were grouped according to their NA genes: H5N1, H5N2, H5N3, H5N4, H5N5, H5N6, and H5N8.

### 2.2. Spatiotemporal Bayesian Evolutionary Analysis of subC2344b H5Nx Viruses

We used a Bayesian discrete phylogeographic approach to investigate spatial diffusion of subC2344b H5Nx viruses [36]. To mitigate the potential sampling bias in Bayesian inference estimation caused by oversampling in the dataset, the subC2344b H5Nx HA dataset was manually randomly subsampled to select less than or equal to 10 sequences per region, per host, and per month of each year. Subsequently, we grouped the HA sequences by sampling countries and different provinces in China to investigate which discrete partitioning strategy could sample the sequence to approximately equal numbers at each location. Ultimately, the final subsampled HA dataset contained 518 sequences, and countries or regions of virus sampling were grouped into 12 geographical regions: West Europe (124), East Europe (60), Russian Federation (Siberia) (68), West Central Asia (33), Korea (29), East China (28), Central China (24), West China (20), Japan (38), Africa (45), North America (42), and South America (7).

Root-to-tip genetic distance inferred from ML trees was regressed against sampling time using TempEst v1.5.3 [37], and then we removed the outlier sequences based on estimated distance to ensure that the selected sequences had sufficient temporal structure in alignment for reliable rate estimation for each dataset. For the discrete phylogeographic analysis, we specified the SRD06 nucleotide substitution model [38] for estimating the divergence time of the virus, the uncorrelated lognormal relaxation clock model, and a Bayesian skyline [39] coalescent tree prior, as well as the Markov chain Monte Carlo (MCMC) [40] method in a Bayesian statistical framework for inference. To determine the most probable viral migration networks, we reconstructed the virus transmission history between states geographically using a combination of asymmetric transition matrix with Bayesian stochastic search variable selection (BSSVS). We assigned the positional states for each virus sequence to estimate the expected number of transitions (Markov jumps) between each pair of regions and the time spent in each state (Markov rewards) along the phylogenetic branches of the tree. For each dataset, three independent MCMC chains were run for 300 million steps in the BEAST v1.10.4 package [41] and were combined with the BEAGLE library [42], with sampling every 30,000 steps. The program Tracer v1.7.2 was used to check multiple MCMC chain convergence and adequate mixing to ensure that all estimated parameters had effective sample size (ESS) values greater than 200. LogCombiner v1.10.4 was used to combine and subsample posterior distribution, after removing the first 10% of the chains as burn-in. The maximum clade credibility (MCC) tree was generated using TreeAnnotator v1.10.4 [41] to summarize the posterior tree distribution for subsequent phylogeographic analysis and visualized with ggtree. Subsequently, SpreaD3 v0.9.6 [43] takes a rate matrix file for location states generated under Beast analysis, calculating the Bayes factor (BF), and posterior probabilities (PP) to assess the statistical support for individual transitions between different geographical locations and hosts. When BF ≥ 3 and PP ≥ 0.5, the migration paths were regarded as being supported; BF ≥ 1,000 was deemed as decisive support, 100 ≤ BF < 1,000 as very strong support, 10 ≤ BF < 100 as strong support, and 3 ≤ BF < 10 as supported. The region and host with the highest BF support were denoted as the most probable transmission hub for global viral migration.

To complement the discrete characterization and obtain a more robust and comprehensive estimates of virus evolutionary inference, we labeled the coordinates in space, such as latitude and longitude of the sampled taxonomic units in trees for continuous Bayesian phylogeography analysis. A relaxed random walk (RRW) diffusion model [44] and a relaxed uncorrelated lognormal molecular clock were employed, with 0.001 random jitter added to the spatial coordinates to reduce overlap. MCMC chains were run for 1 billion generations, with sampling every 100,000 generations, and the first 10% were discarded as burn-in. MCC tree was summarized using TreeAnnotator v1.10.4 from 10,000 trees regularly sampled from each posterior distribution. We used the R package “seraphim” [45, 46] to extract and map the spatiotemporal information embedded in posterior trees.

### 2.3. Species-Crossing Transmission of subC2344b H5Nx Viruses

To identify the dispersal events across different hosts in the whole genome of subC2344b H5Nx viruses, we analyzed the transmission dynamics and the associated species-crossing jumps in the intricate transmission dynamics of eight external genes (H5, N1, N2, N3, N4, N5, N6, and N8) and six internal genes (PB2, PB1, PA, NP, M, and NS). We defined a possible species-crossing transmission event as nucleotide homology >99% in the same region within 3 years and sampled separately from two different species. Different NA genes and internal genes corresponded to the selected subC2344b HA sequences and separately downloaded the above sequences from the EpiFlu database for further analysis. The transition of 14 independent datasets between host taxa was estimated using a discrete ancestral state reconstruction method and asymmetric host transitions: H5, *n* = 518; N1, *n* = 139; N2, *n* = 45; N3, *n* = 25; N4, *n* = 17; N5, *n* = 32; N6, *n* = 131; N8, *n* = 247; PB2, *n* = 608; PB1, *n* = 714; PA, *n* = 552; NP, *n* = 677; M, *n* = 487; and NS, *n* = 666.

To quantify the contributions of different host taxa in the expansion dynamics of subC2344b H5Nx viruses, we capitalized on the subsampled HA dataset to incorporate both a discrete host transmission process and a continuous spatial diffusion process in a single Bayesian analysis. Afterward, we established associations between the branches and specific host taxa to calculate statistical information on host-specific trajectories. We utilized the latitude and longitude of each instance to summarize the average rate of virus dissemination within the host population, referred to as the host-specific diffusion rate (km/year).

## 3. Results

### 3.1. Phylogenetic and Epidemiology of HA Gene of subC2344b H5Nx Viruses

**Figure 1:**
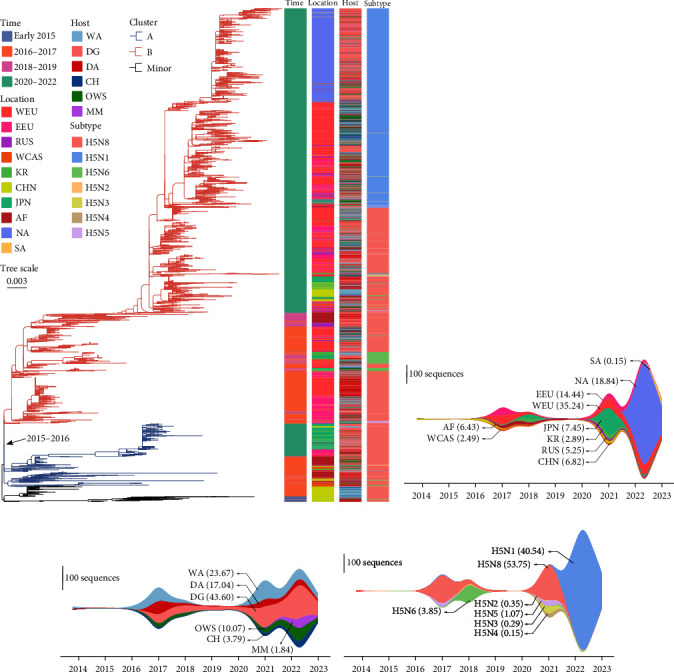
Statistics and phylogeny of the worldwide HA gene of subC2344b H5Nx viruses. (a) Phylogenetic tree of HA gene of global subC2344b H5Nx and its groupings. Categories of virus (such as isolation time, locations, hosts, and subtypes) are identified by the different colors. Different highlighted colors and their corresponding labels depict the major phylogenetic groups. Temporal changes in the prevalence of subC2344b H5 viruses and its corresponding proportions in different locations (b), different hosts (c), and different subtypes (d) estimated using sample collection dates of sequences submitted to the GISAID database. Countries/regions are abbreviated as follows: West Europe = WEU, East Europe = EEU, Russian Federation (Siberia) = RUS, West Central Asia = WCAS, Korea = KR, China = CHN, Japan = JPN, Africa = AF, North America = NA, and South America = SA. Hosts are abbreviated as follows: wild Anseriformes = WA, domestic Galliformes = DG, domestic Anseriformes = DA, Charadriiformes = CH, other wild species = OWS, and mammals = MM.

To better understand the evolution and prevalence of subC2344b H5Nx viruses, we performed phylogenetic analysis of 4,778 HA gene sequences from GISAID database and further classified based on isolation time, locations, hosts, and subtypes (Figure 1(a), *Supplementary table 1*). The phylogenetic analysis of HA gene showed that the subC2344b H5Nx viruses diverged into two distinct genetic clusters, Cluster A and Cluster B, during 2015–2016 period approximately. Geographically, Cluster A and Cluster B had spread to a wide geographical area, including Europe, Asia, and Africa, whereas Cluster B had been further expanded to North America and South America since the late of 2021 and the late of 2022 (Figures 1(a) and 1(b)). By the number, the largest proportion of virus sequences was from West Europe (35.24%), with the next largest from North America (18.84%), followed by East Europe (14.44%) and Japan (7.45%). With regard to the infected hosts, the subC2344b H5Nx virus was first identified from wild Anseriformes in the late of 2013. Subsequently, the virus infected a wide range of hosts within Cluster A and Cluster B, with the largest proportion of domestic Galliformes (43.60%), followed by wild Anseriformes (23.67%), domestic Anseriformes (17.04%), other wild birds (10.07%), Charadriiformes (3.79%), and mammals (1.84%). Before 2017, wild Anseriformes was the major isolated host; nevertheless, a rising trend had been observed in domestic Galliformes since 2017 (Figure 1(c)). As for the subtypes, H5 and multiple NA subtypes were recombined, including H5N1, H5N2, H5N3, H5N4, H5N5, H5N6, and H5N8. Cluster A contained four subtypes (H5N2, H5N5, H5N6, and H5N8), and Cluster B contained seven subtypes, with the largest proportion of H5N8 subtype (53.75%), followed by H5N1 (40.54%) and H5N6 (3.85%) subtypes. By contrast, the ratios of the remaining four H5Nx viruses were low. Among all the viruses, H5N8 subtype HPAI virus was the most frequently detected virus until 2020, while the new recombinant H5N1 virus has increased dramatically and gradually become the dominant virus between 2021 and 2022. It was notable that the rate of H5N6 virus detection was increasing during 2017–2018 and one incident of human infection with subC2344b H5N6 virus (Figure 1(d)) [47].

### 3.2. Spatiotemporal Dispersal of subC2344b H5Nx Viruses

To gain further insight into the large-scale transmission pattern of subC2344b H5Nx virus, the HA gene sequences of 518 viruses were analyzed to reconstruct their spatiotemporal dispersal. A root-to-tip regression of genetic distance against sampling time evidenced sufficient temporal signal in the representative HA sequences, with a correlation coefficient of 0.981 and *R*^2^ of 0.958 (*Supplementary figure 2*).

**Figure 2:**
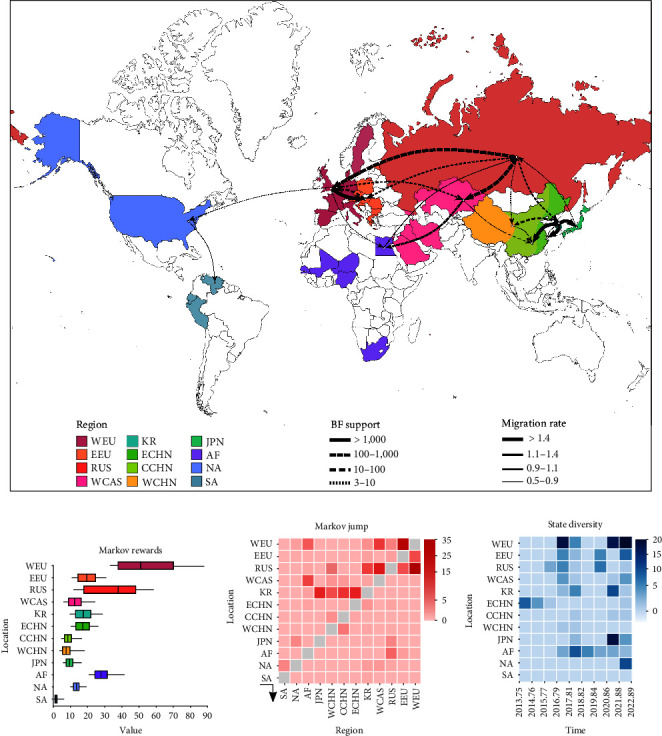
Global migration rates between geographic regions of subC2344b H5Nx viruses. (a) Map showing interregion viral lineage movements, which was determined by Bayesian phylogeography inference of HA gene sequences of subC2344b H5Nx. Colors represent different sampling regions. Curves indicate the interregion virus lineage transitions statistically supported with Bayes factors (BF) ≥ 3 and posterior probabilities (PP) ≥ 0.5; curve widths represent migration rate values. (b) and (c) represent the time spent in each geographical regions and the number of transitions between different geographical regions, respectively. (d) The number of viruses transitions between different geographical regions at different time points. Countries/regions are abbreviated as follows: West Europe = WEU, East Europe = EEU, Russian Federation (Siberia) = RUS, West Central Asia = WCAS, Korea = KR, East China = ECHN, Central China = CCHN, West China = WCHN, Japan = JPN, Africa = AF, North America = NA, and South America = SA.

Discrete phylogeographic analysis revealed a complex global viral migration network of 21 credible migration paths (BF ≥ 3, and PP ≥ 0.5), with the Russian Federation (Siberia) and the West Europe involved in seven and six supported migration paths, which indicated that these two regions played critical roles in the virus dispersal (Figure 2(a), *Supplementary table 3*). In particular, virus transmission in the Russian Federation (Siberia) was confined to the Eurasian–African continent, whereas the transmission in West Europe had expanded to the Eurasian–African and North American continents. Additionally, virus dissemination also happened between Korea, playing a crucial role in virus dispersal throughout Asia, and other five geographical regions on the Asian continent. Among these regions investigated, the Russian Federation (Siberia), West Central Asia, and Korea seem to have been the key probable origins of subC2344b H5Nx viruses found in China, which have invaded West China, Central China, and East China through various routes (Figure 2(a)). Notably, we observed that the stronger supported migration paths (BF > 1,000) occurred between closer regions, including from Korea to East China, Japan, and Central China, from West Europe to East Europe, and from North America to South America. Furthermore, we used Markov rewards to estimate the time spent in each region, and the data revealed that these viruses spent considerable amounts of time in both the Russian Federation (Siberia) and West Europe (Figure 2(b)). Frequent region switches and the high migration rates occurred from the Russian Federation (Siberia) to the West Europe (Markov jumps = 32.61, migration rate = 4.16) and from West Europe to East Europe (Markov jumps = 29.54, migration rate = 2.82) (Figure 2(c), *Supplementary table 4* and *Supplementary table 5*). Region switches reflected this dynamic with outward migration from the Russian Federation (Siberia) and West Europe dominating. However, the total mean migration rates showed migration out of the Russian Federation (Siberia) to other regions, albeit with higher number of inward mean rates for West Europe. Therefore, our data indicated that the Russian Federation (Siberia) acted as the central hotspot for the diffusion and dissemination of subC2344b H5Nx viruses; meanwhile, West Europe established strong epidemiological links with broader regions in this transmission network and played an essential role in the spread of the epidemic. Furthermore, our findings highlighted the pivotal role of Africa in the dissemination of subC2344b H5 viruses during 2017–2019 (Figure 2(d)), with 14 region switching events happened during this period.

**Figure 3:**
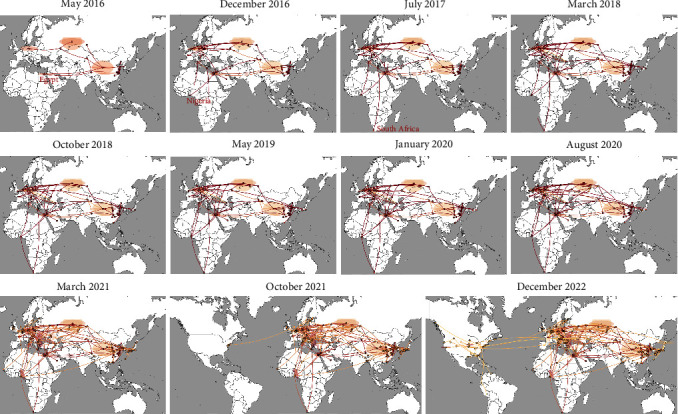
Global spatiotemporal dissemination of subC2344b H5Nx viruses. The continuous phylogeography methods based on the HA dataset for analyzing and inferring the diffusion patterns of the virus over 11 time periods. Lines and dots represent parts of the clades and nodes of the maximum clade credibility (MCC) tree up to each indicated time, with lines and dots colored according to the time (a color scale ranging from red for the oldest to yellow for the youngest). The shaded yellow contours represent statistical uncertainty of the estimated locations at the internal nodes (95% credible contours based on kernel density estimates).

To further elaborate the global dissemination of subC2344b H5Nx viruses in detail, we performed a phylogeographic reconstruction of the HA gene in continuous space. As depicted in the Figure 3, our analysis further revealed the interconnectedness of Africa isolates with lineages in Asia and Europe. Specially, the subC2344b H5Nx viruses remained primarily confined to Eurasia until 2016, and they reached Africa via the West Asian–East African Flyway and the Black Sea–Mediterranean Flyway in May 2016 and December 2016, respectively. Subsequently, the viruses spread southward via the Black Sea–Mediterranean Flyway to South Africa during 2017–2018. From March to May 2019, the viruses might have been moved northward from Africa to East Europe and eventually reached the Russian Federation (Siberia) in early 2020. Since then, subC2344b H5Nx viruses initiated another wave of infections in poultry and wild bird populations across Europe, Africa, and Asia. In October 2021, they spread westward across the Atlantic to North America and thereafter spread southward via Mississippi Americas Flyway to South America.

### 3.3. Host Roles in the Spread of subC2344b H5Nx Viruses

To determine the possible virus dissemination events between different hosts (wild Anseriformes, domestic Galliformes, domestic Anseriformes, Charadriiformes, other wild birds, and mammals), we reconstructed the most likely ancestral host at each internal node in the phylogenetic tree using the Bayesian discrete phylogeography method for the eight external genes (*Supplementary figure 6(a)*, *Supplementary figure 6(b)*, *Supplementary figure 6(c)*, *Supplementary figure 6(d)*, *Supplementary figure 6(e)*, *Supplementary figure 6(f)*, *Supplementary figure 6(g)*, and *Supplementary figure 6(h)*) and six internal genes (*Supplementary figure 6(i)*, *Supplementary figure 6(j)*, *Supplementary figure 6(k)*, *Supplementary figure 6(l)*, and *Supplementary figure 6(m)*) datasets, respectively. We found that wild Anseriformes became the dominant host species for subC2344b H5Nx viruses and were responsible for the viruses spillover to other hosts.

**Figure 4:**
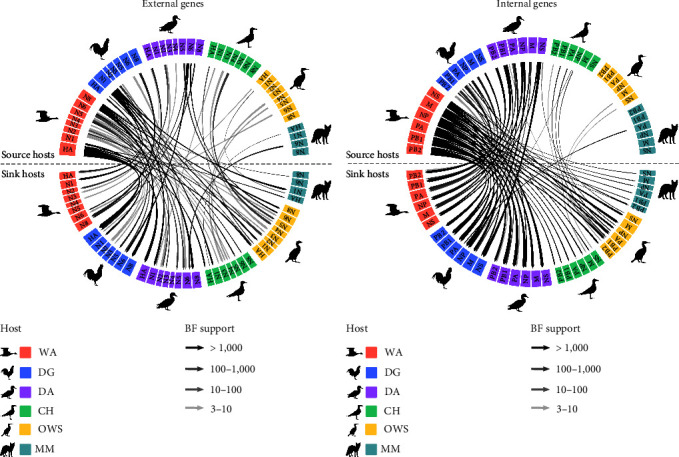
Fully resolved discrete trait diffusion network of gene segments for subC2344b H5Nx host taxa. Chord diagrams depicting the Bayes factor (BF) for viral dissemination among wild Anseriformes = WA, domestic Galliformes = DG, domestic Anseriformes = DA, Charadriiformes = CH, other wild species = OWS, and mammals = MM host taxa of external gene segments (a) and internal gene segments (b). Chord bands represent transition rates from a source host group above the horizontal dashed line to a destination host category below. The magnitude of the viral transition rate is proportional to the width of each arrow. Shaded arrows indicate Bayes factors with statistical support (BF ≥ 3 and PP ≥ 0.5) and the strength of statistical support increasing with intensity of shading. Phylogenetic trees for all 14 gene segments are provided in PDF format in *Supplementary figure 6*, and other comparable information is provided in Excel format in *Supplementary table 7*, *Supplementary table 8*, *Supplementary table 9*, and *Supplementary table 10*. Animal silhouettes were downloaded from PhyloPic (http://PhyloPic.org/) and used under the Creative Commons Attribution 3.0 with a nontransplant license (https://creativecommons.org/licenses/by/3.0/deed.en).

The BSSVS ancestral reconstruction identified 13 supported host switches (BF > 3, PP > 0.95) for the HA genes. Wild Anseriformes and domestic Galliformes were involved in seven and five supported switches, respectively, which suggested that both two hosts played substantial roles in the virus dispersal (Figure 4(a), *Supplementary table 7*). Furthermore, using Markov rewards to estimate the proportion of time the H5 virus spent in each host revealed that wild Anseriformes and domestic Galliformes contributed 42.87% and 25.64% to the transmission of subC2344b H5Nx viruses, respectively (*Supplementary table 8*). Moreover, the most frequent transition and the highest supported transition rate occurred from wild Anseriformes to domestic Galliformes (28.15% Markov jumps, migration rate = 2.97, BF > 1,000, PP = 1) (*Supplementary table 9* and *Supplementary table 10*). By analyzing Markov jumps and total mean migration rates, we found that five species exhibited a distinct inward migration, with the exception of the wild Anseriformes, which displayed a noticeable outward migration.

The dissemination events of different hosts varied slightly across NA subtypes (Figure 4(a), *Supplementary table 7*, *Supplementary table 8*, *Supplementary table 9*, and *Supplementary table 10*). Domestic Galliformes played a crucial role in the migration of the N1 subtype virus, and the total mean rates indicated that they were the dominant exporting host, which showed decisive support (BF > 1,000) to five other hosts. Markov rewards revealed that the N2 subtype virus spent the longest time in domestic Galliformes and domestic Anseriformes. The N3 and N4 subtype viruses spent the longest time in wild Anseriformes, while the large out-migration from the Charadriiformes played a more crucial role in the spread of the viruses. The N5 subtype virus had the longest duration within wild Anseriformes and exhibited an obvious outward migration direction. By summarizing the Markov rewards, Markov jumps, and overall mean migration rates for N6 and N8 subtype viruses, we found that two subtype viruses spent the longest time and exhibited a clear outward migration in domestic Anseriformes and wild Anseriformes, respectively. Notably, the N1 and N8 genes of the viruses provided decisive support for the spillover of domestic Galliformes and wild Anseriformes to mammals, respectively (Figure 4(a), *Supplementary table 7*).

Similarly, a comparable transmission pattern occurred across the six internal genes of the subC2344b H5Nx viruses and provided decisive evidence for the virus spillover from wild Anseriformes to the remaining five hosts (Figure 4(b), *Supplementary table 7*). The Markov rewards indicated that the most time was spent in the wild Anseriformes, followed by domestic Anseriformes and domestic Galliformes (*Supplementary table 8*). Additionally, summarizing the Markov jumps and total mean migration rates of the viruses, it can be seen that the wild Anseriformes exhibited a distinct outward migration, while the domestic Galliformes (PB2, PB1, NP, and NS) and domestic Anseriformes (PA and M) displayed a clear inward migration (*Supplementary table 9* and *Supplementary table 10*). It was notable that six internal genes of the virus existed strong or even decisively support transmission from wild Anseriformes to mammals. Furthermore, we also observed bidirectional transmission of the virus between wild Anseriformes and domestic Galliformes, between wild Anseriformes and domestic Anseriformes, and between domestic Galliformes and domestic Anseriformes. Therefore, we hypothesized that frequent transmission events among these species might have enhanced the divergence of subC2344b H5Nx viruses at the wild–domestic interface.

**Figure 5:**
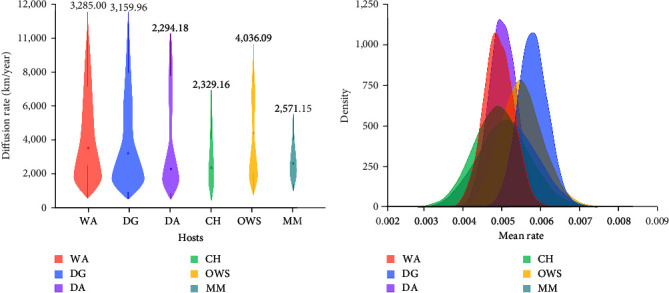
Contribution of different host taxa to subC2344b H5Nx dissemination. (a) The posterior diffusion rate distribution for each host obtained by the joint host analysis of the HA dataset. Posterior diffusion rates for each host are provided above each violin plot. (b) Comparison of nucleotide substitution rates of viruses within different host (from left to right: light coral, WA; light green, CH; orchid, DA; light sea green, MM; tuscan sun, OWS; cornflower blue, DG; in the online color figures.).

To help elucidate the host contributions in the spatial expansion of the subC2344b H5Nx viruses, we conducted a joint analysis of continuous spatial and discrete host traits (*Supplementary figure 11*). The results demonstrated that wild Anseriformes (3285.00 km/year) dispersed the viruses more rapidly and played a critical role for viral transmission compared to the four host taxa (Figure 5(a)). This finding may reflect their long-distance, pelagic movements as well as their immune-naïve status against the virus, contributing to both spatial expansion and rapid diffusion globally. The estimated transmission rates of domestic Galliformes (3159.96 km/year) were slightly lower than that of wild Anseriformes, which also contributed significantly to virus diffusion. Interestingly, the spread by domestic Anseriformes (2294.18 km/year) was the slowest among these hosts. We then further estimated the nucleotide substitution rates of viruses in different hosts (Figure 5(b)). The results indicated that the fastest virus evolution rate happened in domestic Galliformes (5.71 × 10^−3^ substitutions/site/year, 95% HPD: 4.95–6.42 × 10^−3^), while similar evolution rates happened between the domestic Anseriformes (4.95 × 10^−3^ substitutions/site/year, 95% HPD: 4.28–5.62 × 10^−3^) and the wild Anseriformes (4.84 × 10^−3^ substitutions/site/year, 95% HPD: 4.10–5.57 × 10^−3^). This finding might be attributed to the high density of intensive breeding of domestic poultry. However, due to limited sampling of mammalian sequences in the public database and the high variability among the other wild bird species, it is difficult to assess their potential roles in virus diffusion.

### 3.4. Evolutionary Parameter Contrasting of Two Main Clusters in HA Gene of subC2344b H5Nx Viruses

According to the phylogenetic analysis, the subC2344b H5Nx viruses were divided into two main clusters, named Cluster A and Cluster B (Table 1). Here, we conducted a comparative Bayesian analysis of these two clusters to better understand their evolutionary relationship. Our data showed that the average rates of nucleotide substitution of Cluster B (5.07 × 10^−3^ substitutions per site per year) were higher than that of Cluster A (3.65 × 10^−3^ substitutions per site per year). The divergence time of Cluster A (tMRCA = 2015.447, 95% HPD: 2014.93–2015.89) was earlier than Cluster B (tMRCA = 2015.919, 95% HPD: 2015.64–2016.11). Regionally, the rate of region switching events in the evolutionary history of Cluster B (0.40 region switches/unit time) was 1.78 times higher than that of Cluster A (0.22 region switches/unit time). The discrete phylogeographic analysis demonstrated that both two clusters most likely originated from the Russian Federation (Siberia), with Cluster A and Cluster B prevalent longer in Africa and Russian Federation (Siberia), respectively. As for host, the host switching of Cluster B (0.36 host switches/unit time) were 1.51 times higher than that of Cluster A (0.24 host switches/unit time). Although both of Cluster A and Cluster B most likely originated from the wild Anseriformes, Cluster A is prevalent longer in the domestic Galliformes, while Cluster B is prevalent longer in the wild Anseriformes. In conclusion, Cluster B exhibited a relatively faster evolution rate comparing to Cluster A.

## 4. Discussion

To effectively prevent and control the HPAI depend largely on comprehensive understanding the spatiotemporal spreading and transmission dynamics of the HPAI viruses. In this study, we conducted a phylogenetic and epidemiological analysis of the 4,778 HA gene sequences H5Nx viruses belonging to subC2344b existing between 2013 and 2023, which revealed that the HA gene of these viruses diverged into two dominant clusters around 2015 and 2016, exhibiting geographical and host range expansion and recombination with different NA genes. Furthermore, we employed the Bayesian phylodynamic model to investigate their spatiotemporal spread and evolution, which provided new insights into the intercontinental dissemination dynamics of the viruses as well as the potential contribution of different hosts to the viral invasion guided by phylogeographic approach.

Our findings showed that the Russian Federation (Siberia) region was the main communication center of virus transmission, introducing the virus to some Asia and Europe regions and receiving the backflow of virus from Africa region. This finding was consistent with the fact that multiple migration pathways intersect in the Siberia; the region is also a significant breeding ground for wild birds in Eurasia [48]. To better understand this correlation, it becomes necessary to determine the overlap in the Siberia breeding areas of long-distance migratory birds wintering in Europe, Asia, Africa, and North America by technical means such as satellite telemetry. Strong epidemiological links between West Europe and East Europe, Asia, Africa, and North America regions further facilitated to the virus dissemination, while Korea may become the third communication center in the Asia region during the later stages of the epidemic. Despite the subC2344b H5Nx viruses were first detected in Asia and had long been recognized as the source for viruses maintaining with seasonal long-distance transmission to Europe through migratory birds, increasingly comprehensive epidemiological surveillance studies in wild birds indicated that the viruses have been evolved diversely in different regions and dispersed globally via multiple pathways. For instance, subC2344b H5N8 viruses detected in Europe in early 2020 were genetically close to the viruses detected in Africa during 2018–2019, whereas the H5N8 viruses detected in Asia in late 2020 had the similar lineage as the viruses detected in Europe in early 2020. It was reported that the “quiet” period of 2019–2020 provided ample time for subC2344b H5N8 viruses to reassort and evolve into new variants, and the widespread occurrence of new H5N8 variants and spatiotemporal genotype replacement contributed to subC2344b H5N1 emergence in 2021–2022 [49]. Moreover, the new subC2344b H5N1 reassortment viruses had spread by migratory birds to various countries in Africa, Asia, and North America. Here, our findings provide a complement to the confirmation that a shift in the epicenter of subC2344b H5Nx viruses has occurred from Asia to Europe and to America, emphasizing the need to expand the surveillance capacity.

We compared the evolutionary dynamics of subC2344b H5Nx viruses in different host taxa systematically; our findings provide compelling evidence that wild Anseriformes play a critical role for viruses spread and transmission, which contribute to its spatial expansion and rapid diffusion; moreover, it was also identified as primary source hosts for the virus evolution and responsible for the species-crossing transmission, leading to a variety of the other host taxa infected. The wild Anseriformes exhibited a complex population structure with the viruses were mostly detected in species of Anser (382/1131), Cygnus (364/1131), and Anas (301/1131). Studies have shown that these species either act as sentinels in specific areas or migrate partially or completely to other regions, serving as long-distance vectors of the virus [50]. Experimental evidence of prolonged virus shedding in Anser led bar-headed geese recognized as remarkable host for clade 2.2 virus transmission along migration routes in Central Asia in 2005 [51]. With addition of the breeding populations, the Anser has witnessed a significant increase throughout the American and Eurasian continents, indicating that Anser may be an important as yet poorly understood reservoir [52, 53, 54]. In contrast, the Cygnus is relatively more susceptible, has higher mortality rates, and is considered an indicator species for transborder spreading and monitoring [55, 56, 57]. Moreover, experimental findings in Anas such as mallard and Eurasian wigeon provide the strongest evidence that they serve as long-distance vectors of subC2344b H5Nx viruses [11, 58, 59].

It is noteworthy that subC2344b H5Nx viruses evolved into two clusters, but not parallelly with Cluster A appeared earlier than Cluster B but evolved at a slower rate and at a marked decrease in frequency in the system. In addition to this phenomenon being related to competitive interactions between clusters, the migratory behavior of bird hosts and their unique geographic distribution may contribute to the maintenance of two clusters in different host populations. Significantly, animal experimental data indicate that the virulence of Cluster A is weaker than that of Cluster B in ducks and mice [24], which suggests that Cluster A is likely not go extinct, but instead circulating among some asymptomatic host species that are not easily and closely monitored.

Frequent spillover of subC2344b H5Nx viruses from the wild bird to domestic bird and mammals has been observed. Many countries in Europe and North America control and stamp out the HPAI by culling the infected and susceptible birds [60, 61, 62]. However, the culling policy for poultry is not the best choice and mass culling of poultry resulting in a shortage of meat and eggs. “Cull plus vaccination” has been the main strategy for the control and prevention of the HPAI virus in China about 20 years, and the vaccination of poultry successfully eliminated human infection with H7N9 virus [5]. Notably, the H5N8 HPAI viruses were widely spread only among wild birds and domestic waterfowl in China, whereas there was no case occurred in our chickens, even the neighboring countries reported poultry outbreak occasionally [24, 56]. Hence, we strongly suggest to employ the vaccination policy to protect the poultry industry from the worldwide epizootic of H5N1 HPAI, as well as the public health safety.

This study has several limitations. First, while we believe that our subsampling strategy balanced groupings as much as possible in preserving viral, host, and temporal diversity across all regions, it does not completely eliminate sampling bias, which may have affected the Bayesian phylogenic reconstruction and inference of transmission networks. Second, surveillance, sequencing efforts, and commitments to publish sequences vary among countries, and the data accumulated in database may not fully reflect the phenomena occurring in nature.

In conclusion, our findings provide novel insights into the spatiotemporal analysis and species-crossing transmission of subC2344b H5Nx HPAIVs globally. With the continuous evolution and increasing geographic distributions of subC2344b H5N1 HPAI viruses, it is necessary to strengthen continuing local and global surveillance and to improve multifaceted mitigation strategies for outbreak prevention and response to prevent the H5N1 virus epidemic from becoming a pandemic.

## Figures and Tables

**Table 1:** Evolutionary parameter contrasting of two main clusters in subC2344b H5Nx viruses.

Discrete variable	Cluster A	Cluster B
Nucleotide substitution mean rate (95% HPD^a^)	3.65 × 10^−3^ (2.84 × 10^−3^−4.48 × 10^−3^)	5.07 × 10^−3^ (4.53 × 10^−3^−5.61 × 10^−3^)
Time of most recent common ancestor (95% HPD)	2,015.45 (2,014.93–2,015.89)	2,015.92 (2,015.64–2,016.11)
Location		
Mean rate (95% HPD)	0.22 (0.11–0.35)	0.40 (0.32–0.48)
Root (PP^b^)	RUS (0.98)	RUS (1.00)
Maximum rewards (rewards, 95% HPD)	AF (12.62, 8.81–16.77)	RUS (34.44, 32.08–41.14)
Host		
Mean rate (95% HPD)	0.24 (0.11–0.38)	0.36 (0.28–0.44)
Root (PP^b^)	WA (0.99)	WA (1.00)
Maximum rewards (rewards, 95% HPD)	DG (28.20, 20.01–35.74)	WA (67.32, 57.55–76.23)

^a^HPD represents the highest posterior density. ^b^posterior probabilities.

## Data Availability

The data used to support the findings of this study are included within the article and in the supplementary files.

## Supplementary Materials

Supplementary 1 Table 1: virus information downloaded from GISAID and detailed classifications.

Supplementary 2 Figure 1: tempest of subC2344b H5Nx viruses.

Supplementary 3 Table 2: Bayes factor supports between regions.

Supplementary 4 Table 3: Markov jumps for individual transitions between regions.

Supplementary 5 Table 4: migration rate for individual transitions between regions.

Supplementary 6 Figure 2: MCC tree of eight genes of subC2344b H5Nx viruses.

Supplementary 7 Table 5: Bayes factor supports between hosts.

Supplementary 8 Table 6: Markov rewards for time spent at each host.

Supplementary 9 Table 7: Markov jumps for individual transitions between hosts.

Supplementary 10 Table 8: migration rate for individual transitions between hosts.

Supplementary 11 Figure 3: joint inference of host and region.
