# Supplementary material for: Spatiotemporal and Species-Crossing Transmission Dynamics of Subclade 2.3.4.4b H5Nx HPAIVs
Source: Transbound Emerg Dis. 2024 Jul 10;2024:2862053. doi: 10.1155/2024/2862053 (PMC12017169; doi:10.1155/2024/2862053)
Supplement: Supplementary 1 — Table 1: virus information downloaded from GISAID and detailed classifications. [file 2862053.f1.docx]

**Table S1.** Virus information downloaded from GISAID and their classifications in the study.

| **Virus name** | **Year** | **State** | **Host** | **Subtype** |
| --- | --- | --- | --- | --- |
| A/common teal/Shanghai/PD1108-13/2013(H5N8) | Early 2015 | China | Wild Anseriformes | H5N8 |
| A/common teal/Shanghai/PD1108-20/2013(H5N8) | Early 2015 | China | Wild Anseriformes | H5N8 |
| A/common teal/Shanghai/PD1108-24/2013(H5N8) | Early 2015 | China | Wild Anseriformes | H5N8 |
| A/common teal/Shanghai/PD1108-25/2013(H5N8) | Early 2015 | China | Wild Anseriformes | H5N8 |
| A/common teal/Shanghai/PD1108-27/2013(H5N8) | Early 2015 | China | Wild Anseriformes | H5N8 |
| A/common teal/Shanghai/PD1108-3/2013(H5N8) | Early 2015 | China | Wild Anseriformes | H5N8 |
| A/common teal/Shanghai/PD1108-6/2013(H5N8) | Early 2015 | China | Wild Anseriformes | H5N8 |
| A/goose/Eastern China/CZ/2013(H5N8) | Early 2015 | China | Domestic Anseriformes | H5N8 |
| A/mallard/Shanghai/SH-9/2013(H5N8) | Early 2015 | China | Wild Anseriformes | H5N8 |
| A/duck/Shandong/11.14 DL-DKd11/2013(H5N8) | Early 2015 | China | Domestic Anseriformes | H5N8 |
| A/duck/Shandong/11.14 RD/2013(H5N8) | Early 2015 | China | Domestic Anseriformes | H5N8 |
| A/duck/Zhejiang/W24/2013(H5N8) | Early 2015 | China | Domestic Anseriformes | H5N8 |
| A/common teal/Shanghai/PD1118-1/2013(H5N8) | Early 2015 | China | Wild Anseriformes | H5N8 |
| A/mallard duck/Shanghai/SH-9/2013(H5N8) | Early 2015 | China | Wild Anseriformes | H5N8 |
| A/common teal/Shanghai/PD1121-10/2013(H5N8) | Early 2015 | China | Wild Anseriformes | H5N8 |
| A/common teal/Shanghai/PD1121-15/2013(H5N8) | Early 2015 | China | Wild Anseriformes | H5N8 |
| A/common teal/Shanghai/PD1121-18/2013(H5N8) | Early 2015 | China | Wild Anseriformes | H5N8 |
| A/common teal/Shanghai/PD1121-19/2013(H5N8) | Early 2015 | China | Wild Anseriformes | H5N8 |
| A/falcated duck/Shanghai/PD1121-26/2013(H5N8) | Early 2015 | China | Wild Anseriformes | H5N8 |
| A/falcated duck/Shanghai/PD1121-27/2013(H5N8) | Early 2015 | China | Wild Anseriformes | H5N8 |
| A/common teal/Shanghai/PD1202-9/2013(H5N8) | Early 2015 | China | Wild Anseriformes | H5N8 |
| A/spot-billed duck/Shanghai/PD1202-3/2013(H5N8) | Early 2015 | China | Wild Anseriformes | H5N8 |
| A/duck/Eastern China/S1210/2013(H5N8) | Early 2015 | China | Domestic Anseriformes | H5N8 |
| A/duck/Zhejiang/6D18/2013(H5N8) | Early 2015 | China | Domestic Anseriformes | H5N8 |
| A/goose/Shandong/GD-GS/2014(H5N8) | Early 2015 | China | Domestic Anseriformes | H5N8 |
| A/duck/Eastern China/JY/2014(H5N8) | Early 2015 | China | Domestic Anseriformes | H5N8 |
| A/breeder duck/Korea/Gochang1/2014(H5N8) | Early 2015 | Korea | Domestic Anseriformes | H5N8 |
| A/Baikal teal/Korea/H52/2014(H5N8) | Early 2015 | Korea | Wild Anseriformes | H5N8 |
| A/duck/Eastern China/S0215/2014(H5N8) | Early 2015 | China | Domestic Anseriformes | H5N8 |
| A/goose/Jiangsu/WX202/2014(H5N8) | Early 2015 | China | Domestic Anseriformes | H5N8 |
| A/goose/Jiangsu/QD5/2014(H5N8) | Early 2015 | China | Domestic Anseriformes | H5N8 |
| A/goose/Eastern China/S0408/2014(H5N8) | Early 2015 | China | Domestic Anseriformes | H5N8 |
| A/goose/Shandong/WFSG1/2014(H5N8) | Early 2015 | China | Domestic Anseriformes | H5N8 |
| A/goose/Yangzhou/0420/2014(H5N8) | Early 2015 | China | Domestic Anseriformes | H5N8 |
| A/duck/Liaoning/S1001/2014(H5N8) | Early 2015 | China | Domestic Anseriformes | H5N8 |
| A/goose/Shandong/05.23 DT-GS/2014(H5N8) | Early 2015 | China | Domestic Anseriformes | H5N8 |
| A/goose/Shandong/06.14 HZ-GS/2014(Mixed) | Early 2015 | China | Domestic Anseriformes | H5N8 |
| A/goose/Zhejiang/77166/2014(H5N2) | Early 2015 | China | Domestic Anseriformes | H5N2 |
| A/Von Schrencks bittern/Jiangxi/Y9/2014(H5N8) | Early 2015 | China | Other wild species | H5N8 |
| A/duck/Zhejiang/925019/2014(H5N8) | Early 2015 | China | Domestic Anseriformes | H5N8 |
| A/duck/Zhejiang/925169/2014(H5N8) | Early 2015 | China | Domestic Anseriformes | H5N8 |
| A/goose/Zhejiang/925037/2014(H5N8) | Early 2015 | China | Domestic Anseriformes | H5N8 |
| A/goose/Zhejiang/925104/2014(H5N8) | Early 2015 | China | Domestic Anseriformes | H5N8 |
| A/goose/Zhejiang/925106/2014(H5N6) | Early 2015 | China | Domestic Anseriformes | H5N6 |
| A/Chicken/Changzhou/cz93/2014(H5N8) | Early 2015 | China | Domestic Galliformes | H5N8 |
| A/duck/Eastern China/S1109/2014(H5N8) | Early 2015 | China | Domestic Anseriformes | H5N8 |
| A/goose/Shandong/12.08 YG-GS/2014(H5N8) | Early 2015 | China | Domestic Anseriformes | H5N8 |
| A/goose/Shandong/05.23 AY-GS/2015(Mixed) | Early 2015 | China | Domestic Anseriformes | H5N8 |
| A/quail/Jiangxi/B9/2015 H5N6(H5N6) | Early 2015 | China | Domestic Galliformes | H5N6 |
| A/domestic duck/Siberia/49 feather/2016(H5N8) | 2016-2017 | Russian Federation Siberia | Domestic Anseriformes | H5N8 |
| A/Bar-headed Goose/Qinghai/HDT001/2016(H5N8) | 2016-2017 | China | Wild Anseriformes | H5N8 |
| A/Brown-headed Gull/Qinghai/ZTO1-B/2016(H5N8) | 2016-2017 | China | Charadriiformes | H5N8 |
| A/Brown-headed Gull/Qinghai/ZTO1-LU/2016(H5N8) | 2016-2017 | China | Charadriiformes | H5N8 |
| A/Bar-headed Goose/Qinghai/BTY10-B/2016(H5N8) | 2016-2017 | China | Wild Anseriformes | H5N8 |
| A/Bar-headed Goose/Qinghai/BTY10-LU/2016(H5N8) | 2016-2017 | China | Wild Anseriformes | H5N8 |
| A/Bar-headed Goose/Qinghai/BTY11-B/2016(H5N8) | 2016-2017 | China | Wild Anseriformes | H5N8 |
| A/Bar-headed Goose/Qinghai/BTY11-LU/2016(H5N8) | 2016-2017 | China | Wild Anseriformes | H5N8 |
| A/Bar-headed Goose/Qinghai/BTY1-B/2016(H5N8) | 2016-2017 | China | Wild Anseriformes | H5N8 |
| A/Bar-headed Goose/Qinghai/BTY1-LV/2016(H5N8) | 2016-2017 | China | Wild Anseriformes | H5N8 |
| A/Bar-headed Goose/Qinghai/BTY9-B/2016(H5N8) | 2016-2017 | China | Wild Anseriformes | H5N8 |
| A/Bar-headed Goose/Qinghai/BTY9-LU/2016 (H5N8) | 2016-2017 | China | Wild Anseriformes | H5N8 |
| A/Bar-headed Goose/Qinghai/BTY8-B/2016(H5N8) | 2016-2017 | China | Wild Anseriformes | H5N8 |
| A/Bar-headed Goose/Qinghai/BTY8-LU/2016(H5N8) | 2016-2017 | China | Wild Anseriformes | H5N8 |
| A/Bar-headed Goose/Qinghai/BTY6-B/2016(H5N8) | 2016-2017 | China | Wild Anseriformes | H5N8 |
| A/Bar-headed Goose/Qinghai/BTY6-LU/2016(H5N8) | 2016-2017 | China | Wild Anseriformes | H5N8 |
| A/Bar-headed Goose/Qinghai/BTY7-B/2016(H5N8) | 2016-2017 | China | Wild Anseriformes | H5N8 |
| A/Bar-headed Goose/Qinghai/BTY7-LU1/2016(H5N8) | 2016-2017 | China | Wild Anseriformes | H5N8 |
| A/Bar-headed Goose/Qinghai/BTY7-LU2/2016(H5N8) | 2016-2017 | China | Wild Anseriformes | H5N8 |
| A/Brown-headed Gull/Qinghai/ZTO4-B/2016(H5N8) | 2016-2017 | China | Charadriiformes | H5N8 |
| A/Bar-headed Goose/Qinghai/a15/2016(H5N8) | 2016-2017 | China | Wild Anseriformes | H5N8 |
| A/Bar-headed Goose/Qinghai/a27/2016(H5N8) | 2016-2017 | China | Wild Anseriformes | H5N8 |
| A/Bar-headed Goose/Qinghai/a32/2016(H5N8) | 2016-2017 | China | Wild Anseriformes | H5N8 |
| A/Bar-headed Goose/Qinghai/a88/2016(H5N8) | 2016-2017 | China | Wild Anseriformes | H5N8 |
| A/Bar-headed Goose/Qinghai/BTY2-B/2016(H5N8) | 2016-2017 | China | Wild Anseriformes | H5N8 |
| A/Bar-headed Goose/Qinghai/BTY2-LU/2016(H5N8) | 2016-2017 | China | Wild Anseriformes | H5N8 |
| A/Bar-headed Goose/Qinghai/BTY3-B/2016(H5N8) | 2016-2017 | China | Wild Anseriformes | H5N8 |
| A/Bar-headed Goose/Qinghai/BTY3-LU/2016(H5N8) | 2016-2017 | China | Wild Anseriformes | H5N8 |
| A/Bar-headed Goose/Qinghai/BTY4-B/2016(H5N8) | 2016-2017 | China | Wild Anseriformes | H5N8 |
| A/Bar-headed Goose/Qinghai/BTY4-LU/2016(H5N8) | 2016-2017 | China | Wild Anseriformes | H5N8 |
| A/Bar-headed Goose/Qinghai/BTY5-B/2016(H5N8) | 2016-2017 | China | Wild Anseriformes | H5N8 |
| A/Bar-headed Goose/Qinghai/XX111/2016(H5N8) | 2016-2017 | China | Wild Anseriformes | H5N8 |
| A/Bar-headed Goose/Qinghai/XX13/2016(H5N8) | 2016-2017 | China | Wild Anseriformes | H5N8 |
| A/Bar-headed Goose/Qinghai/XX22/2016(H5N8) | 2016-2017 | China | Wild Anseriformes | H5N8 |
| A/Bar-headed Goose/Qinghai/XX76/2016(H5N8) | 2016-2017 | China | Wild Anseriformes | H5N8 |
| A/Brown-headed Gull/Qinghai/ZTO3-B/2016(H5N8) | 2016-2017 | China | Charadriiformes | H5N8 |
| A/Brown-headed Gull/Qinghai/ZTO3-LU/2016(H5N8) | 2016-2017 | China | Charadriiformes | H5N8 |
| A/Bar-headed Goose/Qinghai/a114/2016(H5N8) | 2016-2017 | China | Wild Anseriformes | H5N8 |
| A/Bar-headed Goose/Qinghai/A16/2016(H5N8) | 2016-2017 | China | Wild Anseriformes | H5N8 |
| A/Bar-headed Goose/Qinghai/A17/2016(H5N8) | 2016-2017 | China | Wild Anseriformes | H5N8 |
| A/Bar-headed Goose/Qinghai/A22/2016(H5N8) | 2016-2017 | China | Wild Anseriformes | H5N8 |
| A/Bar-headed Goose/Qinghai/A23/2016(H5N8) | 2016-2017 | China | Wild Anseriformes | H5N8 |
| A/Bar-headed Goose/Qinghai/a24/2016(H5N8) | 2016-2017 | China | Wild Anseriformes | H5N8 |
| A/Bar-headed Goose/Qinghai/a26/2016(H5N8) | 2016-2017 | China | Wild Anseriformes | H5N8 |
| A/Bar-headed Goose/Qinghai/a43/2016(H5N8) | 2016-2017 | China | Wild Anseriformes | H5N8 |
| A/Bar-headed Goose/Qinghai/a45/2016(H5N8) | 2016-2017 | China | Wild Anseriformes | H5N8 |
| A/Bar-headed Goose/Qinghai/BTY12-B/2016 (H5N8) | 2016-2017 | China | Wild Anseriformes | H5N8 |
| A/Bar-headed Goose/Qinghai/BTY12-LU/2016(H5N8) | 2016-2017 | China | Wild Anseriformes | H5N8 |
| A/Bar-headed Goose/Qinghai/p2/2016(H5N8) | 2016-2017 | China | Wild Anseriformes | H5N8 |
| A/Bar-headed Goose/Qinghai/p9/2016(H5N8) | 2016-2017 | China | Wild Anseriformes | H5N8 |
| A/Bar-headed Goose/Qinghai/A11/2016(H5N8) | 2016-2017 | China | Wild Anseriformes | H5N8 |
| A/Bar-headed Goose/Qinghai/a113/2016(H5N8) | 2016-2017 | China | Wild Anseriformes | H5N8 |
| A/Bar-headed Goose/Qinghai/a115/2016(H5N8) | 2016-2017 | China | Wild Anseriformes | H5N8 |
| A/Bar-headed Goose/Qinghai/A12/2016(H5N8) | 2016-2017 | China | Wild Anseriformes | H5N8 |
| A/Bar-headed Goose/Qinghai/A13/2016(H5N8) | 2016-2017 | China | Wild Anseriformes | H5N8 |
| A/Bar-headed Goose/Qinghai/A19/2016(H5N8) | 2016-2017 | China | Wild Anseriformes | H5N8 |
| A/Bar-headed Goose/Qinghai/A20/2016(H5N8) | 2016-2017 | China | Wild Anseriformes | H5N8 |
| A/Bar-headed Goose/Qinghai/A21/2016(H5N8) | 2016-2017 | China | Wild Anseriformes | H5N8 |
| A/Bar-headed Goose/Qinghai/BTY13-B/2016(H5N8) | 2016-2017 | China | Wild Anseriformes | H5N8 |
| A/Bar-headed Goose/Qinghai/BTY13-LU/2016(H5N8) | 2016-2017 | China | Wild Anseriformes | H5N8 |
| A/Bar-headed Goose/Qinghai/BTY14-B/2016(H5N8) | 2016-2017 | China | Wild Anseriformes | H5N8 |
| A/Bar-headed Goose/Qinghai/BTY14-LU/2016(H5N8) | 2016-2017 | China | Wild Anseriformes | H5N8 |
| A/Bar-headed Goose/Qinghai/BTY15-B/2016(H5N8) | 2016-2017 | China | Wild Anseriformes | H5N8 |
| A/Bar-headed Goose/Qinghai/BTY15-LU/2016(H5N8) | 2016-2017 | China | Wild Anseriformes | H5N8 |
| A/Bar-headed Goose/Qinghai/BTY16-B/2016(H5N8) | 2016-2017 | China | Wild Anseriformes | H5N8 |
| A/Bar-headed Goose/Qinghai/BTY16-LU/2016(H5N8) | 2016-2017 | China | Wild Anseriformes | H5N8 |
| A/Bar-headed Goose/Qinghai/XX431/2016(H5N8) | 2016-2017 | China | Wild Anseriformes | H5N8 |
| A/Bar-headed Goose/Qinghai/XX446/2016(H5N8) | 2016-2017 | China | Wild Anseriformes | H5N8 |
| A/Bar-headed Goose/Qinghai/XX782/2016(H5N8) | 2016-2017 | China | Wild Anseriformes | H5N8 |
| A/Brown-headed Gull/Qinghai/ZTO5-B/2016(H5N8) | 2016-2017 | China | Charadriiformes | H5N8 |
| A/Brown-headed Gull/Qinghai/ZTO5-K/2016(H5N8) | 2016-2017 | China | Charadriiformes | H5N8 |
| A/Bar-headed Goose/Qinghai/a61/2016(H5N8) | 2016-2017 | China | Wild Anseriformes | H5N8 |
| A/Bar-headed Goose/Qinghai/a91/2016(H5N8) | 2016-2017 | China | Wild Anseriformes | H5N8 |
| A/Bar-headed Goose/Qinghai/a92/2016(H5N8) | 2016-2017 | China | Wild Anseriformes | H5N8 |
| A/Bar-headed Goose/Qinghai/a93/2016(H5N8) | 2016-2017 | China | Wild Anseriformes | H5N8 |
| A/Bar-headed Goose/Qinghai/B11/2016(H5N8) | 2016-2017 | China | Wild Anseriformes | H5N8 |
| A/Bar-headed Goose/Qinghai/B12/2016(H5N8) | 2016-2017 | China | Wild Anseriformes | H5N8 |
| A/Bar-headed Goose/Qinghai/B34/2016(H5N8) | 2016-2017 | China | Wild Anseriformes | H5N8 |
| A/Bar-headed Goose/Qinghai/B44/2016(H5N8) | 2016-2017 | China | Wild Anseriformes | H5N8 |
| A/Bar-headed Goose/Qinghai/B51/2016(H5N8) | 2016-2017 | China | Wild Anseriformes | H5N8 |
| A/Bar-headed Goose/Qinghai/B54/2016(H5N8) | 2016-2017 | China | Wild Anseriformes | H5N8 |
| A/Bar-headed Goose/Qinghai/B7/2016(H5N8) | 2016-2017 | China | Wild Anseriformes | H5N8 |
| A/Bar-headed Goose/Qinghai/BTY17-B/2016 (H5N8) | 2016-2017 | China | Wild Anseriformes | H5N8 |
| A/Bar-headed Goose/Qinghai/BTY17-LU/2016(H5N8) | 2016-2017 | China | Wild Anseriformes | H5N8 |
| A/Bar-headed Goose/Qinghai/BTY18-B/2016(H5N8) | 2016-2017 | China | Wild Anseriformes | H5N8 |
| A/Bar-headed Goose/Qinghai/BTY18-LU/2016 (H5N8) | 2016-2017 | China | Wild Anseriformes | H5N8 |
| A/Bar-headed Goose/Qinghai/p18/2016(H5N8) | 2016-2017 | China | Wild Anseriformes | H5N8 |
| A/Bar-headed Goose/Qinghai/XXI122/2016(H5N8) | 2016-2017 | China | Wild Anseriformes | H5N8 |
| A/Brown-headed Gull/Qinghai/ZTO6-B/2016(H5N8) | 2016-2017 | China | Charadriiformes | H5N8 |
| A/Brown-headed Gull/Qinghai/ZTO6-MU/2016(H5N8) | 2016-2017 | China | Charadriiformes | H5N8 |
| A/Brown-headed Gull/Qinghai/ZTO6-SP/2016(H5N8) | 2016-2017 | China | Charadriiformes | H5N8 |
| A/Great Black-headed Gull/Qinghai/YO1-B/2016(H5N8) | 2016-2017 | China | Charadriiformes | H5N8 |
| A/black-headed gull/Tyva/41/2016(H5N8) | 2016-2017 | Russian Federation Siberia | Charadriiformes | H5N8 |
| A/common tern /Uvs-Nuur Lake/26/2016(H5N8) | 2016-2017 | Russian Federation Siberia | Other wild species | H5N1 |
| A/great crested grebe/Tyva/34/2016(H5N8) | 2016-2017 | Russian Federation Siberia | Other wild species | H5N8 |
| A/great crested grebe/Uvs-Nuur Lake/341/2016(H5N8) | 2016-2017 | Russian Federation Siberia | Other wild species | H5N8 |
| A/grey heron /Uvs-Nuur Lake/20/2016(H5N8) | 2016-2017 | Russian Federation Siberia | Other wild species | H5N8 |
| A/wild duck/Tyva/35/2016(H5N8) | 2016-2017 | Russian Federation Siberia | Wild Anseriformes | H5N8 |
| A/Great Cormorant/Qinghai/a51/2016(H5N8) | 2016-2017 | China | Other wild species | H5N8 |
| A/Great Cormorant/Qinghai/B82/2016(H5N8) | 2016-2017 | China | Other wild species | H5N8 |
| A/Great Cormorant/Qinghai/Y01/2016(H5N8) | 2016-2017 | China | Other wild species | H5N8 |
| A/gadwall/Kurgan/2442/2016(H5N8) | 2016-2017 | Russian Federation Siberia | Wild Anseriformes | H5N8 |
| A/Luscinia cyane/Jiangxi/U2/2016 H5N6(H5N6) | 2016-2017 | China | Other wild species | H5N6 |
| A/wild duck/Tatarstan/3059/2016(H5N8) | 2016-2017 | Russian Federation Siberia | Wild Anseriformes | H5N8 |
| A/domestic duck/Siberia/103/2016(H5N8) | 2016-2017 | Russian Federation Siberia | Domestic Anseriformes | H5N8 |
| A/domestic duck/Siberia/50K/2016(H5N8) | 2016-2017 | Russian Federation Siberia | Domestic Anseriformes | H5N8 |
| A/duck/India/10CA01/2016(H5N8) | 2016-2017 | West Central Asia | Domestic Anseriformes | H5N8 |
| A/mute swan/Hungary/51049/2016(H5N8) | 2016-2017 | East Europe | Wild Anseriformes | H5N8 |
| A/painted stork/India/10CA03/2016(H5N8) | 2016-2017 | West Central Asia | Other wild species | H5N8 |
| A/mute swan/Croatia/70/2016(H5N8) | 2016-2017 | East Europe | Wild Anseriformes | H5N8 |
| A/tufted duck/Switzerland/V237-L02003/2016(H5N8) | 2016-2017 | West Europe | Wild Anseriformes | H5N8 |
| A/tufted duck/Switzerland/V246-L02001/2016(H5N8) | 2016-2017 | West Europe | Wild Anseriformes | H5N8 |
| A/tufted duck/Switzerland/V254-L02002/2016(H5N8) | 2016-2017 | West Europe | Wild Anseriformes | H5N8 |
| A/Turkey/Hungary/53136/2016(H5N8) | 2016-2017 | East Europe | Domestic Galliformes | H5N8 |
| A/domestic turkey/Hungary/53433/2016(H5N8) | 2016-2017 | East Europe | Domestic Galliformes | H5N8 |
| A/wild duck/Poland/82A/2016(H5N8) | 2016-2017 | East Europe | Wild Anseriformes | H5N8 |
| A/tufted duck/Germany/AR8444-L01986/2016(H5N8) | 2016-2017 | West Europe | Wild Anseriformes | H5N8 |
| A/tufted duck/Germany/AR8444-L01987/2016(H5N8) | 2016-2017 | West Europe | Wild Anseriformes | H5N8 |
| A/tufted duck/Germany-SH/R8444/2016(H5N8) | 2016-2017 | West Europe | Wild Anseriformes | H5N8 |
| A/tufted duck/Germany-SH/R8446/2016(H5N8) | 2016-2017 | West Europe | Wild Anseriformes | H5N8 |
| A/wild duck/Germany-BW/R8455/2016(H5N8) | 2016-2017 | West Europe | Wild Anseriformes | H5N8 |
| A/Duck/France/RG1/2016 (H5N8) | 2016-2017 | West Europe | Domestic Anseriformes | H5N8 |
| A/G c grebe/NL-Monnickendam/16013865-009-010/2016(H5N8) | 2016-2017 | West Europe | Other wild species | H5N8 |
| A/Mulard duck/Hungary/54494/2016(H5N8) | 2016-2017 | East Europe | Wild Anseriformes | H5N8 |
| A/T Dk/NL-Monnickendam/16013865-006-008/2016(H5N8) | 2016-2017 | West Europe | Wild Anseriformes | H5N8 |
| A/tufted duck/Germany/AR8459-L01988/2016(H5N8) | 2016-2017 | West Europe | Wild Anseriformes | H5N8 |
| A/Duck/Hungary/54738/2016(H5N8) | 2016-2017 | East Europe | Domestic Anseriformes | H5N8 |
| A/herring gull/Poland/84/2016(H5N8) | 2016-2017 | East Europe | Charadriiformes | H5N8 |
| A/T Dk/NL-Zeewolde/16013976-001/2016(H5N8) | 2016-2017 | West Europe | Wild Anseriformes | H5N8 |
| A/T Dk/NL-Zeewolde/16013976-001-003/2016(H5N8) | 2016-2017 | West Europe | Wild Anseriformes | H5N8 |
| A/T Dk/NL-Zeewolde/16013976-004/2016(H5N8) | 2016-2017 | West Europe | Wild Anseriformes | H5N8 |
| A/T Dk/NL-Zeewolde/16013976-004-006/2016(H5N8) | 2016-2017 | West Europe | Wild Anseriformes | H5N8 |
| A/T Dk/NL-Zeewolde/16013976-005/2016(H5N8) | 2016-2017 | West Europe | Wild Anseriformes | H5N8 |
| A/T Dk/NL-Zeewolde/16013976-006/2016(H5N8) | 2016-2017 | West Europe | Wild Anseriformes | H5N8 |
| A/turkey/Germany-SH/R8595/2016(H5N8) | 2016-2017 | West Europe | Domestic Galliformes | H5N8 |
| A/Bk swan/NL-Den Oever/16013973-002/2016(H5N8) | 2016-2017 | West Europe | Wild Anseriformes | H5N8 |
| A/Dk/NL-Rotterdam/16014008-001-005/2016(H5N8) | 2016-2017 | West Europe | Domestic Anseriformes | H5N8 |
| A/Bl H gull/NL-Slootdorp/16014102-002/2016(H5N8) | 2016-2017 | West Europe | Charadriiformes | H5N8 |
| A/C Gull/NL-Slootdorp/16014102-003/2016(H5N8) | 2016-2017 | West Europe | Charadriiformes | H5N8 |
| A/chicken/Germany-MV/R8790/2016(H5N8) | 2016-2017 | West Europe | Domestic Galliformes | H5N8 |
| A/chicken/Germany-SH/R8758/2016(H5N8) | 2016-2017 | West Europe | Domestic Galliformes | H5N8 |
| A/duck/Hungary/55191/2016(H5N8) | 2016-2017 | East Europe | Domestic Anseriformes | H5N8 |
| A/Duck/Hungary/55764/2016(H5N8) | 2016-2017 | East Europe | Domestic Anseriformes | H5N8 |
| A/goose/Hungary/55128/2016(H5N8) | 2016-2017 | East Europe | Domestic Anseriformes | H5N8 |
| A/Gr bk bd gull/NL-Slootdorp/16014102-005/2016(H5N8) | 2016-2017 | West Europe | Charadriiformes | H5N8 |
| A/tufted duck/Germany-SN/R8795/2016(H5N8) | 2016-2017 | West Europe | Wild Anseriformes | H5N8 |
| A/mute swan/Croatia/78/2016(H5N8) | 2016-2017 | East Europe | Wild Anseriformes | H5N8 |
| A/Tufted Duck/Switzerland/V237/2016(H5N8) | 2016-2017 | West Europe | Wild Anseriformes | H5N8 |
| A/chicken/Iran/17RS654-01/2016(H5N8) | 2016-2017 | West Central Asia | Domestic Galliformes | H5N8 |
| A/chicken/Ukraine/1/2016(H5N8) | 2016-2017 | East Europe | Domestic Galliformes | H5N8 |
| A/chicken/Ukraine/3/2016(H5N8) | 2016-2017 | East Europe | Domestic Galliformes | H5N8 |
| A/Common Goldeneye/Sweden/SVA161117KU0322/SZ0002165/2016(H5N8) | 2016-2017 | West Europe | Wild Anseriformes | H5N8 |
| A/duck/Ukraine/4/2016(H5N8) | 2016-2017 | East Europe | Domestic Anseriformes | H5N8 |
| A/T Dk/NL-Rotterdam/16014155-001/2016(H5N8) | 2016-2017 | West Europe | Wild Anseriformes | H5N8 |
| A/T Dk/NL-Werkendam/16014159-001/2016(H5N5) | 2016-2017 | West Europe | Wild Anseriformes | H5N5 |
| A/T Dk/NL-Werkendam/16014159-002/2016(H5N8) | 2016-2017 | West Europe | Wild Anseriformes | H5N8 |
| A/T Dk/NL-Werkendam/16014159-003/2016(H5N8) | 2016-2017 | West Europe | Wild Anseriformes | H5N8 |
| A/T Dk/NL-Zuidoost Beemster/16014148-002/2016(H5N8) | 2016-2017 | West Europe | Wild Anseriformes | H5N8 |
| A/T Dk/NL-Zuidoost Beemster/16014148-009/2016(H5N8) | 2016-2017 | West Europe | Wild Anseriformes | H5N8 |
| A/Ch/NL-Den Oever/16014231-001/2016(H5N8) | 2016-2017 | West Europe | Domestic Galliformes | H5N8 |
| A/L-bl-ba-gull/NL-Sovon/16014324-014/2016(H5N8) | 2016-2017 | West Europe | Charadriiformes | H5N8 |
| A/Magpie/NL-Volendam/16014331-002/2016(H5N8) | 2016-2017 | West Europe | Other wild species | H5N8 |
| A/T Dk/NL-Almeerder Zand/16014341-003/2016(H5N8) | 2016-2017 | West Europe | Wild Anseriformes | H5N8 |
| A/Gull/NL-Marker Wadden/16014466-020/2016(H5N8) | 2016-2017 | West Europe | Charadriiformes | H5N8 |
| A/Gull1/NL-Marker Wadden/16014466-011/2016(H5N8) | 2016-2017 | West Europe | Charadriiformes | H5N8 |
| A/Gull10/NL-Marker Wadden/16014466-014/2016(H5N8) | 2016-2017 | West Europe | Charadriiformes | H5N8 |
| A/M Swan/NL-Roggebotsluis/16014462-019/2016(H5N8) | 2016-2017 | West Europe | Wild Anseriformes | H5N8 |
| A/T Dk/NL-Roggebotsluis/16014462-015/2016(H5N8) | 2016-2017 | West Europe | Wild Anseriformes | H5N8 |
| A/Go/NL-Roggebotsluis/16014462-010/2016(H5N8) | 2020-2022 | West Europe | Domestic Anseriformes | H5N1 |
| A/duck/Nigeria/17RS737-43/2016(H5N8) | 2016-2017 | Africa | Domestic Anseriformes | H5N8 |
| A/chicken/Kalmykia/2643/2016(H5N8) | 2016-2017 | East Europe | Domestic Galliformes | H5N8 |
| A/Chicken/Sweden/SVA161122KU0453/SZ0209316/2016(H5N8) | 2016-2017 | West Europe | Domestic Galliformes | H5N8 |
| A/Chicken/Sweden/SVA161122KU0453/SZ0209317/2016(H5N8) | 2016-2017 | West Europe | Domestic Galliformes | H5N8 |
| A/Chicken/Sweden/SVA161122KU0453/SZ0209318/2016(H5N8) | 2016-2017 | West Europe | Domestic Galliformes | H5N8 |
| A/Chicken/Sweden/SVA161122KU0453/SZ0209321/2016(H5N8) | 2016-2017 | West Europe | Domestic Galliformes | H5N8 |
| A/domestic duck/Germany-MV/R9764/2016(H5N8) | 2016-2017 | West Europe | Domestic Anseriformes | H5N8 |
| A/mallard/Hungary/57857/2016(H5N8) | 2016-2017 | East Europe | Wild Anseriformes | H5N8 |
| A/turkey/Germany-NI/R9807/2016(H5N8) | 2016-2017 | West Europe | Domestic Galliformes | H5N8 |
| A/chicken/Kalmykia/2661/2016(H5N8) | 2016-2017 | East Europe | Domestic Galliformes | H5N8 |
| A/domestic duck/Germany-MV/R9869/2016(H5N8) | 2016-2017 | West Europe | Domestic Anseriformes | H5N8 |
| A/Great Black-backed Gull/Netherlands/1/2016(H5N8) | 2016-2017 | West Europe | Charadriiformes | H5N8 |
| A/Mew Gull/Netherlands/1/2016(H5N8) | 2016-2017 | West Europe | Charadriiformes | H5N8 |
| A/Whooper Swan/Sanmenxia/01/2016(H5N8) | 2016-2017 | China | Wild Anseriformes | H5N8 |
| A/Great Black-backed Gull/Netherlands/2/2016(H5N8) | 2020-2022 | West Europe | Charadriiformes | H5N1 |
| A/Mulard duck/Hungary/59163/2016(H5N8) | 2016-2017 | East Europe | Wild Anseriformes | H5N8 |
| A/Mulard Duck/Hungary/59163/2016(H5N8) | 2016-2017 | East Europe | Wild Anseriformes | H5N8 |
| A/chicken/Hungary/59048/2016(H5N8) | 2016-2017 | East Europe | Domestic Galliformes | H5N8 |
| A/Common Pochard/Netherlands/1/2016(H5N8) | 2016-2017 | West Europe | Wild Anseriformes | H5N8 |
| A/Dk/NL-Biddinghuizen/16014829-011-015/2016(H5N8) | 2016-2017 | West Europe | Domestic Anseriformes | H5N8 |
| A/duck/Netherlands/16014829-001005/2016(H5N8) | 2016-2017 | West Europe | Domestic Anseriformes | H5N8 |
| A/Tufted Duck/Netherlands/1/2016(H5N8) | 2016-2017 | West Europe | Wild Anseriformes | H5N8 |
| A/Common-coot/Egypt/CA285/2016(H5N8) | 2016-2017 | Africa | Other wild species | H5N8 |
| A/decoy duck/France/161104e/2016(H5N8) | 2016-2017 | West Europe | Wild Anseriformes | H5N8 |
| A/Goose/Hungary/59712/2016(H5N8) | 2016-2017 | East Europe | Domestic Anseriformes | H5N8 |
| A/chicken/Germany-MV/R10048/2016(H5N8) | 2016-2017 | West Europe | Domestic Galliformes | H5N8 |
| A/Eur Wig/NL-De Waal (Texel | 2016-2017 | West Europe | Wild Anseriformes | H5N8 |
| A/Eur Wig/NL-De Waal (Texel | 2016-2017 | West Europe | Wild Anseriformes | H5N8 |
| A/Goose/Hungary/59763/2016(H5N8) | 2016-2017 | East Europe | Domestic Anseriformes | H5N8 |
| A/Grey seal/361-10/BalticPL/16(H5N8) | 2016-2017 | East Europe | Mammal | H5N8 |
| A/chicken/Iran/17RS654-03/2016(H5N8) | 2016-2017 | West Central Asia | Domestic Galliformes | H5N8 |
| A/duck/France/161108h/2016(H5N8) | 2016-2017 | West Europe | Domestic Anseriformes | H5N8 |
| A/mute swan/Croatia/85/2016(H5N8) | 2016-2017 | East Europe | Wild Anseriformes | H5N8 |
| A/White-tailed sea eagle/Germany/AR10297/2016(H5N8) | 2016-2017 | West Europe | Other wild species | H5N8 |
| A/Mulard duck/Hungary/60369/2016(H5N8) | 2016-2017 | East Europe | Wild Anseriformes | H5N8 |
| A/Buzzard/NL-Durgerdam/16015100-004/2016(H5N8) | 2016-2017 | West Europe | Other wild species | H5N8 |
| A/Dk/NL-Biddinghuizen/16015083-016-020/2016(H5N8) | 2016-2017 | West Europe | Domestic Anseriformes | H5N8 |
| A/Dk/NL-Biddinghuizen/16015145-021-025/2016(H5N8) | 2016-2017 | West Europe | Domestic Anseriformes | H5N8 |
| A/duck/France/161113/2016(H5N8) | 2016-2017 | West Europe | Domestic Anseriformes | H5N8 |
| A/duck/France/161116/2016(H5N8) | 2016-2017 | West Europe | Domestic Anseriformes | H5N8 |
| A/Eur Wig/NL-Ferwert/16015273-002/2016(H5N8) | 2016-2017 | West Europe | Wild Anseriformes | H5N8 |
| A/northern shoveler/Egypt/813C/2016(H5N8) | 2016-2017 | Africa | Wild Anseriformes | H5N8 |
| A/teal/Egypt /823C/2016(H5N8) | 2016-2017 | Africa | Wild Anseriformes | H5N8 |
| A/Teal/NL-Ferwert/16015273-013/2016(H5N8) | 2016-2017 | West Europe | Wild Anseriformes | H5N8 |
| A/domestic goose/Poland/33/2016(H5N8) | 2016-2017 | East Europe | Domestic Anseriformes | H5N8 |
| A/duck/France/161137/2016(H5N8) | 2016-2017 | West Europe | Domestic Anseriformes | H5N8 |
| A/duck/Hungary/60441/2016(H5N8) | 2016-2017 | East Europe | Domestic Anseriformes | H5N8 |
| A/chicken/Iran/17RS654-28/2016(H5N8) | 2016-2017 | West Central Asia | Domestic Galliformes | H5N8 |
| A/duck/France/161141/2016(H5N8) | 2016-2017 | West Europe | Domestic Anseriformes | H5N8 |
| A/duck/France/161142/2016(H5N8) | 2016-2017 | West Europe | Domestic Anseriformes | H5N8 |
| A/duck/France/161143/2016(H5N8) | 2016-2017 | West Europe | Domestic Anseriformes | H5N8 |
| A/Eurasian wigeon/Netherlands/1/2016(H5N8) | 2016-2017 | West Europe | Wild Anseriformes | H5N8 |
| A/Eurasian Wigeon/Netherlands/1/2016(H5N8) | 2016-2017 | West Europe | Wild Anseriformes | H5N8 |
| A/Eurasian Wigeon/Netherlands/9/2016(H5N8) | 2016-2017 | West Europe | Wild Anseriformes | H5N8 |
| A/Crow/NL-Oostwoud/16015372-004/2016(H5N8) | 2016-2017 | West Europe | Other wild species | H5N8 |
| A/duck/France/161147/2016(H5N8) | 2016-2017 | West Europe | Domestic Anseriformes | H5N8 |
| A/Eur Wig/NL-Groningen/16015376-003/2016(H5N8) | 2016-2017 | West Europe | Wild Anseriformes | H5N8 |
| A/Eurasian Wigeon/Netherlands/2/2016(H5N8) | 2016-2017 | West Europe | Wild Anseriformes | H5N8 |
| A/Eurasian Wigeon/Netherlands/21/2016(H5N8) | 2016-2017 | West Europe | Wild Anseriformes | H5N8 |
| A/Eurasian Wigeon/Netherlands/23/2016(H5N8) | 2016-2017 | West Europe | Wild Anseriformes | H5N8 |
| A/Mal/NL-Mastenbroek/16015378-002/2016(H5N8) | 2016-2017 | West Europe | Wild Anseriformes | H5N8 |
| A/Sea eagle/NL-Assen/16015398-002/2016(H5N8) | 2016-2017 | West Europe | Other wild species | H5N8 |
| A/duck/France/161173/2016(H5N8) | 2016-2017 | West Europe | Domestic Anseriformes | H5N8 |
| A/duck/France/161174/2016(H5N8) | 2016-2017 | West Europe | Domestic Anseriformes | H5N8 |
| A/Eur Wig/NL-Drieborg (Dollard | 2016-2017 | West Europe | Wild Anseriformes | H5N8 |
| A/flamingo/Iran/17RS654-18/2016(H5N8) | 2016-2017 | West Central Asia | Other wild species | H5N8 |
| A/Mal/NL-IJsselmuiden/16015448-002/2016(H5N8) | 2016-2017 | West Europe | Wild Anseriformes | H5N8 |
| A/little grebe/Iran/17RS654-10/2016(H5N8) | 2016-2017 | West Central Asia | Other wild species | H5N8 |
| A/Mulard duck/Hungary/62902/2016(H5N8) | 2016-2017 | East Europe | Wild Anseriformes | H5N8 |
| A/P falcon/NL-Vrouwenpolder (Zeeland | 2016-2017 | West Europe | Other wild species | H5N8 |
| A/chicken/Israel/881/2016(H5N8) | 2020-2022 | West Central Asia | Domestic Galliformes | H5N1 |
| A/Common Buzzard/Netherlands/1/2016(H5N8) | 2020-2022 | West Europe | Other wild species | H5N8 |
| A/duck/France/161182/2016(H5N8) | 2016-2017 | West Europe | Domestic Anseriformes | H5N8 |
| A/duck/France/161212/2016(H5N8) | 2016-2017 | West Europe | Domestic Anseriformes | H5N8 |
| A/Eur Wig/NL-Greonterp/16015653-001/2016(H5N8) | 2016-2017 | West Europe | Wild Anseriformes | H5N8 |
| A/Eurasian Wigeon/Netherlands/10/2016(H5N8) | 2016-2017 | West Europe | Wild Anseriformes | H5N8 |
| A/green-winged teal/Egypt/871/2016(H5N8) | 2016-2017 | Africa | Wild Anseriformes | H5N8 |
| A/green-winged teal/Egypt/877/2016(H5N8) | 2016-2017 | Africa | Wild Anseriformes | H5N8 |
| A/duck/France/161207/2016(H5N8) | 2016-2017 | West Europe | Domestic Anseriformes | H5N8 |
| A/Eur Wig/NL-Terschelling/16015692-010/2016(H5N8) | 2016-2017 | West Europe | Wild Anseriformes | H5N8 |
| A/Eurasian Wigeon/Netherlands/4/2016(H5N8) | 2016-2017 | West Europe | Wild Anseriformes | H5N8 |
| A/Eurasian Wigeon/Netherlands/6/2016(H5N8) | 2016-2017 | West Europe | Wild Anseriformes | H5N8 |
| A/Eurasian Wigeon/Netherlands/8/2016(H5N8) | 2016-2017 | West Europe | Wild Anseriformes | H5N8 |
| A/wild bird/Iran/17RS654-24/2016(H5N8) | 2016-2017 | West Central Asia | Other wild species | H5N8 |
| A/duck/France/161227/2016(H5N8) | 2016-2017 | West Europe | Domestic Anseriformes | H5N8 |
| A/duck/France/161228/2016(H5N8) | 2016-2017 | West Europe | Domestic Anseriformes | H5N8 |
| A/duck/France/161229/2016(H5N8) | 2016-2017 | West Europe | Domestic Anseriformes | H5N8 |
| A/Eur Wig/NL-Leeuwarden/16015699-002/2016(H5N8) | 2016-2017 | West Europe | Wild Anseriformes | H5N8 |
| A/Eur Wig/NL-Leidschendam/16015697-007/2016(H5N8) | 2016-2017 | West Europe | Wild Anseriformes | H5N8 |
| A/Eur Wig/NL-Zoeterwoude/16015702-010/2016(H5N8) | 2016-2017 | West Europe | Wild Anseriformes | H5N8 |
| A/duck/France/161224/2016(H5N8) | 2016-2017 | West Europe | Domestic Anseriformes | H5N8 |
| A/duck/France/161225/2016(H5N8) | 2016-2017 | West Europe | Domestic Anseriformes | H5N8 |
| A/duck/France/161239/2016(H5N8) | 2016-2017 | West Europe | Domestic Anseriformes | H5N8 |
| A/Eur Wig/NL-Enumatil-Groningen/16015704-001/2016(H5N8) | 2016-2017 | West Europe | Wild Anseriformes | H5N8 |
| A/Goose/Hungary/63743/2016(H5N8) | 2016-2017 | East Europe | Domestic Anseriformes | H5N8 |
| A/Ch/NL-Abbega/X16015736/2016(H5N8) | 2016-2017 | West Europe | Domestic Galliformes | H5N8 |
| A/duck/France/161230/2016(H5N8) | 2016-2017 | West Europe | Domestic Anseriformes | H5N8 |
| A/duck/France/161231/2016(H5N8) | 2016-2017 | West Europe | Domestic Anseriformes | H5N8 |
| A/duck/France/161233/2016(H5N8) | 2016-2017 | West Europe | Domestic Anseriformes | H5N8 |
| A/duck/France/161234/2016(H5N8) | 2016-2017 | West Europe | Domestic Anseriformes | H5N8 |
| A/duck/France/161240/2016(H5N8) | 2016-2017 | West Europe | Domestic Anseriformes | H5N8 |
| A/Eur Wig/NL-West Graftdijk/16015746-003/2016(H5N8) | 2016-2017 | West Europe | Wild Anseriformes | H5N8 |
| A/turkey/Poland/63/2016(H5N8) | 2016-2017 | East Europe | Domestic Galliformes | H5N8 |
| A/Anser cygnoides/Hubei/FW44/2016(H5N8) | 2016-2017 | China | Wild Anseriformes | H5N8 |
| A/chicken/Astrakhan/3131/2016(H5N8) | 2016-2017 | East Europe | Domestic Galliformes | H5N8 |
| A/duck/France/161242/2016(H5N8) | 2016-2017 | West Europe | Domestic Anseriformes | H5N8 |
| A/duck/France/161243/2016(H5N8) | 2016-2017 | West Europe | Domestic Anseriformes | H5N8 |
| A/Eur Wig/NL-Akkrum/16015817-003/2016(H5N8) | 2016-2017 | West Europe | Wild Anseriformes | H5N8 |
| A/Eur Wig/NL-Gouda/16015824-001/2016(H5N8) | 2016-2017 | West Europe | Wild Anseriformes | H5N8 |
| A/Eur Wig/NL-Reeuwijk/16015903-003/2016(H5N8) | 2016-2017 | West Europe | Wild Anseriformes | H5N8 |
| A/Eur Wig/NL-Vianen/16015917-006/2016(H5N8) | 2016-2017 | West Europe | Wild Anseriformes | H5N8 |
| A/Eur Wig/NL-Zwolle/16015820-002/2016(H5N8) | 2016-2017 | West Europe | Wild Anseriformes | H5N8 |
| A/Eurasian Wigeon/Netherlands/11/2016(H5N8) | 2016-2017 | West Europe | Wild Anseriformes | H5N8 |
| A/M Swan/NL-Groningen/16015826-001/2016(H5N5) | 2016-2017 | West Europe | Wild Anseriformes | H5N5 |
| A/swan/Germany-SN/R10645/2016(H5N5) | 2016-2017 | West Europe | Wild Anseriformes | H5N5 |
| A/turkey/Germany-NI/R10523/2016(H5N8) | 2016-2017 | West Europe | Domestic Galliformes | H5N8 |
| A/domestic goose/Poland/72/2016(H5N8) | 2016-2017 | East Europe | Domestic Anseriformes | H5N8 |
| A/Eur Wig/NL-Walterswald/16015923-003/2016(H5N8) | 2016-2017 | West Europe | Wild Anseriformes | H5N8 |
| A/Eurasian Wigeon/Netherlands/12/2016(H5N8) | 2016-2017 | West Europe | Wild Anseriformes | H5N8 |
| A/Eurasian Wigeon/Netherlands/13/2016(H5N8) | 2016-2017 | West Europe | Wild Anseriformes | H5N8 |
| A/Eurasian Wigeon/Netherlands/22/2016(H5N8) | 2016-2017 | West Europe | Wild Anseriformes | H5N8 |
| A/Goose/Hungary/64909/2016(H5N8) | 2016-2017 | East Europe | Domestic Anseriformes | H5N8 |
| A/Great Black-backed Gull/Netherlands/4/2016(H5N8) | 2016-2017 | West Europe | Charadriiformes | H5N8 |
| A/Grey Go/NL-Groot-Ammers/16015901-012/2016(H5N8) | 2016-2017 | West Europe | Wild Anseriformes | H5N8 |
| A/Ruddy Shelduck/AN/2-14-12/2016(H5N8) | 2016-2017 | East Europe | Wild Anseriformes | H5N8 |
| A/common teal/Korea/W547/2016(H5N8) | 2016-2017 | Korea | Wild Anseriformes | H5N8 |
| A/common teal/Korea/W548/2016(H5N8) | 2016-2017 | Korea | Wild Anseriformes | H5N8 |
| A/common teal/Korea/W549/2016(H5N8) | 2016-2017 | Korea | Wild Anseriformes | H5N8 |
| A/common teal/Korea/W550/2016(H5N8) | 2016-2017 | Korea | Wild Anseriformes | H5N8 |
| A/Dk/NL-Kamperveen/16016104-001-005/2016(H5N8) | 2016-2017 | West Europe | Domestic Anseriformes | H5N8 |
| A/duck/France/161263/2016(H5N8) | 2016-2017 | West Europe | Domestic Anseriformes | H5N8 |
| A/turkey/England/052131/2016(H5N8) | 2016-2017 | West Europe | Domestic Galliformes | H5N8 |
| A/White-fronted Goose/AN/1-15-12/2016(H5N8) | 2016-2017 | East Europe | Wild Anseriformes | H5N8 |
| A/duck/France/161256/2016(H5N8) | 2016-2017 | West Europe | Domestic Anseriformes | H5N8 |
| A/duck/France/161271/2016(H5N8) | 2016-2017 | West Europe | Domestic Anseriformes | H5N8 |
| A/duck/France/161298/2016(H5N8) | 2016-2017 | West Europe | Domestic Anseriformes | H5N8 |
| A/duck/France/161476/2016(H5N8) | 2016-2017 | West Europe | Domestic Anseriformes | H5N8 |
| A/turkey/Poland/78/2016(H5N8) | 2016-2017 | East Europe | Domestic Galliformes | H5N8 |
| A/turkey/Poland/78s1/2016(H5N8) | 2016-2017 | East Europe | Domestic Galliformes | H5N8 |
| A/Ch/NL-Hiaure/16016112-001-005/2016(H5N8) | 2016-2017 | West Europe | Domestic Galliformes | H5N8 |
| A/Ch/NL-Rhenen/16016141-006/2016(H5N8) | 2016-2017 | West Europe | Domestic Galliformes | H5N8 |
| A/chicken/Poland/79A/2016(H5N8) | 2016-2017 | East Europe | Domestic Galliformes | H5N8 |
| A/duck/France/161277/2016(H5N8) | 2016-2017 | West Europe | Domestic Anseriformes | H5N8 |
| A/duck/France/161275/2016(H5N8) | 2016-2017 | West Europe | Domestic Anseriformes | H5N8 |
| A/duck/Xinjiang/12.18 WLMQXL001-C/2016(H5N8) | 2016-2017 | China | Domestic Anseriformes | H5N8 |
| A/Eur Wig/NL-Wormer/16016143-002/2016(H5N8) | 2016-2017 | West Europe | Wild Anseriformes | H5N8 |
| A/Goose/Hungary/65817/2016(H5N8) | 2016-2017 | East Europe | Domestic Anseriformes | H5N8 |
| A/goose/Xinjiang/12.18 WLMQXL003-C/2016(H5N8) | 2016-2017 | China | Domestic Anseriformes | H5N8 |
| A/goose/Xinjiang/12.18 WLMQXL003-O/2016(H5N8) | 2016-2017 | China | Domestic Anseriformes | H5N8 |
| A/goose/Xinjiang/12.18 WLMQXL004-C/2016(H5N8) | 2016-2017 | China | Domestic Anseriformes | H5N8 |
| A/goose/Xinjiang/12.18 WLMQXL004-O/2016(H5N8) | 2016-2017 | China | Domestic Anseriformes | H5N8 |
| A/swan/China/ST/2016(H5N8) | 2016-2017 | China | Wild Anseriformes | H5N8 |
| A/Ch/NL-Boven Leeuwen/16016151-006-010/2016(H5N8) | 2016-2017 | West Europe | Domestic Galliformes | H5N8 |
| A/chicken/Iran/17RS654-08/2016(H5N8) | 2016-2017 | West Central Asia | Domestic Galliformes | H5N8 |
| A/chicken/Poland/85A/2016(H5N8) | 2016-2017 | East Europe | Domestic Galliformes | H5N8 |
| A/cormorant/Israel/1035/2016(H5N8) | 2016-2017 | West Central Asia | Other wild species | H5N8 |
| A/pochard duck/England/SA12 157809/2016(H5N8) | 2016-2017 | West Europe | Wild Anseriformes | H5N8 |
| A/turkey/Poland/81/2016(H5N8) | 2016-2017 | East Europe | Domestic Galliformes | H5N8 |
| A/turkey/Poland/83/2016(H5N8) | 2016-2017 | East Europe | Domestic Galliformes | H5N8 |
| A/Back-headed Gull/Netherlands/8/2016(H5N8) | 2016-2017 | West Europe | Charadriiformes | H5N8 |
| A/Back-headed Gull/Netherlands/9/2016(H5N8) | 2016-2017 | West Europe | Charadriiformes | H5N8 |
| A/Black-headed Gull/Netherlands/17/2016(H5N8) | 2016-2017 | West Europe | Charadriiformes | H5N8 |
| A/Caspian Gull/Netherlands/1/2016(H5N8) | 2016-2017 | West Europe | Charadriiformes | H5N8 |
| A/chicken/Israel/1048/2016(H5N8) | 2016-2017 | West Central Asia | Domestic Galliformes | H5N8 |
| A/Common Eider/Netherlands/2/2016(H5N8)) | 2016-2017 | West Europe | Wild Anseriformes | H5N8 |
| A/Cygnus atratus/Hubei/HF-1/2016(H5N8) | 2016-2017 | China | Wild Anseriformes | H5N8 |
| A/Eurasian Herring Gull/Netherlands/2/2016(H5N8) | 2016-2017 | West Europe | Charadriiformes | H5N8 |
| A/Lesser Black-backed Gull/Netherlands/1/2016(H5N8) | 2016-2017 | West Europe | Charadriiformes | H5N8 |
| A/turkey/Israel/1045/2016(H5N8) | 2016-2017 | West Central Asia | Domestic Galliformes | H5N8 |
| A/whooper swan/Shanxi/RC01/2016(H5N8) | 2016-2017 | China | Wild Anseriformes | H5N8 |
| A/Mallard/Netherlands/51/2016(H5N8) | 2020-2022 | West Europe | Wild Anseriformes | H5N1 |
| A/Dk/NL-Stolwijk/16016291-016-020/2016(H5N8) | 2016-2017 | West Europe | Domestic Anseriformes | H5N8 |
| A/duck/France/161383/2016(H5N8) | 2016-2017 | West Europe | Domestic Anseriformes | H5N8 |
| A/Great Crested Grebe/Netherlands/2/2016(H5N8) | 2016-2017 | West Europe | Other wild species | H5N8 |
| A/mute swan/Poland/108/2016(H5N8) | 2016-2017 | East Europe | Wild Anseriformes | H5N8 |
| A/domestic duck/Poland/88/2016(H5N8) | 2016-2017 | East Europe | Domestic Anseriformes | H5N8 |
| A/duck/France/161443/2016(H5N8) | 2016-2017 | West Europe | Domestic Anseriformes | H5N8 |
| A/duck/France/161445/2016(H5N8) | 2016-2017 | West Europe | Domestic Anseriformes | H5N8 |
| A/duck/France/161449/2016(H5N8) | 2016-2017 | West Europe | Domestic Anseriformes | H5N8 |
| A/duck/France/161450/2016(H5N8) | 2016-2017 | West Europe | Domestic Anseriformes | H5N8 |
| A/duck/France/161457/2016(H5N8) | 2016-2017 | West Europe | Domestic Anseriformes | H5N8 |
| A/turkey/Poland/89/2016(H5N8) | 2016-2017 | East Europe | Domestic Galliformes | H5N8 |
| A/duck/France/161444/2016(H5N8) | 2016-2017 | West Europe | Domestic Anseriformes | H5N8 |
| A/duck/France/161455/2016(H5N8) | 2016-2017 | West Europe | Domestic Anseriformes | H5N8 |
| A/Ch/NL-Zoeterwoude/16016484-021-025/2016(H5N8) | 2016-2017 | West Europe | Domestic Galliformes | H5N8 |
| A/chicken/Germany-NI/R11406/2016(H5N8) | 2016-2017 | West Europe | Domestic Galliformes | H5N8 |
| A/duck/France/161473/2016(H5N8) | 2016-2017 | West Europe | Domestic Anseriformes | H5N8 |
| A/turkey/Israel/1076/2016(H5N8) | 2016-2017 | West Central Asia | Domestic Galliformes | H5N8 |
| A/Whooper swan/Shanxi/6/2016(H5N8) | 2016-2017 | China | Wild Anseriformes | H5N8 |
| A/Whooper swan/Shanxi/7/2016(H5N8) | 2016-2017 | China | Wild Anseriformes | H5N8 |
| A/duck/France/161477/2016(H5N8) | 2016-2017 | West Europe | Domestic Anseriformes | H5N8 |
| A/duck/France/161478/2016(H5N8) | 2016-2017 | West Europe | Domestic Anseriformes | H5N8 |
| A/great egret/Israel/1084/2016(H5N8) | 2016-2017 | West Central Asia | Other wild species | H5N8 |
| A/great egret/Israel/1088/2016(H5N8) | 2016-2017 | West Central Asia | Other wild species | H5N8 |
| A/peregrine falcon/Israel/1086/2016(H5N8) | 2016-2017 | West Central Asia | Other wild species | H5N8 |
| A/turkey/Poland/93/2016(H5N8) | 2016-2017 | East Europe | Domestic Galliformes | H5N8 |
| A/duck/France/161456/2016(H5N8) | 2016-2017 | West Europe | Domestic Anseriformes | H5N8 |
| A/duck/France/161475/2016(H5N8) | 2016-2017 | West Europe | Domestic Anseriformes | H5N8 |
| A/duck/France/161500/2016(H5N8) | 2016-2017 | West Europe | Domestic Anseriformes | H5N8 |
| A/duck/France/161501/2016(H5N8) | 2016-2017 | West Europe | Domestic Anseriformes | H5N8 |
| A/turkey/Poland/100/2016(H5N8) | 2016-2017 | East Europe | Domestic Galliformes | H5N8 |
| A/turkey/Poland/100s3/2016(H5N8) | 2016-2017 | East Europe | Domestic Galliformes | H5N8 |
| A/chicken/Croatia/103/2016(H5N8) | 2016-2017 | East Europe | Domestic Galliformes | H5N8 |
| A/chicken/Poland/103/2016(H5N8) | 2016-2017 | East Europe | Domestic Galliformes | H5N8 |
| A/duck/France/161497/2016(H5N8) | 2016-2017 | West Europe | Domestic Anseriformes | H5N8 |
| A/duck/France/161577/2016(H5N8) | 2016-2017 | West Europe | Domestic Anseriformes | H5N8 |
| A/duck/France/161587/2016(H5N8) | 2016-2017 | West Europe | Domestic Anseriformes | H5N8 |
| A/greylag goose/Germany-NI/AR11353-L02142/2016(H5N5) | 2016-2017 | West Europe | Wild Anseriformes | H5N5 |
| A/mute swan/Croatia/102/2016(H5N5) | 2016-2017 | East Europe | Wild Anseriformes | H5N5 |
| A/turkey/Poland/107/2016(H5N8) | 2016-2017 | East Europe | Domestic Galliformes | H5N8 |
| A/chicken/Poland/114/2016(H5N8) | 2016-2017 | East Europe | Domestic Galliformes | H5N8 |
| A/Cygnus atratus/Hubei/2Z2-O/2016(H5N8) | 2016-2017 | China | Wild Anseriformes | H5N8 |
| A/domestic duck/112/2016(H5N8) | 2016-2017 | East Europe | Domestic Anseriformes | H5N8 |
| A/duck/France/161498/2016(H5N8) | 2016-2017 | West Europe | Domestic Anseriformes | H5N8 |
| A/duck/France/161587/2016(H5N8) | 2016-2017 | West Europe | Domestic Anseriformes | H5N8 |
| A/little grebe/Egypt/1056OP/2016(H5N8) | 2016-2017 | Africa | Other wild species | H5N8 |
| A/turkey/Poland/109s1/2016(H5N8) | 2016-2017 | East Europe | Domestic Galliformes | H5N8 |
| A/chicken/Poland/115/2016(H5N8) | 2016-2017 | East Europe | Domestic Galliformes | H5N8 |
| A/duck/France/161600/2016(H5N8) | 2016-2017 | West Europe | Domestic Anseriformes | H5N8 |
| A/mute swan/Croatia/104/2016(H5N8) | 2016-2017 | East Europe | Wild Anseriformes | H5N8 |
| A/White-tailed sea eagle/Germany/AR3093/2017(H5N8) | 2016-2017 | West Europe | Other wild species | H5N8 |
| A/wigeon/Italy/16VIR9616-3/2016(H5N5) | 2016-2017 | West Europe | Wild Anseriformes | H5N5 |
| A/chicken/Poland/116/2016(H5N8) | 2016-2017 | East Europe | Domestic Galliformes | H5N8 |
| A/chicken/Poland/117/2016(H5N8) | 2016-2017 | East Europe | Domestic Galliformes | H5N8 |
| A/Cygnus olor/England/WULH/2016(H5N8) | 2016-2017 | West Europe | Wild Anseriformes | H5N8 |
| A/duck/France/161601/2016(H5N8) | 2016-2017 | West Europe | Domestic Anseriformes | H5N8 |
| A/duck/France/170013/2016(H5N8) | 2016-2017 | West Europe | Domestic Anseriformes | H5N8 |
| A/duck/France/170016/2016(H5N8) | 2016-2017 | West Europe | Domestic Anseriformes | H5N8 |
| A/duck/France/170017/2016(H5N8) | 2016-2017 | West Europe | Domestic Anseriformes | H5N8 |
| A/chicken/Wales/000023/2016(H5N8) | 2016-2017 | West Europe | Domestic Galliformes | H5N8 |
| A/Cygnus olor/England/AS00918/2016(H5N8) | 2016-2017 | West Europe | Wild Anseriformes | H5N8 |
| A/Cygnus olor/England/WVJX/2016(H5N8) | 2016-2017 | West Europe | Wild Anseriformes | H5N8 |
| A/Cygnus olor/England/WVUK/2016(H5N8) | 2016-2017 | West Europe | Wild Anseriformes | H5N8 |
| A/Cygnus olor/England/WVZP/2016(H5N8) | 2016-2017 | West Europe | Wild Anseriformes | H5N8 |
| A/duck/France/170030/2016(H5N8) | 2016-2017 | West Europe | Domestic Anseriformes | H5N8 |
| A/duck/France/170033/2016(H5N8) | 2016-2017 | West Europe | Domestic Anseriformes | H5N8 |
| A/Bulbul/Riyadh/AI4/2017(H5N8) | 2016-2017 | West Central Asia | Other wild species | H5N8 |
| A/Chicken/Al-Ahsaa/AI7/2017(H5N8) | 2016-2017 | Africa | Domestic Galliformes | H5N8 |
| A/Chicken/Al-Ahsaa/AI8/2017(H5N8) | 2016-2017 | Africa | Domestic Galliformes | H5N8 |
| A/chicken/Cairo/1794FAO-S/2017(H5N8) | 2016-2017 | Africa | Domestic Galliformes | H5N8 |
| A/chicken/Cameroon/17RS1661-1/2017(H5N8) | 2016-2017 | Africa | Domestic Galliformes | H5N8 |
| A/chicken/Egypt/Buheira-12/2017(H5N8) | 2016-2017 | Africa | Domestic Galliformes | H5N8 |
| A/Chicken/Egypt/F1366A/2017(H5N8) | 2016-2017 | Africa | Domestic Galliformes | H5N8 |
| A/chicken/Egypt/Gharbiya-15/2017(H5N8) | 2016-2017 | Africa | Domestic Galliformes | H5N8 |
| A/chicken/Egypt/Kafr-Elshiekh-18/2017(H5N8) | 2016-2017 | Africa | Domestic Galliformes | H5N8 |
| A/Chicken/Riyadh/A15/2018(H5N8) | 2016-2017 | West Central Asia | Domestic Galliformes | H5N8 |
| A/Chicken/Riyadh/AI10/2017(H5N8) | 2016-2017 | West Central Asia | Domestic Galliformes | H5N8 |
| A/Chicken/Riyadh/AI6/2017(H5N8) | 2016-2017 | West Central Asia | Domestic Galliformes | H5N8 |
| A/chicken/Uganda/17RS115-15/2017(H5N8) | 2016-2017 | Africa | Domestic Galliformes | H5N8 |
| A/Cygnus olor/England/UnringedBirdB/2017(H5N8) | 2016-2017 | West Europe | Wild Anseriformes | H5N8 |
| A/Cygnus olor/England/YBOV/2017(H5N8) | 2016-2017 | West Europe | Wild Anseriformes | H5N8 |
| A/Duck/Al-Shaqiya/172AS/2017(H5N8) | 2016-2017 | Africa | Domestic Anseriformes | H5N8 |
| A/Duck/Al-Sharqiya/1733FM/2017(H5N8) | 2016-2017 | Africa | Domestic Anseriformes | H5N8 |
| A/duck/Cameroon/17RS1661-3/2017(H5N8) | 2016-2017 | Africa | Domestic Anseriformes | H5N8 |
| A/duck/Egypt/Buheira-21/2017(H5N8) | 2016-2017 | Africa | Domestic Anseriformes | H5N8 |
| A/Duck/Ismalia/171Fao-SI/2017(H5N8) | 2016-2017 | Africa | Domestic Anseriformes | H5N8 |
| A/Duck/Riyadh/AI2/2017(H5N8) | 2016-2017 | West Central Asia | Domestic Anseriformes | H5N8 |
| A/duck/Uganda/17RS115-9/2017(H5N8) | 2016-2017 | Africa | Domestic Anseriformes | H5N8 |
| A/Falcon/Riyadh/AI5/2017(H5N8) | 2016-2017 | West Central Asia | Other wild species | H5N8 |
| A/Grey-Headed Gull/Uganda/200144/2017(H5N8) | 2016-2017 | Africa | Charadriiformes | H5N8 |
| A/Holland pigeon/Riyadh/AI3/2017(H5N8) | 2016-2017 | West Central Asia | Other wild species | H5N8 |
| A/Indian peafowl/Cameroon/17RS1661-6/2017(H5N8) | 2016-2017 | Africa | Other wild species | H5N8 |
| A/Ornamental bird/Al-Qasim/AI9/2017(H5N8) | 2016-2017 | Africa | Other wild species | H5N8 |
| A/pigeon/Cameroon/17RS1661-4/2017(H5N8) | 2016-2017 | Africa | Domestic Galliformes | H5N8 |
| A/Turkey/Riyadh/AI1/2017(H5N8) | 2016-2017 | West Central Asia | Domestic Galliformes | H5N8 |
| A/turkey/Rostov-on-Don/11/2017(H5N8) | 2018-2019 | East Europe | Domestic Galliformes | H5N8 |
| A/chicken/Czech Republic/55-17 1/2017 (H5N8) | 2016-2017 | East Europe | Domestic Galliformes | H5N8 |
| A/chicken/Poland/002/2017(H5N8) | 2016-2017 | East Europe | Domestic Galliformes | H5N8 |
| A/duck/France/170018/2017(H5N8) | 2016-2017 | West Europe | Domestic Anseriformes | H5N8 |
| A/duck/France/170031/2017(H5N8) | 2016-2017 | West Europe | Domestic Anseriformes | H5N8 |
| A/duck/France/170036/2017(H5N8) | 2016-2017 | West Europe | Domestic Anseriformes | H5N8 |
| A/duck/France/170038/2017(H5N8) | 2016-2017 | West Europe | Domestic Anseriformes | H5N8 |
| A/duck/France/170098/2017(H5N8) | 2016-2017 | West Europe | Domestic Anseriformes | H5N8 |
| A/goose/Czech Republic/197-17/2017 (H5N8) | 2016-2017 | East Europe | Domestic Anseriformes | H5N8 |
| A/Harris Hawk/Hungary/120/2017(H5N8) | 2016-2017 | East Europe | Other wild species | H5N8 |
| A/mute swan/Czech Republic/54-17 1/2017 (H5N8) | 2016-2017 | East Europe | Wild Anseriformes | H5N8 |
| A/mute swan/Czech Republic/54-17 2/2017 (H5N8) | 2016-2017 | East Europe | Wild Anseriformes | H5N8 |
| A/Mute swan/Hungary/119/2017(H5N8) | 2016-2017 | East Europe | Wild Anseriformes | H5N8 |
| A/peafowl/Cameroon/17RS1661-6/2017(H5N8) | 2016-2017 | Africa | Other wild species | H5N8 |
| A/turkey/Poland/004/2017(H5N8) | 2016-2017 | East Europe | Domestic Galliformes | H5N8 |
| A/turkey/Poland/005/2017(H5N8) | 2016-2017 | East Europe | Domestic Galliformes | H5N8 |
| A/chicken/Egypt/Q13804A/2017(H5N8) | 2016-2017 | Africa | Domestic Galliformes | H5N8 |
| A/duck/France/170034/2017(H5N8) | 2016-2017 | West Europe | Domestic Anseriformes | H5N8 |
| A/mute swan/Croatia/9/2017(H5N8) | 2016-2017 | East Europe | Wild Anseriformes | H5N8 |
| A/shelduck/Italy/17VIR1572-24/2017(H5N8) | 2016-2017 | West Europe | Wild Anseriformes | H5N8 |
| A/turkey/Czech Republic/38-17 1/2017 (H5N8) | 2016-2017 | East Europe | Domestic Galliformes | H5N8 |
| A/turkey/Czech Republic/38-17 5/2017 (H5N8) | 2016-2017 | East Europe | Domestic Galliformes | H5N8 |
| A/wigeon/Italy/17VIR57-3/2017(H5N8) | 2016-2017 | West Europe | Wild Anseriformes | H5N8 |
| A/chicken/Czech Republic/1689-17/2017 (H5N8) | 2016-2017 | East Europe | Domestic Galliformes | H5N8 |
| A/chicken/France/170063/2017(H5N8) | 2016-2017 | West Europe | Domestic Galliformes | H5N8 |
| A/common teal/Korea/W555/2017(H5N8) | 2016-2017 | Korea | Wild Anseriformes | H5N8 |
| A/duck/France/170032/2017(H5N8) | 2016-2017 | West Europe | Domestic Anseriformes | H5N8 |
| A/duck/France/170064/2017(H5N8) | 2016-2017 | West Europe | Domestic Anseriformes | H5N8 |
| A/duck/France/170067/2017(H5N8) | 2016-2017 | West Europe | Domestic Anseriformes | H5N8 |
| A/eurasian wigeon/Germany-NI/AR249-L02143/2017(H5N8) | 2016-2017 | West Europe | Wild Anseriformes | H5N8 |
| A/goose/Czech Republic/136-17 1/2017 (H5N8) | 2016-2017 | East Europe | Domestic Anseriformes | H5N8 |
| A/grey-headed gull/Uganda/MUWRP-538/2017(H5N8) | 2016-2017 | Africa | Charadriiformes | H5N8 |
| A/Greylag goose/Hungary/320/2017(H5N8) | 2016-2017 | East Europe | Wild Anseriformes | H5N8 |
| A/mallard/Czech Republic/136-17 2/2017 (H5N8) | 2016-2017 | East Europe | Wild Anseriformes | H5N8 |
| A/chicken/Iran/17RS654-15/2017(H5N8) | 2016-2017 | West Central Asia | Domestic Galliformes | H5N8 |
| A/chicken/Poland/16/2017(H5N8) | 2016-2017 | East Europe | Domestic Galliformes | H5N8 |
| A/turkey/Poland/15/2017(H5N8) | 2016-2017 | East Europe | Domestic Galliformes | H5N8 |
| A/turkey/Poland/15s4/2017(H5N8) | 2016-2017 | East Europe | Domestic Galliformes | H5N8 |
| A/chicken/Czech Republic/206-17 2/2017(H5N8) | 2016-2017 | East Europe | Domestic Galliformes | H5N8 |
| A/chicken/Czech Republic/206-17 2/2017(H5N8) | 2016-2017 | East Europe | Domestic Galliformes | H5N8 |
| A/chicken/Voronezh/18/2017(H5N8) | 2016-2017 | East Europe | Domestic Galliformes | H5N8 |
| A/chicken/Voronezh/19/2017(H5N8) | 2016-2017 | East Europe | Domestic Galliformes | H5N8 |
| A/chicken/Voronezh/20/2017(H5N8) | 2016-2017 | East Europe | Domestic Galliformes | H5N8 |
| A/duck/France/170118/2017(H5N8) | 2016-2017 | West Europe | Domestic Anseriformes | H5N8 |
| A/goose/Krasnodar/3144/2017(H5N8) | 2016-2017 | East Europe | Domestic Anseriformes | H5N8 |
| A/GuineaFowl/Hungary/596/2017(H5N8) | 2016-2017 | East Europe | Other wild species | H5N8 |
| A/long-eared owl/Voronezh/15/2017(H5N8) | 2016-2017 | East Europe | Other wild species | H5N8 |
| A/long-eared owl/Voronezh/16/2017(H5N8) | 2016-2017 | East Europe | Other wild species | H5N8 |
| A/mute swan/Krasnodar/25/2017(H5N8) | 2016-2017 | East Europe | Wild Anseriformes | H5N8 |
| A/Ural owl/Voronezh/14/2017(H5N8) | 2016-2017 | East Europe | Other wild species | H5N8 |
| A/chicken/France/170176/2017(H5N8) | 2016-2017 | West Europe | Domestic Galliformes | H5N8 |
| A/chicken/Niger/17RS167-1/2017(H5N1) | 2016-2017 | Africa | Domestic Galliformes | H5N1 |
| A/chicken/Niger/17RS167-3/2017(H5N1) | 2016-2017 | Africa | Domestic Galliformes | H5N1 |
| A/Mallard/Netherlands/2/2017(H5N8) | 2016-2017 | West Europe | Wild Anseriformes | H5N8 |
| A/Mallard/Netherlands/1/2017(H5N8) | 2020-2022 | West Europe | Wild Anseriformes | H5N1 |
| A/duck/France/170117/2017(H5N8) | 2016-2017 | West Europe | Domestic Anseriformes | H5N8 |
| A/duck/France/170177/2017(H5N8) | 2016-2017 | West Europe | Domestic Anseriformes | H5N8 |
| A/duck/France/170178/2017(H5N8) | 2016-2017 | West Europe | Domestic Anseriformes | H5N8 |
| A/turkey/Germany-BB/R234ff/2017(H5N8) | 2016-2017 | West Europe | Domestic Galliformes | H5N8 |
| A/White fronted goose/Hungary/801/2017(H5N8) | 2016-2017 | East Europe | Wild Anseriformes | H5N8 |
| A/duck/France/170245/2017(H5N8) | 2016-2017 | West Europe | Domestic Anseriformes | H5N8 |
| A/duck/France/170249/2017(H5N8) | 2016-2017 | West Europe | Domestic Anseriformes | H5N8 |
| A/duck/France/170250/2017(H5N8) | 2016-2017 | West Europe | Domestic Anseriformes | H5N8 |
| A/gadwall/Italy/17VIR133-2/2017(H5N5) | 2016-2017 | West Europe | Wild Anseriformes | H5N5 |
| A/mute swan/Croatia/15/2017(H5N8) | 2016-2017 | East Europe | Wild Anseriformes | H5N8 |
| A/swan/France/170166/2017(H5N8) | 2016-2017 | West Europe | Wild Anseriformes | H5N8 |
| A/chicken/Poland/34/2017(H5N8) | 2016-2017 | East Europe | Domestic Galliformes | H5N8 |
| A/duck/Bangladesh/19D770/2017(H5N6) | 2016-2017 | West Central Asia | Domestic Anseriformes | H5N6 |
| A/duck/France/170280/2017(H5N8) | 2016-2017 | West Europe | Domestic Anseriformes | H5N8 |
| A/duck/France/170284/2017(H5N8) | 2016-2017 | West Europe | Domestic Anseriformes | H5N8 |
| A/Duck/Hungary/984/2017(H5N8) | 2016-2017 | East Europe | Domestic Anseriformes | H5N8 |
| A/goose/Bangladesh/19D764/2017(H5N6) | 2016-2017 | West Central Asia | Domestic Anseriformes | H5N6 |
| A/Goose/Hungary/1030/2017(H5N8) | 2016-2017 | East Europe | Domestic Anseriformes | H5N8 |
| A/Goose/Hungary/982/2017(H5N8) | 2016-2017 | East Europe | Domestic Anseriformes | H5N8 |
| A/Mallard/Netherlands/3/2017(H5N8) | 2016-2017 | West Europe | Wild Anseriformes | H5N8 |
| A/mute swan/Czech Republic/499-17/2017 (H5N8) | 2016-2017 | East Europe | Wild Anseriformes | H5N8 |
| A/turkey/France/170278/2017(H5N8) | 2016-2017 | West Europe | Domestic Galliformes | H5N8 |
| A/Black swan/Hubei/2/2017(H5N8) | 2016-2017 | China | Wild Anseriformes | H5N8 |
| A/Black swan/Hubei/3/2017(H5N8) | 2016-2017 | China | Wild Anseriformes | H5N8 |
| A/chicken/Czech Republic/508-17 1/2017 (H5N8) | 2016-2017 | East Europe | Domestic Galliformes | H5N8 |
| A/Duck/Egypt/FAO-SI2/2017(H5N8) | 2016-2017 | Africa | Domestic Anseriformes | H5N8 |
| A/duck/Egypt/SS19/2017(H5N8) | 2016-2017 | Africa | Domestic Anseriformes | H5N8 |
| A/duck/Egypt/SS19/2017 (H5N8) | 2016-2017 | Africa | Domestic Anseriformes | H5N8 |
| A/duck/France/170180/2017(H5N8) | 2016-2017 | West Europe | Domestic Anseriformes | H5N8 |
| A/duck/France/170325/2017(H5N8) | 2016-2017 | West Europe | Domestic Anseriformes | H5N8 |
| A/mallard/Czech Republic/508-17 4/2017 (H5N8) | 2016-2017 | East Europe | Wild Anseriformes | H5N8 |
| A/mute swan/Poland/12/2017(H5N8) | 2016-2017 | East Europe | Wild Anseriformes | H5N8 |
| A/chicken/France/170340/2017(H5N8) | 2016-2017 | West Europe | Domestic Galliformes | H5N8 |
| A/Cygnus olor/England/AS00778/2017(H5N8) | 2016-2017 | West Europe | Wild Anseriformes | H5N8 |
| A/duck/France/170308/2017(H5N8) | 2016-2017 | West Europe | Domestic Anseriformes | H5N8 |
| A/duck/France/170309/2017(H5N8) | 2016-2017 | West Europe | Domestic Anseriformes | H5N8 |
| A/duck/France/170330/2017(H5N8) | 2016-2017 | West Europe | Domestic Anseriformes | H5N8 |
| A/duck/France/170331/2017(H5N8) | 2016-2017 | West Europe | Domestic Anseriformes | H5N8 |
| A/duck/France/170339/2017(H5N8) | 2016-2017 | West Europe | Domestic Anseriformes | H5N8 |
| A/duck/France/170341/2017(H5N8) | 2016-2017 | West Europe | Domestic Anseriformes | H5N8 |
| A/duck/France/170349/2017(H5N8) | 2016-2017 | West Europe | Domestic Anseriformes | H5N8 |
| A/mallard/Poland/17/2017(H5N8) | 2016-2017 | East Europe | Wild Anseriformes | H5N8 |
| A/mute swan/Czech Republic/572-17 3/2017 (H5N8) | 2016-2017 | East Europe | Wild Anseriformes | H5N8 |
| A/mute swan/Czech Republic/581-17/2017 (H5N8) | 2016-2017 | East Europe | Wild Anseriformes | H5N8 |
| A/breeder duck/Croatia/21/2017(H5N8) | 2016-2017 | East Europe | Domestic Anseriformes | H5N8 |
| A/duck/France/170369/2017(H5N8) | 2016-2017 | West Europe | Domestic Anseriformes | H5N8 |
| A/duck/France/170370/2017(H5N8) | 2016-2017 | West Europe | Domestic Anseriformes | H5N8 |
| A/Mallard/Hungary/1574a/2017(H5N8) | 2016-2017 | East Europe | Wild Anseriformes | H5N8 |
| A/Mallard/Hungary/1574b/2017(H5N8) | 2016-2017 | East Europe | Wild Anseriformes | H5N8 |
| A/mute swan/Czech Republic/722-17 1/2017 (H5N8) | 2016-2017 | East Europe | Wild Anseriformes | H5N8 |
| A/chicken/Czech Republic/585-17 1/2017 (H5N8) | 2016-2017 | East Europe | Domestic Galliformes | H5N8 |
| A/Cygnus olor/England/AS00868/2017(H5N8) | 2016-2017 | West Europe | Wild Anseriformes | H5N8 |
| A/mallard/Czech Republic/722-17 2/2017 (H5N8) | 2016-2017 | East Europe | Wild Anseriformes | H5N8 |
| A/mute swan/Czech Republic/653-17/2017 (H5N8) | 2016-2017 | East Europe | Wild Anseriformes | H5N8 |
| A/turkey/England/003778/2017(H5N8) | 2016-2017 | West Europe | Domestic Galliformes | H5N8 |
| A/chicken/France/170407/2017(H5N8) | 2016-2017 | West Europe | Domestic Galliformes | H5N8 |
| A/chicken/France/170433/2017(H5N8) | 2016-2017 | West Europe | Domestic Galliformes | H5N8 |
| A/domestic duck/Poland/47/2017(H5N8) | 2016-2017 | East Europe | Domestic Anseriformes | H5N8 |
| A/duck/France/170338/2017(H5N8) | 2016-2017 | West Europe | Domestic Anseriformes | H5N8 |
| A/duck/France/170368/2017(H5N8) | 2016-2017 | West Europe | Domestic Anseriformes | H5N8 |
| A/Duck/Hungary/1588/2017(H5N8) | 2016-2017 | East Europe | Domestic Anseriformes | H5N8 |
| A/Indian Runner Duck/Czech Republic/749-17/2017 (H5N8) | 2016-2017 | East Europe | Wild Anseriformes | H5N8 |
| A/chicken/France/170408/2017(H5N8) | 2016-2017 | West Europe | Domestic Galliformes | H5N8 |
| A/chicken/France/170436/2017(H5N8) | 2016-2017 | West Europe | Domestic Galliformes | H5N8 |
| A/Chicken/Hungary/1751/2017(H5N8) | 2016-2017 | East Europe | Domestic Galliformes | H5N8 |
| A/duck/France/170437/2017(H5N8) | 2016-2017 | West Europe | Domestic Anseriformes | H5N8 |
| A/goose/Czech Republic/821-17 2/2017 (H5N8) | 2016-2017 | East Europe | Domestic Anseriformes | H5N8 |
| A/guineafowl/France/170409/2017(H5N8) | 2016-2017 | West Europe | Other wild species | H5N8 |
| A/swan/Poland/23/2017(H5N8) | 2016-2017 | East Europe | Wild Anseriformes | H5N8 |
| A/turkey/Germany-BB/R377ff/2017(H5N8) | 2016-2017 | West Europe | Domestic Galliformes | H5N8 |
| A/duck/France/170432/2017(H5N8) | 2016-2017 | West Europe | Domestic Anseriformes | H5N8 |
| A/duck/France/170518/2017(H5N8) | 2016-2017 | West Europe | Domestic Anseriformes | H5N8 |
| A/greylag goose/Croatia/33/2017(H5N8) | 2016-2017 | East Europe | Wild Anseriformes | H5N8 |
| A/Greylag goose/Hungary/1941/2017(H5N8) | 2016-2017 | East Europe | Wild Anseriformes | H5N8 |
| A/mute swan/Czech Republic/1058-17/2017 (H5N8) | 2016-2017 | East Europe | Wild Anseriformes | H5N8 |
| A/mute swan/Czech Republic/879-17/2017 (H5N8) | 2016-2017 | East Europe | Wild Anseriformes | H5N8 |
| A/Mute swan/Hungary/1955/2017(H5N8) | 2016-2017 | East Europe | Wild Anseriformes | H5N8 |
| A/Turkey/Hungary/2030/2017(H5N8) | 2016-2017 | East Europe | Domestic Galliformes | H5N8 |
| A/turkey/Poland/54/2017(H5N8) | 2016-2017 | East Europe | Domestic Galliformes | H5N8 |
| A/mute swan/Czech Republic/1060-17/2017 (H5N8) | 2016-2017 | East Europe | Wild Anseriformes | H5N8 |
| A/mute swan/Czech Republic/964-17/2017 (H5N8) | 2016-2017 | East Europe | Wild Anseriformes | H5N8 |
| A/mute swan/Czech Republic/967-17/2017 (H5N8) | 2016-2017 | East Europe | Wild Anseriformes | H5N8 |
| A/Mute swan/Hungary/2193/2017(H5N8) | 2016-2017 | East Europe | Wild Anseriformes | H5N8 |
| A/mute swan/Poland/30/2017(H5N8) | 2016-2017 | East Europe | Wild Anseriformes | H5N8 |
| A/swan/Italy/17VIR537-2/2017(H5N8) | 2016-2017 | West Europe | Wild Anseriformes | H5N8 |
| A/swan/Poland/32/2017(H5N8) | 2016-2017 | East Europe | Wild Anseriformes | H5N8 |
| A/chicken/Czech Republic/988-17/2017 (H5N8) | 2016-2017 | East Europe | Domestic Galliformes | H5N8 |
| A/chicken/Iran/17RS654-29/2017(H5N8) | 2016-2017 | West Central Asia | Domestic Galliformes | H5N8 |
| A/duck/France/170683/2017(H5N8) | 2016-2017 | West Europe | Domestic Anseriformes | H5N8 |
| A/mallard/Poland/33/2017(H5N8) | 2016-2017 | East Europe | Wild Anseriformes | H5N8 |
| A/mute swan/Croatia/42/2017(H5N5) | 2016-2017 | East Europe | Wild Anseriformes | H5N5 |
| A/mute swan/Czech Republic/1330-17 1/2017 (H5N8) | 2016-2017 | East Europe | Wild Anseriformes | H5N8 |
| A/mute swan/Czech Republic/987-17 2/2017 (H5N8) | 2016-2017 | East Europe | Wild Anseriformes | H5N8 |
| A/turkey/Italy/17VIR538-1/2017(H5N8) | 2016-2017 | West Europe | Domestic Galliformes | H5N8 |
| A/chicken/Republic of Macedonia/AR1167-L02131/2017(H5N8) | 2016-2017 | East Europe | Domestic Galliformes | H5N8 |
| A/mallard/Czech Republic/1226-17/2017 (H5N8) | 2016-2017 | East Europe | Wild Anseriformes | H5N8 |
| A/grey heron/Germany-SN/R572/2017(H5N5) | 2016-2017 | West Europe | Other wild species | H5N5 |
| A/mute swan/Croatia/30/2017(H5N8) | 2016-2017 | East Europe | Wild Anseriformes | H5N8 |
| A/mute swan/Czech Republic/1155-17/2017 (H5N8) | 2016-2017 | East Europe | Wild Anseriformes | H5N8 |
| A/mute swan/Czech Republic/1156-17/2017 (H5N8) | 2016-2017 | East Europe | Wild Anseriformes | H5N8 |
| A/mute swan/Czech Republic/1170-17 2/2017 (H5N8) | 2016-2017 | East Europe | Wild Anseriformes | H5N8 |
| A/mute swan/Czech Republic/1171-17/2017 (H5N8) | 2016-2017 | East Europe | Wild Anseriformes | H5N8 |
| A/mute swan/Czech Republic/1227-17/2017 (H5N8) | 2016-2017 | East Europe | Wild Anseriformes | H5N8 |
| A/Mute swan/Hungary/2508/2017(H5N8) | 2016-2017 | East Europe | Wild Anseriformes | H5N8 |
| A/turkey/Germany-SH/R425/2017(H5N5) | 2016-2017 | West Europe | Domestic Galliformes | H5N5 |
| A/chicken/Iran/17RS654-26/2017(H5N8) | 2016-2017 | West Central Asia | Domestic Galliformes | H5N8 |
| A/chicken/Iran/17RS654-34/2017(H5N8) | 2016-2017 | West Central Asia | Domestic Galliformes | H5N8 |
| A/chicken/Niger/17RS167-19/2017(H5N8) | 2016-2017 | Africa | Domestic Galliformes | H5N8 |
| A/duck/France/170612/2017(H5N8) | 2016-2017 | West Europe | Domestic Anseriformes | H5N8 |
| A/duck/France/170685/2017(H5N8) | 2016-2017 | West Europe | Domestic Anseriformes | H5N8 |
| A/goose/Niger/17RS167-22/2017(H5N8) | 2016-2017 | Africa | Domestic Anseriformes | H5N8 |
| A/mute swan/Czech Republic/1331-17 1/2017 (H5N8) | 2016-2017 | East Europe | Wild Anseriformes | H5N8 |
| A/turkey/Italy/17VIR576-11/2017(H5N8) | 2016-2017 | West Europe | Domestic Galliformes | H5N8 |
| A/chicken/Czech Republic/1208-17 1/2017 (H5N8) | 2016-2017 | East Europe | Domestic Galliformes | H5N8 |
| A/chicken/France/170686/2017(H5N8) | 2016-2017 | West Europe | Domestic Galliformes | H5N8 |
| A/chicken/France/170774/2017(H5N8) | 2016-2017 | West Europe | Domestic Galliformes | H5N8 |
| A/Chicken/Hungary/2496/2017(H5N8) | 2016-2017 | East Europe | Domestic Galliformes | H5N8 |
| A/duck/France/170738/2017(H5N8) | 2016-2017 | West Europe | Domestic Anseriformes | H5N8 |
| A/duck/France/170775/2017(H5N8) | 2016-2017 | West Europe | Domestic Anseriformes | H5N8 |
| A/Harris hawk/Hungary/2762a/2017(H5N8) | 2016-2017 | East Europe | Other wild species | H5N8 |
| A/Harris hawk/Hungary/2762b/2017(H5N8) | 2016-2017 | East Europe | Other wild species | H5N8 |
| A/mallard duck/Korea/WA137/2017(H5N8) | 2016-2017 | Korea | Wild Anseriformes | H5N8 |
| A/mute swan/Czech Republic/1296-17 1/2017 (H5N8) | 2016-2017 | East Europe | Wild Anseriformes | H5N8 |
| A/Mute swan/Hungary/2825/2017(H5N8) | 2016-2017 | East Europe | Wild Anseriformes | H5N8 |
| A/chicken/Italy/17VIR653-12/2017(H5N8) | 2016-2017 | West Europe | Domestic Galliformes | H5N8 |
| A/duck/France/170733/2017(H5N8) | 2016-2017 | West Europe | Domestic Anseriformes | H5N8 |
| A/duck/France/170734/2017(H5N8) | 2016-2017 | West Europe | Domestic Anseriformes | H5N8 |
| A/duck/France/170735/2017(H5N8) | 2016-2017 | West Europe | Domestic Anseriformes | H5N8 |
| A/duck/France/170806/2017(H5N8) | 2016-2017 | West Europe | Domestic Anseriformes | H5N8 |
| A/greylag goose/Germany-NI/AR703-L02138/2017(H5N8) | 2016-2017 | West Europe | Wild Anseriformes | H5N8 |
| A/mallard/Czech Republic/1219-17 1/2017 (H5N8) | 2016-2017 | East Europe | Wild Anseriformes | H5N8 |
| A/chicken/Egypt/F13660A/2017(H5N8) | 2016-2017 | Africa | Domestic Galliformes | H5N8 |
| A/chicken/Egypt/F13664A/2017(H5N8) | 2016-2017 | Africa | Domestic Galliformes | H5N8 |
| A/domestic duck/Germany-BB/R681ff/2017(H5N8) | 2016-2017 | West Europe | Domestic Anseriformes | H5N8 |
| A/duck/Egypt/F13663C/2017(H5N8) | 2016-2017 | Africa | Domestic Anseriformes | H5N8 |
| A/duck/Egypt/F13666A/2017(H5N8) | 2016-2017 | Africa | Domestic Anseriformes | H5N8 |
| A/duck/Egypt/F13667A/2017(H5N8) | 2016-2017 | Africa | Domestic Anseriformes | H5N8 |
| A/Mute swan/Hungary/3137/2017(H5N8) | 2016-2017 | East Europe | Wild Anseriformes | H5N8 |
| A/Mute swan/Hungary/3139/2017(H5N8) | 2016-2017 | East Europe | Wild Anseriformes | H5N8 |
| A/mute swan/Poland/54/2017(H5N8) | 2016-2017 | East Europe | Wild Anseriformes | H5N8 |
| A/swan/Poland/49/2017(H5N8) | 2016-2017 | East Europe | Wild Anseriformes | H5N8 |
| A/avian/France/170977/2017(H5N8) | 2016-2017 | West Europe | Domestic Galliformes | H5N8 |
| A/chicken/France/170773/2017(H5N8) | 2016-2017 | West Europe | Domestic Galliformes | H5N8 |
| A/domestic goose/Poland/69/2017(H5N8) | 2016-2017 | East Europe | Domestic Anseriformes | H5N8 |
| A/duck/France/170772/2017(H5N8) | 2016-2017 | West Europe | Domestic Anseriformes | H5N8 |
| A/grey heron/W779/2017(H5N8) | 2016-2017 | Korea | Other wild species | H5N8 |
| A/mute swan/Czech Republic/1337-17/2017 (H5N8) | 2016-2017 | East Europe | Wild Anseriformes | H5N8 |
| A/Mute swan/Hungary/3513/2017(H5N8) | 2016-2017 | East Europe | Wild Anseriformes | H5N8 |
| A/swan/Poland/56/2017(H5N8) | 2016-2017 | East Europe | Wild Anseriformes | H5N8 |
| A/turkey/Poland/72/2017(H5N8) | 2016-2017 | East Europe | Domestic Galliformes | H5N8 |
| A/wild duck/Poland/57/2017(H5N8) | 2016-2017 | East Europe | Wild Anseriformes | H5N8 |
| A/chicken/Republic of Macedonia/466/2017(H5N8) | 2016-2017 | East Europe | Domestic Galliformes | H5N8 |
| A/mallard/Czech Republic/1577-17/2017 (H5N8) | 2016-2017 | East Europe | Wild Anseriformes | H5N8 |
| A/mute swan/Czech Republic/1461-17/2017 (H5N8) | 2016-2017 | East Europe | Wild Anseriformes | H5N8 |
| A/Mute swan/Hungary/3542/2017(H5N8) | 2016-2017 | East Europe | Wild Anseriformes | H5N8 |
| A/bronze turkey/Czech Republic/1414-17/2017 (H5N8) | 2016-2017 | East Europe | Domestic Galliformes | H5N8 |
| A/chicken/Czech Republic/1465-17/2017 (H5N8) | 2016-2017 | East Europe | Domestic Galliformes | H5N8 |
| A/chicken/Poland/77/2017(H5N8) | 2016-2017 | East Europe | Domestic Galliformes | H5N8 |
| A/cormorant/Germany-SH/R896/2017(H5N5) | 2016-2017 | West Europe | Other wild species | H5N5 |
| A/duck/Czech Republic/1467-17/2017 (H5N8) | 2016-2017 | East Europe | Domestic Anseriformes | H5N8 |
| A/duck/France/170820/2017(H5N8) | 2016-2017 | West Europe | Domestic Anseriformes | H5N8 |
| A/duck/France/170822/2017(H5N8) | 2016-2017 | West Europe | Domestic Anseriformes | H5N8 |
| A/mallard/Czech Republic/1672-17/2017 (H5N8) | 2016-2017 | East Europe | Wild Anseriformes | H5N8 |
| A/chicken/Czech Republic/1344-17/2017 (H5N8) | 2016-2017 | East Europe | Domestic Galliformes | H5N8 |
| A/duck/France/170974/2017(H5N8) | 2016-2017 | West Europe | Domestic Anseriformes | H5N8 |
| A/duck/France/170975/2017(H5N8) | 2016-2017 | West Europe | Domestic Anseriformes | H5N8 |
| A/mute swan/Czech Republic/1339-17/2017 (H5N8) | 2016-2017 | East Europe | Wild Anseriformes | H5N8 |
| A/mute swan/Czech Republic/1576-17 C/2017 (H5N8) | 2016-2017 | East Europe | Wild Anseriformes | H5N8 |
| A/mute swan/Poland/64/2017(H5N5) | 2016-2017 | East Europe | Wild Anseriformes | H5N5 |
| A/mute swan/Poland/68/2017(H5N8) | 2016-2017 | East Europe | Wild Anseriformes | H5N8 |
| A/chicken/Belgium/807/2017(H5N8) | 2016-2017 | West Europe | Domestic Galliformes | H5N8 |
| A/domestic duck/Poland/90/2017(H5N8) | 2016-2017 | East Europe | Domestic Anseriformes | H5N8 |
| A/Duck/Ismalia/175Fao-SI/2017(H5N8) | 2016-2017 | Africa | Domestic Anseriformes | H5N8 |
| A/Duck/Ismalia/176FAO-SI/2017(H5N8) | 2016-2017 | Africa | Domestic Anseriformes | H5N8 |
| A/goose/France/170976/2017(H5N8) | 2016-2017 | West Europe | Domestic Anseriformes | H5N8 |
| A/Guinea fowl/Belgium/810/2017(H5N8) | 2016-2017 | West Europe | Other wild species | H5N8 |
| A/mute swan/Poland/72/2017(H5N8) | 2016-2017 | East Europe | Wild Anseriformes | H5N8 |
| A/turkey/Italy/17VIR973-2/2017(H5N8) | 2016-2017 | West Europe | Domestic Galliformes | H5N8 |
| A/duck/France/171131/2017(H5N8) | 2016-2017 | West Europe | Domestic Anseriformes | H5N8 |
| A/grey heron/Czech Republic/1680-17 2/2017 (H5N8) | 2016-2017 | East Europe | Other wild species | H5N8 |
| A/greylag goose/Germany-NI/AR1395-L02144/2017(H5N8) | 2016-2017 | West Europe | Wild Anseriformes | H5N8 |
| A/mute swan/Czech Republic/1519-17/2017 (H5N8) | 2016-2017 | East Europe | Wild Anseriformes | H5N8 |
| A/mute swan/Czech Republic/1640-17/2017 (H5N8) | 2016-2017 | East Europe | Wild Anseriformes | H5N8 |
| A/mute swan/Czech Republic/1691-17/2017 (H5N8) | 2016-2017 | East Europe | Wild Anseriformes | H5N8 |
| A/mute swan/Poland/76/2017(H5N8) | 2016-2017 | East Europe | Wild Anseriformes | H5N8 |
| A/swan/Poland/81/2017(H5N5) | 2016-2017 | East Europe | Wild Anseriformes | H5N5 |
| A/wild duck/Poland/78/2017(H5N8) | 2016-2017 | East Europe | Wild Anseriformes | H5N8 |
| A/mute swan/Croatia/61/2017(H5N8) | 2020-2022 | East Europe | Wild Anseriformes | H5N1 |
| A/chicken/Czech Republic/1675-17 2/2017 (H5N8) | 2016-2017 | East Europe | Domestic Galliformes | H5N8 |
| A/chicken/Poland/101/2017(H5N8) | 2016-2017 | East Europe | Domestic Galliformes | H5N8 |
| A/domestic duck/Poland/106/2017(H5N8) | 2016-2017 | East Europe | Domestic Anseriformes | H5N8 |
| A/duck/France/171133/2017(H5N8) | 2016-2017 | West Europe | Domestic Anseriformes | H5N8 |
| A/duck/France/171134/2017(H5N8) | 2016-2017 | West Europe | Domestic Anseriformes | H5N8 |
| A/duck/France/171202/2017(H5N8) | 2016-2017 | West Europe | Domestic Anseriformes | H5N8 |
| A/Indian Runner Duck/Czech Republic/1683-17 1/2017 (H5N8) | 2016-2017 | East Europe | Wild Anseriformes | H5N8 |
| A/turkey/Poland/94/2017(H5N8) | 2016-2017 | East Europe | Domestic Galliformes | H5N8 |
| A/chicken/Czech Republic/1687-17 2/2017 (H5N8) | 2016-2017 | East Europe | Domestic Galliformes | H5N8 |
| A/chicken/Czech Republic/1688-17 1/2017 (H5N8) | 2016-2017 | East Europe | Domestic Galliformes | H5N8 |
| A/grey heron/Germany-TH/R1125/2017(H5N8) | 2016-2017 | West Europe | Other wild species | H5N8 |
| A/mute swan/Germany-TH/R1126/2017(H5N8) | 2016-2017 | West Europe | Wild Anseriformes | H5N8 |
| A/swan/Poland/88/2017(H5N8) | 2016-2017 | East Europe | Wild Anseriformes | H5N8 |
| A/bronze turkey/Czech Republic/1755-17 1/2017 (H5N8) | 2016-2017 | East Europe | Domestic Galliformes | H5N8 |
| A/chicken/Bangladesh/1896-17-HA/2017(H5N6) | 2016-2017 | West Central Asia | Domestic Galliformes | H5N6 |
| A/duck/France/171253/2017(H5N8) | 2016-2017 | West Europe | Domestic Anseriformes | H5N8 |
| A/mallard/Czech Republic/1690-17 2/2017 (H5N8) | 2016-2017 | East Europe | Wild Anseriformes | H5N8 |
| A/mute swan/Czech Republic/1813-17/2017 (H5N8) | 2016-2017 | East Europe | Wild Anseriformes | H5N8 |
| A/turkey/Israel/184/2017(H5N8) | 2016-2017 | West Central Asia | Domestic Galliformes | H5N8 |
| A/chicken/Greece/39 2017/2017(H5N6) | 2016-2017 | East Europe | Domestic Galliformes | H5N6 |
| A/chicken/Greece/39 2017a/2017(H5N6) | 2016-2017 | East Europe | Domestic Galliformes | H5N6 |
| A/chicken/Greece/39 2017b/2017(H5N6) | 2016-2017 | East Europe | Domestic Galliformes | H5N6 |
| A/chicken/Korea/Gimje2/2017(H5N8) | 2016-2017 | Korea | Domestic Galliformes | H5N8 |
| A/domestic goose/Poland/124/2017(H5N8) | 2016-2017 | East Europe | Domestic Anseriformes | H5N8 |
| A/duck/France/171379/2017(H5N8) | 2016-2017 | West Europe | Domestic Anseriformes | H5N8 |
| A/goose/Denmark/1365-1p1c/2017(H5N8) | 2016-2017 | West Europe | Domestic Anseriformes | H5N8 |
| A/goose/France/171278/2017(H5N8) | 2016-2017 | West Europe | Domestic Anseriformes | H5N8 |
| A/mute swan/Czech Republic/1848-17 1/2017 (H5N8) | 2016-2017 | East Europe | Wild Anseriformes | H5N8 |
| A/mute swan/Czech Republic/1848-17 2/2017 (H5N8) | 2016-2017 | East Europe | Wild Anseriformes | H5N8 |
| A/turkey/Czech Republic/1767-17 2/2017 (H5N8) | 2016-2017 | East Europe | Domestic Galliformes | H5N8 |
| A/white stork/Germany-TH/R1149/2017(H5N8) | 2016-2017 | West Europe | Other wild species | H5N8 |
| A/common buzzard/Germany-SN/R1117/2017(H5N5) | 2020-2022 | West Europe | Other wild species | H5N1 |
| A/chicken/Czech Republic/1863-17 1/2017 (H5N8) | 2016-2017 | East Europe | Domestic Galliformes | H5N8 |
| A/chicken/Czech Republic/1896-17 1/2017 (H5N8) | 2016-2017 | East Europe | Domestic Galliformes | H5N8 |
| A/duck/France/171270/2017(H5N8) | 2016-2017 | West Europe | Domestic Anseriformes | H5N8 |
| A/duck/France/171275/2017(H5N8) | 2016-2017 | West Europe | Domestic Anseriformes | H5N8 |
| A/duck/France/171378/2017(H5N8) | 2016-2017 | West Europe | Domestic Anseriformes | H5N8 |
| A/duck/France/171408/2017(H5N8) | 2016-2017 | West Europe | Domestic Anseriformes | H5N8 |
| A/mute swan/Czech Republic/2008-17 1/2017 (H5N8) | 2016-2017 | East Europe | Wild Anseriformes | H5N8 |
| A/Peregrine falcon/Hungary/4882/2017(H5N8) | 2016-2017 | East Europe | Other wild species | H5N8 |
| A/chicken/Czech Republic/1953-17/2017 (H5N8) | 2016-2017 | East Europe | Domestic Galliformes | H5N8 |
| A/chicken/Korea/H903/2017(H5N8) | 2016-2017 | Korea | Domestic Galliformes | H5N8 |
| A/duck/France/171410/2017(H5N8) | 2016-2017 | West Europe | Domestic Anseriformes | H5N8 |
| A/goose/Czech Republic/1954-17/2017 (H5N8) | 2016-2017 | East Europe | Domestic Anseriformes | H5N8 |
| A/peacock/Belgium/1017/2017(H5N8) | 2016-2017 | West Europe | Other wild species | H5N8 |
| A/Rook/Hungary/4975/2017(H5N8) | 2016-2017 | East Europe | Other wild species | H5N8 |
| A/tawny owl/Germany-SN/R1186/2017(H5N8) | 2016-2017 | West Europe | Other wild species | H5N8 |
| A/chicken/Croatia/70/2017(H5N8) | 2020-2022 | East Europe | Domestic Galliformes | H5N1 |
| A/goose/Czech Republic/1998-17 1/2017 (H5N8) | 2016-2017 | East Europe | Domestic Anseriformes | H5N8 |
| A/mute swan/Czech Republic/2031-17/2017 (H5N5) | 2016-2017 | East Europe | Wild Anseriformes | H5N5 |
| A/Mute swan/Hungary/5316/2017(H5N8) | 2016-2017 | East Europe | Wild Anseriformes | H5N8 |
| A/swan/Poland/99/2017(H5N8) | 2016-2017 | East Europe | Wild Anseriformes | H5N8 |
| A/duck/France/171466/2017(H5N8) | 2016-2017 | West Europe | Domestic Anseriformes | H5N8 |
| A/duck/France/171467/2017(H5N8) | 2016-2017 | West Europe | Domestic Anseriformes | H5N8 |
| A/duck/France/171468/2017(H5N8) | 2016-2017 | West Europe | Domestic Anseriformes | H5N8 |
| A/chicken/Czech Republic/2216-17 1/2017 (H5N8) | 2016-2017 | East Europe | Domestic Galliformes | H5N8 |
| A/chicken/France/171471/2017(H5N8) | 2016-2017 | West Europe | Domestic Galliformes | H5N8 |
| A/duck/France/171455/2017(H5N8) | 2016-2017 | West Europe | Domestic Anseriformes | H5N8 |
| A/duck/France/171457/2017(H5N8) | 2016-2017 | West Europe | Domestic Anseriformes | H5N8 |
| A/duck/France/171469/2017(H5N8) | 2016-2017 | West Europe | Domestic Anseriformes | H5N8 |
| A/duck/France/171472/2017(H5N8) | 2016-2017 | West Europe | Domestic Anseriformes | H5N8 |
| A/duck/France/171486/2017(H5N8) | 2016-2017 | West Europe | Domestic Anseriformes | H5N8 |
| A/duck/France/171514/2017(H5N8) | 2016-2017 | West Europe | Domestic Anseriformes | H5N8 |
| A/quail/Czech Republic/2063-17 1/2017 (H5N8) | 2016-2017 | East Europe | Domestic Galliformes | H5N8 |
| A/black swan/Germany-BW/R1364/2017(H5N8) | 2016-2017 | West Europe | Wild Anseriformes | H5N8 |
| A/chicken/France/171534/2017(H5N8) | 2016-2017 | West Europe | Domestic Galliformes | H5N8 |
| A/duck/France/171461/2017(H5N8) | 2016-2017 | West Europe | Domestic Anseriformes | H5N8 |
| A/duck/France/171462/2017(H5N8) | 2016-2017 | West Europe | Domestic Anseriformes | H5N8 |
| A/duck/France/171463/2017(H5N8) | 2016-2017 | West Europe | Domestic Anseriformes | H5N8 |
| A/duck/France/171535/2017(H5N8) | 2016-2017 | West Europe | Domestic Anseriformes | H5N8 |
| A/mute swan/Poland/109/2017(H5N8) | 2016-2017 | East Europe | Wild Anseriformes | H5N8 |
| A/swan/Poland/107/2017(H5N8) | 2016-2017 | East Europe | Wild Anseriformes | H5N8 |
| A/chicken/France/171525/2017(H5N8) | 2016-2017 | West Europe | Domestic Galliformes | H5N8 |
| A/duck/France/171533/2017(H5N8) | 2016-2017 | West Europe | Domestic Anseriformes | H5N8 |
| A/duck/France/171542/2017(H5N8) | 2016-2017 | West Europe | Domestic Anseriformes | H5N8 |
| A/Mallard/Hungary/5821/2017(H5N8) | 2016-2017 | East Europe | Wild Anseriformes | H5N8 |
| A/mute swan/Kaliningrad/132/2017(H5N8) | 2016-2017 | East Europe | Wild Anseriformes | H5N8 |
| A/chicken/ Egypt/N13732A/2017(H5N8) | 2016-2017 | Africa | Domestic Galliformes | H5N8 |
| A/chicken/Egypt/N13717E/2017(H5N8) | 2016-2017 | Africa | Domestic Galliformes | H5N8 |
| A/chicken/Egypt/N13720A/2017(H5N8) | 2016-2017 | Africa | Domestic Galliformes | H5N8 |
| A/chicken/Egypt/N13726/2017(H5N8) | 2016-2017 | Africa | Domestic Galliformes | H5N8 |
| A/chicken/France/171537/2017(H5N8) | 2016-2017 | West Europe | Domestic Galliformes | H5N8 |
| A/duck/France/171536/2017(H5N8) | 2016-2017 | West Europe | Domestic Anseriformes | H5N8 |
| A/duck/France/171539/2017(H5N8) | 2016-2017 | West Europe | Domestic Anseriformes | H5N8 |
| A/duck/France/171541/2017(H5N8) | 2016-2017 | West Europe | Domestic Anseriformes | H5N8 |
| A/duck/France/171543/2017(H5N8) | 2016-2017 | West Europe | Domestic Anseriformes | H5N8 |
| A/duck/France/171546/2017(H5N8) | 2016-2017 | West Europe | Domestic Anseriformes | H5N8 |
| A/duck/France/171550/2017(H5N8) | 2016-2017 | West Europe | Domestic Anseriformes | H5N8 |
| A/duck/France/171641/2017(H5N8) | 2016-2017 | West Europe | Domestic Anseriformes | H5N8 |
| A/egret/Germany-SH/R1459/2017(H5N5) | 2016-2017 | West Europe | Other wild species | H5N5 |
| A/mute swan/Germany-NI/AR1529-L02145/2017(H5N8) | 2016-2017 | West Europe | Wild Anseriformes | H5N8 |
| A/Mute swan/Hungary/5879/2017(H5N5) | 2016-2017 | East Europe | Wild Anseriformes | H5N5 |
| A/spot-billed pelican/Czech Republic/2270-17/2017 (H5N5) | 2016-2017 | East Europe | Wild Anseriformes | H5N5 |
| A/turkey/Italy/17VIR1338-3/2017(H5N8) | 2016-2017 | West Europe | Domestic Galliformes | H5N8 |
| A/avian/France/171599/2017(H5N8) | 2016-2017 | West Europe | Domestic Galliformes | H5N8 |
| A/Cormorant/Hungary/6102/2017(H5N8) | 2016-2017 | East Europe | Other wild species | H5N8 |
| A/duck/Egypt/N13733B/2017(H5N8) | 2016-2017 | Africa | Domestic Anseriformes | H5N8 |
| A/duck/Egypt/N13734B/2017(H5N8) | 2016-2017 | Africa | Domestic Anseriformes | H5N8 |
| A/duck/Egypt/N13735A/2017(H5N8) | 2016-2017 | Africa | Domestic Anseriformes | H5N8 |
| A/duck/Egypt/N13736B/2017(H5N8) | 2016-2017 | Africa | Domestic Anseriformes | H5N8 |
| A/duck/Egypt/N13736E/2017(H5N8) | 2016-2017 | Africa | Domestic Anseriformes | H5N8 |
| A/duck/France/171607/2017(H5N8) | 2016-2017 | West Europe | Domestic Anseriformes | H5N8 |
| A/duck/France/171639/2017(H5N8) | 2016-2017 | West Europe | Domestic Anseriformes | H5N8 |
| A/duck/France/171650/2017(H5N8) | 2016-2017 | West Europe | Domestic Anseriformes | H5N8 |
| A/Mute swan/Hungary/6092/2017(H5N8) | 2016-2017 | East Europe | Wild Anseriformes | H5N8 |
| A/swan/France/170496/2017(H5N8) | 2016-2017 | West Europe | Wild Anseriformes | H5N8 |
| A/avian/France/171606/2017(H5N8) | 2016-2017 | West Europe | Domestic Galliformes | H5N8 |
| A/duck/France/171603/2017(H5N8) | 2016-2017 | West Europe | Domestic Anseriformes | H5N8 |
| A/Mute swan/Hungary/6276/2017(H5N8) | 2016-2017 | East Europe | Wild Anseriformes | H5N8 |
| A/Pheasant/Hungary/6553/2017(H5N8) | 2016-2017 | East Europe | Other wild species | H5N8 |
| A/turkey/Italy/17VIR1452-22/2017(H5N8) | 2016-2017 | West Europe | Domestic Galliformes | H5N8 |
| A/mallard/Czech Republic/2641-17/2017 (H5N8) | 2016-2017 | East Europe | Wild Anseriformes | H5N8 |
| A/chicken/Czech Republic/2514-17/2017 (H5N8) | 2016-2017 | East Europe | Domestic Galliformes | H5N8 |
| A/domestic goose/Poland/190/2017(H5N8) | 2016-2017 | East Europe | Domestic Anseriformes | H5N8 |
| A/duck/France/171779/2017(H5N8) | 2016-2017 | West Europe | Domestic Anseriformes | H5N8 |
| A/duck/France/171884/2017(H5N8) | 2016-2017 | West Europe | Domestic Anseriformes | H5N8 |
| A/mallard/Czech Republic/2678-17 1/2017 (H5N8) | 2016-2017 | East Europe | Wild Anseriformes | H5N8 |
| A/turkey/Poland/192/2017(H5N8) | 2016-2017 | East Europe | Domestic Galliformes | H5N8 |
| A/chicken/France/171915/2017(H5N8) | 2016-2017 | West Europe | Domestic Galliformes | H5N8 |
| A/chicken/Poland/199/2017(H5N8) | 2016-2017 | East Europe | Domestic Galliformes | H5N8 |
| A/Common buzzard/Hungary/7061/2017(H5N8) | 2016-2017 | East Europe | Other wild species | H5N5 |
| A/Cygnus olor/Belgium/1567/2017(H5N8) | 2016-2017 | West Europe | Wild Anseriformes | H5N8 |
| A/domestic goose/Poland/204/2017(H5N8) | 2016-2017 | East Europe | Domestic Anseriformes | H5N8 |
| A/duck/France/171860/2017(H5N8) | 2016-2017 | West Europe | Domestic Anseriformes | H5N8 |
| A/mute swan/Poland/137/2017(H5N8) | 2016-2017 | East Europe | Wild Anseriformes | H5N8 |
| A/turkey/Italy/17VIR1574-1/2017(H5N8) | 2016-2017 | West Europe | Domestic Galliformes | H5N8 |
| A/chicken/Czech Republic/2643-17 1/2017 (H5N8) | 2016-2017 | East Europe | Domestic Galliformes | H5N8 |
| A/chicken/Czech Republic/2644-17 1/2017 (H5N8) | 2016-2017 | East Europe | Domestic Galliformes | H5N8 |
| A/chicken/Czech Republic/2677-17 2/2017 (H5N8) | 2016-2017 | East Europe | Domestic Galliformes | H5N8 |
| A/chicken/Poland/208/2017(H5N8) | 2016-2017 | East Europe | Domestic Galliformes | H5N8 |
| A/duck/France/171874/2017(H5N8) | 2016-2017 | West Europe | Domestic Anseriformes | H5N8 |
| A/mallard/Czech Republic/2705-17/2017 (H5N8) | 2016-2017 | East Europe | Wild Anseriformes | H5N8 |
| A/duck/France/172012/2017(H5N8) | 2016-2017 | West Europe | Domestic Anseriformes | H5N8 |
| A/Bean goose/Hubei/CH-i119/2017(H5N8) | 2016-2017 | China | Wild Anseriformes | H5N8 |
| A/Bean goose/Hubei/CH-i122/2017 H5N8(H5N8) | 2016-2017 | China | Wild Anseriformes | H5N8 |
| A/chicken/Czech Republic/2764-17 1/2017 (H5N8) | 2016-2017 | East Europe | Domestic Galliformes | H5N8 |
| A/chicken/Czech Republic/2764-17 2/2017 (H5N8) | 2016-2017 | East Europe | Domestic Galliformes | H5N8 |
| A/duck/France/171929/2017(H5N8) | 2016-2017 | West Europe | Domestic Anseriformes | H5N8 |
| A/duck/France/171932/2017(H5N8) | 2016-2017 | West Europe | Domestic Anseriformes | H5N8 |
| A/Herring Gull/Hubei/CH-i149/2017 H5N8(H5N8) | 2016-2017 | China | Charadriiformes | H5N8 |
| A/Pheasant/Hungary/7685/2017(H5N8) | 2016-2017 | East Europe | Other wild species | H5N8 |
| A/chicken/Egypt/A13775D/2017(H5N8) | 2016-2017 | Africa | Domestic Galliformes | H5N8 |
| A/chicken/Egypt/A13776B/2017(H5N8) | 2016-2017 | Africa | Domestic Galliformes | H5N8 |
| A/chicken/Egypt/A13777B/2017(H5N8) | 2016-2017 | Africa | Domestic Galliformes | H5N8 |
| A/chicken/France/172002/2017(H5N8) | 2016-2017 | West Europe | Domestic Galliformes | H5N8 |
| A/chicken/France/172003/2017(H5N8) | 2016-2017 | West Europe | Domestic Galliformes | H5N8 |
| A/duck/France/172004/2017(H5N8) | 2016-2017 | West Europe | Domestic Anseriformes | H5N8 |
| A/goose/Egypt/A13779B/2017(H5N8) | 2016-2017 | Africa | Domestic Anseriformes | H5N8 |
| A/teal/Egypt/1198C/2017(H5N8) | 2016-2017 | Africa | Wild Anseriformes | H5N8 |
| A/teal/Egypt/1202C/2017(H5N8) | 2016-2017 | Africa | Wild Anseriformes | H5N8 |
| A/chicken/Egypt/H13791C/2017(H5N8) | 2016-2017 | Africa | Domestic Galliformes | H5N8 |
| A/chicken/Egypt/H13793E/2017(H5N8) | 2016-2017 | Africa | Domestic Galliformes | H5N8 |
| A/chicken/Egypt/H13794E/2017(H5N8) | 2016-2017 | Africa | Domestic Galliformes | H5N8 |
| A/chicken/Egypt/H13795A/2017(H5N8) | 2016-2017 | Africa | Domestic Galliformes | H5N8 |
| A/chicken/Egypt/H13796A/2017(H5N8) | 2016-2017 | Africa | Domestic Galliformes | H5N8 |
| A/chicken/Egypt/H13799B/2017(H5N8) | 2016-2017 | Africa | Domestic Galliformes | H5N8 |
| A/chicken/Egypt/H13800C/2017(H5N8) | 2016-2017 | Africa | Domestic Galliformes | H5N8 |
| A/duck/Egypt/H13797E/2017(H5N8) | 2016-2017 | Africa | Domestic Anseriformes | H5N8 |
| A/Anas platyrhynchos/Belgium/1899/2017(H5N8) | 2016-2017 | West Europe | Wild Anseriformes | H5N8 |
| A/chicken/Czech Republic/2821-17 2/2017 (H5N8) | 2016-2017 | East Europe | Domestic Galliformes | H5N8 |
| A/chicken/Czech Republic/2822-17 2/2017 (H5N8) | 2016-2017 | East Europe | Domestic Galliformes | H5N8 |
| A/chicken/Italy/17VIR1684-2/2017(H5N8) | 2016-2017 | West Europe | Domestic Galliformes | H5N8 |
| A/chicken/Italy/17VIR1751-3/2017(H5N8) | 2016-2017 | West Europe | Domestic Galliformes | H5N8 |
| A/duck/France/172038/2017(H5N8) | 2016-2017 | West Europe | Domestic Anseriformes | H5N8 |
| A/duck/France/172057/2017(H5N8) | 2016-2017 | West Europe | Domestic Anseriformes | H5N8 |
| A/duck/France/172144/2017(H5N8) | 2016-2017 | West Europe | Domestic Anseriformes | H5N8 |
| A/mallard/Czech Republic/2820-17 1/2017 (H5N8) | 2016-2017 | East Europe | Wild Anseriformes | H5N8 |
| A/mallard/Czech Republic/2820-17 2/2017 (H5N8) | 2016-2017 | East Europe | Wild Anseriformes | H5N8 |
| A/mallard/Czech Republic/2821-17 1/2017 (H5N8) | 2016-2017 | East Europe | Wild Anseriformes | H5N8 |
| A/mallard/Czech Republic/2822-17 1/2017 (H5N8) | 2016-2017 | East Europe | Wild Anseriformes | H5N8 |
| A/chicken/Czech Republic/3507-17/2017 (H5N8) | 2016-2017 | East Europe | Domestic Galliformes | H5N8 |
| A/chicken/France/172092/2017(H5N8) | 2016-2017 | West Europe | Domestic Galliformes | H5N8 |
| A/chicken/France/172093/2017(H5N8) | 2016-2017 | West Europe | Domestic Galliformes | H5N8 |
| A/Common tern/Hungary/8187/2017(H5N8) | 2016-2017 | East Europe | Other wild species | H5N8 |
| A/domestic duck/Poland/238/2017(H5N8) | 2016-2017 | East Europe | Domestic Anseriformes | H5N8 |
| A/chicken/Al Qaliobia/1755FAO-S/2017(H5N8) | 2016-2017 | Africa | Domestic Galliformes | H5N8 |
| A/Duck/Cairo/1895CA/2017(H5N8) | 2016-2017 | Africa | Domestic Anseriformes | H5N8 |
| A/duck/France/172094/2017(H5N8) | 2016-2017 | West Europe | Domestic Anseriformes | H5N8 |
| A/duck/France/172096/2017(H5N8) | 2016-2017 | West Europe | Domestic Anseriformes | H5N8 |
| A/duck/France/172182/2017(H5N8) | 2016-2017 | West Europe | Domestic Anseriformes | H5N8 |
| A/Duck/Giza/1754FAO-S/2017(H5N8) | 2016-2017 | Africa | Domestic Anseriformes | H5N8 |
| A/chicken/France/172137/2017(H5N8) | 2016-2017 | West Europe | Domestic Galliformes | H5N8 |
| A/chicken/Sergiyev Posad/38/2017(H5N8) | 2016-2017 | Russian Federation Siberia | Domestic Galliformes | H5N8 |
| A/chicken/Sergiyev Posad/39/2017(H5N8) | 2016-2017 | Russian Federation Siberia | Domestic Galliformes | H5N8 |
| A/duck/France/171928/2017(H5N8) | 2016-2017 | West Europe | Domestic Anseriformes | H5N8 |
| A/goose/Spain/IA17CR02699/2017(H5N8) | 2016-2017 | West Europe | Domestic Anseriformes | H5N8 |
| A/chicken/Poland/263/2017(H5N8) | 2016-2017 | East Europe | Domestic Galliformes | H5N8 |
| A/duck/France/172289/2017(H5N8) | 2016-2017 | West Europe | Domestic Anseriformes | H5N8 |
| A/chicken/Shchyolkovo/47/2017(H5N8) | 2016-2017 | Russian Federation Siberia | Domestic Galliformes | H5N8 |
| A/Bean goose/Hubei/CH-i320/2017 H5N8(H5N8) | 2016-2017 | China | Wild Anseriformes | H5N8 |
| A/domestic goose/Poland/270/2017(H5N8) | 2016-2017 | East Europe | Domestic Anseriformes | H5N8 |
| A/chicken/Croatia/104/2017(H5N5) | 2016-2017 | East Europe | Domestic Galliformes | H5N5 |
| A/chicken/Egypt/F13829A/2017(H5N8) | 2016-2017 | Africa | Domestic Galliformes | H5N8 |
| A/chicken/Egypt/F13830A/2017(H5N8) | 2016-2017 | Africa | Domestic Galliformes | H5N8 |
| A/chicken/France/172287/2017(H5N8) | 2016-2017 | West Europe | Domestic Galliformes | H5N8 |
| A/Duck/Egypt/AR518/2017(H5N8) | 2016-2017 | Africa | Domestic Anseriformes | H5N8 |
| A/turkey/Poland/285/2017(H5N8) | 2016-2017 | East Europe | Domestic Galliformes | H5N8 |
| A/Chicken/Egypt/AR520/2017(H5N8) | 2016-2017 | Africa | Domestic Galliformes | H5N8 |
| A/duck/France/172331/2017(H5N8) | 2016-2017 | West Europe | Domestic Anseriformes | H5N8 |
| A/Duck/Egypt/CAL28/2017(H5N8) | 2016-2017 | Africa | Domestic Anseriformes | H5N8 |
| A/Buzzard/Netherlands/18004242-002/2018(H5N6) | 2016-2017 | West Europe | Other wild species | H5N6 |
| A/chicken/Egypt/M13844B/2017(H5N8) | 2016-2017 | Africa | Domestic Galliformes | H5N8 |
| A/chicken/Egypt/Q13845B/2017(H5N8) | 2016-2017 | Africa | Domestic Galliformes | H5N8 |
| A/chicken/Italy/17VIR2294-5/2017(H5N8) | 2016-2017 | West Europe | Domestic Galliformes | H5N8 |
| A/duck/France/172362/2017(H5N8) | 2016-2017 | West Europe | Domestic Anseriformes | H5N8 |
| A/swan/France/171265/2017(H5N8) | 2016-2017 | West Europe | Wild Anseriformes | H5N8 |
| A/chicken/France/172383/2017(H5N8) | 2016-2017 | West Europe | Domestic Galliformes | H5N8 |
| A/Cygnus olor/Belgium/2967/2017(H5N8) | 2016-2017 | West Europe | Wild Anseriformes | H5N8 |
| A/duck/France/172390/2017(H5N8) | 2016-2017 | West Europe | Domestic Anseriformes | H5N8 |
| A/duck/France/172394/2017(H5N8) | 2016-2017 | West Europe | Domestic Anseriformes | H5N8 |
| A/swan/France/171267/2017(H5N8) | 2016-2017 | West Europe | Wild Anseriformes | H5N8 |
| A/duck/France/172384/2017(H5N8) | 2016-2017 | West Europe | Domestic Anseriformes | H5N8 |
| A/Buteo buteo/Belgium/3022/2017(H5N8) | 2016-2017 | West Europe | Other wild species | H5N8 |
| A/swan/France/171376/2017(H5N8) | 2016-2017 | West Europe | Wild Anseriformes | H5N8 |
| A/swan/France/171377/2017(H5N8) | 2016-2017 | West Europe | Wild Anseriformes | H5N8 |
| A/chicken/Italy/17VIR2658-3/2017(H5N8) | 2016-2017 | West Europe | Domestic Galliformes | H5N8 |
| A/chicken/Italy/17VIR2788/2017(H5N8) | 2016-2017 | West Europe | Domestic Galliformes | H5N8 |
| A/turkey/Italy/17VIR2722-29/2017(H5N8) | 2016-2017 | West Europe | Domestic Galliformes | H5N8 |
| A/goose/Bangladesh/19D820/2017(H5N6) | 2016-2017 | West Central Asia | Domestic Anseriformes | H5N6 |
| A/Duck/AL-Minia/1777FAO-S/2017(H5N8) | 2016-2017 | Africa | Domestic Anseriformes | H5N8 |
| A/chicken/Egypt/FL34/2017(H5N8) | 2016-2017 | Africa | Domestic Galliformes | H5N8 |
| A/duck/Egypt/F446/2017(H5N8) | 2016-2017 | Africa | Domestic Anseriformes | H5N8 |
| A/chicken/Italy/17VIR3078/2017(H5N8) | 2016-2017 | West Europe | Domestic Galliformes | H5N8 |
| A/Goose/Hungary/15729/2017(H5N8) | 2016-2017 | East Europe | Domestic Anseriformes | H5N8 |
| A/turkey/Italy/17VIR3192-1/2017(H5N8) | 2016-2017 | West Europe | Domestic Galliformes | H5N8 |
| A/chicken/Egypt/Q13936B/2017(H5N8) | 2016-2017 | Africa | Domestic Galliformes | H5N8 |
| A/chicken/Egypt/Q13941B/2017(H5N8) | 2016-2017 | Africa | Domestic Galliformes | H5N8 |
| A/chicken/Egypt/Q13944C/2017(H5N8) | 2016-2017 | Africa | Domestic Galliformes | H5N8 |
| A/Great Crested Grebe/Qinghai/a737/2017(H5N8) | 2016-2017 | China | Other wild species | H5N8 |
| A/chicken/Rostov-on-Don/44/2017(H5N8) | 2016-2017 | East Europe | Domestic Galliformes | H5N8 |
| A/Goose/Hungary/17051/2017(H5N8) | 2016-2017 | East Europe | Domestic Anseriformes | H5N8 |
| A/Goose/Hungary/17261/2017(H5N8) | 2016-2017 | East Europe | Domestic Anseriformes | H5N8 |
| A/Goose/Hungary/17580/2017(H5N8) | 2016-2017 | East Europe | Domestic Anseriformes | H5N8 |
| A/Goose/Hungary/17985/2017(H5N8) | 2016-2017 | East Europe | Domestic Anseriformes | H5N8 |
| A/Grey seal/361-13/BalticPL/16 (H5N8) | 2016-2017 | East Europe | Mammal | H5N8 |
| A/swan/France/171585/2017(H5N8) | 2016-2017 | West Europe | Wild Anseriformes | H5N8 |
| A/swan/Italy/17VIR3594-3/2017(H5N8) | 2016-2017 | West Europe | Wild Anseriformes | H5N8 |
| A/chicken/Al-Minia/1785Fao-S/2017(H5N8) | 2016-2017 | Africa | Domestic Galliformes | H5N8 |
| A/chicken/Giza/1836CAL/2017(H5N8) | 2016-2017 | Africa | Domestic Galliformes | H5N8 |
| A/Duck/Ismalia/1719Fao-SI/2017(H5N8) | 2016-2017 | Africa | Domestic Anseriformes | H5N8 |
| A/duck/Bangladesh/19D849/2017(H5N6) | 2016-2017 | West Central Asia | Domestic Anseriformes | H5N6 |
| A/duck/Bangladesh/19D851/2017(H5N6) | 2016-2017 | West Central Asia | Domestic Anseriformes | H5N6 |
| A/duck/Bangladesh/19D852/2017(H5N6) | 2016-2017 | West Central Asia | Domestic Anseriformes | H5N6 |
| A/duck/Bangladesh/19D853/2017(H5N6) | 2016-2017 | West Central Asia | Domestic Anseriformes | H5N6 |
| A/duck/Bangladesh/19D857/2017(H5N6) | 2016-2017 | West Central Asia | Domestic Anseriformes | H5N6 |
| A/goose/Bangladesh/19D855/2017(H5N6) | 2016-2017 | West Central Asia | Domestic Anseriformes | H5N6 |
| A/chicken/Tatarstan/88/2017(H5N8) | 2016-2017 | Russian Federation Siberia | Domestic Galliformes | H5N8 |
| A/chicken/Egypt/CA137/2017(H5N8) | 2016-2017 | Africa | Domestic Galliformes | H5N8 |
| A/chicken/Egypt/M14081D/2017(H5N8) | 2016-2017 | Africa | Domestic Galliformes | H5N8 |
| A/chicken/Tatarstan/112/2017(H5N8) | 2016-2017 | Russian Federation Siberia | Domestic Galliformes | H5N8 |
| A/duck/Democratic Republic of the Congo/17RS882-40/2017(H5N8) | 2016-2017 | Africa | Domestic Anseriformes | H5N8 |
| A/Bar-headed Goose/Qinghai/a230/2017(H5N8) | 2016-2017 | China | Wild Anseriformes | H5N8 |
| A/Bar-headed Goose/Qinghai/a765/2017(H5N8) | 2016-2017 | China | Wild Anseriformes | H5N8 |
| A/duck/Democratic Republic of the Congo/17RS882-33/2017(H5N8) | 2016-2017 | Africa | Domestic Anseriformes | H5N8 |
| A/duck/Democratic Republic of the Congo/17RS882-5/2017(H5N8) | 2016-2017 | Africa | Domestic Anseriformes | H5N8 |
| A/duck/Democratic Republic of the Congo/17RS882-29/2017(H5N8) | 2016-2017 | Africa | Domestic Anseriformes | H5N8 |
| A/Bar-headed Goose/Qinghai/a210/2017(H5N8) | 2016-2017 | China | Wild Anseriformes | H5N8 |
| A/Bar-headed Goose/Qinghai/a218/2017(H5N8) | 2016-2017 | China | Wild Anseriformes | H5N8 |
| A/Bar-headed Goose/Qinghai/a237/2017(H5N8) | 2016-2017 | China | Wild Anseriformes | H5N8 |
| A/Bar-headed Goose/Qinghai/B655/2017(H5N8) | 2016-2017 | China | Wild Anseriformes | H5N8 |
| A/Go/NL-Utrecht/17006881-001/2017(H5N5) | 2016-2017 | West Europe | Domestic Anseriformes | H5N8 |
| A/chicken/Zimbabwe/AI4935/2017(H5N8) | 2016-2017 | Africa | Domestic Galliformes | H5N8 |
| A/turkey/Italy/17VIR4407-1/2017(H5N8) | 2016-2017 | West Europe | Domestic Galliformes | H5N8 |
| A/Duck/Ismalia/1721Fao-SI/2017(H5N8) | 2016-2017 | Africa | Domestic Anseriformes | H5N8 |
| A/chicken/Korea/Gunsan/2017(H5N8) | 2016-2017 | Korea | Domestic Galliformes | H5N8 |
| A/duck/Egypt/N14200B/2017(H5N8) | 2016-2017 | Africa | Domestic Anseriformes | H5N8 |
| A/chicken/Belgium/5990/2017(H5N8) | 2016-2017 | West Europe | Domestic Galliformes | H5N8 |
| A/Guinea fowl/Belgium/6102/2017(H5N8) | 2016-2017 | West Europe | Other wild species | H5N8 |
| A/Brahma chicken/Belgium/6153/2017(H5N8) | 2016-2017 | West Europe | Domestic Galliformes | H5N8 |
| A/chicken/South Africa/Villiers/2017(H5N8) | 2016-2017 | Africa | Domestic Galliformes | H5N8 |
| A/Bar-headed Goose/Qinghai/a893/2017(H5N8) | 2016-2017 | China | Wild Anseriformes | H5N8 |
| A/chicken/South Africa/Standerton/2017(H5N8) | 2016-2017 | Africa | Domestic Galliformes | H5N8 |
| A/goose/France/171953/2017(H5N8) | 2016-2017 | West Europe | Domestic Anseriformes | H5N8 |
| A/swan/France/171960/2017(H5N8) | 2016-2017 | West Europe | Wild Anseriformes | H5N8 |
| A/chicken/France/172610/2017(H5N8) | 2016-2017 | West Europe | Domestic Galliformes | H5N8 |
| A/chicken/South Africa/436893/2017(H5N8) | 2016-2017 | Africa | Domestic Galliformes | H5N8 |
| A/Duck/Egypt/FAO-S78/2017(H5N8) | 2016-2017 | Africa | Domestic Anseriformes | H5N8 |
| A/Egyptian goose/South Africa/001/2017(H5N8) | 2016-2017 | Africa | Wild Anseriformes | H5N8 |
| A/duck/Italy/17VIR5769-5/2017(H5N8) | 2016-2017 | West Europe | Domestic Anseriformes | H5N8 |
| A/turkey/Italy/17VIR5773-2/2017(H5N8) | 2016-2017 | West Europe | Domestic Galliformes | H5N8 |
| A/chicken/Italy/17VIR5794-5/2017(H5N8) | 2016-2017 | West Europe | Domestic Galliformes | H5N8 |
| A/turkey/Italy/17VIR5878-3/2017(H5N8) | 2016-2017 | West Europe | Domestic Galliformes | H5N8 |
| A/mallard/Italy/17VIR6103-1/2017(H5N8) | 2016-2017 | West Europe | Wild Anseriformes | H5N8 |
| A/chicken/Italy/17VIR5986-1/2017(H5N8) | 2016-2017 | West Europe | Domestic Galliformes | H5N8 |
| A/turkey/Italy/17VIR5964-8/2017(H5N8) | 2016-2017 | West Europe | Domestic Galliformes | H5N8 |
| A/turkey/Italy/17VIR6111-16/2017(H5N8) | 2016-2017 | West Europe | Domestic Galliformes | H5N8 |
| A/chicken/South Africa/440638A/2017(H5N8) | 2016-2017 | Africa | Domestic Galliformes | H5N8 |
| A/chicken/South Africa/440638B/2017(H5N8) | 2016-2017 | Africa | Domestic Galliformes | H5N8 |
| A/turkey/Italy/17VIR6108-3/2017(H5N8) | 2016-2017 | West Europe | Domestic Galliformes | H5N8 |
| A/Ostrich/South Africa/S2017/08 0046 AF/2017(H5N8) | 2016-2017 | Africa | Other wild species | H5N8 |
| A/Ostrich/South Africa/S2017/08 0046 P2/2017(H5N8) | 2016-2017 | Africa | Other wild species | H5N8 |
| A/Ostrich/South Africa/S2017/08 0046 P3/2017(H5N8) | 2016-2017 | Africa | Other wild species | H5N8 |
| A/Ostrich/South Africa/S2017/08 0047 P7/2017(H5N8) | 2016-2017 | Africa | Other wild species | H5N8 |
| A/chicken/South Africa/441587/2017(H5N8) | 2016-2017 | Africa | Domestic Galliformes | H5N8 |
| A/chicken/South Africa/MC002/2017(H5N8) | 2016-2017 | Africa | Domestic Galliformes | H5N8 |
| A/turkey/Italy/17VIR6355-1/2017(H5N8) | 2016-2017 | West Europe | Domestic Galliformes | H5N8 |
| A/turkey/Italy/17VIR6356-1/2017(H5N8) | 2016-2017 | West Europe | Domestic Galliformes | H5N8 |
| A/turkey/Italy/17VIR6357-2/2017(H5N8) | 2016-2017 | West Europe | Domestic Galliformes | H5N8 |
| A/goose/Italy/17VIR6358-3/2017(H5N8) | 2016-2017 | West Europe | Domestic Anseriformes | H5N8 |
| A/chicken/South Africa/441839/2017(H5N8) | 2016-2017 | Africa | Domestic Galliformes | H5N8 |
| A/turkey/Italy/17VIR6469-2/2017(H5N8) | 2016-2017 | West Europe | Domestic Galliformes | H5N8 |
| A/ostrich/South Africa/17080046/2017(H5N8) | 2016-2017 | Africa | Other wild species | H5N8 |
| A/mute swan/Switzerland/V0244.2-L02307/2017(H5N8) | 2016-2017 | West Europe | Wild Anseriformes | H5N8 |
| A/Ostrich/South Africa/S2017/08 0161 P8/2017(H5N8) | 2016-2017 | Africa | Other wild species | H5N8 |
| A/Guineafowl/South Africa/S2017/08 0190 9/2017(H5N8) | 2016-2017 | Africa | Other wild species | H5N8 |
| A/Speckled pigeon/South Africa/08-004B/2017(H5N8) | 2016-2017 | Africa | Other wild species | H5N8 |
| A/Sacred ibis/South Africa/009/2017(H5N8) | 2016-2017 | Africa | Other wild species | H5N8 |
| A/Guinea fowl/South Africa/17080243/2017(H5N8) | 2016-2017 | Africa | Other wild species | H5N8 |
| A/Guinea fowl/South Africa/17080274/2017(H5N8) | 2016-2017 | Africa | Other wild species | H5N8 |
| A/Guineafowl/South Africa/S2017/08 0243 P1/2017(H5N8) | 2016-2017 | Africa | Other wild species | H5N8 |
| A/Guineafowl/South Africa/S2017/08 0243 P2/2017(H5N8) | 2016-2017 | Africa | Other wild species | H5N8 |
| A/Guineafowl/South Africa/S2017/08 0274 P1/2017(H5N8) | 2016-2017 | Africa | Other wild species | H5N8 |
| A/Ostrich/South Africa/S2017/08 0268 P2/2017(H5N8) | 2016-2017 | Africa | Other wild species | H5N8 |
| A/Ostrich/South Africa/S2017/08 0268 P9/2017(H5N8) | 2016-2017 | Africa | Other wild species | H5N8 |
| A/swan/Italy/17VIR9836-1/2017(H5N8) | 2016-2017 | West Europe | Wild Anseriformes | H5N8 |
| A/Wildbirds Guineafowl Makou Egyptian Geese/South Africa/S2017/08 0275 P1/2017(H5N8) | 2016-2017 | Africa | Wild Anseriformes | H5N8 |
| A/chicken/South Africa/443397/2017(H5N8) | 2016-2017 | Africa | Domestic Galliformes | H5N8 |
| A/dove/South Africa/17080324/2017(H5N8) | 2016-2017 | Africa | Other wild species | H5N8 |
| A/pigeon/South Africa/17080323/2017(H5N8) | 2016-2017 | Africa | Domestic Galliformes | H5N8 |
| A/Pigeon/South Africa/S2017/08 0323 P1/2017(H5N8) | 2016-2017 | Africa | Domestic Galliformes | H5N8 |
| A/Pigeon/South Africa/S2017/08 0324 24/2017(H5N8) | 2016-2017 | Africa | Domestic Galliformes | H5N8 |
| A/pheasant/Italy/17VIR7065-21/2017(H5N8) | 2016-2017 | West Europe | Other wild species | H5N8 |
| A/chicken/South Africa/17080336/2017(H5N8) | 2016-2017 | Africa | Domestic Galliformes | H5N8 |
| A/Chicken/South Africa/S2017/08 0336 P1/2017(H5N8) | 2016-2017 | Africa | Domestic Galliformes | H5N8 |
| A/Chicken/South Africa/S2017/08 0336 P3/2017(H5N8) | 2016-2017 | Africa | Domestic Galliformes | H5N8 |
| A/turkey/Italy/17VIR6882-1/2017(H5N8) | 2016-2017 | West Europe | Domestic Galliformes | H5N8 |
| A/Duck/Egypt/FD189/2017(H5N8) | 2016-2017 | Africa | Domestic Anseriformes | H5N8 |
| A/Duck/South Africa/S2017/08 0340 P1/2017(H5N8) | 2016-2017 | Africa | Domestic Anseriformes | H5N8 |
| A/Duck/South Africa/S2017/08 0340 P2/2017(H5N8) | 2016-2017 | Africa | Domestic Anseriformes | H5N8 |
| A/Ostrich/South Africa/S2017/08 0361 P17/2017(H5N8) | 2016-2017 | Africa | Other wild species | H5N8 |
| A/Ostrich/South Africa/S2017/08 0362 P10/2017(H5N8) | 2016-2017 | Africa | Other wild species | H5N8 |
| A/Ostrich/South Africa/S2017/08 0362 P11/2017(H5N8) | 2016-2017 | Africa | Other wild species | H5N8 |
| A/Ostrich/South Africa/S2017/08 0362 P7/2017(H5N8) | 2016-2017 | Africa | Other wild species | H5N8 |
| A/Ostrich/South Africa/S2017/08 0362 P8 33/2017(H5N8) | 2016-2017 | Africa | Other wild species | H5N8 |
| A/Ostrich/South Africa/S2017/08 0362 P8 34/2017(H5N8) | 2016-2017 | Africa | Other wild species | H5N8 |
| A/Pekin duck/South Africa/17080340/2017(H5N8) | 2016-2017 | Africa | Wild Anseriformes | H5N8 |
| A/turkey/Italy/17VIR6928-5/2017(H5N8) | 2016-2017 | West Europe | Domestic Galliformes | H5N8 |
| A/turkey/Italy/17VIR6930-4/2017(H5N8) | 2016-2017 | West Europe | Domestic Galliformes | H5N8 |
| A/Chicken/South Africa/S2017/08 0416 38/2017(H5N8) | 2016-2017 | Africa | Domestic Galliformes | H5N8 |
| A/Duck/South Africa/S2017/08 0481 P2/2017(H5N8) | 2016-2017 | Africa | Domestic Anseriformes | H5N8 |
| A/Pekin duck/South Africa/17080481/2017(H5N8) | 2016-2017 | Africa | Wild Anseriformes | H5N8 |
| A/chicken/Italy/17VIR7062-1/2017(H5N8) | 2016-2017 | West Europe | Domestic Galliformes | H5N8 |
| A/swan/Italy/17VIR7064-1/2017(H5N8) | 2016-2017 | West Europe | Wild Anseriformes | H5N8 |
| A/Geese/South Africa/S2017/08 0520 46/2017(H5N8) | 2016-2017 | Africa | Domestic Anseriformes | H5N8 |
| A/Swan/South Africa/17080517/2017(H5N8) | 2016-2017 | Africa | Wild Anseriformes | H5N8 |
| A/Swan/South Africa/S2017/08 0517 P2/2017(H5N8) | 2016-2017 | Africa | Wild Anseriformes | H5N8 |
| A/turkey/Italy/17VIR7197-1/2017(H5N8) | 2016-2017 | West Europe | Domestic Galliformes | H5N8 |
| A/chicken/South Africa/17080561/2017(H5N8) | 2016-2017 | Africa | Domestic Galliformes | H5N8 |
| A/Chicken/South Africa/S2017/08 0561 P1/2017(H5N8) | 2016-2017 | Africa | Domestic Galliformes | H5N8 |
| A/Chicken/South Africa/S2017/08 0561 P2/2017(H5N8) | 2016-2017 | Africa | Domestic Galliformes | H5N8 |
| A/Geese/South Africa/S2017/08 0558 P1/2017(H5N8) | 2016-2017 | Africa | Domestic Anseriformes | H5N8 |
| A/Geese/South Africa/S2017/08 0558 P2/2017(H5N8) | 2016-2017 | Africa | Domestic Anseriformes | H5N8 |
| A/chicken/South Africa/17080581/2017(H5N8) | 2016-2017 | Africa | Domestic Galliformes | H5N8 |
| A/Chicken/South Africa/S2017/08 0581 P1/2017(H5N8) | 2016-2017 | Africa | Domestic Galliformes | H5N8 |
| A/Chicken/South Africa/S2017/08 0581 P2/2017(H5N8) | 2016-2017 | Africa | Domestic Galliformes | H5N8 |
| A/chicken/Netherlands/2113/2017(H5N8) | 2016-2017 | West Europe | Domestic Galliformes | H5N8 |
| A/swan/Italy/17VIR8513/2017(H5N8) | 2016-2017 | West Europe | Wild Anseriformes | H5N8 |
| A/turkey/Italy/17VIR7337-1/2017(H5N8) | 2016-2017 | West Europe | Domestic Galliformes | H5N8 |
| A/chicken/South Africa/17090050/2017(H5N8) | 2016-2017 | Africa | Domestic Galliformes | H5N8 |
| A/Chicken/South Africa/S2017/09 0050 56/2017(H5N8) | 2016-2017 | Africa | Domestic Galliformes | H5N8 |
| A/Domestic goose/South Africa/17090065/2017(H5N8) | 2016-2017 | Africa | Domestic Anseriformes | H5N8 |
| A/Geese/South Africa/S2017/09 0055 P1/2017(H5N8) | 2016-2017 | Africa | Domestic Anseriformes | H5N8 |
| A/Geese/South Africa/S2017/09 0055 P2/2017(H5N8) | 2016-2017 | Africa | Domestic Anseriformes | H5N8 |
| A/Geese/South Africa/S2017/09 0065 P1/2017(H5N8) | 2016-2017 | Africa | Domestic Anseriformes | H5N8 |
| A/Geese/South Africa/S2017/09 0065 P2/2017(H5N8) | 2016-2017 | Africa | Domestic Anseriformes | H5N8 |
| A/chicken/South Africa/17090100/2017(H5N8) | 2016-2017 | Africa | Domestic Galliformes | H5N8 |
| A/chicken/South Africa/17090108/2017(H5N8) | 2016-2017 | Africa | Domestic Galliformes | H5N8 |
| A/Chicken/South Africa/S2017/09 0184 62/2017(H5N8) | 2016-2017 | Africa | Domestic Galliformes | H5N8 |
| A/Chicken/South Africa/S2017/09 0184 63/2017(H5N8) | 2016-2017 | Africa | Domestic Galliformes | H5N8 |
| A/Crow/Aghakhan/2017(H5N8) | 2016-2017 | West Central Asia | Other wild species | H5N8 |
| A/goose/Italy/17VIR7626-5/2017(H5N8) | 2016-2017 | West Europe | Domestic Anseriformes | H5N8 |
| A/chicken/South Africa/17090202/2017(H5N8) | 2016-2017 | Africa | Domestic Galliformes | H5N8 |
| A/chicken/South Africa/448475/2017(H5N8) | 2016-2017 | Africa | Domestic Galliformes | H5N8 |
| A/chicken/Iran/18VIR2027-01/2017(H5N8) | 2016-2017 | West Central Asia | Domestic Galliformes | H5N8 |
| A/chicken/Iran/18VIR2027-02/2017(H5N8) | 2016-2017 | West Central Asia | Domestic Galliformes | H5N8 |
| A/chicken/South Africa/17090325/2017(H5N8) | 2016-2017 | Africa | Domestic Galliformes | H5N8 |
| A/chicken/South Africa/449300/2017(H5N8) | 2016-2017 | Africa | Domestic Galliformes | H5N8 |
| A/chicken/South Africa/17090335/2017(H5N8) | 2016-2017 | Africa | Domestic Galliformes | H5N8 |
| A/chicken/South Africa/17090348/2017(H5N8) | 2016-2017 | Africa | Domestic Galliformes | H5N8 |
| A/chicken/South Africa/449418/2017(H5N8) | 2016-2017 | Africa | Domestic Galliformes | H5N8 |
| A/chicken/South Africa/449443/2017(H5N8) | 2016-2017 | Africa | Domestic Galliformes | H5N8 |
| A/turkey/South Africa/450199/2017(H5N8) | 2016-2017 | Africa | Domestic Galliformes | H5N8 |
| A/chicken/South Africa/115370/2017(H5N8) | 2016-2017 | Africa | Domestic Galliformes | H5N8 |
| A/duck/Italy/17VIR8200-12/2017(H5N8) | 2016-2017 | West Europe | Domestic Anseriformes | H5N8 |
| A/duck/Italy/17VIR8349/2017(H5N8) | 2016-2017 | West Europe | Domestic Anseriformes | H5N8 |
| A/turkey/Italy/17VIR8199-1/2017(H5N8) | 2016-2017 | West Europe | Domestic Galliformes | H5N8 |
| A/chicken/South Africa/450628/2017(H5N8) | 2016-2017 | Africa | Domestic Galliformes | H5N8 |
| A/turkey/Italy/17VIR8275-3/2017(H5N8) | 2016-2017 | West Europe | Domestic Galliformes | H5N8 |
| A/chicken/Italy/17VIR8678/2017(H5N8) | 2016-2017 | West Europe | Domestic Galliformes | H5N8 |
| A/chicken/Italy/17VIR8783-5/2017(H5N8) | 2016-2017 | West Europe | Domestic Galliformes | H5N8 |
| A/chicken/Italy/17VIR8744-9/2017(H5N8) | 2016-2017 | West Europe | Domestic Galliformes | H5N8 |
| A/chicken/Italy/17VIR8821/2017(H5N8) | 2016-2017 | West Europe | Domestic Galliformes | H5N8 |
| A/turkey/Italy/17VIR8819-3/2017(H5N8) | 2016-2017 | West Europe | Domestic Galliformes | H5N8 |
| A/chicken/Italy/17VIR8885/2017(H5N8) | 2016-2017 | West Europe | Domestic Galliformes | H5N8 |
| A/swan/Italy/17VIR9017/2017(H5N8) | 2016-2017 | West Europe | Wild Anseriformes | H5N8 |
| A/swan/Italy/17VIR9038-1/2017(H5N8) | 2016-2017 | West Europe | Wild Anseriformes | H5N8 |
| A/swan/Italy/17VIR9038-2/2017(H5N8) | 2016-2017 | West Europe | Wild Anseriformes | H5N8 |
| A/turkey/Italy/17VIR8886-3/2017(H5N8) | 2016-2017 | West Europe | Domestic Galliformes | H5N8 |
| A/chicken/Egypt/S167/2017(H5N8) | 2016-2017 | Africa | Domestic Galliformes | H5N8 |
| A/chicken/Italy/17VIR9048-2/2017(H5N8) | 2016-2017 | West Europe | Domestic Galliformes | H5N8 |
| A/turkey/Italy/17VIR9049-2/2017(H5N8) | 2016-2017 | West Europe | Domestic Galliformes | H5N8 |
| A/turkey/Italy/17VIR9106-2/2017(H5N8) | 2016-2017 | West Europe | Domestic Galliformes | H5N8 |
| A/chicken/Iran/18VIR2027-14/2017(H5N8) | 2016-2017 | West Central Asia | Domestic Galliformes | H5N8 |
| A/chicken/Iran/18VIR2027-04/2017(H5N8) | 2016-2017 | West Central Asia | Domestic Galliformes | H5N8 |
| A/chicken/Italy/17VIR9107/2017(H5N8) | 2016-2017 | West Europe | Domestic Galliformes | H5N8 |
| A/chicken/Italy/17VIR9113/2017(H5N8) | 2016-2017 | West Europe | Domestic Galliformes | H5N8 |
| A/duck/Italy/17VIR8898/2017(H5N8) | 2016-2017 | West Europe | Domestic Anseriformes | H5N8 |
| A/duck/Bulgaria/Dobrich/407/2017(H5N8) | 2016-2017 | East Europe | Domestic Anseriformes | H5N8 |
| A/chicken/Italy/17VIR9456/2017(H5N8) | 2016-2017 | West Europe | Domestic Galliformes | H5N8 |
| A/chicken/Rostov-on-Don/1321/2017(H5N8) | 2016-2017 | East Europe | Domestic Galliformes | H5N8 |
| A/turkey/Italy/17VIR9245/2017(H5N8) | 2016-2017 | West Europe | Domestic Galliformes | H5N8 |
| A/turkey/Italy/17VIR9246/2017(H5N8) | 2016-2017 | West Europe | Domestic Galliformes | H5N8 |
| A/chicken/Bulgaria/Haskovo/411/2017(H5N8) | 2016-2017 | East Europe | Domestic Galliformes | H5N8 |
| A/quail/Italy/17VIR9455-2/2017(H5N8) | 2016-2017 | West Europe | Domestic Galliformes | H5N8 |
| A/chicken/Iran/18VIR2027-03/2017(H5N8) | 2016-2017 | West Central Asia | Domestic Galliformes | H5N8 |
| A/chicken/Iran/18VIR2027-05/2017(H5N8) | 2016-2017 | West Central Asia | Domestic Galliformes | H5N8 |
| A/chicken/Iran/18VIR2027-06/2017(H5N8) | 2016-2017 | West Central Asia | Domestic Galliformes | H5N8 |
| A/kestrel/Italy/17VIR9840/2017(H5N8) | 2016-2017 | West Europe | Other wild species | H5N8 |
| A/pigeon/Italy/17VIR9842/2017(H5N8) | 2016-2017 | West Europe | Domestic Galliformes | H5N8 |
| A/wild goose/Italy/17VIR9841/2017(H5N8) | 2016-2017 | West Europe | Wild Anseriformes | H5N8 |
| A/duck/Italy/17VIR9533/2017(H5N8) | 2016-2017 | West Europe | Domestic Anseriformes | H5N8 |
| A/ostrich/South Africa/002/2017(H5N8) | 2016-2017 | Africa | Other wild species | H5N8 |
| A/chicken/Italy/17VIR9679/2017(H5N8) | 2016-2017 | West Europe | Domestic Galliformes | H5N8 |
| A/chicken/Italy/17VIR9680/2017(H5N8) | 2016-2017 | West Europe | Domestic Galliformes | H5N8 |
| A/turkey/Italy/17VIR9699/2017(H5N8) | 2016-2017 | West Europe | Domestic Galliformes | H5N8 |
| A/turkey/Italy/17VIR9773/2017(H5N8) | 2016-2017 | West Europe | Domestic Galliformes | H5N8 |
| A/turkey/Italy/17VIR9775-1/2017(H5N8) | 2016-2017 | West Europe | Domestic Galliformes | H5N8 |
| A/chicken/South Korea/AIV49/2017(H5N6) | 2016-2017 | Korea | Domestic Galliformes | H5N6 |
| A/turkey/Italy/17VIR9908/2017(H5N8) | 2016-2017 | West Europe | Domestic Galliformes | H5N8 |
| A/chicken/Bulgaria/Sliven/432/2017(H5N8) | 2016-2017 | East Europe | Domestic Galliformes | H5N8 |
| A/chicken/Italy/17VIR9868-1/2017(H5N8) | 2016-2017 | West Europe | Domestic Galliformes | H5N8 |
| A/turkey/Italy/17VIR9905-1/2017(H5N8) | 2016-2017 | West Europe | Domestic Galliformes | H5N8 |
| A/chicken/Italy/17VIR9846-4/2017(H5N8) | 2016-2017 | West Europe | Domestic Galliformes | H5N8 |
| A/chicken/Italy/17VIR9941-1/2017(H5N8) | 2016-2017 | West Europe | Domestic Galliformes | H5N8 |
| A/duck/Bulgaria/Yambol/436/2017(H5N8) | 2016-2017 | East Europe | Domestic Anseriformes | H5N8 |
| A/duck/Hunan/11.30 YYGK74E3-OC/2017(H5N8) | 2016-2017 | China | Domestic Anseriformes | H5N8 |
| A/duck/Hunan/11.30 YYGK75E3-OC/2017(H5N6) | 2016-2017 | China | Domestic Anseriformes | H5N6 |
| A/duck/Italy/17VIR9906-1/2017(H5N8) | 2016-2017 | West Europe | Domestic Anseriformes | H5N8 |
| A/duck/Italy/17VIR9907-2/2017(H5N8) | 2016-2017 | West Europe | Domestic Anseriformes | H5N8 |
| A/turkey/Italy/17VIR9942-2/2017(H5N8) | 2016-2017 | West Europe | Domestic Galliformes | H5N8 |
| A/mute swan/Shimane/3211A001/2017(H5N6) | 2016-2017 | Japan | Wild Anseriformes | H5N6 |
| A/chicken/Italy/17VIR10083/2017(H5N8) | 2016-2017 | West Europe | Domestic Galliformes | H5N8 |
| A/turkey/Italy/17VIR10133-8/2017(H5N8) | 2016-2017 | West Europe | Domestic Galliformes | H5N8 |
| A/chicken/China/HJ11/2017(H5N6) | 2016-2017 | China | Domestic Galliformes | H5N6 |
| A/chicken/Italy/17VIR10053/2017(H5N8) | 2016-2017 | West Europe | Domestic Galliformes | H5N8 |
| A/tufted duck/Shimane/3211TY001/2017(H5N6) | 2016-2017 | Japan | Wild Anseriformes | H5N6 |
| A/chicken/Italy/17VIR10082/2017(H5N8) | 2016-2017 | West Europe | Domestic Galliformes | H5N8 |
| A/chicken/Italy/17VIR10130-2/2017(H5N8) | 2016-2017 | West Europe | Domestic Galliformes | H5N8 |
| A/chicken/Italy/17VIR10131-1/2017(H5N8) | 2016-2017 | West Europe | Domestic Galliformes | H5N8 |
| A/chicken/Italy/17VIR10132-1/2017(H5N8) | 2016-2017 | West Europe | Domestic Galliformes | H5N8 |
| A/mute swan/Shimane/3211A002/2017(H5N6) | 2016-2017 | Japan | Wild Anseriformes | H5N6 |
| A/chicken/Rostov-on-Don/1598/2017(H5N8) | 2016-2017 | East Europe | Domestic Galliformes | H5N8 |
| A/duck/Korea/HD1/2017(H5N6) | 2016-2017 | Korea | Domestic Anseriformes | H5N6 |
| A/duck/Denmark/19062-5Sp1c/2017(H5N8) | 2016-2017 | West Europe | Domestic Anseriformes | H5N8 |
| A/chicken/Italy/17VIR10408/2017(H5N8) | 2016-2017 | West Europe | Domestic Galliformes | H5N8 |
| A/duck/Bulgaria/Stara-Zagora/623/2017(H5N8) | 2016-2017 | East Europe | Domestic Anseriformes | H5N8 |
| A/chicken/Italy/17VIR10437-2/2017(H5N8) | 2016-2017 | West Europe | Domestic Galliformes | H5N8 |
| A/gull/France/161176/2017(H5N8) | 2016-2017 | West Europe | Charadriiformes | H5N8 |
| A/mallard/Korea/Jeju-H24/2017(H5N6) | 2016-2017 | Korea | Wild Anseriformes | H5N6 |
| A/goose/Xinjiang/11.29 WLMQXL001-O/2017(H5N6) | 2016-2017 | China | Domestic Anseriformes | H5N6 |
| A/Duck/Al-Monfia/1727/2017(H5N8) | 2016-2017 | Africa | Domestic Anseriformes | H5N8 |
| A/duck/Italy/17VIR10767-13/2017(H5N8) | 2016-2017 | West Europe | Domestic Anseriformes | H5N8 |
| A/duck/Saudi Arabia/KFU-HKU3616 2017/2017(H5N8) | 2016-2017 | West Central Asia | Domestic Anseriformes | H5N8 |
| A/duck/Saudi Arabia/KFU-HKU3617 2017/2017(H5N8) | 2016-2017 | West Central Asia | Domestic Anseriformes | H5N8 |
| A/duck/Saudi Arabia/KFU-HKU3618 2017/2017(H5N8) | 2016-2017 | West Central Asia | Domestic Anseriformes | H5N8 |
| A/duck/Saudi Arabia/KFU-HKU3619 2017/2017(H5N8) | 2016-2017 | West Central Asia | Domestic Anseriformes | H5N8 |
| A/ostrich/Saudi Arabia/KFU-HKU3590 2017/2017(H5N8) | 2016-2017 | West Central Asia | Other wild species | H5N8 |
| A/ostrich/Saudi Arabia/KFU-HKU3591 2017/2017(H5N8) | 2016-2017 | West Central Asia | Other wild species | H5N8 |
| A/ostrich/Saudi Arabia/KFU-HKU3592 2017/2017(H5N8) | 2016-2017 | West Central Asia | Other wild species | H5N8 |
| A/ostrich/Saudi Arabia/KFU-HKU3593 2017/2017(H5N8) | 2016-2017 | West Central Asia | Other wild species | H5N8 |
| A/ostrich/Saudi Arabia/KFU-HKU3594 2017/2017(H5N8) | 2016-2017 | West Central Asia | Other wild species | H5N8 |
| A/pigeon/Saudi Arabia/KFU-HKU3598 2017/2017(H5N8) | 2016-2017 | West Central Asia | Domestic Galliformes | H5N8 |
| A/pigeon/Saudi Arabia/KFU-HKU3599 2017/2017(H5N8) | 2016-2017 | West Central Asia | Domestic Galliformes | H5N8 |
| A/pigeon/Saudi Arabia/KFU-HKU3600 2017/2017(H5N8) | 2016-2017 | West Central Asia | Domestic Galliformes | H5N8 |
| A/spoonbill/Taiwan/DB645/2017(H5N6) | 2016-2017 | China | Charadriiformes | H5N6 |
| A/Duck/Netherlands/17017236-001-005/2017(H5N6) | 2016-2017 | West Europe | Domestic Anseriformes | H5N6 |
| A/Duck/Netherlands/17017237-001-005/2017(H5N6) | 2016-2017 | West Europe | Domestic Anseriformes | H5N6 |
| A/Mute Swan/Netherlands/17017367-012/2017(H5N6) | 2016-2017 | West Europe | Wild Anseriformes | H5N6 |
| A/Mute Swan/Netherlands/17017377-001/2017(H5N6) | 2016-2017 | West Europe | Wild Anseriformes | H5N6 |
| A/Tufted Duck/Netherlands/17017367-007/2017(H5N6) | 2016-2017 | West Europe | Wild Anseriformes | H5N6 |
| A/chicken/Iran/18VIR2027-10/2017(H5N8) | 2016-2017 | West Central Asia | Domestic Galliformes | H5N8 |
| A/duck/Korea/H35/2017(H5N6) | 2016-2017 | Korea | Domestic Anseriformes | H5N6 |
| A/turkey/Italy/17VIR10983-22/2017(H5N8) | 2016-2017 | West Europe | Domestic Galliformes | H5N8 |
| A/Anas platyrhynchos/Korea/W612/2017(H5N6) | 2016-2017 | Korea | Wild Anseriformes | H5N6 |
| A/Anas platyrhynchos/Korea/W613/2017(H5N6) | 2016-2017 | Korea | Wild Anseriformes | H5N6 |
| A/Anas platyrhynchos/Korea/W614/2017(H5N6) | 2016-2017 | Korea | Wild Anseriformes | H5N6 |
| A/Anas platyrhynchos/Korea/W615/2017(H5N6) | 2016-2017 | Korea | Wild Anseriformes | H5N6 |
| A/Bird/Netherlands/17017775-035-039/2017(H5N6) | 2016-2017 | West Europe | Other wild species | H5N6 |
| A/chicken/Iran/18VIR2027-08/2017(H5N8) | 2016-2017 | West Central Asia | Domestic Galliformes | H5N8 |
| A/Guineafowl/Netherlands/17017775-020-024/2017(H5N6) | 2016-2017 | West Europe | Other wild species | H5N6 |
| A/Peacock/Netherlands/17017775-064-068/2017(H5N6) | 2016-2017 | West Europe | Other wild species | H5N6 |
| A/Great Black-backed Gull/Netherlands/1/2017 (H5N6) | 2016-2017 | West Europe | Charadriiformes | H5N6 |
| A/duck/Korea/H56/2017(H5N6) | 2016-2017 | Korea | Domestic Anseriformes | H5N6 |
| A/mandarin duck/Korea/H69/2017(H5N6) | 2016-2017 | Korea | Wild Anseriformes | H5N6 |
| A/Mute swan/Netherlands/17017903-002/2017(H5N6) | 2016-2017 | West Europe | Wild Anseriformes | H5N6 |
| A/mandarin duck/Korea/H71/2017(H5N6) | 2016-2017 | Korea | Wild Anseriformes | H5N6 |
| A/chicken/Iran/18VIR2027-09/2017(H5N8) | 2016-2017 | West Central Asia | Domestic Galliformes | H5N8 |
| A/chicken/Iran/18VIR2027-15/2017(H5N8) | 2016-2017 | West Central Asia | Domestic Galliformes | H5N8 |
| A/chicken/Kostroma/1717/2017(H5N2) | 2016-2017 | East Europe | Domestic Galliformes | H5N2 |
| A/chicken/Kostroma/1718/2017(H5N2) | 2016-2017 | East Europe | Domestic Galliformes | H5N2 |
| A/chicken/Kostroma/1719/2017(H5N2) | 2016-2017 | East Europe | Domestic Galliformes | H5N2 |
| A/chicken/Kostroma/1720/2017(H5N2) | 2016-2017 | East Europe | Domestic Galliformes | H5N2 |
| A/chicken/Kostroma/1721/2017(H5N2) | 2016-2017 | East Europe | Domestic Galliformes | H5N2 |
| A/duck/Korea/H70/2017(H5N6) | 2016-2017 | Korea | Domestic Anseriformes | H5N6 |
| A/Mallard/Korea/K17-1825/2017(H5N6) | 2016-2017 | Korea | Wild Anseriformes | H5N6 |
| A/Mandarin duck/Korea/K17-1815/2017(H5N6) | 2016-2017 | Korea | Wild Anseriformes | H5N6 |
| A/Mandarin duck/Korea/K17-1817/2017(H5N6) | 2016-2017 | Korea | Wild Anseriformes | H5N6 |
| A/Mandarin duck/Korea/K17-1826/2017(H5N6) | 2016-2017 | Korea | Wild Anseriformes | H5N6 |
| A/Mandarin duck/Korea/K17-1828/2017(H5N6) | 2016-2017 | Korea | Wild Anseriformes | H5N6 |
| A/Spoonbill/HK/17-18259/2017(H5N6) | 2016-2017 | China | Charadriiformes | H5N6 |
| A/Mandarin duck/Korea/K17-1862/2017(H5N6) | 2016-2017 | Korea | Wild Anseriformes | H5N6 |
| A/Mandarin duck/Korea/K17-1866/2017(H5N6) | 2016-2017 | Korea | Wild Anseriformes | H5N6 |
| A/Mandarin duck/Korea/K17-1869/2017(H5N6) | 2016-2017 | Korea | Wild Anseriformes | H5N6 |
| A/Mandarin duck/Korea/K17-1873/2017(H5N6) | 2016-2017 | Korea | Wild Anseriformes | H5N6 |
| A/Mandarin duck/Korea/K17-1879/2017(H5N6) | 2016-2017 | Korea | Wild Anseriformes | H5N6 |
| A/Mandarin duck/Korea/K17-1881/2017(H5N6) | 2016-2017 | Korea | Wild Anseriformes | H5N6 |
| A/Mandarin duck/Korea/K17-1885/2017(H5N6) | 2016-2017 | Korea | Wild Anseriformes | H5N6 |
| A/Mandarin duck/Korea/K17-1887/2017(H5N6) | 2016-2017 | Korea | Wild Anseriformes | H5N6 |
| A/Mandarin duck/Korea/K17-1889/2017(H5N6) | 2016-2017 | Korea | Wild Anseriformes | H5N6 |
| A/Mandarin duck/Korea/K17-1891/2017(H5N6) | 2016-2017 | Korea | Wild Anseriformes | H5N6 |
| A/Mandarin duck/Korea/K17-1893/2017(H5N6) | 2016-2017 | Korea | Wild Anseriformes | H5N6 |
| A/Mandarin duck/Korea/K17-1894/2017(H5N6) | 2016-2017 | Korea | Wild Anseriformes | H5N6 |
| A/Mandarin duck/Korea/K17-1896/2017(H5N6) | 2016-2017 | Korea | Wild Anseriformes | H5N6 |
| A/chicken/Central Russia/1/2017(H5N2) | 2016-2017 | Russian Federation Siberia | Domestic Galliformes | H5N2 |
| A/Fujian-Sanyuan/21099/2017(H5N6) | 2016-2017 | China | Human | H5N6 |
| A/duck/Korea/H80/2017(H5N6) | 2016-2017 | Korea | Domestic Anseriformes | H5N6 |
| A/duck/Korea/H81/2017(H5N6) | 2016-2017 | Korea | Domestic Anseriformes | H5N6 |
| A/Armenian Gull/Republic of Georgia/4/2017(H5N6) | 2016-2017 | West Central Asia | Charadriiformes | H5N6 |
| A/common pochard/Germany-BY/AR09-18-L02421/2017(H5N6) | 2016-2017 | West Europe | Wild Anseriformes | H5N6 |
| A/duck/Korea/H103/2017(H5N6) | 2016-2017 | Korea | Domestic Anseriformes | H5N6 |
| A/duck/Korea/H107/2017(H5N6) | 2016-2017 | Korea | Domestic Anseriformes | H5N6 |
| A/mandarin duck/Korea/H119-3/2017(H5N6) | 2016-2017 | Korea | Wild Anseriformes | H5N6 |
| A/mandarin duck/Korea/H119-4/2017(H5N6) | 2016-2017 | Korea | Wild Anseriformes | H5N6 |
| A/mute swan/England/AVP 18 001986/2017(H5N6) | 2016-2017 | West Europe | Wild Anseriformes | H5N6 |
| A/chicken/AL Suez/184/2018(H5N8) | 2018-2019 | Africa | Domestic Galliformes | H5N8 |
| A/chicken/Al-Minia/183/2018(H5N8) | 2018-2019 | Africa | Domestic Galliformes | H5N8 |
| A/chicken/Al-Sharqia/1822FM/2018(H5N8) | 2018-2019 | Africa | Domestic Galliformes | H5N8 |
| A/Duck/EL-WadiAlgidid/185/2018(H5N8) | 2018-2019 | Africa | Domestic Anseriformes | H5N8 |
| A/duck/Korea/H125-2/2018(H5N6) | 2018-2019 | Korea | Domestic Anseriformes | H5N6 |
| A/Duck/Luxor/51/2018(H5N8) | 2018-2019 | Africa | Domestic Anseriformes | H5N8 |
| A/Duck/Luxor/62/2018(H5N8) | 2018-2019 | Africa | Domestic Anseriformes | H5N8 |
| A/duck/Korea/H125-1/2018(H5N6) | 2020-2022 | Korea | Domestic Anseriformes | H5N1 |
| A/Chicken/Egypt/AR539/2018(H5N8) | 2018-2019 | Africa | Domestic Galliformes | H5N8 |
| A/duck/Korea/H182/2018(H5N6) | 2018-2019 | Korea | Domestic Anseriformes | H5N6 |
| A/duck/Korea/H187/2018(H5N6) | 2018-2019 | Korea | Domestic Anseriformes | H5N6 |
| A/canada goose/England/AV58 18OPpoolEP1/2018(H5N6) | 2018-2019 | West Europe | Wild Anseriformes | H5N6 |
| A/Chicken/Egypt/AR541/2018(H5N8) | 2018-2019 | Africa | Domestic Galliformes | H5N8 |
| A/Northern Goshawk/Tokyo/1301B003/2018(H5N6) | 2018-2019 | Japan | Other wild species | H5N6 |
| A/Northern Goshawk/Tokyo/1301B003T/2018(H5N6) | 2018-2019 | Japan | Other wild species | H5N6 |
| A/duck/Korea/H191/2018(H5N6) | 2018-2019 | Korea | Domestic Anseriformes | H5N6 |
| A/chicken/Kagawa/1C-1/2018(H5N6) | 2018-2019 | Japan | Domestic Galliformes | H5N6 |
| A/chicken/Kagawa/1T-2/2018(H5N6) | 2018-2019 | Japan | Domestic Galliformes | H5N6 |
| A/chicken/Kagawa/2C-1/2018(H5N6) | 2018-2019 | Japan | Domestic Galliformes | H5N6 |
| A/chicken/Kagawa/2C-2/2018(H5N6) | 2018-2019 | Japan | Domestic Galliformes | H5N6 |
| A/chicken/Kagawa/2T-2/2018(H5N6) | 2018-2019 | Japan | Domestic Galliformes | H5N6 |
| A/chicken/Kagawa/3C-1/2018(H5N6) | 2018-2019 | Japan | Domestic Galliformes | H5N6 |
| A/chicken/Kagawa/3C-2/2018(H5N6) | 2018-2019 | Japan | Domestic Galliformes | H5N6 |
| A/chicken/Kagawa/3T-1/2018(H5N6) | 2018-2019 | Japan | Domestic Galliformes | H5N6 |
| A/chicken/Kagawa/3T-2/2018(H5N6) | 2018-2019 | Japan | Domestic Galliformes | H5N6 |
| A/chicken/Kagawa/4C-1/2018(H5N6) | 2018-2019 | Japan | Domestic Galliformes | H5N6 |
| A/chicken/Kagawa/4T-1/2018(H5N6) | 2018-2019 | Japan | Domestic Galliformes | H5N6 |
| A/chicken/Kagawa/5C-1/2018(H5N6) | 2018-2019 | Japan | Domestic Galliformes | H5N6 |
| A/chicken/Kagawa/5C-2/2018(H5N6) | 2018-2019 | Japan | Domestic Galliformes | H5N6 |
| A/chicken/Kagawa/5T-1/2018(H5N6) | 2018-2019 | Japan | Domestic Galliformes | H5N6 |
| A/duck/Korea/H192/2018(H5N6) | 2018-2019 | Korea | Domestic Anseriformes | H5N6 |
| A/duck/Korea/H196/2018(H5N6) | 2018-2019 | Korea | Domestic Anseriformes | H5N6 |
| A/pochard duck/England/AVP 18 003254/2018(H5N6) | 2018-2019 | West Europe | Wild Anseriformes | H5N6 |
| A/chicken/Kagawa/2T-1/2018(H5N6) | 2020-2022 | Japan | Domestic Galliformes | H5N8 |
| A/Chicken/Egypt/AR543/2018(H5N8) | 2018-2019 | Africa | Domestic Galliformes | H5N8 |
| A/chicken/Kagawa/11C-2/2018(H5N6) | 2018-2019 | Japan | Domestic Galliformes | H5N6 |
| A/chicken/Kagawa/11T-1/2018(H5N6) | 2018-2019 | Japan | Domestic Galliformes | H5N6 |
| A/chicken/Kagawa/11T-2/2018(H5N6) | 2018-2019 | Japan | Domestic Galliformes | H5N6 |
| A/chicken/Kagawa/12C-1/2018(H5N6) | 2018-2019 | Japan | Domestic Galliformes | H5N6 |
| A/chicken/Kagawa/12C-2/2018(H5N6) | 2018-2019 | Japan | Domestic Galliformes | H5N6 |
| A/chicken/Kagawa/12T-1/2018(H5N6) | 2018-2019 | Japan | Domestic Galliformes | H5N6 |
| A/chicken/Kagawa/12T-2/2018(H5N6) | 2018-2019 | Japan | Domestic Galliformes | H5N6 |
| A/chicken/Kagawa/13T/2018(H5N6) | 2018-2019 | Japan | Domestic Galliformes | H5N6 |
| A/chicken/Kagawa/14T-1/2018(H5N6) | 2018-2019 | Japan | Domestic Galliformes | H5N6 |
| A/chicken/Kagawa/14T-2/2018(H5N6) | 2018-2019 | Japan | Domestic Galliformes | H5N6 |
| A/chicken/Kagawa/15T/2018(H5N6) | 2018-2019 | Japan | Domestic Galliformes | H5N6 |
| A/Duck/Egypt/AR542/2018(H5N8) | 2018-2019 | Africa | Domestic Anseriformes | H5N8 |
| A/chicken/Kagawa/11C-1/2018(H5N6) | 2020-2022 | Japan | Domestic Galliformes | H5N8 |
| A/chicken/Egypt/F15099/2018(H5N8) | 2018-2019 | Africa | Domestic Galliformes | H5N8 |
| A/Mandarin duck/Korea/K18-3/2018(H5N6) | 2018-2019 | Korea | Wild Anseriformes | H5N6 |
| A/Chicken/Netherlands/18000887-005/2018(H5N6) | 2018-2019 | West Europe | Domestic Galliformes | H5N6 |
| A/Peacock/Netherlands/18000887-006/2018(H5N6) | 2018-2019 | West Europe | Other wild species | H5N6 |
| A/Turkey/Netherlands/18000887-007-010/2018(H5N6) | 2018-2019 | West Europe | Domestic Galliformes | H5N6 |
| A/Great black-backed gull/Netherlands/1/2018(H5N6) | 2018-2019 | West Europe | Charadriiformes | H5N6 |
| A/chicken/Korea/H203/2018(H5N6) | 2018-2019 | Korea | Domestic Galliformes | H5N6 |
| A/mink/Northern China/110/2018(H5N6) | 2018-2019 | China | Mammal | H5N6 |
| A/chicken/Korea/H204/2018(H5N6) | 2018-2019 | Korea | Domestic Galliformes | H5N6 |
| A/Mallard/Republic of Georgia/1/2018(H5N6) | 2018-2019 | West Central Asia | Wild Anseriformes | H5N6 |
| A/gadwall/Iran/18VIR2027-20/2018(H5N6) | 2018-2019 | West Central Asia | Wild Anseriformes | H5N6 |
| A/chicken/Al- Minia/189CA/2018(H5N8) | 2018-2019 | Africa | Domestic Galliformes | H5N8 |
| A/chicken/Giza/1810CA/2018(H5N8) | 2018-2019 | Africa | Domestic Galliformes | H5N8 |
| A/chicken/Luxor/103/2018(H5N8) | 2018-2019 | Africa | Domestic Galliformes | H5N8 |
| A/spotbill duck/Korea/WA159/2018(H5N6) | 2018-2019 | Korea | Wild Anseriformes | H5N6 |
| A/Turkey/Beni Sueif/18296F/2018(H5N8) | 2018-2019 | Africa | Domestic Galliformes | H5N8 |
| A/goose/China/Wuhu02/2018(H5N6) | 2018-2019 | China | Domestic Anseriformes | H5N6 |
| A/chicken/Korea/H214/2018(H5N6) | 2018-2019 | Korea | Domestic Galliformes | H5N6 |
| A/chicken/Egypt/N15173D/2018(H5N8) | 2018-2019 | Africa | Domestic Galliformes | H5N8 |
| A/Eurasian wigeon/Netherlands/1/2018(H5N6) | 2018-2019 | West Europe | Wild Anseriformes | H5N6 |
| A/chicken/Korea/H219/2018(H5N6) | 2018-2019 | Korea | Domestic Galliformes | H5N6 |
| A/white-tailed eagle/Denmark/3073-1w/2018-02-13(H5N6) | 2018-2019 | West Europe | Other wild species | H5N6 |
| A/Chicken/Netherlands/18003041-001-005/2018(H5N6) | 2018-2019 | West Europe | Domestic Galliformes | H5N6 |
| A/chicken/Egypt/CA12/2018(H5N8) | 2018-2019 | Africa | Domestic Galliformes | H5N8 |
| A/chicken/Egypt/FML2/2018(H5N8) | 2018-2019 | Africa | Domestic Galliformes | H5N8 |
| A/Chicken/Netherlands/EMC-1/2018(H5N6) | 2018-2019 | West Europe | Domestic Galliformes | H5N6 |
| A/Chicken/Netherlands/EMC-14/2018(H5N6) | 2018-2019 | West Europe | Domestic Galliformes | H5N6 |
| A/Duck/Egypt/FM36/2018(H5N8) | 2018-2019 | Africa | Domestic Anseriformes | H5N8 |
| A/Duck/Luxor/248/2018(H5N8) | 2018-2019 | Africa | Domestic Anseriformes | H5N8 |
| A/jungle crow/Hyogo/2803A002/2018(H5N6) | 2018-2019 | Japan | Other wild species | H5N6 |
| A/Perigrine falcon/Netherlands/18003274-001/2018(H5N6) | 2018-2019 | West Europe | Other wild species | H5N6 |
| A/Turkey/Luxor/240/2018(H5N8) | 2018-2019 | Africa | Domestic Galliformes | H5N8 |
| A/chicken/Bulgaria/Dobrich/12-1/2018(H5N8) | 2018-2019 | East Europe | Domestic Galliformes | H5N8 |
| A/chicken/Bulgaria/Dobrich/12-2/2018(H5N8) | 2018-2019 | East Europe | Domestic Galliformes | H5N8 |
| A/jungle crow/Hyogo/2803E011/2018(H5N6) | 2018-2019 | Japan | Other wild species | H5N6 |
| A/jungle crow/Hyogo/2803E022/2018(H5N6) | 2018-2019 | Japan | Other wild species | H5N6 |
| A/Jungle crow/Hyogo/2803E023C/2018(H5N6) | 2018-2019 | Japan | Other wild species | H5N6 |
| A/Jungle crow/Hyogo/2803E023T/2018(H5N6) | 2018-2019 | Japan | Other wild species | H5N6 |
| A/Jungle crow/Hyogo/2803E024C/2018(H5N6) | 2018-2019 | Japan | Other wild species | H5N6 |
| A/Jungle crow/Hyogo/2803E024T/2018(H5N6) | 2018-2019 | Japan | Other wild species | H5N6 |
| A/Jungle crow/Hyogo/2803E025C/2018(H5N6) | 2018-2019 | Japan | Other wild species | H5N6 |
| A/Jungle crow/Hyogo/2803E025T/2018(H5N6) | 2018-2019 | Japan | Other wild species | H5N6 |
| A/Jungle crow/Hyogo/2803E026C/2018(H5N6) | 2018-2019 | Japan | Other wild species | H5N6 |
| A/Jungle crow/Hyogo/2803E026T/2018(H5N6) | 2018-2019 | Japan | Other wild species | H5N6 |
| A/Jungle crow/Hyogo/2803E027C/2018(H5N6) | 2018-2019 | Japan | Other wild species | H5N6 |
| A/Jungle crow/Hyogo/2803E027T/2018(H5N6) | 2018-2019 | Japan | Other wild species | H5N6 |
| A/duck/Korea/H229/2018(H5N6) | 2018-2019 | Korea | Domestic Anseriformes | H5N6 |
| A/Domestic Duck/Netherlands/EMC-2/2018(H5N6) | 2018-2019 | West Europe | Domestic Anseriformes | H5N6 |
| A/Domestic Duck/Netherlands/EMC-6/2018(H5N6) | 2018-2019 | West Europe | Domestic Anseriformes | H5N6 |
| A/Duck/Netherlands/18003885-001-005/2018(H5N6) | 2018-2019 | West Europe | Domestic Anseriformes | H5N6 |
| A/Jungle crow/Hyogo/2803E028C/2018(H5N6) | 2018-2019 | Japan | Other wild species | H5N6 |
| A/Jungle crow/Hyogo/2803E028T/2018(H5N6) | 2018-2019 | Japan | Other wild species | H5N6 |
| A/chicken/Egypt/V1410/2018(H5N8) | 2018-2019 | Africa | Domestic Galliformes | H5N8 |
| A/chicken/Korea/H238/2018(H5N6) | 2018-2019 | Korea | Domestic Galliformes | H5N6 |
| A/chicken/Korea/H239/2018(H5N6) | 2018-2019 | Korea | Domestic Galliformes | H5N6 |
| A/chicken/Korea/H241/2018(H5N6) | 2018-2019 | Korea | Domestic Galliformes | H5N6 |
| A/chicken/Germany-SH/AR163-L02542/2018(H5N6) | 2018-2019 | West Europe | Domestic Galliformes | H5N6 |
| A/chicken/Germany-SH/AR164-L02543/2018(H5N6) | 2018-2019 | West Europe | Domestic Galliformes | H5N6 |
| A/duck/Germany-SH/AR165-L02544/2018(H5N6) | 2018-2019 | West Europe | Domestic Anseriformes | H5N6 |
| A/turkey/Germany-SH/AR185-L02549/2018(H5N6) | 2018-2019 | West Europe | Domestic Galliformes | H5N6 |
| A/Chicken/Egypt/AR548/2018(H5N8) | 2018-2019 | Africa | Domestic Galliformes | H5N8 |
| A/Chicken/Egypt/AR549/2018(H5N8) | 2018-2019 | Africa | Domestic Galliformes | H5N8 |
| A/black-headed gull/Denmark/4663-1/2018(H5N6) | 2018-2019 | West Europe | Charadriiformes | H5N6 |
| A/Turkey/Egypt/AR550/2018(H5N8) | 2018-2019 | Africa | Domestic Galliformes | H5N8 |
| A/chicken/Alkharj/910/2018(H5N8) | 2018-2019 | West Central Asia | Domestic Galliformes | H5N8 |
| A/chicken/Albehra/18319F/2018(H5N8) | 2018-2019 | Africa | Domestic Galliformes | H5N8 |
| A/chicken/Al-Minia/188Fao-S/2018(H5N8) | 2018-2019 | Africa | Domestic Galliformes | H5N8 |
| A/Duck/AL-Qaliobia/182Fao-s/2018(H5N8) | 2018-2019 | Africa | Domestic Anseriformes | H5N8 |
| A/Duck/Cairo/187Fao-S/2018(H5N8) | 2018-2019 | Africa | Domestic Anseriformes | H5N8 |
| A/Duck/Cairo/189Fao-S/2018(H5N8) | 2018-2019 | Africa | Domestic Anseriformes | H5N8 |
| A/white stork/Germany-NI/AR251/2018(H5N6) | 2018-2019 | West Europe | Other wild species | H5N6 |
| A/duck/Bulgaria/Yambol/35-1/2018(H5N8) | 2018-2019 | East Europe | Domestic Anseriformes | H5N8 |
| A/Chicken/Egypt/AR552/2018(H5N8) | 2018-2019 | Africa | Domestic Galliformes | H5N8 |
| A/Chicken/Egypt/AR553/2018(H5N8) | 2018-2019 | Africa | Domestic Galliformes | H5N8 |
| A/quail/South Africa/AI5930/2018(H5N8) | 2018-2019 | Africa | Domestic Galliformes | H5N8 |
| A/duck/Bulgaria/Plovdiv/74-1/2018(H5N8) | 2018-2019 | East Europe | Domestic Anseriformes | H5N8 |
| A/duck/Bulgaria/Plovdiv/74-2/2018(H5N2) | 2018-2019 | East Europe | Domestic Anseriformes | H5N2 |
| A/duck/Bulgaria/Plovdiv/76-1/2018(H5N8) | 2018-2019 | East Europe | Domestic Anseriformes | H5N8 |
| A/duck/Bulgaria/Plovdiv/76-2/2018(H5N8) | 2018-2019 | East Europe | Domestic Anseriformes | H5N8 |
| A/Chicken/Egypt/AR556/2018(H5N8) | 2018-2019 | Africa | Domestic Galliformes | H5N8 |
| A/Chicken/Egypt/AR557/2018(H5N8) | 2018-2019 | Africa | Domestic Galliformes | H5N8 |
| A/buzzard/Germany-NRW/AR279/2018(H5N6) | 2018-2019 | West Europe | Other wild species | H5N6 |
| A/Chicken/Egypt/AR591/2018(H5N8) | 2018-2019 | Africa | Domestic Galliformes | H5N8 |
| A/Chicken/Egypt/AR558/2018(H5N8) | 2018-2019 | Africa | Domestic Galliformes | H5N8 |
| A/Chicken/Egypt/AR559/2018(H5N8) | 2018-2019 | Africa | Domestic Galliformes | H5N8 |
| A/Duck/Egypt/AR560/2018(H5N8) | 2018-2019 | Africa | Domestic Anseriformes | H5N8 |
| A/Duck/Egypt/V1778/2018(H5N8) | 2018-2019 | Africa | Domestic Anseriformes | H5N8 |
| A/Duck/Giza/1814/2018(H5N8) | 2018-2019 | Africa | Domestic Anseriformes | H5N8 |
| A/chicken/South Africa/499723/2018(H5N8) | 2018-2019 | Africa | Domestic Galliformes | H5N8 |
| A/chicken/Kursk/284/2018(H5N8) | 2018-2019 | East Europe | Domestic Galliformes | H5N8 |
| A/chicken/Bulgaria/Dobrich/115/2018(H5N8) | 2018-2019 | East Europe | Domestic Galliformes | H5N8 |
| A/chicken/Kursk/526/2018(H5N8) | 2018-2019 | East Europe | Domestic Galliformes | H5N8 |
| A/chicken/Penza/300/2018(H5N8) | 2018-2019 | East Europe | Domestic Galliformes | H5N8 |
| A/chicken/Penza/301/2018(H5N8) | 2018-2019 | East Europe | Domestic Galliformes | H5N8 |
| A/chicken/Penza/605/2018(H5N8) | 2018-2019 | East Europe | Domestic Galliformes | H5N8 |
| A/chicken/Penza/607/2018(H5N8) | 2018-2019 | East Europe | Domestic Galliformes | H5N8 |
| A/chicken/Kursk/757/2018(H5N8) | 2018-2019 | East Europe | Domestic Galliformes | H5N8 |
| A/chicken/Orel/533/2018(H5N8) | 2018-2019 | Russian Federation Siberia | Domestic Galliformes | H5N8 |
| A/chicken/Samara/446/2018(H5N8) | 2018-2019 | Russian Federation Siberia | Domestic Galliformes | H5N8 |
| A/chicken/Samara/447/2018(H5N8) | 2018-2019 | Russian Federation Siberia | Domestic Galliformes | H5N8 |
| A/duck/Samara/452/2018(H5N8) | 2018-2019 | Russian Federation Siberia | Domestic Anseriformes | H5N8 |
| A/goose/Samara/455/2018(H5N8) | 2018-2019 | Russian Federation Siberia | Domestic Anseriformes | H5N8 |
| A/goose/Samara/459/2018(H5N8) | 2018-2019 | Russian Federation Siberia | Domestic Anseriformes | H5N8 |
| A/Chicken/Egypt/AR562/2018(H5N8) | 2018-2019 | Africa | Domestic Galliformes | H5N8 |
| A/chicken/Kursk/760/2018(H5N8) | 2018-2019 | East Europe | Domestic Galliformes | H5N8 |
| A/chicken/Kursk/762/2018(H5N8) | 2018-2019 | East Europe | Domestic Galliformes | H5N8 |
| A/chicken/Samara/679/2018(H5N8) | 2018-2019 | Russian Federation Siberia | Domestic Galliformes | H5N8 |
| A/goose/Samara/673/2018(H5N8) | 2018-2019 | Russian Federation Siberia | Domestic Anseriformes | H5N8 |
| A/goose/Samara/675/2018(H5N8) | 2018-2019 | Russian Federation Siberia | Domestic Anseriformes | H5N8 |
| A/chicken/Bulgaria/Dobrich/163-1/2018(H5N8) | 2018-2019 | East Europe | Domestic Galliformes | H5N8 |
| A/chicken/Bulgaria/Dobrich/163-2/2018(H5N8) | 2018-2019 | East Europe | Domestic Galliformes | H5N8 |
| A/chicken/Rostov-on-Don/766/2018(H5N8) | 2018-2019 | East Europe | Domestic Galliformes | H5N8 |
| A/chicken/Cheboksary/805/2018(H5N8) | 2018-2019 | East Europe | Domestic Galliformes | H5N8 |
| A/chicken/Cheboksary/806/2018(H5N8) | 2018-2019 | East Europe | Domestic Galliformes | H5N8 |
| A/turkey/Rostov-on-Don/817/2018(H5N8) | 2018-2019 | East Europe | Domestic Galliformes | H5N8 |
| A/chicken/Cheboksary/849/2018(H5N8) | 2018-2019 | East Europe | Domestic Galliformes | H5N8 |
| A/chicken/Cheboksary/850/2018(H5N8) | 2018-2019 | East Europe | Domestic Galliformes | H5N8 |
| A/chicken/Cheboksary/851/2018(H5N8) | 2018-2019 | East Europe | Domestic Galliformes | H5N8 |
| A/chicken/Cheboksary/853/2018(H5N8) | 2018-2019 | East Europe | Domestic Galliformes | H5N8 |
| A/chicken/Cheboksary/854/2018(H5N8) | 2018-2019 | East Europe | Domestic Galliformes | H5N8 |
| A/chicken/Egypt/FL6/2018(H5N8) | 2018-2019 | Africa | Domestic Galliformes | H5N8 |
| A/chicken/Tatarstan/7/2018(H5N8) | 2018-2019 | Russian Federation Siberia | Domestic Galliformes | H5N8 |
| A/chicken/Mari El/870/2018(H5N8) | 2018-2019 | Russian Federation Siberia | Domestic Galliformes | H5N8 |
| A/Mallard/Netherlands/18012508-017/2018(H5N6) | 2018-2019 | West Europe | Wild Anseriformes | H5N6 |
| A/mallard/Denmark/12106-1/2018(H5N6) | 2018-2019 | West Europe | Wild Anseriformes | H5N6 |
| A/mallard/Denmark/12106-2/2018(H5N6) | 2018-2019 | West Europe | Wild Anseriformes | H5N6 |
| A/pheasant/Denmark/12106-03/2018(H5N6) | 2018-2019 | West Europe | Other wild species | H5N6 |
| A/domestic duck/Germany-MV/AR613-L02727/2018(H5N6) | 2018-2019 | West Europe | Domestic Anseriformes | H5N6 |
| A/chicken/Bulgaria/Plovdiv/224-1/2018(H5N8) | 2018-2019 | East Europe | Domestic Galliformes | H5N8 |
| A/chicken/Bulgaria/Plovdiv/224-2/2018(H5N8) | 2018-2019 | East Europe | Domestic Galliformes | H5N8 |
| A/chicken/Bulgaria/Plovdiv/224-3/2018(H5N8) | 2018-2019 | East Europe | Domestic Galliformes | H5N8 |
| A/turkey/Rostov-on-Don/1117/2018(H5N8) | 2016-2017 | East Europe | Domestic Galliformes | H5N8 |
| A/Turkey/Central Russia/5/2018(H5N8) | 2018-2019 | Russian Federation Siberia | Domestic Galliformes | H5N8 |
| A/chicken/Bulgaria/Haskovo/286/2018(H5N8) | 2018-2019 | East Europe | Domestic Galliformes | H5N8 |
| A/chicken/Bulgaria/Plovdiv/295/2018(H5N8) | 2018-2019 | East Europe | Domestic Galliformes | H5N8 |
| A/chicken/Pakistan/531/2018(H5N8) | 2018-2019 | West Central Asia | Domestic Galliformes | H5N8 |
| A/chicken/Bulgaria/Plovdiv/333/2018(H5N8) | 2018-2019 | East Europe | Domestic Galliformes | H5N8 |
| A/turkey/Bulgaria/Haskovo/336/2018(H5N8) | 2018-2019 | East Europe | Domestic Galliformes | H5N8 |
| A/chicken/Voronezh/1488/2018(H5N8) | 2018-2019 | East Europe | Domestic Galliformes | H5N8 |
| A/chicken/Voronezh/1504/2018(H5N8) | 2018-2019 | East Europe | Domestic Galliformes | H5N8 |
| A/chicken/Voronezh/1513/2018(H5N8) | 2018-2019 | East Europe | Domestic Galliformes | H5N8 |
| A/chiken/Voronezh/1491/2018(H5N8) | 2018-2019 | East Europe | Domestic Galliformes | H5N8 |
| A/chicken/Bulgaria/Vidin-Kosovo/550/2018(H5N8) | 2018-2019 | East Europe | Domestic Galliformes | H5N8 |
| A/chicken/Pakistan/1000/2018(H5N8) | 2018-2019 | West Central Asia | Domestic Galliformes | H5N8 |
| A/quail/Pakistan/998/2018(H5N8) | 2018-2019 | West Central Asia | Domestic Galliformes | H5N8 |
| A/Duck/Egypt/SMG6/2018(H5N8) | 2018-2019 | Africa | Domestic Anseriformes | H5N8 |
| A/duck/Egypt/VG1099/2018(H5N2) | 2018-2019 | Africa | Domestic Anseriformes | H5N2 |
| A/chicken/Egypt/AL1/2019(H5N8) | 2018-2019 | Africa | Domestic Galliformes | H5N8 |
| A/Duck/Egypt/F131/2019(H5N8) | 2018-2019 | Africa | Domestic Anseriformes | H5N8 |
| A/Duck/Egypt/AI20251/2019(H5N8) | 2018-2019 | Africa | Domestic Anseriformes | H5N8 |
| A/Turkey/Egypt/F111/2019(H5N8) | 2018-2019 | Africa | Domestic Galliformes | H5N8 |
| A/Duck/Egypt/SMG4/2019(H5N8) | 2018-2019 | Africa | Domestic Anseriformes | H5N8 |
| A/Duck/Egypt/AI20252/2019 (H5N8) | 2018-2019 | Africa | Domestic Anseriformes | H5N8 |
| A/Chicken/Egypt/Al00994/2019(H5N2) | 2018-2019 | Africa | Domestic Galliformes | H5N2 |
| A/Duck/Egypt/SMG5/2019(H5N8) | 2018-2019 | Africa | Domestic Anseriformes | H5N8 |
| A/chicken/Egypt/Q16710A/2019(H5N8) | 2018-2019 | Africa | Domestic Galliformes | H5N8 |
| A/chicken/Egypt/Q16710B/2019(H5N8) | 2018-2019 | Africa | Domestic Galliformes | H5N8 |
| A/chicken/Egypt/Q16710C/2019(H5N8) | 2018-2019 | Africa | Domestic Galliformes | H5N8 |
| A/chicken/Egypt/Q16711A/2019(H5N8) | 2018-2019 | Africa | Domestic Galliformes | H5N8 |
| A/chicken/Egypt/Q16711B/2019(H5N8) | 2018-2019 | Africa | Domestic Galliformes | H5N8 |
| A/chicken/Egypt/Q16711C/2019(H5N8) | 2018-2019 | Africa | Domestic Galliformes | H5N8 |
| A/chicken/Egypt/Q16712A/2019(H5N8) | 2018-2019 | Africa | Domestic Galliformes | H5N8 |
| A/duck/Egypt/Q16716A/2019(H5N8) | 2018-2019 | Africa | Domestic Anseriformes | H5N8 |
| A/chicken/Egypt/V1748/2019(H5N8) | 2018-2019 | Africa | Domestic Galliformes | H5N8 |
| A/chicken/Egypt/N16730/2019(H5N8) | 2018-2019 | Africa | Domestic Galliformes | H5N8 |
| A/chicken/Egypt/N16732/2019(H5N8) | 2018-2019 | Africa | Domestic Galliformes | H5N8 |
| A/Duck/Egypt/AI20263/2019 (H5N8) | 2018-2019 | Africa | Domestic Anseriformes | H5N8 |
| A/duck/Egypt/N16717/2019(H5N8) | 2018-2019 | Africa | Domestic Anseriformes | H5N8 |
| A/duck/Egypt/N16719/2019(H5N8) | 2018-2019 | Africa | Domestic Anseriformes | H5N8 |
| A/duck/Egypt/N16720/2019(H5N8) | 2018-2019 | Africa | Domestic Anseriformes | H5N8 |
| A/duck/Egypt/N16721/2019(H5N8) | 2018-2019 | Africa | Domestic Anseriformes | H5N8 |
| A/duck/Egypt/N16722/2019(H5N8) | 2018-2019 | Africa | Domestic Anseriformes | H5N8 |
| A/duck/Egypt/A16793/2019(H5N8) | 2018-2019 | Africa | Domestic Anseriformes | H5N8 |
| A/Duck/Egypt/AI20266/2019 (H5N8) | 2018-2019 | Africa | Domestic Anseriformes | H5N8 |
| A/Duck/Luxor/294/2019(H5N8) | 2018-2019 | Africa | Domestic Anseriformes | H5N8 |
| A/Duck/Luxor/297/2019(H5N8) | 2018-2019 | Africa | Domestic Anseriformes | H5N8 |
| A/chicken/Egypt/Q16807B/2019(H5N8) | 2018-2019 | Africa | Domestic Galliformes | H5N8 |
| A/chicken/Egypt/Q16807C/2019(H5N8) | 2018-2019 | Africa | Domestic Galliformes | H5N8 |
| A/chicken/Egypt/Q16807E/2019(H5N8) | 2018-2019 | Africa | Domestic Galliformes | H5N8 |
| A/chicken/Egypt/Q16808B/2019(H5N8) | 2018-2019 | Africa | Domestic Galliformes | H5N8 |
| A/chicken/Egypt/S30/2019(H5N8) | 2018-2019 | Africa | Domestic Galliformes | H5N8 |
| A/Chicken/Egypt/AI20268/2019 (H5N8) | 2018-2019 | Africa | Domestic Galliformes | H5N8 |
| A/Chicken/Egypt/AI20284/2019 (H5N8) | 2018-2019 | Africa | Domestic Galliformes | H5N8 |
| A/duck/Bulgaria/130-1 19VIR3314-1/2019(H5N8) | 2018-2019 | East Europe | Domestic Anseriformes | H5N8 |
| A/Duck/Egypt/A3/2019(H5N8) | 2018-2019 | Africa | Domestic Anseriformes | H5N8 |
| A/chicken/Bulgaria/136 19VIR3315-1/2019(H5N8) | 2018-2019 | East Europe | Domestic Galliformes | H5N8 |
| A/Turkey/Egypt/AI20285/2019(H5N8) | 2018-2019 | Africa | Domestic Galliformes | H5N8 |
| A/chicken/Bulgaria/143 19VIR3315-2/2019(H5N8) | 2018-2019 | East Europe | Domestic Galliformes | H5N8 |
| A/chicken/Bulgaria/148-01 19VIR3315-3/2019(H5N8) | 2018-2019 | East Europe | Domestic Galliformes | H5N8 |
| A/chicken/Bulgaria/150-01 19VIR3315-5/2019(H5N8) | 2018-2019 | East Europe | Domestic Galliformes | H5N8 |
| A/Chicken/Egypt/AI20286/2019(H5N8) | 2018-2019 | Africa | Domestic Galliformes | H5N8 |
| A/chicken/Luxor/313/2019(H5N8) | 2018-2019 | Africa | Domestic Galliformes | H5N8 |
| A/Teal/Dakahlia/VRLCU/2019(H5N8) | 2018-2019 | Africa | Wild Anseriformes | H5N8 |
| A/chicken/Egypt/F17229A/2019(H5N8) | 2018-2019 | Africa | Domestic Galliformes | H5N8 |
| A/chicken/Egypt/F17229B/2019(H5N8) | 2018-2019 | Africa | Domestic Galliformes | H5N8 |
| A/chicken/Egypt/F17230A/2019(H5N8) | 2018-2019 | Africa | Domestic Galliformes | H5N8 |
| A/chicken/Egypt/F17230B/2019(H5N8) | 2018-2019 | Africa | Domestic Galliformes | H5N8 |
| A/chicken/Egypt/F17230C/2019(H5N8) | 2018-2019 | Africa | Domestic Galliformes | H5N8 |
| A/chicken/Egypt/F17230D/2019(H5N8) | 2018-2019 | Africa | Domestic Galliformes | H5N8 |
| A/duck/Nigeria/SK28T 19VIR8424-2/2019(H5N6) | 2018-2019 | Africa | Domestic Anseriformes | H5N6 |
| A/guinea fowl/Nigeria/OG-GF11T 19VIR8424-7/2019 H5N8(H5N8) | 2018-2019 | Africa | Other wild species | H5N8 |
| A/turkey/Poland/23/2019(H5N8) | 2018-2019 | East Europe | Domestic Galliformes | H5N8 |
| A/laying hen/Poland/002/2020(H5N8) | 2020-2022 | East Europe | Domestic Galliformes | H5N8 |
| A/chicken/Poland/003/2020(H5N8) | 2020-2022 | East Europe | Domestic Galliformes | H5N8 |
| A/chicken/Poland/004/2020(H5N8) | 2020-2022 | East Europe | Domestic Galliformes | H5N8 |
| A/hawk/Poland/003/2020(H5N8) | 2020-2022 | East Europe | Other wild species | H5N8 |
| A/turkey/Hungary/1020 20VIR749-1/2020(H5N8) | 2020-2022 | East Europe | Domestic Galliformes | H5N8 |
| A/turkey/Poland/027/2020(H5N8) | 2020-2022 | East Europe | Domestic Galliformes | H5N8 |
| A/Larus argentatus/Belgium/568/2021(H5N8) | 2020-2022 | West Europe | Charadriiformes | H5N8 |
| A/domestic goose/Poland/028/2020(H5N8) | 2020-2022 | East Europe | Domestic Anseriformes | H5N8 |
| A/Duck/Egypt/BEH2/2020(H5N8) | 2020-2022 | Africa | Domestic Anseriformes | H5N8 |
| A/white-fronted goose/Germany-BB/AI00018/2020(H5N8) | 2020-2022 | West Europe | Wild Anseriformes | H5N8 |
| A/chicken/Czech Republic/1175-1/2020(H5N8) | 2020-2022 | East Europe | Domestic Galliformes | H5N8 |
| A/chicken/Poland/054/2020(H5N8) | 2020-2022 | East Europe | Domestic Galliformes | H5N8 |
| A/turkey/Poland/079/2020(H5N8) | 2020-2022 | East Europe | Domestic Galliformes | H5N8 |
| A/laying hen/Poland/095/2020(H5N8) | 2020-2022 | East Europe | Domestic Galliformes | H5N8 |
| A/turkey/Poland/096/2020(H5N8) | 2020-2022 | East Europe | Domestic Galliformes | H5N8 |
| A/chicken/Germany-BW/AI00049/2020(H5N8) | 2020-2022 | West Europe | Domestic Galliformes | H5N8 |
| A/turkey/Poland/182/2020(H5N8) | 2020-2022 | East Europe | Domestic Galliformes | H5N8 |
| A/chicken/Egypt/GIZ4/2020(H5N8) | 2020-2022 | Africa | Domestic Galliformes | H5N8 |
| A/turkey/Czech Republic/3071/2020(H5N8) | 2020-2022 | East Europe | Domestic Galliformes | H5N8 |
| A/domestic duck/Poland/219/2020(H5N8) | 2020-2022 | East Europe | Domestic Anseriformes | H5N8 |
| A/domestic duck/Poland/221/2020(H5N8) | 2020-2022 | East Europe | Domestic Anseriformes | H5N8 |
| A/domestic duck/Poland/222/2020(H5N8) | 2020-2022 | East Europe | Domestic Anseriformes | H5N8 |
| A/domestic duck/Poland/223/2020(H5N8) | 2020-2022 | East Europe | Domestic Anseriformes | H5N8 |
| A/duck/Bulgaria/78-4t 20VIR1416-3/2020(H5N8) | 2020-2022 | East Europe | Domestic Anseriformes | H5N8 |
| A/domestic duck/Poland/229/2020(H5N8) | 2020-2022 | East Europe | Domestic Anseriformes | H5N8 |
| A/domestic duck/Poland/230/2020(H5N8) | 2020-2022 | East Europe | Domestic Anseriformes | H5N8 |
| A/domestic duck/Poland/237/2020(H5N8) | 2020-2022 | East Europe | Domestic Anseriformes | H5N8 |
| A/domestic duck/Poland/263/2020(H5N8) | 2020-2022 | East Europe | Domestic Anseriformes | H5N8 |
| A/domestic duck/Poland/271/2020(H5N8) | 2020-2022 | East Europe | Domestic Anseriformes | H5N8 |
| A/domestic goose/Poland/274/2020(H5N8) | 2020-2022 | East Europe | Domestic Anseriformes | H5N8 |
| A/domestic duck/Poland/285/2020(H5N8) | 2020-2022 | East Europe | Domestic Anseriformes | H5N8 |
| A/laying hen/Poland/312/2020(H5N8) | 2020-2022 | East Europe | Domestic Galliformes | H5N8 |
| A/chicken/Germany-SN/AI00276/2020(H5N8) | 2020-2022 | West Europe | Domestic Galliformes | H5N8 |
| A/buzzard/Germany-SN/AI00285/2020(H5N8) | 2020-2022 | West Europe | Other wild species | H5N8 |
| A/turkey/Germany-NI/AI00334/2020(H5N8) | 2020-2022 | West Europe | Domestic Galliformes | H5N8 |
| A/turkey/Poland/366/2020(H5N8) | 2020-2022 | East Europe | Domestic Galliformes | H5N8 |
| A/Duck/Hungary/14788/2020 (H5N8) | 2020-2022 | East Europe | Domestic Anseriformes | H5N8 |
| A/Goose/Hungary/15267/2020(H5N8) | 2020-2022 | East Europe | Domestic Anseriformes | H5N8 |
| A/steamer duck/Germany-SN/AI00346/2020(H5N8) | 2020-2022 | West Europe | Wild Anseriformes | H5N8 |
| A/turkey/Germany-ST/AI00352/2020(H5N8) | 2020-2022 | West Europe | Domestic Galliformes | H5N8 |
| A/Mallard duck/Hungary/17319/2020 (H5N8) | 2020-2022 | East Europe | Wild Anseriformes | H5N8 |
| A/Duck/Hungary/17806/2020 (H5N8) | 2020-2022 | East Europe | Domestic Anseriformes | H5N8 |
| A/Duck/Hungary/17957/2020 (H5N8) | 2020-2022 | East Europe | Domestic Anseriformes | H5N8 |
| A/Goose/Hungary/18325/2020 (H5N8) | 2020-2022 | East Europe | Domestic Anseriformes | H5N8 |
| A/Duck/Hungary/18358/2020 (H5N8) | 2020-2022 | East Europe | Domestic Anseriformes | H5N8 |
| A/Chicken/Hungary/18466/2020 (H5N8) | 2020-2022 | East Europe | Domestic Galliformes | H5N8 |
| A/Chicken/Hungary/18467/2020 (H5N8) | 2020-2022 | East Europe | Domestic Galliformes | H5N8 |
| A/Duck/Hungary/18414/2020 (H5N8) | 2020-2022 | East Europe | Domestic Anseriformes | H5N8 |
| A/Duck/Hungary/18444/2020 (H5N8) | 2020-2022 | East Europe | Domestic Anseriformes | H5N8 |
| A/Goose/Hungary/18406/2020 (H5N8) | 2020-2022 | East Europe | Domestic Anseriformes | H5N8 |
| A/Mallard duck/Hungary/18410/2020 (H5N8) | 2020-2022 | East Europe | Wild Anseriformes | H5N8 |
| A/Goose/Hungary/19118/2020 (H5N8) | 2020-2022 | East Europe | Domestic Anseriformes | H5N8 |
| A/Goose/Hungary/19128/2020 (H5N8) | 2020-2022 | East Europe | Domestic Anseriformes | H5N8 |
| A/Turkey/Hungary/19338/2020 (H5N8) | 2020-2022 | East Europe | Domestic Galliformes | H5N8 |
| A/Turkey/Hungary/19394/2020 (H5N8) | 2020-2022 | East Europe | Domestic Galliformes | H5N8 |
| A/Pheasant/Hungary/18731/2020 (H5N8) | 2020-2022 | East Europe | Other wild species | H5N8 |
| A/Chicken/Hungary/19776/2020 (H5N8) | 2020-2022 | East Europe | Domestic Galliformes | H5N8 |
| A/Goose/Hungary/19953/2020(H5N8) | 2020-2022 | East Europe | Domestic Anseriformes | H5N8 |
| A/Goose/Hungary/19959/2020 (H5N8) | 2020-2022 | East Europe | Domestic Anseriformes | H5N8 |
| A/Chicken/Hungary/20227/2020 (H5N8) | 2020-2022 | East Europe | Domestic Galliformes | H5N8 |
| A/Chicken/Hungary/21379/2020(H5N8) | 2020-2022 | East Europe | Domestic Galliformes | H5N8 |
| A/Goose/Hungary/21737/2020 (H5N8) | 2020-2022 | East Europe | Domestic Anseriformes | H5N8 |
| A/Turkey/Hungary/21753/2020 (H5N8) | 2020-2022 | East Europe | Domestic Galliformes | H5N8 |
| A/Goose/Hungary/22493/2020 (H5N8) | 2020-2022 | East Europe | Domestic Anseriformes | H5N8 |
| A/Turkey/Hungary/22494/2020 (H5N8) | 2020-2022 | East Europe | Domestic Galliformes | H5N8 |
| A/Goose/Hungary/24021/2020 (H5N8) | 2020-2022 | East Europe | Domestic Anseriformes | H5N8 |
| A/chicken/Iraq/1/2020(H5N8) | 2020-2022 | West Central Asia | Domestic Galliformes | H5N8 |
| A/Chicken/Hungary/24596/2020 (H5N8) | 2020-2022 | East Europe | Domestic Galliformes | H5N8 |
| A/chicken/Bulgaria/380-1 20VIR3542-1/2020(H5N8) | 2020-2022 | East Europe | Domestic Galliformes | H5N8 |
| A/Chicken/Hungary/29723/2020(H5N8) | 2020-2022 | East Europe | Domestic Galliformes | H5N8 |
| A/duck/Chelyabinsk/1207-1/2020(H5N8) | 2020-2022 | Russian Federation Siberia | Domestic Anseriformes | H5N8 |
| A/chicken/Chelyabinsk/401/2020(H5N8) | 2020-2022 | Russian Federation Siberia | Domestic Galliformes | H5N8 |
| A/chicken/Chelyabinsk/402/2020(H5N8) | 2020-2022 | Russian Federation Siberia | Domestic Galliformes | H5N8 |
| A/chicken/Chelyabinsk/403/2020(H5N8) | 2020-2022 | Russian Federation Siberia | Domestic Galliformes | H5N8 |
| A/chicken/Chelyabinsk/404/2020(H5N8) | 2020-2022 | Russian Federation Siberia | Domestic Galliformes | H5N8 |
| A/duck/Omsk/0004/2020(H5N8) | 2020-2022 | Russian Federation Siberia | Domestic Anseriformes | H5N8 |
| A/goose/Omsk/0002/2020(H5N8) | 2020-2022 | Russian Federation Siberia | Domestic Anseriformes | H5N8 |
| A/turkey/Omsk/0001/2020(H5N8) | 2020-2022 | Russian Federation Siberia | Domestic Galliformes | H5N8 |
| A/turkey/Omsk/0003/2020(H5N8) | 2020-2022 | Russian Federation Siberia | Domestic Galliformes | H5N8 |
| A/chicken/Omsk/0073/2020(H5N8) | 2020-2022 | Russian Federation Siberia | Domestic Galliformes | H5N8 |
| A/chicken/Omsk/0112/2020(H5N8) | 2020-2022 | Russian Federation Siberia | Domestic Galliformes | H5N8 |
| A/chicken/Omsk/0118/2020(H5N8) | 2020-2022 | Russian Federation Siberia | Domestic Galliformes | H5N8 |
| A/chicken/Omsk/0119/2020(H5N8) | 2020-2022 | Russian Federation Siberia | Domestic Galliformes | H5N8 |
| A/duck/Omsk/0075/2020(H5N8) | 2020-2022 | Russian Federation Siberia | Domestic Anseriformes | H5N8 |
| A/duck/Omsk/0076/2020(H5N8) | 2020-2022 | Russian Federation Siberia | Domestic Anseriformes | H5N8 |
| A/duck/Omsk/0077/2020(H5N8) | 2020-2022 | Russian Federation Siberia | Domestic Anseriformes | H5N8 |
| A/duck/Russian Federation/Omsk/1328-2/2020(H5N8) | 2020-2022 | Russian Federation Siberia | Domestic Anseriformes | H5N8 |
| A/goose/Omsk/0071/2020(H5N8) | 2020-2022 | Russian Federation Siberia | Domestic Anseriformes | H5N8 |
| A/goose/Omsk/0074/2020(H5N8) | 2020-2022 | Russian Federation Siberia | Domestic Anseriformes | H5N8 |
| A/goose/Omsk/0111/2020(H5N8) | 2020-2022 | Russian Federation Siberia | Domestic Anseriformes | H5N8 |
| A/goose/Omsk/011101/2020(H5N8) | 2020-2022 | Russian Federation Siberia | Domestic Anseriformes | H5N8 |
| A/goose/Omsk/0113/2020(H5N8) | 2020-2022 | Russian Federation Siberia | Domestic Anseriformes | H5N8 |
| A/goose/Omsk/0114/2020(H5N8) | 2020-2022 | Russian Federation Siberia | Domestic Anseriformes | H5N8 |
| A/goose/Omsk/0115/2020(H5N8) | 2020-2022 | Russian Federation Siberia | Domestic Anseriformes | H5N8 |
| A/goose/Omsk/01161/2020(H5N8) | 2020-2022 | Russian Federation Siberia | Domestic Anseriformes | H5N8 |
| A/goose/Omsk/01171/2020(H5N8) | 2020-2022 | Russian Federation Siberia | Domestic Anseriformes | H5N8 |
| A/wild duck/Omsk/01111/2020(H5N8) | 2020-2022 | Russian Federation Siberia | Wild Anseriformes | H5N8 |
| A/goose/Russian Federation/Kurgan/1345-25/2020 (H5N8) | 2020-2022 | Russian Federation Siberia | Domestic Anseriformes | H5N8 |
| A/chicken/Kurgan/1001/2020(H5N8) | 2020-2022 | Russian Federation Siberia | Domestic Galliformes | H5N8 |
| A/chicken/Kurgan/1003/2020(H5N8) | 2020-2022 | Russian Federation Siberia | Domestic Galliformes | H5N8 |
| A/chicken/Kurgan/1004/2020(H5N8) | 2020-2022 | Russian Federation Siberia | Domestic Galliformes | H5N8 |
| A/chicken/Kurgan/1005/2020(H5N8) | 2020-2022 | Russian Federation Siberia | Domestic Galliformes | H5N8 |
| A/chicken/Kurgan/1010/2020(H5N8) | 2020-2022 | Russian Federation Siberia | Domestic Galliformes | H5N8 |
| A/goose/Kurgan/01/2020(H5N8) | 2020-2022 | Russian Federation Siberia | Domestic Anseriformes | H5N8 |
| A/common teal/Chany Lake/213/2020(H5N2) | 2020-2022 | Russian Federation Siberia | Wild Anseriformes | H5N2 |
| A/goose/Russia Omsk region/55-1/2020(H5N8) | 2020-2022 | Russian Federation Siberia | Domestic Anseriformes | H5N8 |
| A/mallard/Novosibirsk region/3509k/2020(H5N8) | 2020-2022 | Russian Federation Siberia | Wild Anseriformes | H5N8 |
| A/chicken/Kazakhstan/220-B-2-H5N8-4/2020(H5N8) | 2020-2022 | West Central Asia | Domestic Galliformes | H5N8 |
| A/goose/Kazakhstan/4-190-20-B-H5N8-1/2020(H5N8) | 2020-2022 | West Central Asia | Domestic Anseriformes | H5N8 |
| A/chicken/Omsk/30007/2020(H5N8) | 2020-2022 | Russian Federation Siberia | Domestic Galliformes | H5N8 |
| A/goose/Omsk/30001/2020(H5N8) | 2020-2022 | Russian Federation Siberia | Domestic Anseriformes | H5N8 |
| A/goose/Omsk/30003/2020(H5N8) | 2020-2022 | Russian Federation Siberia | Domestic Anseriformes | H5N8 |
| A/goose/Omsk/30004/2020(H5N8) | 2020-2022 | Russian Federation Siberia | Domestic Anseriformes | H5N8 |
| A/goose/Omsk/30006/2020(H5N8) | 2020-2022 | Russian Federation Siberia | Domestic Anseriformes | H5N8 |
| A/goose/Omsk/30009/2020(H5N8) | 2020-2022 | Russian Federation Siberia | Domestic Anseriformes | H5N8 |
| A/goose/Omsk/30010/2020(H5N8) | 2020-2022 | Russian Federation Siberia | Domestic Anseriformes | H5N8 |
| A/chicken/Chelyabinsk/201/2020(H5N8) | 2020-2022 | Russian Federation Siberia | Domestic Galliformes | H5N8 |
| A/swan/Tumen/1479-2/2020(H5N8) | 2020-2022 | Russian Federation Siberia | Wild Anseriformes | H5N8 |
| A/chicken/Saratov/29801/2020(H5N8) | 2020-2022 | East Europe | Domestic Galliformes | H5N8 |
| A/crow/Kazakhstan/15-20-B-Talg-4/2020(H5N8) | 2020-2022 | West Central Asia | Other wild species | H5N8 |
| A/duck/Kazakhstan/12-20-B-Talg-11/2020(H5N8) | 2020-2022 | West Central Asia | Domestic Anseriformes | H5N8 |
| A/duck/Saratov/29804/2020(H5N8) | 2020-2022 | East Europe | Domestic Anseriformes | H5N8 |
| A/goose/Russia Novosibirsk region/1-12/2020(H5N8) | 2020-2022 | Russian Federation Siberia | Domestic Anseriformes | H5N8 |
| A/pigeon/Kazakhstan/15-20-B-Talg-5/2020(H5N8) | 2020-2022 | West Central Asia | Domestic Galliformes | H5N8 |
| A/chicken/Kazakhstan/Kn-3/2020(H5N8) | 2020-2022 | West Central Asia | Domestic Galliformes | H5N8 |
| A/chicken/Kazakhstan/Kn-6/2020(H5N8) | 2020-2022 | West Central Asia | Domestic Galliformes | H5N8 |
| A/duck/Russian Federation/Saratov/1578-2/2020 (H5N8) | 2020-2022 | East Europe | Domestic Anseriformes | H5N8 |
| A/chicken/Kazakhstan/12-20-B-Talg-45/2020(H5N8) | 2020-2022 | West Central Asia | Domestic Galliformes | H5N8 |
| A/domestic goose/Kazakhstan/1-242 2-20-B/2020(H5N8) | 2020-2022 | West Central Asia | Domestic Anseriformes | H5N8 |
| A/chicken/Russia Novosibirsk region/3-1/2020(H5N8) | 2020-2022 | Russian Federation Siberia | Domestic Galliformes | H5N8 |
| A/chicken/Russia Novosibirsk region/3-15/2020(H5N8) | 2020-2022 | Russian Federation Siberia | Domestic Galliformes | H5N8 |
| A/chicken/Russia Novosibirsk region/3-29/2020(H5N8) | 2020-2022 | Russian Federation Siberia | Domestic Galliformes | H5N8 |
| A/domestic goose/Kazakhstan/1-248 2-20-B/2020(H5N8) | 2020-2022 | West Central Asia | Domestic Anseriformes | H5N8 |
| A/swan/Kazakhstan/9-20-B-Talg-39/2020(H5N8) | 2020-2022 | West Central Asia | Wild Anseriformes | H5N8 |
| A/chicken/Russia Novosibirsk region/1910-1/2020(H5N8) | 2020-2022 | Russian Federation Siberia | Domestic Galliformes | H5N8 |
| A/chicken/Russia Novosibirsk region/1910-2/2020(H5N8) | 2020-2022 | Russian Federation Siberia | Domestic Galliformes | H5N8 |
| A/goose/Kazakhstan/7-20-B-Talg-12/2020(H5N8) | 2020-2022 | West Central Asia | Domestic Anseriformes | H5N8 |
| A/mute swan/Kazakhstan/1-267-20-B/2020(H5N8) | 2020-2022 | West Central Asia | Wild Anseriformes | H5N8 |
| A/swan/Kazakhstan/1-267-20-B-Talg-52/2020(H5N8) | 2020-2022 | West Central Asia | Wild Anseriformes | H5N8 |
| A/domestic duck/Kazakhstan/1-274-20-B/2020(H5N8) | 2020-2022 | West Central Asia | Domestic Anseriformes | H5N8 |
| A/chicken/Tyumen/302-01/2020(H5N8) | 2020-2022 | Russian Federation Siberia | Domestic Galliformes | H5N8 |
| A/chicken/Tyumen/302-02/2020(H5N8) | 2020-2022 | Russian Federation Siberia | Domestic Galliformes | H5N8 |
| A/green-winged-teal/Georgia/DT-22246/2020(H5N8) | 2020-2022 | West Central Asia | Wild Anseriformes | H5N8 |
| A/chicken/Russian Federation/Omsk/1680-10/2020(H5N5) | 2020-2022 | Russian Federation Siberia | Domestic Galliformes | H5N5 |
| A/domestic duck/Georgia/DT-22368/2020(H5N8) | 2020-2022 | West Central Asia | Domestic Anseriformes | H5N8 |
| A/goose/Russian Federation/Omsk/1680-6/2020(H5N5) | 2020-2022 | Russian Federation Siberia | Domestic Anseriformes | H5N5 |
| A/green-winged-teal/Georgia/DT-22332/2020(H5N8) | 2020-2022 | West Central Asia | Wild Anseriformes | H5N8 |
| A/mallard/Georgia/DT-22356/2020(H5N8) | 2020-2022 | West Central Asia | Wild Anseriformes | H5N8 |
| A/mallard/Georgia/DT-22360/2020(H5N8) | 2020-2022 | West Central Asia | Wild Anseriformes | H5N8 |
| A/mallard/Georgia/DT-22362/2020(H5N8) | 2020-2022 | West Central Asia | Wild Anseriformes | H5N8 |
| A/green-winged-teal/Georgia/DT-22392/2020(H5N8) | 2020-2022 | West Central Asia | Wild Anseriformes | H5N8 |
| A/mallard/Georgia/DT-22456/2020(H5N8) | 2020-2022 | West Central Asia | Wild Anseriformes | H5N8 |
| A/chicken/Egypt/MEN6/2020(H5N8) | 2020-2022 | Africa | Domestic Galliformes | H5N8 |
| A/chicken/Kazakhstan/1-20-B-Talg-67/2020(H5N8) | 2020-2022 | West Central Asia | Domestic Galliformes | H5N8 |
| A/goose/Tatarstan/1730-2/2020 (H5N8) | 2020-2022 | Russian Federation Siberia | Domestic Anseriformes | H5N8 |
| A/mallard/Georgia/DT-22495/2020(H5N8) | 2020-2022 | West Central Asia | Wild Anseriformes | H5N8 |
| A/common teal/Ningxia/105/2020(H5N8) | 2020-2022 | China | Wild Anseriformes | H5N8 |
| A/garganey/Georgia/DT-22572/2020(H5N8) | 2020-2022 | West Central Asia | Wild Anseriformes | H5N8 |
| A/mallard/Georgia/DT-22620/2020(H5N8) | 2020-2022 | West Central Asia | Wild Anseriformes | H5N8 |
| A/common teal/Ningxia/181/2020(H5N8) | 2020-2022 | China | Wild Anseriformes | H5N8 |
| A/common teal/Ningxia/189/2020(H5N8) | 2020-2022 | China | Wild Anseriformes | H5N8 |
| A/mallard/Ningxia/175/2020(H5N8) | 2020-2022 | China | Wild Anseriformes | H5N8 |
| A/mallard/Ningxia/176/2020(H5N8) | 2020-2022 | China | Wild Anseriformes | H5N8 |
| A/common pochard/Ningxia/243/2020(H5N8) | 2020-2022 | China | Wild Anseriformes | H5N8 |
| A/common teal/Ningxia/237/2020(H5N8) | 2020-2022 | China | Wild Anseriformes | H5N8 |
| A/common teal/Ningxia/245/2020(H5N8) | 2020-2022 | China | Wild Anseriformes | H5N8 |
| A/common teal/Ningxia/253/2020(H5N8) | 2020-2022 | China | Wild Anseriformes | H5N8 |
| A/Eurasian Wigeon/Netherlands/1/2020(H5N1) | 2020-2022 | West Europe | Wild Anseriformes | H5N1 |
| A/Eurasian Wigeon/Netherlands/4/2020(H5N1) | 2020-2022 | West Europe | Wild Anseriformes | H5N1 |
| A/Eurasian Wigeon/Netherlands/5/2020(H5N1) | 2020-2022 | West Europe | Wild Anseriformes | H5N1 |
| A/Eurasian Wigeon/Netherlands/7/2020(H5N8) | 2020-2022 | West Europe | Wild Anseriformes | H5N8 |
| A/garganey/Georgia/DT-22770/2020(H5N8) | 2020-2022 | West Central Asia | Wild Anseriformes | H5N8 |
| A/green-winged-teal/Georgia/DT-22792/2020(H5N8) | 2020-2022 | West Central Asia | Wild Anseriformes | H5N8 |
| A/green-winged-teal/Georgia/DT-22810/2020(H5N8) | 2020-2022 | West Central Asia | Wild Anseriformes | H5N8 |
| A/mallard/Ningxia/239/2020(H5N8) | 2020-2022 | China | Wild Anseriformes | H5N8 |
| A/mallard/Ningxia/241/2020(H5N8) | 2020-2022 | China | Wild Anseriformes | H5N8 |
| A/mallard/Ningxia/247/2020(H5N8) | 2020-2022 | China | Wild Anseriformes | H5N8 |
| A/mallard/Ningxia/249/2020(H5N8) | 2020-2022 | China | Wild Anseriformes | H5N8 |
| A/chicken/Kostroma/304-01/2020(H5N8) | 2020-2022 | East Europe | Domestic Galliformes | H5N8 |
| A/chicken/Kostroma/304-03/2020(H5N8) | 2020-2022 | East Europe | Domestic Galliformes | H5N8 |
| A/chicken/Kostroma/304-04/2020(H5N8) | 2020-2022 | East Europe | Domestic Galliformes | H5N8 |
| A/chicken/Kostroma/304-06/2020(H5N8) | 2020-2022 | East Europe | Domestic Galliformes | H5N8 |
| A/chicken/Kostroma/304-08/2020(H5N8) | 2020-2022 | East Europe | Domestic Galliformes | H5N8 |
| A/chicken/Kostroma/304-10/2020(H5N8) | 2020-2022 | East Europe | Domestic Galliformes | H5N8 |
| A/mute swan/Inner Mongolia/w2-1/2020(H5N8) | 2020-2022 | China | Wild Anseriformes | H5N8 |
| A/mute swan/Netherlands/20015931-001/2020(H5N8) | 2020-2022 | West Europe | Wild Anseriformes | H5N8 |
| A/whooper swan/Inner Mongolia/w1-1/2020(H5N8) | 2020-2022 | China | Wild Anseriformes | H5N8 |
| A/green-winged-teal/Georgia/DT-22894/2020(H5N3) | 2020-2022 | West Central Asia | Wild Anseriformes | H5N3 |
| A/mallard/Georgia/DT-22862/2020(H5N8) | 2020-2022 | West Central Asia | Wild Anseriformes | H5N8 |
| A/Mandarin duck/Korea/H242/2020(H5N8) | 2020-2022 | Korea | Wild Anseriformes | H5N8 |
| A/green-winged-teal/Georgia/DT-22978/2020(H5N8) | 2020-2022 | West Central Asia | Wild Anseriformes | H5N8 |
| A/Madarin duck/Korea/K20-551-4/2020(H5N8) | 2020-2022 | Korea | Wild Anseriformes | H5N8 |
| A/northern pintail/Hokkaido/M13/2020(H5N8) | 2020-2022 | Japan | Wild Anseriformes | H5N8 |
| A/chicken/Rostov-on-Don/308-02/2020(H5N8) | 2020-2022 | East Europe | Domestic Galliformes | H5N8 |
| A/chicken/Rostov-on-Don/308-03/2020(H5N8) | 2020-2022 | East Europe | Domestic Galliformes | H5N8 |
| A/chicken/Rostov-on-Don/308-04/2020(H5N8) | 2020-2022 | East Europe | Domestic Galliformes | H5N8 |
| A/greylag goose/Netherlands/20017058-002/2020(H5N8) | 2020-2022 | West Europe | Wild Anseriformes | H5N8 |
| A/greylag goose/Netherlands/20016414-001/2020(H5N8) | 2020-2022 | West Europe | Wild Anseriformes | H5N8 |
| A/greylag goose/Netherlands/20016523-001/2020(H5N8) | 2020-2022 | West Europe | Wild Anseriformes | H5N8 |
| A/peregrine falcon/Germany-SH/AI02162/2020(H5N8) | 2020-2022 | West Europe | Other wild species | H5N8 |
| A/wild bird/Netherlands/20016515-002/2020(H5N8) | 2020-2022 | West Europe | Other wild species | H5N8 |
| A/barnacle goose/Netherlands/20016511-002/2020(H5N8) | 2020-2022 | West Europe | Wild Anseriformes | H5N8 |
| A/green-winged-teal/Georgia/DT-23070/2020(H5N8) | 2020-2022 | West Central Asia | Wild Anseriformes | H5N8 |
| A/greylag goose/Netherlands/20016494-001/2020(H5N8) | 2020-2022 | West Europe | Wild Anseriformes | H5N8 |
| A/mallard/Georgia/DT-23072/2020(H5N8) | 2020-2022 | West Central Asia | Wild Anseriformes | H5N8 |
| A/mute swan/Netherlands/20016516-001/2020(H5N8) | 2020-2022 | West Europe | Wild Anseriformes | H5N8 |
| A/barnacle goose/Germany-SH/AI02167/2020(H5N8) | 2020-2022 | West Europe | Wild Anseriformes | H5N8 |
| A/barnacle goose/Germany-SH/AI02168/2020(H5N8) | 2020-2022 | West Europe | Wild Anseriformes | H5N8 |
| A/chicken/Netherlands/20016597-026030/2020(H5N8) | 2020-2022 | West Europe | Domestic Galliformes | H5N8 |
| A/cormorant/Netherlands/20016582-005/2020(H5N8) | 2020-2022 | West Europe | Other wild species | H5N8 |
| A/greylag goose/Netherlands/20016582-004/2020(H5N1) | 2020-2022 | West Europe | Wild Anseriformes | H5N1 |
| A/mute swan/Netherlands/20016618-001/2020(H5N8) | 2020-2022 | West Europe | Wild Anseriformes | H5N8 |
| A/mute swan/Netherlands/20016634-001/2020(H5N8) | 2020-2022 | West Europe | Wild Anseriformes | H5N8 |
| A/barnacle goose/Netherlands/20016888-001/2020(H5N8) | 2020-2022 | West Europe | Wild Anseriformes | H5N8 |
| A/barnacle goose/Netherlands/20016888-002/2020(H5N8) | 2020-2022 | West Europe | Wild Anseriformes | H5N8 |
| A/barnacle goose/Netherlands/20016888-003/2020(H5N8) | 2020-2022 | West Europe | Wild Anseriformes | H5N8 |
| A/buzzard/Germany-MV/AI02166/2020(H5N5) | 2020-2022 | West Europe | Other wild species | H5N5 |
| A/eurasian wigeon/Netherlands/20016758-001/2020(H5N8) | 2020-2022 | West Europe | Wild Anseriformes | H5N8 |
| A/greylag goose/Netherlands/20016756-001/2020(H5N8) | 2020-2022 | West Europe | Wild Anseriformes | H5N8 |
| A/white-tailed eagle/Germany-SH/AI02170/2020(H5N8) | 2020-2022 | West Europe | Other wild species | H5N8 |
| A/barnacle goose/Germany-SH/AI02172/2020(H5N8) | 2020-2022 | West Europe | Wild Anseriformes | H5N8 |
| A/Eurasian wigeon/Germany-SH/AI02176/2020(H5N8) | 2020-2022 | West Europe | Wild Anseriformes | H5N8 |
| A/Greylag goose/England/033100/2020(H5N8) | 2020-2022 | West Europe | Wild Anseriformes | H5N8 |
| A/greylag goose/Netherlands/20016877-001/2020(H5N8) | 2020-2022 | West Europe | Wild Anseriformes | H5N8 |
| A/peregrine falcon/Denmark/13776-1/2020-10-30(H5N5) | 2020-2022 | West Europe | Other wild species | H5N5 |
| A/barnacle goose/Netherlands/20016951-001/2020(H5N8) | 2020-2022 | West Europe | Wild Anseriformes | H5N8 |
| A/brant goose/Netherlands/20016948-002/2020(H5N8) | 2020-2022 | West Europe | Wild Anseriformes | H5N8 |
| A/greylag goose/Germany-SH/AI02191/2020(H5N8) | 2020-2022 | West Europe | Wild Anseriformes | H5N8 |
| A/barnacle goose/Germany-SH/AI02180/2020(H5N8) | 2020-2022 | West Europe | Wild Anseriformes | H5N8 |
| A/barnacle goose/Netherlands/20016935-002/2020(H5N8) | 2020-2022 | West Europe | Wild Anseriformes | H5N8 |
| A/barnacle goose/Netherlands/20016935-003/2020(H5N8) | 2020-2022 | West Europe | Wild Anseriformes | H5N8 |
| A/eurasian curlew/Netherlands/20016890-001/2020(H5N1) | 2020-2022 | West Europe | Charadriiformes | H5N8 |
| A/Eurasian wigeon/Germany-SH/AI02179/2020 (H5N8) | 2020-2022 | West Europe | Wild Anseriformes | H5N8 |
| A/gadwall/Netherlands/20017254-001/2020(H5N8) | 2020-2022 | West Europe | Wild Anseriformes | H5N8 |
| A/goose/China/21FU001/2020(H5N8) | 2020-2022 | China | Domestic Anseriformes | H5N8 |
| A/goose/China/21FU002/2020(H5N8) | 2020-2022 | China | Domestic Anseriformes | H5N8 |
| A/goose/China/21FU003/2020(H5N8) | 2020-2022 | China | Domestic Anseriformes | H5N8 |
| A/goose/China/21FU004/2020(H5N8) | 2020-2022 | China | Domestic Anseriformes | H5N8 |
| A/goose/China/21FU005/2020(H5N8) | 2020-2022 | China | Domestic Anseriformes | H5N8 |
| A/goose/China/21FU006/2020(H5N8) | 2020-2022 | China | Domestic Anseriformes | H5N8 |
| A/goose/China/21FU007/2020(H5N8) | 2020-2022 | China | Domestic Anseriformes | H5N8 |
| A/goose/China/21FU008/2020(H5N8) | 2020-2022 | China | Domestic Anseriformes | H5N8 |
| A/great egret/Netherlands/20017754-002/2020(H5N8) | 2020-2022 | West Europe | Other wild species | H5N8 |
| A/greylag goose/Netherlands/20016879-001/2020(H5N8) | 2020-2022 | West Europe | Wild Anseriformes | H5N8 |
| A/northern lapwing/Netherlands/20017255-001/2020(H5N8) | 2020-2022 | West Europe | Other wild species | H5N8 |
| A/peregrine falcon/Ireland/20VIR7872-1/2020(H5N8) | 2020-2022 | West Europe | Other wild species | H5N8 |
| A/peregrine falcon/Netherlands/20017773-002/2020(H5N8) | 2020-2022 | West Europe | Other wild species | H5N8 |
| A/wild goose/Netherlands/20017755-002/2020(H5N8) | 2020-2022 | West Europe | Wild Anseriformes | H5N8 |
| A/barnacle goose/Germany-SH/AI02190/2020(H5N8) | 2020-2022 | West Europe | Wild Anseriformes | H5N8 |
| A/barnacle goose/Netherlands/20016896-011/2020(H5N8) | 2020-2022 | West Europe | Wild Anseriformes | H5N8 |
| A/barnacle goose/Netherlands/20016896-012/2020(H5N8) | 2020-2022 | West Europe | Wild Anseriformes | H5N8 |
| A/barnacle goose/Netherlands/20016974-002/2020(H5N8) | 2020-2022 | West Europe | Wild Anseriformes | H5N8 |
| A/chicken/England/030720/2020(H5N8) | 2020-2022 | West Europe | Domestic Galliformes | H5N8 |
| A/chicken/Netherlands/20016978-001/2020(H5N8) | 2020-2022 | West Europe | Domestic Galliformes | H5N8 |
| A/eurasian curlew/Netherlands/20016896-019/2020(H5N8) | 2020-2022 | West Europe | Charadriiformes | H5N4 |
| A/eurasian teal/Netherlands/20016896-013/2020(H5N1) | 2020-2022 | West Europe | Wild Anseriformes | H5N1 |
| A/eurasian wigeon/Netherlands/20016896-025/2020(H5N8) | 2020-2022 | West Europe | Wild Anseriformes | H5N8 |
| A/greylag goose/Netherlands/20016896-001/2020(H5N8) | 2020-2022 | West Europe | Wild Anseriformes | H5N8 |
| A/greylag goose/Netherlands/20016975-004/2020(H5N8) | 2020-2022 | West Europe | Wild Anseriformes | H5N8 |
| A/herring gull/Germany-HH/AI02182/2020 (H5N8) | 2020-2022 | West Europe | Charadriiformes | H5N8 |
| A/herring gull/Germany-MV/AI02300/2020(H5N8) | 2020-2022 | West Europe | Charadriiformes | H5N8 |
| A/mute swan/Netherlands/20016960-001/2020(H5N8) | 2020-2022 | West Europe | Wild Anseriformes | H5N8 |
| A/mute swan/Netherlands/20016973-001/2020(H5N8) | 2020-2022 | West Europe | Wild Anseriformes | H5N8 |
| A/mute swan/Netherlands/20017061-001/2020(H5N8) | 2020-2022 | West Europe | Wild Anseriformes | H5N8 |
| A/short-eared owl/Netherlands/20016896-017/2020(H5N8) | 2020-2022 | West Europe | Other wild species | H5N8 |
| A/whooper swan/Henan/SMQ5/2020(H5N8) | 2020-2022 | China | Wild Anseriformes | H5N8 |
| A/wild duck/Germany-SH/AI02189/2020(H5N8) | 2020-2022 | West Europe | Wild Anseriformes | H5N8 |
| A/wild goose/Germany-SH/AI02194/2020(H5N8) | 2020-2022 | West Europe | Wild Anseriformes | H5N8 |
| A/wild goose/Netherlands/20016959-001/2020(H5N8) | 2020-2022 | West Europe | Wild Anseriformes | H5N8 |
| A/barnacle goose/Germany-SH/AI02199/2020(H5N8) | 2020-2022 | West Europe | Wild Anseriformes | H5N8 |
| A/barnacle goose/Netherlands/20017051-001/2020(H5N8) | 2020-2022 | West Europe | Wild Anseriformes | H5N8 |
| A/barnacle goose/Netherlands/20017051-006/2020(H5N8) | 2020-2022 | West Europe | Wild Anseriformes | H5N8 |
| A/barnacle goose/Netherlands/20017052-001/2020(H5N8) | 2020-2022 | West Europe | Wild Anseriformes | H5N8 |
| A/Canada goose/England/032697/2020(H5N8) | 2020-2022 | West Europe | Wild Anseriformes | H5N8 |
| A/egyptian goose/Netherlands/20017027-002/2020(H5N8) | 2020-2022 | West Europe | Wild Anseriformes | H5N8 |
| A/Greylag goose/England/032698/2020(H5N8) | 2020-2022 | West Europe | Wild Anseriformes | H5N8 |
| A/greylag goose/Germany-SH/AI02207/2020(H5N8) | 2020-2022 | West Europe | Wild Anseriformes | H5N8 |
| A/greylag goose/Netherlands/20017064-002/2020(H5N8) | 2020-2022 | West Europe | Wild Anseriformes | H5N8 |
| A/greylag goose/Netherlands/20017256-002/2020(H5N8) | 2020-2022 | West Europe | Wild Anseriformes | H5N8 |
| A/mute swan/Netherlands/20017153-002/2020(H5N8) | 2020-2022 | West Europe | Wild Anseriformes | H5N8 |
| A/spot-billed duck/Korea/WA612/2020(H5N8) | 2020-2022 | Korea | Wild Anseriformes | H5N8 |
| A/chicken/Kagawa/11C/2020(H5N8) | 2018-2019 | Japan | Domestic Galliformes | H5N6 |
| A/chicken/Kagawa/2T/2020(H5N8) | 2018-2019 | Japan | Domestic Galliformes | H5N6 |
| A/barnacle goose/Denmark/14138-1/2020-11-04(H5N8) | 2020-2022 | West Europe | Wild Anseriformes | H5N8 |
| A/barnacle goose/Denmark/14139-1/2020(H5N8) | 2020-2022 | West Europe | Wild Anseriformes | H5N8 |
| A/barnacle goose/Denmark/14139-2/2020(H5N8) | 2020-2022 | West Europe | Wild Anseriformes | H5N8 |
| A/barnacle goose/Denmark/14139-3/2020(H5N8) | 2020-2022 | West Europe | Wild Anseriformes | H5N8 |
| A/barnacle goose/Denmark/14534-1/2020(H5N8) | 2020-2022 | West Europe | Wild Anseriformes | H5N8 |
| A/barnacle goose/Denmark/14537-1/2020(H5N8) | 2020-2022 | West Europe | Wild Anseriformes | H5N8 |
| A/barnacle goose/Netherlands/20017159-006/2020(H5N8) | 2020-2022 | West Europe | Wild Anseriformes | H5N8 |
| A/black-headed gull/Denmark/14139-4/2020(H5N8) | 2020-2022 | West Europe | Charadriiformes | H5N8 |
| A/chicken/Kagawa/10T/2020(H5N8) | 2020-2022 | Japan | Domestic Galliformes | H5N8 |
| A/chicken/Kagawa/6T/2020(H5N8) | 2020-2022 | Japan | Domestic Galliformes | H5N8 |
| A/chicken/Kagawa/7C/2020(H5N8) | 2020-2022 | Japan | Domestic Galliformes | H5N8 |
| A/chicken/Kagawa/7T/2020(H5N8) | 2020-2022 | Japan | Domestic Galliformes | H5N8 |
| A/chicken/Netherlands/20017138-016020/2020(H5N8) | 2020-2022 | West Europe | Domestic Galliformes | H5N8 |
| A/Cygnus columbianus/Hubei/56/2020(H5N8) | 2020-2022 | China | Wild Anseriformes | H5N8 |
| A/Cygnus olor/Belgium/11956 001/2020 (H5N8) | 2020-2022 | West Europe | Wild Anseriformes | H5N8 |
| A/mute swan/Netherlands/20017131-006/2020(H5N8) | 2020-2022 | West Europe | Wild Anseriformes | H5N8 |
| A/tundra swan/Hubei/BQ2/2020(H5N8) | 2020-2022 | China | Wild Anseriformes | H5N8 |
| A/wild goose/Netherlands/20017761-002/2020(H5N8) | 2020-2022 | West Europe | Wild Anseriformes | H5N8 |
| A/barnacle goose/Denmark/14536-1/2020(H5N8) | 2020-2022 | West Europe | Wild Anseriformes | H5N8 |
| A/common kestrel/Netherlands/20017381-001/2020(H5N8) | 2020-2022 | West Europe | Other wild species | H5N8 |
| A/greylag goose/Netherlands/20017386-001/2020(H5N8) | 2020-2022 | West Europe | Wild Anseriformes | H5N8 |
| A/barnacle goose/Germany-SH/AI02379/2020(H5N8) | 2020-2022 | West Europe | Wild Anseriformes | H5N8 |
| A/common buzzard/Denmark/14600-1/2020(H5N8) | 2020-2022 | West Europe | Other wild species | H5N1 |
| A/Common Buzzard/Netherlands/4/2020(H5N8) | 2020-2022 | West Europe | Other wild species | H5N1 |
| A/Eurasian oystercatcher/Germany-SH/AI02269/2020(H5N8) | 2020-2022 | West Europe | Charadriiformes | H5N8 |
| A/greater canada goose/Netherlands/20017479-002/2020(H5N8) | 2020-2022 | West Europe | Wild Anseriformes | H5N8 |
| A/Greylag Goose/Netherlands/1/2020(H5N8) | 2020-2022 | West Europe | Wild Anseriformes | H5N1 |
| A/greylag goose/Netherlands/20017399-006/2020(H5N8) | 2020-2022 | West Europe | Wild Anseriformes | H5N8 |
| A/greylag goose/Netherlands/20017476-001/2020(H5N8) | 2020-2022 | West Europe | Wild Anseriformes | H5N8 |
| A/Herring Gull/Netherlands/1/2020(H5N8) | 2020-2022 | West Europe | Charadriiformes | H5N8 |
| A/Herring Gull/Netherlands/2/2020(H5N8) | 2020-2022 | West Europe | Charadriiformes | H5N8 |
| A/Herring Gull/Netherlands/3/2020(H5N8) | 2020-2022 | West Europe | Charadriiformes | H5N8 |
| A/Mute Swan/Netherlands/1/2020(H5N8) | 2020-2022 | West Europe | Wild Anseriformes | H5N8 |
| A/Mute Swan/Netherlands/3/2020(H5N8) | 2020-2022 | West Europe | Wild Anseriformes | H5N8 |
| A/Mute Swan/Netherlands/5/2020(H5N8) | 2020-2022 | West Europe | Wild Anseriformes | H5N8 |
| A/northern lapwing/Netherlands/20017480-001/2020(H5N8) | 2020-2022 | West Europe | Other wild species | H5N8 |
| A/peregrine falcon/Denmark/14596-1/2020(H5N8) | 2020-2022 | West Europe | Other wild species | H5N8 |
| A/pink-footed goose/Netherlands/20017382-001/2020(H5N8) | 2020-2022 | West Europe | Wild Anseriformes | H5N8 |
| A/whooper swan/Henan/SMQ6/2020(H5N8) | 2020-2022 | China | Wild Anseriformes | H5N8 |
| A/Anser albifrons/Belgium/11956 005/2020(H5N8) | 2020-2022 | West Europe | Wild Anseriformes | H5N8 |
| A/barnacle goose/Denmark/14599-1/2020(H5N8) | 2020-2022 | West Europe | Wild Anseriformes | H5N8 |
| A/barnacle goose/Denmark/14600-2/2020(H5N8) | 2020-2022 | West Europe | Wild Anseriformes | H5N8 |
| A/barnacle goose/Netherlands/20017405-002/2020(H5N8) | 2020-2022 | West Europe | Wild Anseriformes | H5N8 |
| A/chicken/Kagawa/B10T/2020(H5N8) | 2020-2022 | Japan | Domestic Galliformes | H5N8 |
| A/chicken/Kagawa/B13C/2020(H5N8) | 2020-2022 | Japan | Domestic Galliformes | H5N8 |
| A/chicken/Kagawa/B7T/2020(H5N8) | 2020-2022 | Japan | Domestic Galliformes | H5N8 |
| A/chicken/Kagawa/B8T/2020(H5N8) | 2020-2022 | Japan | Domestic Galliformes | H5N8 |
| A/chicken/Kagawa/B9T/2020(H5N8) | 2020-2022 | Japan | Domestic Galliformes | H5N8 |
| A/greater canada goose/Netherlands/20017403-003/2020(H5N8) | 2020-2022 | West Europe | Wild Anseriformes | H5N8 |
| A/greater white-fronted goose/Netherlands/20017403-004/2020(H5N5) | 2020-2022 | West Europe | Wild Anseriformes | H5N5 |
| A/Numenius arquata/Belgium/11956 003/2020(H5N8) | 2020-2022 | West Europe | Charadriiformes | H5N8 |
| A/barnacle goose/Netherlands/20017604-001/2020(H5N8) | 2020-2022 | West Europe | Wild Anseriformes | H5N8 |
| A/barnacle goose/Netherlands/20017713-002/2020(H5N8) | 2020-2022 | West Europe | Wild Anseriformes | H5N8 |
| A/brent goose/England/233339/2020(H5N8) | 2020-2022 | West Europe | Wild Anseriformes | H5N8 |
| A/chicken/Germany-MV/AI02431/2020 (H5N5) | 2020-2022 | West Europe | Domestic Galliformes | H5N5 |
| A/chicken/Netherlands/20017460-011015/2020(H5N8) | 2020-2022 | West Europe | Domestic Galliformes | H5N8 |
| A/graylag goose/Denmark/14535-1/2020(H5N8) | 2020-2022 | West Europe | Wild Anseriformes | H5N8 |
| A/swan/Netherlands/20017605-002/2020(H5N1) | 2020-2022 | West Europe | Wild Anseriformes | H5N1 |
| A/tundra swan/Hubei/BQ3/2020(H5N8) | 2020-2022 | China | Wild Anseriformes | H5N8 |
| A/whooper swan/Henan/SMQ7/2020(H5N8) | 2020-2022 | China | Wild Anseriformes | H5N8 |
| A/whooper swan/Shaanxi/SXY26/2020(H5N8) | 2020-2022 | China | Wild Anseriformes | H5N8 |
| A/wild goose/Netherlands/20017495-002/2020(H5N8) | 2020-2022 | West Europe | Wild Anseriformes | H5N8 |
| A/barnacle goose/Netherlands/20017557-001/2020(H5N8) | 2020-2022 | West Europe | Wild Anseriformes | H5N8 |
| A/barnacle goose/Netherlands/20017557-002/2020(H5N8) | 2020-2022 | West Europe | Wild Anseriformes | H5N8 |
| A/chicken/England/033708/2020(H5N8) | 2020-2022 | West Europe | Domestic Galliformes | H5N8 |
| A/chicken/France/20P016448/2020(H5N8) | 2020-2022 | West Europe | Domestic Galliformes | H5N8 |
| A/chicken/Kagawa/C1T/2020(H5N8) | 2020-2022 | Japan | Domestic Galliformes | H5N8 |
| A/chicken/Kagawa/C2T/2020(H5N8) | 2020-2022 | Japan | Domestic Galliformes | H5N8 |
| A/chicken/Kagawa/C3C/2020(H5N8) | 2020-2022 | Japan | Domestic Galliformes | H5N8 |
| A/chicken/Kagawa/C3T/2020(H5N8) | 2020-2022 | Japan | Domestic Galliformes | H5N8 |
| A/chicken/Kagawa/C5T/2020(H5N8) | 2020-2022 | Japan | Domestic Galliformes | H5N8 |
| A/chicken/Netherlands/20017639-001/2020(H5N8) | 2020-2022 | West Europe | Domestic Galliformes | H5N8 |
| A/common teal/Shaanxi/SXY1-1/2020(H5N8) | 2020-2022 | China | Wild Anseriformes | H5N8 |
| A/eurasian oystercatcher/Netherlands/20017557-003/2020(H5N8) | 2020-2022 | West Europe | Charadriiformes | H5N8 |
| A/eurasian wigeon/Netherlands/20017908-002/2020(H5N8) | 2020-2022 | West Europe | Wild Anseriformes | H5N8 |
| A/gadwall/Netherlands/20017716-001/2020(H5N8) | 2020-2022 | West Europe | Wild Anseriformes | H5N8 |
| A/mandarin duck/Korea/WB80/2020(H5N8) | 2020-2022 | Korea | Wild Anseriformes | H5N8 |
| A/muscovy duck/Netherlands/20017611-002/2020(H5N8) | 2020-2022 | West Europe | Wild Anseriformes | H5N8 |
| A/mute swan/England/263814/2020(H5N8) | 2020-2022 | West Europe | Wild Anseriformes | H5N8 |
| A/mute swan/Netherlands/20017547-002/2020(H5N8) | 2020-2022 | West Europe | Wild Anseriformes | H5N8 |
| A/mute swan/Netherlands/20017717-002/2020(H5N8) | 2020-2022 | West Europe | Wild Anseriformes | H5N8 |
| A/Peregrine falcon/Sweden/SVA201117SZ0467/20KN003345/2020(H5N8) | 2020-2022 | West Europe | Other wild species | H5N8 |
| A/swan/Netherlands/20017772-002/2020(H5N1) | 2020-2022 | West Europe | Wild Anseriformes | H5N1 |
| A/whooper swan/Henan/SM1/2020(H5N8) | 2020-2022 | China | Wild Anseriformes | H5N8 |
| A/whooper swan/Henan/SM111/2020(H5N8) | 2020-2022 | China | Wild Anseriformes | H5N8 |
| A/whooper swan/Henan/SM16/2020(H5N8) | 2020-2022 | China | Wild Anseriformes | H5N8 |
| A/whooper swan/Henan/SM31/2020(H5N8) | 2020-2022 | China | Wild Anseriformes | H5N8 |
| A/whooper swan/Henan/SM61/2020(H5N8) | 2020-2022 | China | Wild Anseriformes | H5N8 |
| A/whooper swan/Henan/SM76/2020(H5N8) | 2020-2022 | China | Wild Anseriformes | H5N8 |
| A/whooper swan/Henan/SM86/2020(H5N8) | 2020-2022 | China | Wild Anseriformes | H5N8 |
| A/whooper swan/Henan/SMQ10/2020(H5N8) | 2020-2022 | China | Wild Anseriformes | H5N8 |
| A/whooper swan/Henan/SMQ9/2020(H5N8) | 2020-2022 | China | Wild Anseriformes | H5N8 |
| A/whooper swan/Shaanxi/SXY2-1/2020(H5N8) | 2020-2022 | China | Wild Anseriformes | H5N8 |
| A/whooper swan/Shaanxi/SXY66/2020(H5N8) | 2020-2022 | China | Wild Anseriformes | H5N8 |
| A/whooper swan/Shanxi/4-1/2020(H5N8) | 2020-2022 | China | Wild Anseriformes | H5N8 |
| A/whooper swan/Shanxi/4-2/2020(H5N8) | 2020-2022 | China | Wild Anseriformes | H5N8 |
| A/whooper swan/Shanxi/SX106/2020(H5N8) | 2020-2022 | China | Wild Anseriformes | H5N8 |
| A/whooper swan/Shanxi/SX116/2020(H5N2) | 2020-2022 | China | Wild Anseriformes | H5N2 |
| A/whooper swan/Shanxi/SX16/2020(H5N8) | 2020-2022 | China | Wild Anseriformes | H5N8 |
| A/whooper swan/Shanxi/SX166/2020(H5N8) | 2020-2022 | China | Wild Anseriformes | H5N8 |
| A/whooper swan/Shanxi/SX206/2020(H5N8) | 2020-2022 | China | Wild Anseriformes | H5N8 |
| A/whooper swan/Shanxi/SX216/2020(H5N8) | 2020-2022 | China | Wild Anseriformes | H5N8 |
| A/whooper swan/Shanxi/SX231/2020(H5N8) | 2020-2022 | China | Wild Anseriformes | H5N8 |
| A/whooper swan/Shanxi/SX251/2020(H5N8) | 2020-2022 | China | Wild Anseriformes | H5N8 |
| A/whooper swan/Shanxi/SX276/2020(H5N8) | 2020-2022 | China | Wild Anseriformes | H5N8 |
| A/whooper swan/Shanxi/SX291/2020(H5N8) | 2020-2022 | China | Wild Anseriformes | H5N8 |
| A/whooper swan/Shanxi/SX31/2020(H5N8) | 2020-2022 | China | Wild Anseriformes | H5N8 |
| A/whooper swan/Shanxi/SX346/2020(H5N8) | 2020-2022 | China | Wild Anseriformes | H5N8 |
| A/whooper swan/Shanxi/SX56/2020(H5N8) | 2020-2022 | China | Wild Anseriformes | H5N8 |
| A/wild goose/Netherlands/20017819-001/2020(H5N8) | 2020-2022 | West Europe | Wild Anseriformes | H5N8 |
| A/chicken/Egypt/MEN10/2020(H5N8) | 2020-2022 | Africa | Domestic Galliformes | H5N8 |
| A/chicken/Netherlands/20017694-004/2020(H5N8) | 2020-2022 | West Europe | Domestic Galliformes | H5N8 |
| A/eurasian eagle-owl/Henan/SMQ11/2020(H5N8) | 2020-2022 | China | Other wild species | H5N8 |
| A/tundra swan/Hubei/BQ4/2020(H5N8) | 2020-2022 | China | Wild Anseriformes | H5N8 |
| A/wild goose/Netherlands/20017816-001/2020(H5N8) | 2020-2022 | West Europe | Wild Anseriformes | H5N8 |
| A/barnacle goose/Sweden/SVA201117SZ0468/KN003355/2020(H5N8) | 2020-2022 | West Europe | Wild Anseriformes | H5N8 |
| A/brent goose/England/095684/2020(H5N5) | 2020-2022 | West Europe | Wild Anseriformes | H5N5 |
| A/chicken/Kagawa/D1T/2020(H5N8) | 2020-2022 | Japan | Domestic Galliformes | H5N8 |
| A/chicken/Kagawa/D2C/2020(H5N8) | 2020-2022 | Japan | Domestic Galliformes | H5N8 |
| A/chicken/Kagawa/D2T/2020(H5N8) | 2020-2022 | Japan | Domestic Galliformes | H5N8 |
| A/chicken/Kagawa/D3T/2020(H5N8) | 2020-2022 | Japan | Domestic Galliformes | H5N8 |
| A/chicken/Kagawa/D5T/2020(H5N8) | 2020-2022 | Japan | Domestic Galliformes | H5N8 |
| A/common buzzard/Netherlands/20017824-001/2020(H5N8) | 2020-2022 | West Europe | Other wild species | H5N1 |
| A/Cygnus columbianus/Hubei/49/2020(H5N8) | 2020-2022 | China | Wild Anseriformes | H5N8 |
| A/Cygnus columbianus/Hubei/50/2020(H5N8) | 2020-2022 | China | Wild Anseriformes | H5N8 |
| A/Cygnus columbianus/Hubei/51/2020(H5N8) | 2020-2022 | China | Wild Anseriformes | H5N8 |
| A/muscovy duck/Netherlands/20018067-001/2020(H5N8) | 2020-2022 | West Europe | Wild Anseriformes | H5N8 |
| A/wild duck/Netherlands/20017794-001/2020(H5N8) | 2020-2022 | West Europe | Wild Anseriformes | H5N8 |
| A/barnacle goose/Netherlands/20017984-004/2020(H5N8) | 2020-2022 | West Europe | Wild Anseriformes | H5N8 |
| A/duck/Netherlands/20017868-016020/2020(H5N8) | 2020-2022 | West Europe | Domestic Anseriformes | H5N8 |
| A/Pica pica/Belgium/12100 005/2020(H5N8) | 2020-2022 | West Europe | Other wild species | H5N8 |
| A/Turkey/Sweden/SVA201114SZ0001/20KN303106/2020(H5N8) | 2020-2022 | West Europe | Domestic Galliformes | H5N8 |
| A/chicken/Kagawa/E13T/2020(H5N8) | 2020-2022 | Japan | Domestic Galliformes | H5N8 |
| A/chicken/Kagawa/E4T/2020(H5N8) | 2020-2022 | Japan | Domestic Galliformes | H5N8 |
| A/chicken/Kagawa/E5T/2020(H5N8) | 2020-2022 | Japan | Domestic Galliformes | H5N8 |
| A/chicken/Kagawa/E7T/2020(H5N8) | 2020-2022 | Japan | Domestic Galliformes | H5N8 |
| A/Eurasian wigeon/Italy/20VIR7139-121/2020(H5N8) | 2020-2022 | West Europe | Wild Anseriformes | H5N8 |
| A/mallard/Italy/20VIR7139-124 feather/2020(H5N8) | 2020-2022 | West Europe | Wild Anseriformes | H5N8 |
| A/mallard/Italy/20VIR7139-73/2020(H5N8) | 2020-2022 | West Europe | Wild Anseriformes | H5N8 |
| A/pink-footed goose/Netherlands/20018068-001/2020(H5N8) | 2020-2022 | West Europe | Wild Anseriformes | H5N8 |
| A/barnacle goose/Denmark/14538-1/2020(H5N8) | 2020-2022 | West Europe | Wild Anseriformes | H5N8 |
| A/chicken/Denmark/14819-6/2020(H5N8) | 2020-2022 | West Europe | Domestic Galliformes | H5N8 |
| A/greylag goose/Netherlands/20018070-002/2020(H5N8) | 2020-2022 | West Europe | Wild Anseriformes | H5N8 |
| A/bean goose/Hubei/BQ11/2020(H5N8) | 2020-2022 | China | Wild Anseriformes | H5N8 |
| A/black swan/Netherlands/20018185-001/2020(H5N8) | 2020-2022 | West Europe | Wild Anseriformes | H5N8 |
| A/Chlidonias hybrida/Hubei/55/2020(H5N8) | 2020-2022 | China | Charadriiformes | H5N8 |
| A/common buzzard/Netherlands/20018339-002/2020(H5N8) | 2020-2022 | West Europe | Other wild species | H5N8 |
| A/Cygnus columbianus/Hubei/116/2020(H5N8) | 2020-2022 | China | Wild Anseriformes | H5N8 |
| A/Cygnus columbianus/Hubei/52/2020(H5N8) | 2020-2022 | China | Wild Anseriformes | H5N8 |
| A/Cygnus columbianus/Hubei/53/2020(H5N8) | 2020-2022 | China | Wild Anseriformes | H5N8 |
| A/swan/Niigata/151118/2020(H5N8) | 2020-2022 | Africa | Wild Anseriformes | H5N8 |
| A/tundra swan/Hubei/BQ6/2020(H5N8) | 2020-2022 | China | Wild Anseriformes | H5N8 |
| A/tundra swan/Hubei/BQ7/2020(H5N8) | 2020-2022 | China | Wild Anseriformes | H5N8 |
| A/tundra swan/Hubei/BQ8/2020(H5N8) | 2020-2022 | China | Wild Anseriformes | H5N8 |
| A/tundra swan/Hubei/BQ9/2020(H5N8) | 2020-2022 | China | Wild Anseriformes | H5N8 |
| A/whiskered tern/Hubei/BQ10/2020(H5N8) | 2020-2022 | China | Charadriiformes | H5N8 |
| A/common buzzard/Italy/21VIR431-7/2020(H5N8) | 2016-2017 | West Europe | Other wild species | H5N8 |
| A/mute swan/Slovenia/1639-20 21VIR959-1/2020(H5N8) | 2020-2022 | East Europe | Wild Anseriformes | H5N8 |
| A/turkey/Croatia/104/2020(H5N8) | 2020-2022 | East Europe | Domestic Galliformes | H5N8 |
| A/wild duck/Korea/H331/2020(H5N8) | 2020-2022 | Korea | Wild Anseriformes | H5N8 |
| A/domestic goose/Germany-SH/AI02884/2020(H5N8) | 2020-2022 | West Europe | Domestic Anseriformes | H5N8 |
| A/Gallus gallus/Belgium/12168 002/2020(H5N5) | 2020-2022 | West Europe | Domestic Galliformes | H5N5 |
| A/chicken/Kagawa/F2T/2020(H5N8) | 2020-2022 | Japan | Domestic Galliformes | H5N8 |
| A/chicken/Kagawa/F3C/2020(H5N8) | 2020-2022 | Japan | Domestic Galliformes | H5N8 |
| A/chicken/Kagawa/F3T/2020(H5N8) | 2020-2022 | Japan | Domestic Galliformes | H5N8 |
| A/chicken/Kagawa/F4T/2020(H5N8) | 2020-2022 | Japan | Domestic Galliformes | H5N8 |
| A/chicken/Kagawa/F5T/2020(H5N8) | 2020-2022 | Japan | Domestic Galliformes | H5N8 |
| A/chicken/Kagawa/G10T/2020(H5N8) | 2020-2022 | Japan | Domestic Galliformes | H5N8 |
| A/chicken/Kagawa/G12C/2020(H5N8) | 2020-2022 | Japan | Domestic Galliformes | H5N8 |
| A/chicken/Kagawa/G12T/2020(H5N8) | 2020-2022 | Japan | Domestic Galliformes | H5N8 |
| A/chicken/Kagawa/G4T/2020(H5N8) | 2020-2022 | Japan | Domestic Galliformes | H5N8 |
| A/whistling duck/England/035643/2020(H5N8) | 2020-2022 | West Europe | Wild Anseriformes | H5N8 |
| A/wild goose/Germany-NI/AI03101/2020 (H5N8) | 2020-2022 | West Europe | Wild Anseriformes | H5N8 |
| A/chicken/Kagawa/H1T/2020(H5N8) | 2020-2022 | Japan | Domestic Galliformes | H5N8 |
| A/chicken/Kagawa/H2T/2020(H5N8) | 2020-2022 | Japan | Domestic Galliformes | H5N8 |
| A/chicken/Kagawa/H4T/2020(H5N8) | 2020-2022 | Japan | Domestic Galliformes | H5N8 |
| A/chicken/Kagawa/H8C/2020(H5N8) | 2020-2022 | Japan | Domestic Galliformes | H5N8 |
| A/chicken/Kagawa/H8T/2020(H5N8) | 2020-2022 | Japan | Domestic Galliformes | H5N8 |
| A/chicken/Netherlands/20018496-006010/2020(H5N8) | 2020-2022 | West Europe | Domestic Galliformes | H5N8 |
| A/peregrine falcon/Spain/3365-1 21VIR1230-1/2020(H5N8) | 2020-2022 | West Europe | Other wild species | H5N8 |
| A/chicken/England/037052/2020(H5N8) | 2020-2022 | West Europe | Domestic Galliformes | H5N8 |
| A/chicken/Netherlands/20018523-001005/2020(H5N8) | 2020-2022 | West Europe | Domestic Galliformes | H5N8 |
| A/Eurasian wigeon/Italy/20VIR7301-206/2020(H5N1) | 2020-2022 | West Europe | Wild Anseriformes | H5N1 |
| A/Eurasian wigeon/Italy/20VIR7301-31/2020(H5N8) | 2020-2022 | West Europe | Wild Anseriformes | H5N8 |
| A/Eurasian wigeon/Italy/20VIR7301-34/2020(H5N8) | 2020-2022 | West Europe | Wild Anseriformes | H5N8 |
| A/Eurasian wigeon/Italy/20VIR7301-362/2020(H5N8) | 2020-2022 | West Europe | Wild Anseriformes | H5N8 |
| A/northern goshawk/Netherlands/20018560-002/2020(H5N8) | 2020-2022 | West Europe | Other wild species | H5N8 |
| A/greater white-fronted goose/Italy/20VIR8073-4/2020(H5N1) | 2020-2022 | West Europe | Wild Anseriformes | H5N1 |
| A/wild bird/Korea/H357/2020(H5N8) | 2020-2022 | Korea | Other wild species | H5N8 |
| A/barnacle goose/Netherlands/20018737-002/2020(H5N8) | 2020-2022 | West Europe | Wild Anseriformes | H5N8 |
| A/chicken/Fukuoka/C1/2020(H5N8) | 2020-2022 | Japan | Domestic Galliformes | H5N8 |
| A/chicken/Fukuoka/C2/2020(H5N8) | 2020-2022 | Japan | Domestic Galliformes | H5N8 |
| A/chicken/Fukuoka/T1/2020(H5N8) | 2020-2022 | Japan | Domestic Galliformes | H5N8 |
| A/chicken/Fukuoka/T2/2020(H5N8) | 2020-2022 | Japan | Domestic Galliformes | H5N8 |
| A/chicken/Poland/448/2020(H5N8) | 2020-2022 | East Europe | Domestic Galliformes | H5N8 |
| A/mute swan/Netherlands/20018738-001/2020(H5N8) | 2020-2022 | West Europe | Wild Anseriformes | H5N8 |
| A/mute swan/Netherlands/20018754-004/2020(H5N8) | 2020-2022 | West Europe | Wild Anseriformes | H5N8 |
| A/mute swan/Netherlands/20018754-006/2020(H5N8) | 2020-2022 | West Europe | Wild Anseriformes | H5N8 |
| A/mute swan/Wales/048068/2020(H5N5) | 2020-2022 | West Europe | Wild Anseriformes | H5N5 |
| A/Podiceps cristatus/Belgium/12659 0015/2020(H5N8) | 2020-2022 | West Europe | Other wild species | H5N8 |
| A/wild goose/Netherlands/20018735-002/2020(H5N8) | 2020-2022 | West Europe | Wild Anseriformes | H5N8 |
| A/chicken/Hyogo/1T/2020(H5N8) | 2020-2022 | Japan | Domestic Galliformes | H5N8 |
| A/chicken/Hyogo/2T/2020(H5N8) | 2020-2022 | Japan | Domestic Galliformes | H5N8 |
| A/chicken/Hyogo/3T/2020(H5N8) | 2020-2022 | Japan | Domestic Galliformes | H5N8 |
| A/chicken/Hyogo/4T/2020(H5N8) | 2020-2022 | Japan | Domestic Galliformes | H5N8 |
| A/mallard/Korea/WA820/2020(H5N8) | 2020-2022 | Korea | Wild Anseriformes | H5N8 |
| A/mandarin duck/Korea/WA831/2020(H5N8) | 2020-2022 | Korea | Wild Anseriformes | H5N8 |
| A/mute swan/Netherlands/20018824-002/2020(H5N8) | 2020-2022 | West Europe | Wild Anseriformes | H5N8 |
| A/peregrine falcon/Netherlands/20018821-002/2020(H5N8) | 2020-2022 | West Europe | Other wild species | H5N8 |
| A/swan/Netherlands/20018830-004/2020(H5N8) | 2020-2022 | West Europe | Wild Anseriformes | H5N8 |
| A/wild goose/Netherlands/20018822-002/2020(H5N8) | 2020-2022 | West Europe | Wild Anseriformes | H5N8 |
| A/common snipe/Netherlands/20018931-003/2020(H5N8) | 2020-2022 | West Europe | Other wild species | H5N8 |
| A/duck/Korea/H338/2020(H5N8) | 2020-2022 | Korea | Domestic Anseriformes | H5N8 |
| A/mandarin duck/Korea/WA857/2020(H5N8) | 2020-2022 | Korea | Wild Anseriformes | H5N8 |
| A/mute swan/Netherlands/20018923-001/2020(H5N8) | 2020-2022 | West Europe | Wild Anseriformes | H5N8 |
| A/spot-billed duck/Korea/WA854/2020(H5N8) | 2020-2022 | Korea | Wild Anseriformes | H5N8 |
| A/common teal/Italy/20VIR7439-190/2020(H5N5) | 2020-2022 | West Europe | Wild Anseriformes | H5N5 |
| A/common teal/Italy/20VIR7439-191/2020(H5N8) | 2020-2022 | West Europe | Wild Anseriformes | H5N8 |
| A/turkey/England/037784/2020(H5N8) | 2020-2022 | West Europe | Domestic Galliformes | H5N8 |
| A/greylag goose/Italy/20VIR7660-6/2020(H5N8) | 2020-2022 | West Europe | Wild Anseriformes | H5N8 |
| A/chicken/Miyazaki/1T/2020(H5N8) | 2020-2022 | Japan | Domestic Galliformes | H5N8 |
| A/chicken/Miyazaki/2T/2020(H5N8) | 2020-2022 | Japan | Domestic Galliformes | H5N8 |
| A/chicken/Miyazaki/4T/2020(H5N8) | 2020-2022 | Japan | Domestic Galliformes | H5N8 |
| A/chicken/Miyazaki/6T/2020(H5N8) | 2020-2022 | Japan | Domestic Galliformes | H5N8 |
| A/mute swan/Netherlands/20019137-005/2020(H5N8) | 2020-2022 | West Europe | Wild Anseriformes | H5N8 |
| A/turkey/Norway/FU496/2020(H5N8) | 2020-2022 | West Europe | Domestic Galliformes | H5N8 |
| A/chicken/Kagawa/I1C/2020(H5N8) | 2020-2022 | Japan | Domestic Galliformes | H5N8 |
| A/chicken/Kagawa/I1T/2020(H5N8) | 2020-2022 | Japan | Domestic Galliformes | H5N8 |
| A/chicken/Kagawa/I2C/2020(H5N8) | 2020-2022 | Japan | Domestic Galliformes | H5N8 |
| A/chicken/Kagawa/I2T/2020(H5N8) | 2020-2022 | Japan | Domestic Galliformes | H5N8 |
| A/chicken/Kagawa/I4C/2020(H5N8) | 2020-2022 | Japan | Domestic Galliformes | H5N8 |
| A/chicken/Kagawa/I4T/2020(H5N8) | 2020-2022 | Japan | Domestic Galliformes | H5N8 |
| A/chicken/Kagawa/I5C/2020(H5N8) | 2020-2022 | Japan | Domestic Galliformes | H5N8 |
| A/chicken/Kagawa/I5T/2020(H5N8) | 2020-2022 | Japan | Domestic Galliformes | H5N8 |
| A/chicken/Kagawa/I6C/2020(H5N8) | 2020-2022 | Japan | Domestic Galliformes | H5N8 |
| A/chicken/Kagawa/I6T/2020(H5N8) | 2020-2022 | Japan | Domestic Galliformes | H5N8 |
| A/chicken/Kagawa/J3C/2020(H5N8) | 2020-2022 | Japan | Domestic Galliformes | H5N8 |
| A/chicken/Kagawa/J3T/2020(H5N8) | 2020-2022 | Japan | Domestic Galliformes | H5N8 |
| A/chicken/Kagawa/J4C/2020(H5N8) | 2020-2022 | Japan | Domestic Galliformes | H5N8 |
| A/chicken/Kagawa/J4T/2020(H5N8) | 2020-2022 | Japan | Domestic Galliformes | H5N8 |
| A/chicken/Kagawa/J7C/2020(H5N8) | 2020-2022 | Japan | Domestic Galliformes | H5N8 |
| A/chicken/Kagawa/J7T/2020(H5N8) | 2020-2022 | Japan | Domestic Galliformes | H5N8 |
| A/chicken/Kagawa/J8C/2020(H5N8) | 2020-2022 | Japan | Domestic Galliformes | H5N8 |
| A/chicken/Kagawa/J8T/2020(H5N8) | 2020-2022 | Japan | Domestic Galliformes | H5N8 |
| A/chicken/Kagawa/J9C/2020(H5N8) | 2020-2022 | Japan | Domestic Galliformes | H5N8 |
| A/chicken/Kagawa/J9T/2020(H5N8) | 2020-2022 | Japan | Domestic Galliformes | H5N8 |
| A/chicken/Korea/H365/2020(H5N8) | 2020-2022 | Korea | Domestic Galliformes | H5N8 |
| A/chicken/Miyazaki/B10T/2020(H5N8) | 2020-2022 | Japan | Domestic Galliformes | H5N8 |
| A/chicken/Miyazaki/B1T/2020(H5N8) | 2020-2022 | Japan | Domestic Galliformes | H5N8 |
| A/chicken/Miyazaki/B2T/2020(H5N8) | 2020-2022 | Japan | Domestic Galliformes | H5N8 |
| A/chicken/Miyazaki/B6T/2020(H5N8) | 2020-2022 | Japan | Domestic Galliformes | H5N8 |
| A/mandarin duck/Korea/WA877/2020(H5N8) | 2020-2022 | Korea | Wild Anseriformes | H5N8 |
| A/mandarin duck/Korea/WA899/2020(H5N8) | 2020-2022 | Korea | Wild Anseriformes | H5N8 |
| A/mandarin duck/Korea/WA913/2020(H5N8) | 2020-2022 | Korea | Wild Anseriformes | H5N8 |
| A/mute swan/England/234135/2020(H5N8) | 2020-2022 | West Europe | Wild Anseriformes | H5N8 |
| A/spot-billed duck/Korea/WA889/2020(H5N8) | 2020-2022 | Korea | Wild Anseriformes | H5N8 |
| A/turkey/Poland/464/2020(H5N8) | 2020-2022 | East Europe | Domestic Galliformes | H5N8 |
| A/chicken/Miyazaki/C4T/2020(H5N8) | 2020-2022 | Japan | Domestic Galliformes | H5N8 |
| A/chicken/Miyazaki/C6T/2020(H5N8) | 2020-2022 | Japan | Domestic Galliformes | H5N8 |
| A/chicken/Miyazaki/C7T/2020(H5N8) | 2020-2022 | Japan | Domestic Galliformes | H5N8 |
| A/chicken/Miyazaki/C8T/2020(H5N8) | 2020-2022 | Japan | Domestic Galliformes | H5N8 |
| A/chicken/Netherlands/20019226-001/2020(H5N8) | 2020-2022 | West Europe | Domestic Galliformes | H5N8 |
| A/chicken/Netherlands/20019237-001005/2020(H5N8) | 2020-2022 | West Europe | Domestic Galliformes | H5N8 |
| A/mute swan/Netherlands/20019252-002/2020(H5N8) | 2020-2022 | West Europe | Wild Anseriformes | H5N8 |
| A/mute swan/Netherlands/20019255-002/2020(H5N8) | 2020-2022 | West Europe | Wild Anseriformes | H5N8 |
| A/mute swan/Slovenia/1756-20 21VIR959-3/2020(H5N8) | 2020-2022 | East Europe | Wild Anseriformes | H5N8 |
| A/turkey/England/038115/2020(H5N8) | 2020-2022 | West Europe | Domestic Galliformes | H5N8 |
| A/wild bird/Korea/H379/2020(H5N8) | 2020-2022 | Korea | Other wild species | H5N8 |
| A/Anser brachyrhynchus/Belgium/13275 0009/2020(H5N8) | 2020-2022 | West Europe | Wild Anseriformes | H5N8 |
| A/Anser Brachyrhynchus Anser Anser/Belgium/13846/2020(H5N8) | 2020-2022 | West Europe | Wild Anseriformes | H5N8 |
| A/chicken/Poland/474/2020(H5N8) | 2020-2022 | East Europe | Domestic Galliformes | H5N8 |
| A/mute swan/England/234255/2020(H5N1) | 2020-2022 | West Europe | Wild Anseriformes | H5N1 |
| A/turkey/England/038730/2020(H5N8) | 2020-2022 | West Europe | Domestic Galliformes | H5N8 |
| A/turkey/Poland/475/2020(H5N8) | 2020-2022 | East Europe | Domestic Galliformes | H5N8 |
| A/chicken/Netherlands/20019411-006010/2020(H5N8) | 2020-2022 | West Europe | Domestic Galliformes | H5N8 |
| A/chicken/Poland/476/2020(H5N8) | 2020-2022 | East Europe | Domestic Galliformes | H5N8 |
| A/common teal/Italy/20VIR7608-73/2020(H5N8) | 2020-2022 | West Europe | Wild Anseriformes | H5N8 |
| A/duck/Korea/H385/2020(H5N8) | 2020-2022 | Korea | Domestic Anseriformes | H5N8 |
| A/turkey/England/039352/2020(H5N8) | 2020-2022 | West Europe | Domestic Galliformes | H5N8 |
| A/turkey/England/039472/2020(H5N8) | 2020-2022 | West Europe | Domestic Galliformes | H5N8 |
| A/chicken/Nara/12T/2020(H5N8) | 2020-2022 | Japan | Domestic Galliformes | H5N8 |
| A/chicken/Nara/1C/2020(H5N8) | 2020-2022 | Japan | Domestic Galliformes | H5N8 |
| A/chicken/Nara/5T/2020(H5N8) | 2020-2022 | Japan | Domestic Galliformes | H5N8 |
| A/chicken/Nara/9T/2020(H5N8) | 2020-2022 | Japan | Domestic Galliformes | H5N8 |
| A/chicken/Hiroshima/1T/2020(H5N8) | 2020-2022 | Japan | Domestic Galliformes | H5N8 |
| A/chicken/Hiroshima/2T/2020(H5N8) | 2020-2022 | Japan | Domestic Galliformes | H5N8 |
| A/chicken/Hiroshima/3T/2020(H5N8) | 2020-2022 | Japan | Domestic Galliformes | H5N8 |
| A/chicken/Hiroshima/4T/2020(H5N8) | 2020-2022 | Japan | Domestic Galliformes | H5N8 |
| A/chicken/Korea/H390/2020(H5N8) | 2020-2022 | Korea | Domestic Galliformes | H5N8 |
| A/chicken/Netherlands/20019422-001005/2020(H5N8) | 2020-2022 | West Europe | Domestic Galliformes | H5N8 |
| A/chicken/Astrakhan/2171-1/2020 (H5N8) | 2020-2022 | East Europe | Domestic Galliformes | H5N8 |
| A/chicken/Miyazaki/D5C/2020(H5N8) | 2020-2022 | Japan | Domestic Galliformes | H5N8 |
| A/chicken/Miyazaki/D5T/2020(H5N8) | 2020-2022 | Japan | Domestic Galliformes | H5N8 |
| A/chicken/Miyazaki/E1T/2020(H5N8) | 2020-2022 | Japan | Domestic Galliformes | H5N8 |
| A/chicken/Miyazaki/E3T/2020(H5N8) | 2020-2022 | Japan | Domestic Galliformes | H5N8 |
| A/chicken/Miyazaki/E6T/2020(H5N8) | 2020-2022 | Japan | Domestic Galliformes | H5N8 |
| A/chicken/Miyazaki/E9T/2020(H5N8) | 2020-2022 | Japan | Domestic Galliformes | H5N8 |
| A/quail/Korea/H394/2020(H5N8) | 2020-2022 | Korea | Domestic Galliformes | H5N8 |
| A/turkey/Poland/477/2020(H5N8) | 2020-2022 | East Europe | Domestic Galliformes | H5N8 |
| A/greylag goose/Netherlands/20019685-002/2020(H5N1) | 2020-2022 | West Europe | Wild Anseriformes | H5N1 |
| A/quail/Korea/H412/2020(H5N8) | 2020-2022 | Korea | Domestic Galliformes | H5N8 |
| A/red fox/England/AVP-M1-21-01/2020(H5N8) | 2020-2022 | West Europe | Mammal | H5N8 |
| A/seal/England/AVP-031141/2020(H5N8) | 2020-2022 | West Europe | Mammal | H5N8 |
| A/tundra bean goose/Poland/MB128/2020(H5N8) | 2020-2022 | East Europe | Wild Anseriformes | H5N8 |
| A/chicken/Oita/27T/2020(H5N8) | 2020-2022 | Japan | Domestic Galliformes | H5N8 |
| A/chicken/Oita/2T/2020(H5N8) | 2020-2022 | Japan | Domestic Galliformes | H5N8 |
| A/chicken/Oita/3T/2020(H5N8) | 2020-2022 | Japan | Domestic Galliformes | H5N8 |
| A/chicken/Oita/5T/2020(H5N8) | 2020-2022 | Japan | Domestic Galliformes | H5N8 |
| A/chicken/Wakayama/1T/2020(H5N8) | 2020-2022 | Japan | Domestic Galliformes | H5N8 |
| A/chicken/Wakayama/2T/2020(H5N8) | 2020-2022 | Japan | Domestic Galliformes | H5N8 |
| A/chicken/Wakayama/3T/2020(H5N8) | 2020-2022 | Japan | Domestic Galliformes | H5N8 |
| A/chicken/Wakayama/4T/2020(H5N8) | 2020-2022 | Japan | Domestic Galliformes | H5N8 |
| A/duck/Korea/H419/2020(H5N8) | 2020-2022 | Korea | Domestic Anseriformes | H5N8 |
| A/mute swan/Slovenia/1799-20 21VIR959-2/2020(H5N8) | 2020-2022 | East Europe | Wild Anseriformes | H5N8 |
| A/tundra bean goose/Poland/MB132/2020(H5N8) | 2020-2022 | East Europe | Wild Anseriformes | H5N8 |
| A/chicken/Okayama/1T/2020(H5N8) | 2020-2022 | Japan | Domestic Galliformes | H5N8 |
| A/chicken/Okayama/2T/2020(H5N8) | 2020-2022 | Japan | Domestic Galliformes | H5N8 |
| A/chicken/Okayama/6T/2020(H5N8) | 2020-2022 | Japan | Domestic Galliformes | H5N8 |
| A/chicken/Okayama/7T/2020(H5N8) | 2020-2022 | Japan | Domestic Galliformes | H5N8 |
| A/duck/Korea/H431/2020(H5N8) | 2020-2022 | Korea | Domestic Anseriformes | H5N8 |
| A/duck/Korea/H432/2020(H5N8) | 2020-2022 | Korea | Domestic Anseriformes | H5N8 |
| A/mute swan/Slovenia/1820-20 21VIR959-4/2020(H5N8) | 2020-2022 | East Europe | Wild Anseriformes | H5N8 |
| A/wild goose/Germany-NI/AI03220/2020(H5N8) | 2020-2022 | West Europe | Wild Anseriformes | H5N8 |
| A/duck/Korea/H438/2020(H5N8) | 2020-2022 | Korea | Domestic Anseriformes | H5N8 |
| A/duck/Korea/H439/2020(H5N8) | 2020-2022 | Korea | Domestic Anseriformes | H5N8 |
| A/muscovy duck/Netherlands/20019914-001/2020(H5N8) | 2020-2022 | West Europe | Wild Anseriformes | H5N8 |
| A/turkey/Stavropol/320-01/2020(H5N8) | 2020-2022 | East Europe | Domestic Galliformes | H5N8 |
| A/turkey/Stavropol/320-02/2020(H5N8) | 2020-2022 | East Europe | Domestic Galliformes | H5N8 |
| A/turkey/Stavropol/320-03/2020(H5N8) | 2020-2022 | East Europe | Domestic Galliformes | H5N8 |
| A/Astrakhan/3212/2020(H5N8) | 2020-2022 | East Europe | Human | H5N8 |
| A/chicken/Astrakhan/321-01/2020(H5N8) | 2020-2022 | East Europe | Domestic Galliformes | H5N8 |
| A/chicken/Astrakhan/321-05/2020(H5N8) | 2020-2022 | East Europe | Domestic Galliformes | H5N8 |
| A/chicken/Astrakhan/321-06/2020(H5N8) | 2020-2022 | East Europe | Domestic Galliformes | H5N8 |
| A/chicken/Astrakhan/321-09/2020(H5N8) | 2020-2022 | East Europe | Domestic Galliformes | H5N8 |
| A/chicken/Astrakhan/321-10/2020(H5N8) | 2020-2022 | East Europe | Domestic Galliformes | H5N8 |
| A/chicken/Korea/H440/2020(H5N8) | 2020-2022 | Korea | Domestic Galliformes | H5N8 |
| A/chicken/Shiga/4T/2020(H5N8) | 2020-2022 | Japan | Domestic Galliformes | H5N8 |
| A/chicken/Shiga/5T/2020(H5N8) | 2020-2022 | Japan | Domestic Galliformes | H5N8 |
| A/chicken/Shiga/6T/2020(H5N8) | 2020-2022 | Japan | Domestic Galliformes | H5N8 |
| A/chicken/Shiga/7T/2020(H5N8) | 2020-2022 | Japan | Domestic Galliformes | H5N8 |
| A/chicken/Kagawa/K2T/2020(H5N8) | 2020-2022 | Japan | Domestic Galliformes | H5N8 |
| A/chicken/Kagawa/K3T/2020(H5N8) | 2020-2022 | Japan | Domestic Galliformes | H5N8 |
| A/chicken/Kagawa/K4T/2020(H5N8) | 2020-2022 | Japan | Domestic Galliformes | H5N8 |
| A/chicken/Kagawa/K6T/2020(H5N8) | 2020-2022 | Japan | Domestic Galliformes | H5N8 |
| A/chicken/Miyazaki/F1T/2020(H5N8) | 2020-2022 | Japan | Domestic Galliformes | H5N8 |
| A/chicken/Miyazaki/F4T/2020(H5N8) | 2020-2022 | Japan | Domestic Galliformes | H5N8 |
| A/chicken/Miyazaki/F5T/2020(H5N8) | 2020-2022 | Japan | Domestic Galliformes | H5N8 |
| A/chicken/Miyazaki/F7T/2020(H5N8) | 2020-2022 | Japan | Domestic Galliformes | H5N8 |
| A/chicken/Miyazaki/G1T/2020(H5N8) | 2020-2022 | Japan | Domestic Galliformes | H5N8 |
| A/chicken/Miyazaki/G3T/2020(H5N8) | 2020-2022 | Japan | Domestic Galliformes | H5N8 |
| A/chicken/Miyazaki/G5T/2020(H5N8) | 2020-2022 | Japan | Domestic Galliformes | H5N8 |
| A/chicken/Miyazaki/G9T/2020(H5N8) | 2020-2022 | Japan | Domestic Galliformes | H5N8 |
| A/peregrine falcon/Netherlands/20020038-001/2020(H5N8) | 2020-2022 | West Europe | Other wild species | H5N8 |
| A/chicken/Korea/H441/2020(H5N8) | 2020-2022 | Korea | Domestic Galliformes | H5N8 |
| A/chicken/Korea/H450/2020(H5N8) | 2020-2022 | Korea | Domestic Galliformes | H5N8 |
| A/chicken/Netherlands/20019879-001005/2020(H5N1) | 2020-2022 | West Europe | Domestic Galliformes | H5N1 |
| A/falcon/England/041976/2020(H5N8) | 2020-2022 | West Europe | Other wild species | H5N8 |
| A/goose/Korea/H449/2020(H5N8) | 2020-2022 | Korea | Domestic Anseriformes | H5N8 |
| A/red knot/Germany-SH/AI03419/2020(H5N3) | 2020-2022 | West Europe | Charadriiformes | H5N3 |
| A/red knot/Germany-SH/AI03421/2020(H5N3) | 2020-2022 | West Europe | Charadriiformes | H5N3 |
| A/wild bird/Korea/H467/2020(H5N8) | 2020-2022 | Korea | Other wild species | H5N8 |
| A/wild goose/Germany-NI/AI03471/2020(H5N8) | 2020-2022 | West Europe | Wild Anseriformes | H5N8 |
| A/chicken/Kagawa/L4T/2020(H5N8) | 2020-2022 | Japan | Domestic Galliformes | H5N8 |
| A/chicken/Kagawa/L6T/2020(H5N8) | 2020-2022 | Japan | Domestic Galliformes | H5N8 |
| A/chicken/Kagawa/L7T/2020(H5N8) | 2020-2022 | Japan | Domestic Galliformes | H5N8 |
| A/chicken/Kagawa/L9T/2020(H5N8) | 2020-2022 | Japan | Domestic Galliformes | H5N8 |
| A/chicken/Kochi/4C/2020(H5N8) | 2020-2022 | Japan | Domestic Galliformes | H5N8 |
| A/chicken/Kochi/5C/2020(H5N8) | 2020-2022 | Japan | Domestic Galliformes | H5N8 |
| A/chicken/Kochi/6T/2020(H5N8) | 2020-2022 | Japan | Domestic Galliformes | H5N8 |
| A/chicken/Kochi/7T/2020(H5N8) | 2020-2022 | Japan | Domestic Galliformes | H5N8 |
| A/mute swan/Netherlands/20020133-001/2020(H5N8) | 2020-2022 | West Europe | Wild Anseriformes | H5N8 |
| A/spot-billed duck/Korea/WA1000/2020(H5N8) | 2020-2022 | Korea | Wild Anseriformes | H5N8 |
| A/wild goose/Poland/MB142/2020(H5N8) | 2020-2022 | East Europe | Wild Anseriformes | H5N8 |
| A/chicken/Korea/H470/2020(H5N8) | 2020-2022 | Korea | Domestic Galliformes | H5N8 |
| A/chicken/Scotland/043405/2020(H5N8) | 2020-2022 | West Europe | Domestic Galliformes | H5N8 |
| A/duck/Korea/H471/2020(H5N8) | 2020-2022 | Korea | Domestic Anseriformes | H5N8 |
| A/red knot/Germany-SH/AI03424/2020(H5N3) | 2020-2022 | West Europe | Charadriiformes | H5N3 |
| A/swan/Poland/MB141/2020(H5N8) | 2020-2022 | East Europe | Wild Anseriformes | H5N8 |
| A/wild bird/Korea/H496-3/2020(H5N8) | 2020-2022 | Korea | Other wild species | H5N8 |
| A/wild duck/Germany-NI/AI03479/2020(H5N8) | 2020-2022 | West Europe | Wild Anseriformes | H5N8 |
| A/black-headed gull/Netherlands/20020162-002/2020(H5N8) | 2020-2022 | West Europe | Charadriiformes | H5N1 |
| A/chicken/England/043683/2020(H5N8) | 2020-2022 | West Europe | Domestic Galliformes | H5N8 |
| A/common kestrel/Netherlands/20020264-002/2020(H5N8) | 2020-2022 | West Europe | Other wild species | H5N8 |
| A/wild goose/Germany-NI/AI03473/2020(H5N8) | 2020-2022 | West Europe | Wild Anseriformes | H5N8 |
| A/chicken/Miyazaki/H1T/2020(H5N8) | 2020-2022 | Japan | Domestic Galliformes | H5N8 |
| A/chicken/Miyazaki/H3T/2020(H5N8) | 2020-2022 | Japan | Domestic Galliformes | H5N8 |
| A/chicken/Miyazaki/H6T/2020(H5N8) | 2020-2022 | Japan | Domestic Galliformes | H5N8 |
| A/chicken/Miyazaki/H9T/2020(H5N8) | 2020-2022 | Japan | Domestic Galliformes | H5N8 |
| A/chicken/Tokushima/1T/20200(H5N8) | 2020-2022 | Japan | Domestic Galliformes | H5N8 |
| A/chicken/Tokushima/2T/2020(H5N8) | 2020-2022 | Japan | Domestic Galliformes | H5N8 |
| A/chicken/Tokushima/3T/2020(H5N8) | 2020-2022 | Japan | Domestic Galliformes | H5N8 |
| A/chicken/Tokushima/4T/2020(H5N8) | 2020-2022 | Japan | Domestic Galliformes | H5N8 |
| A/duck/England/043628/2020(H5N8) | 2020-2022 | West Europe | Domestic Anseriformes | H5N8 |
| A/duck/Korea/H499/2020(H5N8) | 2020-2022 | Korea | Domestic Anseriformes | H5N8 |
| A/turkey/Germany-NI/AI03432/2020 (H5N8) | 2020-2022 | West Europe | Domestic Galliformes | H5N8 |
| A/chicken/Korea/H491/2020(H5N8) | 2020-2022 | Korea | Domestic Galliformes | H5N8 |
| A/duck/Northern China/LSP/2020(H5N8) | 2020-2022 | China | Domestic Anseriformes | H5N8 |
| A/kestrel/Germany-NI/AI03672/2020(H5N8) | 2020-2022 | West Europe | Other wild species | H5N8 |
| A/turkey/Germany-NI/AI03452/2020 (H5N8) | 2020-2022 | West Europe | Domestic Galliformes | H5N8 |
| A/turkey/Germany-NI/AI03453/2020(H5N8) | 2020-2022 | West Europe | Domestic Galliformes | H5N8 |
| A/turkey/Poland/542/2020(H5N8) | 2020-2022 | East Europe | Domestic Galliformes | H5N8 |
| A/chicken/Kagawa/M10T/2020(H5N8) | 2020-2022 | Japan | Domestic Galliformes | H5N8 |
| A/chicken/Kagawa/M11T/2020(H5N8) | 2020-2022 | Japan | Domestic Galliformes | H5N8 |
| A/chicken/Kagawa/M12T/2020(H5N8) | 2020-2022 | Japan | Domestic Galliformes | H5N8 |
| A/chicken/Kagawa/M2T/2020(H5N8) | 2020-2022 | Japan | Domestic Galliformes | H5N8 |
| A/chicken/Korea/H510/2020(H5N8) | 2020-2022 | Korea | Domestic Galliformes | H5N8 |
| A/duck/Korea/H509/2020(H5N8) | 2020-2022 | Korea | Domestic Anseriformes | H5N8 |
| A/duck/Korea/H511/2020(H5N8) | 2020-2022 | Korea | Domestic Anseriformes | H5N8 |
| A/Mandarin duck/Kagoshima/KU-d57/2020 (H5N8) | 2020-2022 | Japan | Wild Anseriformes | H5N8 |
| A/chicken/Chiba/1T/2020(H5N8) | 2020-2022 | Japan | Domestic Galliformes | H5N8 |
| A/chicken/Chiba/2T/2020(H5N8) | 2020-2022 | Japan | Domestic Galliformes | H5N8 |
| A/chicken/Chiba/3T/2020(H5N8) | 2020-2022 | Japan | Domestic Galliformes | H5N8 |
| A/chicken/Chiba/4T/2020(H5N8) | 2020-2022 | Japan | Domestic Galliformes | H5N8 |
| A/chicken/Egypt/CAI11/2020(H5N8) | 2020-2022 | Africa | Domestic Galliformes | H5N8 |
| A/chicken/Senegal/21VIR1084-3/2021(H5N1) | 2020-2022 | Africa | Domestic Galliformes | H5N1 |
| A/chicken/Senegal/21VIR1084-4/2021(H5N1) | 2020-2022 | Africa | Domestic Galliformes | H5N1 |
| A/chicken/Senegal/21VIR1084-5/2021(H5N1) | 2020-2022 | Africa | Domestic Galliformes | H5N1 |
| A/duck/Korea/H514/2020(H5N8) | 2020-2022 | Korea | Domestic Anseriformes | H5N8 |
| A/duck/Korea/H515/2020(H5N8) | 2020-2022 | Korea | Domestic Anseriformes | H5N8 |
| A/duck/Korea/H516/2020(H5N8) | 2020-2022 | Korea | Domestic Anseriformes | H5N8 |
| A/turkey/Germany-NI/AI03584/2020 (H5N8) | 2020-2022 | West Europe | Domestic Galliformes | H5N8 |
| A/duck/Korea/H524/2020(H5N8) | 2020-2022 | Korea | Domestic Anseriformes | H5N8 |
| A/mute swan/Slovenia/1914-20 21VIR959-5/2020(H5N5) | 2020-2022 | East Europe | Wild Anseriformes | H5N5 |
| A/chicken/England/045984/2020(H5N8) | 2020-2022 | West Europe | Domestic Galliformes | H5N8 |
| A/chicken/Korea/H525/2020(H5N8) | 2020-2022 | Korea | Domestic Galliformes | H5N8 |
| A/chicken/Korea/H526/2020(H5N8) | 2020-2022 | Korea | Domestic Galliformes | H5N8 |
| A/turkey/Germany-NI/AI03594/2020 (H5N8) | 2020-2022 | West Europe | Domestic Galliformes | H5N8 |
| A/Anser brachyrhynchus/Belgium/151/2020(H5N8) | 2020-2022 | West Europe | Wild Anseriformes | H5N8 |
| A/turkey/Germany-NI/AI03599/2020(H5N8) | 2020-2022 | West Europe | Domestic Galliformes | H5N8 |
| A/turkey/Germany-NI/AI03606/2020 (H5N8) | 2020-2022 | West Europe | Domestic Galliformes | H5N8 |
| A/turkey/Germany-NI/AI03609/2020(H5N8) | 2020-2022 | West Europe | Domestic Galliformes | H5N8 |
| A/duck/England/046311/2020(H5N8) | 2020-2022 | West Europe | Domestic Anseriformes | H5N8 |
| A/duck/Korea/H528/2020(H5N8) | 2020-2022 | Korea | Domestic Anseriformes | H5N8 |
| A/chicken/England/046491/2020(H5N8) | 2020-2022 | West Europe | Domestic Galliformes | H5N8 |
| A/chicken/Korea/H531/2020(H5N8) | 2020-2022 | Korea | Domestic Galliformes | H5N8 |
| A/chicken/Korea/H532/2020(H5N8) | 2020-2022 | Korea | Domestic Galliformes | H5N8 |
| A/chicken/Poland/565/2020(H5N8) | 2020-2022 | East Europe | Domestic Galliformes | H5N8 |
| A/duck/Korea/H007/2020(H5N8) | 2020-2022 | Korea | Domestic Anseriformes | H5N8 |
| A/duck/Korea/H538/2020(H5N8) | 2020-2022 | Korea | Domestic Anseriformes | H5N8 |
| A/White peacock/Korea/H533/2020(H5N8) | 2020-2022 | Korea | Other wild species | H5N8 |
| A/chicken/Korea/H541/2020(H5N8) | 2020-2022 | Korea | Domestic Galliformes | H5N8 |
| A/chicken/Korea/H544/2020(H5N8) | 2020-2022 | Korea | Domestic Galliformes | H5N8 |
| A/chicken/Miyazaki/I11T/2020(H5N8) | 2020-2022 | Japan | Domestic Galliformes | H5N8 |
| A/chicken/Miyazaki/I12T/2020(H5N8) | 2020-2022 | Japan | Domestic Galliformes | H5N8 |
| A/chicken/Miyazaki/I5T/2020(H5N8) | 2020-2022 | Japan | Domestic Galliformes | H5N8 |
| A/chicken/Miyazaki/I9T/2020(H5N8) | 2020-2022 | Japan | Domestic Galliformes | H5N8 |
| A/duck/Korea/H542/2020(H5N8) | 2020-2022 | Korea | Domestic Anseriformes | H5N8 |
| A/duck/Northern China/ZGL/2020(H5N8) | 2020-2022 | China | Domestic Anseriformes | H5N8 |
| A/mallard/Georgia/DT-20222/2020(H5N8) | 2020-2022 | West Central Asia | Wild Anseriformes | H5N8 |
| A/turkey/Germany-NI/AI03654/2020(H5N8) | 2020-2022 | West Europe | Domestic Galliformes | H5N8 |
| A/duck/Korea/H548/2020(H5N8) | 2020-2022 | Korea | Domestic Anseriformes | H5N8 |
| A/duck/Korea/H549/2020(H5N8) | 2020-2022 | Korea | Domestic Anseriformes | H5N8 |
| A/red knot/Germany-NI/AI00394/2020(H5N3) | 2020-2022 | West Europe | Charadriiformes | H5N3 |
| A/chicken/Korea/H550/2020(H5N8) | 2020-2022 | Korea | Domestic Galliformes | H5N8 |
| A/chicken/Northern Ireland/2020-17671 21VIR113-11/2020(H5N8) | 2020-2022 | West Europe | Domestic Galliformes | H5N8 |
| A/duck/Southwestern China/B1904/2020(H5N8) | 2020-2022 | China | Domestic Anseriformes | H5N8 |
| A/mute swan/North Ossetia-Alania/325-01/2020(H5N8) | 2020-2022 | East Europe | Wild Anseriformes | H5N8 |
| A/mute swan/North Ossetia-Alania/325-02/2020(H5N8) | 2020-2022 | East Europe | Wild Anseriformes | H5N8 |
| A/mute swan/North Ossetia-Alania/325-03/2020(H5N8) | 2020-2022 | East Europe | Wild Anseriformes | H5N8 |
| A/quail/Korea/H551/2020(H5N8) | 2020-2022 | Korea | Domestic Galliformes | H5N8 |
| A/turkey/Germany-NI/AI00016/2021(H5N8) | 2020-2022 | West Europe | Domestic Galliformes | H5N8 |
| A/chicken/Gifu/10T/2021(H5N8) | 2020-2022 | Japan | Domestic Galliformes | H5N8 |
| A/chicken/Gifu/11T/2021(H5N8) | 2020-2022 | Japan | Domestic Galliformes | H5N8 |
| A/chicken/Gifu/2T/2021(H5N8) | 2020-2022 | Japan | Domestic Galliformes | H5N8 |
| A/chicken/Gifu/3T/2021(H5N8) | 2020-2022 | Japan | Domestic Galliformes | H5N8 |
| A/chicken/Gifu/6T/2021(H5N8) | 2020-2022 | Japan | Domestic Galliformes | H5N8 |
| A/chicken/Gifu/7T/2021(H5N8) | 2020-2022 | Japan | Domestic Galliformes | H5N8 |
| A/chicken/Korea/H001/2021(H5N8) | 2020-2022 | Korea | Domestic Galliformes | H5N8 |
| A/common kestrel/Denmark/16023-01/2021-01-01(H5N3) | 2020-2022 | West Europe | Other wild species | H5N3 |
| A/knot wader/Ireland/472 21VIR2956-1/2021(H5N3) | 2020-2022 | West Europe | Charadriiformes | H5N3 |
| A/knot wader/Ireland/473 21VIR2956-2/2021(H5N3) | 2020-2022 | West Europe | Charadriiformes | H5N3 |
| A/mute swan/Finland/1325 21VIR7689-2/2021(H5N8) | 2020-2022 | West Europe | Wild Anseriformes | H5N8 |
| A/pheasant/Finland/499 21VIR7689-1/2021(H5N8) | 2020-2022 | West Europe | Other wild species | H5N8 |
| A/turkey/Germany-NI/AI00038/2021(H5N8) | 2020-2022 | West Europe | Domestic Galliformes | H5N8 |
| A/turkey/Germany-NI/AI00042/2021(H5N8) | 2020-2022 | West Europe | Domestic Galliformes | H5N8 |
| A/chicken/Korea/H002/2021(H5N8) | 2020-2022 | Korea | Domestic Galliformes | H5N8 |
| A/chicken/Korea/H008/2021(H5N8) | 2020-2022 | Korea | Domestic Galliformes | H5N8 |
| A/common buzzard/Netherlands/21021023-002/2021(H5N3) | 2020-2022 | West Europe | Other wild species | H5N8 |
| A/common buzzard/Netherlands/21021187-001/2021(H5N8) | 2020-2022 | West Europe | Other wild species | H5N3 |
| A/domestic duck/Germany-NI/AI00079/2021(H5N8) | 2020-2022 | West Europe | Domestic Anseriformes | H5N8 |
| A/duck/Korea/H009/2021(H5N8) | 2020-2022 | Korea | Domestic Anseriformes | H5N8 |
| A/turkey/Germany-NI/AI00063/2021(H5N8) | 2020-2022 | West Europe | Domestic Galliformes | H5N8 |
| A/turkey/Germany-NI/AI00072/2021(H5N8) | 2020-2022 | West Europe | Domestic Galliformes | H5N8 |
| A/turkey/Netherlands/21020942-001005/2021(H5N8) | 2020-2022 | West Europe | Domestic Galliformes | H5N8 |
| A/chicken/Krasnodar/334-01/2021(H5N8) | 2020-2022 | East Europe | Domestic Galliformes | H5N8 |
| A/chicken/Krasnodar/334-02/2021(H5N8) | 2020-2022 | East Europe | Domestic Galliformes | H5N8 |
| A/chicken/Krasnodar/334-03/2021(H5N8) | 2020-2022 | East Europe | Domestic Galliformes | H5N8 |
| A/chicken/Northern Ireland/2021-000067 21VIR114-19/2021(H5N8) | 2020-2022 | West Europe | Domestic Galliformes | H5N8 |
| A/common buzzard/Netherlands/21021278-002/2021(H5N8) | 2020-2022 | West Europe | Other wild species | H5N8 |
| A/duck/Korea/H010/2021(H5N8) | 2020-2022 | Korea | Domestic Anseriformes | H5N8 |
| A/mute swan/Norway/FU5 21VIR850-3/2021(H5N8) | 2020-2022 | West Europe | Wild Anseriformes | H5N8 |
| A/duck/Korea/H016/2021(H5N8) | 2020-2022 | Korea | Domestic Anseriformes | H5N8 |
| A/turkey/Germany-NI/AI00402/2021(H5N8) | 2020-2022 | West Europe | Domestic Galliformes | H5N8 |
| A/turkey/Germany-NI/AI00406/2021(H5N8) | 2020-2022 | West Europe | Domestic Galliformes | H5N8 |
| A/anser anser/Spain/102-8 21VIR1230-4/2021(H5N8) | 2020-2022 | West Europe | Wild Anseriformes | H5N8 |
| A/ciconia ciconia/Spain/102-1 21VIR1230-2/2021(H5N8) | 2020-2022 | West Europe | Other wild species | H5N8 |
| A/chicken/Korea/H022/2021(H5N8) | 2020-2022 | Korea | Domestic Galliformes | H5N8 |
| A/common kestrel/Netherlands/21021301-039/2021(H5N8) | 2020-2022 | West Europe | Other wild species | H5N8 |
| A/muscovy duck/Slovakia/Pah1 21VIR1086-1/2021(H5N8) | 2020-2022 | East Europe | Wild Anseriformes | H5N8 |
| A/turkey/Germany-NI/AI00429/2021(H5N8) | 2020-2022 | West Europe | Domestic Galliformes | H5N8 |
| A/whooper swan/Romania/10122 21VIR2593-23/2021(H5N5) | 2020-2022 | East Europe | Wild Anseriformes | H5N5 |
| A/whooper swan/Romania/10123 21VIR849-1/2021(H5N5) | 2020-2022 | East Europe | Wild Anseriformes | H5N5 |
| A/common buzzard/Netherlands/21021396-002/2021(H5N1) | 2020-2022 | West Europe | Other wild species | H5N8 |
| A/duck/Korea/H025/2021(H5N8) | 2020-2022 | Korea | Domestic Anseriformes | H5N8 |
| A/chicken/Chiba/B2T/2021(H5N8) | 2020-2022 | Japan | Domestic Galliformes | H5N8 |
| A/chicken/Chiba/B3T/2021(H5N8) | 2020-2022 | Japan | Domestic Galliformes | H5N8 |
| A/chicken/Chiba/B4T/2021(H5N8) | 2020-2022 | Japan | Domestic Galliformes | H5N8 |
| A/chicken/Chiba/B5T/2021(H5N8) | 2020-2022 | Japan | Domestic Galliformes | H5N8 |
| A/common buzzard/Netherlands/21021497-001/2021(H5N8) | 2020-2022 | West Europe | Other wild species | H5N1 |
| A/Branta canadensis/Belgium/500/2021(H5N8) | 2020-2022 | West Europe | Wild Anseriformes | H5N8 |
| A/turkey/Germany-NI/AI00439/2021(H5N8) | 2020-2022 | West Europe | Domestic Galliformes | H5N8 |
| A/wild goose/Germany-NI/AI00626/2021(H5N8) | 2020-2022 | West Europe | Wild Anseriformes | H5N8 |
| A/chicken/Germany-MV/AI00539/2021(H5N8) | 2020-2022 | West Europe | Domestic Galliformes | H5N8 |
| A/chicken/Germany-NI/AI00547/2021(H5N8) | 2020-2022 | West Europe | Domestic Galliformes | H5N8 |
| A/chicken/Kagoshima/1T/2021(H5N8) | 2020-2022 | Japan | Domestic Galliformes | H5N8 |
| A/chicken/Kagoshima/3T/2021(H5N8) | 2020-2022 | Japan | Domestic Galliformes | H5N8 |
| A/chicken/Kagoshima/5T/2021(H5N8) | 2020-2022 | Japan | Domestic Galliformes | H5N8 |
| A/chicken/Kagoshima/6T/2021(H5N8) | 2020-2022 | Japan | Domestic Galliformes | H5N8 |
| A/turkey/Germany-NI/AI00589/2021(H5N8) | 2020-2022 | West Europe | Domestic Galliformes | H5N8 |
| A/whooper swan/Romania/10171 21VIR2593-15/2021(H5N5) | 2020-2022 | East Europe | Wild Anseriformes | H5N5 |
| A/wild goose/Germany-NI/AI00625/2021(H5N8) | 2020-2022 | West Europe | Wild Anseriformes | H5N8 |
| A/duck/Romania/10202 21VIR2593-31/2021(H5N8) | 2020-2022 | East Europe | Domestic Anseriformes | H5N8 |
| A/duck/Romania/10205-t4 21VIR2593-3/2021(H5N8) | 2020-2022 | East Europe | Domestic Anseriformes | H5N8 |
| A/duck/Romania/10206 21VIR849-4/2021(H5N8) | 2020-2022 | East Europe | Domestic Anseriformes | H5N8 |
| A/geese/Romania/10206 21VIR849-3/2021(H5N8) | 2020-2022 | East Europe | Domestic Anseriformes | H5N8 |
| A/goose/Romania/10205-t3 21VIR2593-7/2021(H5N8) | 2020-2022 | East Europe | Domestic Anseriformes | H5N8 |
| A/goose/Romania/10205-t5 21VIR2593-8/2021(H5N8) | 2020-2022 | East Europe | Domestic Anseriformes | H5N8 |
| A/barnacle goose/Netherlands/21021591-001/2021(H5N1) | 2020-2022 | West Europe | Wild Anseriformes | H5N1 |
| A/mute swan/Shandong/1/2021(H5N8) | 2020-2022 | China | Wild Anseriformes | H5N8 |
| A/turkey/Germany-NI/AI00591/2021 (H5N8) | 2020-2022 | West Europe | Domestic Galliformes | H5N8 |
| A/whooper swan/Romania/10213 21VIR2593-27/2021(H5N5) | 2020-2022 | East Europe | Wild Anseriformes | H5N5 |
| A/mute swan/Slovakia/Pah6 21VIR1086-2/2021(H5N5) | 2020-2022 | East Europe | Wild Anseriformes | H5N5 |
| A/turkey/Germany-NI/AI00612/2021 (H5N8) | 2020-2022 | West Europe | Domestic Galliformes | H5N8 |
| A/turkey/Germany-NI/AI00616/2021(H5N8) | 2020-2022 | West Europe | Domestic Galliformes | H5N8 |
| A/mallard/Kagoshima/KU-d89/2021(H5N8) | 2020-2022 | Japan | Wild Anseriformes | H5N8 |
| A/turkey/Germany-NI/AI00621/2021(H5N8) | 2020-2022 | West Europe | Domestic Galliformes | H5N8 |
| A/Hawaiian goose/Germany-RP/AI00856/2021(H5N8) | 2020-2022 | West Europe | Wild Anseriformes | H5N8 |
| A/red knot/France/21P003249/2021(H5N3) | 2020-2022 | West Europe | Charadriiformes | H5N3 |
| A/sanderling/Netherlands/21021794-002/2021(H5N8) | 2020-2022 | West Europe | Charadriiformes | H5N1 |
| A/wild goose/Germany-NI/AI00854/2021(H5N8) | 2020-2022 | West Europe | Wild Anseriformes | H5N8 |
| A/barnacle goose/Netherlands/21022039-002/2021(H5N8) | 2020-2022 | West Europe | Wild Anseriformes | H5N8 |
| A/crane/Kagoshima/KU-93/2021(H5N8) | 2020-2022 | Japan | Other wild species | H5N8 |
| A/domestic duck/Germany-NI/AI00646/2021(H5N8) | 2020-2022 | West Europe | Domestic Anseriformes | H5N8 |
| A/goose/Romania/10330 21VIR2593-35/2021(H5N8) | 2020-2022 | East Europe | Domestic Anseriformes | H5N8 |
| A/house sparrow/Romania/10327 21VIR849-5/2021(H5N8) | 2020-2022 | East Europe | Other wild species | H5N8 |
| A/mute swan/Czech Republic/1410-2/2021(H5N8) | 2020-2022 | East Europe | Wild Anseriformes | H5N8 |
| A/duck/Chiba/C1T/2021(H5N8) | 2020-2022 | Japan | Domestic Anseriformes | H5N8 |
| A/duck/Chiba/C2T/2021(H5N8) | 2020-2022 | Japan | Domestic Anseriformes | H5N8 |
| A/duck/Chiba/C3T/2021(H5N8) | 2020-2022 | Japan | Domestic Anseriformes | H5N8 |
| A/duck/Chiba/C5T/2021(H5N8) | 2020-2022 | Japan | Domestic Anseriformes | H5N8 |
| A/green-winged teal/Guangdong/SD004/2021(H5N8) | 2020-2022 | China | Wild Anseriformes | H5N8 |
| A/mallard/Germany-MV/AI00639/2021(H5N8) | 2020-2022 | West Europe | Wild Anseriformes | H5N8 |
| A/turkey/Germany-NI/AI00647/2021(H5N8) | 2020-2022 | West Europe | Domestic Galliformes | H5N8 |
| A/whooper swan/Romania/10311 21VIR849-2/2021(H5N5) | 2020-2022 | East Europe | Wild Anseriformes | H5N5 |
| A/wild goose/Germany-NI/AI00855/2021(H5N8) | 2020-2022 | West Europe | Wild Anseriformes | H5N8 |
| A/chicken/Czech Republic/1566-1/2021(H5N8) | 2020-2022 | East Europe | Domestic Galliformes | H5N8 |
| A/chicken/Czech Republic/1566-2/2021(H5N8) | 2020-2022 | East Europe | Domestic Galliformes | H5N8 |
| A/chicken/Germany-NI/AI00887/2021(H5N8) | 2020-2022 | West Europe | Domestic Galliformes | H5N8 |
| A/chicken/Slovakia/Pah10 21VIR1086-5/2021(H5N5) | 2020-2022 | East Europe | Domestic Galliformes | H5N5 |
| A/chicken/Toyama/1T/2021(H5N8) | 2020-2022 | Japan | Domestic Galliformes | H5N8 |
| A/chicken/Toyama/2T/2021(H5N8) | 2020-2022 | Japan | Domestic Galliformes | H5N8 |
| A/chicken/Toyama/3T/2021(H5N8) | 2020-2022 | Japan | Domestic Galliformes | H5N8 |
| A/chicken/Toyama/4T/2021(H5N8) | 2020-2022 | Japan | Domestic Galliformes | H5N8 |
| A/curlew/France/21P003648/2021(H5N3) | 2020-2022 | West Europe | Charadriiformes | H5N3 |
| A/hawk/Romania/10363 21VIR2593-11/2021(H5N8) | 2020-2022 | East Europe | Other wild species | H5N8 |
| A/mute swan/Czech Republic/1410-1/2021(H5N8) | 2020-2022 | East Europe | Wild Anseriformes | H5N8 |
| A/turkey/Germany-BB/AI00868/2021(H5N8) | 2020-2022 | West Europe | Domestic Galliformes | H5N8 |
| A/whooper swan/Romania/10362 21VIR2593-20/2021(H5N5) | 2020-2022 | East Europe | Wild Anseriformes | H5N5 |
| A/common coot/Shandong/SC197/2021(H5N8) | 2020-2022 | China | Other wild species | H5N8 |
| A/duck/Chiba/D1-3T/2021(H5N8) | 2020-2022 | Japan | Domestic Anseriformes | H5N8 |
| A/duck/Chiba/D1A-10T/2021(H5N8) | 2020-2022 | Japan | Domestic Anseriformes | H5N8 |
| A/duck/Chiba/D1A-1T/2021(H5N8) | 2020-2022 | Japan | Domestic Anseriformes | H5N8 |
| A/duck/Chiba/D1A-2T/2021(H5N8) | 2020-2022 | Japan | Domestic Anseriformes | H5N8 |
| A/duck/Chiba/D1A-3T/2021(H5N8) | 2020-2022 | Japan | Domestic Anseriformes | H5N8 |
| A/duck/Chiba/D1A-4T/2021(H5N8) | 2020-2022 | Japan | Domestic Anseriformes | H5N8 |
| A/duck/Chiba/D1A-5T/2021(H5N8) | 2020-2022 | Japan | Domestic Anseriformes | H5N8 |
| A/duck/Chiba/D1A-6T/2021(H5N8) | 2020-2022 | Japan | Domestic Anseriformes | H5N8 |
| A/duck/Chiba/D1A-7T/2021(H5N8) | 2020-2022 | Japan | Domestic Anseriformes | H5N8 |
| A/duck/Chiba/D1A-8T/2021(H5N8) | 2020-2022 | Japan | Domestic Anseriformes | H5N8 |
| A/duck/Chiba/D1A-9T/2021(H5N8) | 2020-2022 | Japan | Domestic Anseriformes | H5N8 |
| A/duck/Chiba/D1B-10T/2021(H5N8) | 2020-2022 | Japan | Domestic Anseriformes | H5N8 |
| A/duck/Chiba/D1B-1T/2021(H5N8) | 2020-2022 | Japan | Domestic Anseriformes | H5N8 |
| A/duck/Chiba/D1B-2T/2021(H5N8) | 2020-2022 | Japan | Domestic Anseriformes | H5N8 |
| A/duck/Chiba/D1B-3T/2021(H5N8) | 2020-2022 | Japan | Domestic Anseriformes | H5N8 |
| A/duck/Chiba/D1B-4T/2021(H5N8) | 2020-2022 | Japan | Domestic Anseriformes | H5N8 |
| A/duck/Chiba/D1B-5T/2021(H5N8) | 2020-2022 | Japan | Domestic Anseriformes | H5N8 |
| A/duck/Chiba/D1B-6T/2021(H5N8) | 2020-2022 | Japan | Domestic Anseriformes | H5N8 |
| A/duck/Chiba/D1B-7T/2021(H5N8) | 2020-2022 | Japan | Domestic Anseriformes | H5N8 |
| A/duck/Chiba/D1B-8T/2021(H5N8) | 2020-2022 | Japan | Domestic Anseriformes | H5N8 |
| A/duck/Chiba/D1B-9T/2021(H5N8) | 2020-2022 | Japan | Domestic Anseriformes | H5N8 |
| A/duck/Chiba/D2A-10T/2021(H5N8) | 2020-2022 | Japan | Domestic Anseriformes | H5N8 |
| A/duck/Chiba/D2A-1T/2021(H5N8) | 2020-2022 | Japan | Domestic Anseriformes | H5N8 |
| A/duck/Chiba/D2A-2T/2021(H5N8) | 2020-2022 | Japan | Domestic Anseriformes | H5N8 |
| A/duck/Chiba/D2A-3T/2021(H5N8) | 2020-2022 | Japan | Domestic Anseriformes | H5N8 |
| A/duck/Chiba/D2A-5T/2021(H5N8) | 2020-2022 | Japan | Domestic Anseriformes | H5N8 |
| A/duck/Chiba/D2A-7T/2021(H5N8) | 2020-2022 | Japan | Domestic Anseriformes | H5N8 |
| A/duck/Chiba/D2A-8C/2021(H5N8) | 2020-2022 | Japan | Domestic Anseriformes | H5N8 |
| A/duck/Chiba/D2A-9T/2021(H5N8) | 2020-2022 | Japan | Domestic Anseriformes | H5N8 |
| A/duck/Chiba/D2B-7C/2021(H5N8) | 2020-2022 | Japan | Domestic Anseriformes | H5N8 |
| A/duck/Chiba/D2B-9C/2021(H5N8) | 2020-2022 | Japan | Domestic Anseriformes | H5N8 |
| A/great-white pelican/Senegal/21-67 21VIR1084-8/2021(H5N1) | 2020-2022 | Africa | Other wild species | H5N1 |
| A/whooper swan/Shandong/SC195/2021(H5N8) | 2020-2022 | China | Wild Anseriformes | H5N8 |
| A/whooper swan/Shandong/SC198/2021(H5N8) | 2020-2022 | China | Wild Anseriformes | H5N8 |
| A/whooper swan/Shandong/SC199/2021(H5N8) | 2020-2022 | China | Wild Anseriformes | H5N8 |
| A/whooper swan/Shandong/SC200/2021(H5N8) | 2020-2022 | China | Wild Anseriformes | H5N8 |
| A/wild goose/Shandong/SC196/2021(H5N8) | 2020-2022 | China | Wild Anseriformes | H5N8 |
| A/mute swan/Czech Republic/1656-1/2021(H5N8) | 2020-2022 | East Europe | Wild Anseriformes | H5N8 |
| A/black swan/Beijing/1/2021(H5N8) | 2020-2022 | China | Wild Anseriformes | H5N8 |
| A/gadwall/Netherlands/21024401-002/2021(H5N8) | 2020-2022 | West Europe | Wild Anseriformes | H5N1 |
| A/peregrine falcon/Northern Ireland/21VIR1662-3/2021(H5N3) | 2020-2022 | West Europe | Other wild species | H5N3 |
| A/egret/Jiangsu/SD021/2021(H5N8) | 2020-2022 | China | Other wild species | H5N8 |
| A/mute swan/Norway/FU48/2021(H5N8) | 2020-2022 | West Europe | Wild Anseriformes | H5N8 |
| A/turkey/Germany-MV/AI00909/2021(H5N8) | 2020-2022 | West Europe | Domestic Galliformes | H5N8 |
| A/wild duck/Jiangsu/SD019/2021(H5N8) | 2020-2022 | China | Wild Anseriformes | H5N8 |
| A/anser anser/Spain/297-1 21VIR1230-5/2021(H5N8) | 2020-2022 | West Europe | Wild Anseriformes | H5N8 |
| A/pheasant/Wales/000252/2021(H5N8) | 2020-2022 | West Europe | Other wild species | H5N8 |
| A/seagull/Italy/21VIR2479/2021(H5N8) | 2020-2022 | West Europe | Charadriiformes | H5N8 |
| A/turkey/Germany-MV/AI01015/2021(H5N8) | 2020-2022 | West Europe | Domestic Galliformes | H5N8 |
| A/mute swan/Slovakia/Pah15 21VIR1086-3/2021(H5N5) | 2020-2022 | East Europe | Wild Anseriformes | H5N5 |
| A/turkey/Rostov-on-Don/332-08/2021(H5N8) | 2020-2022 | East Europe | Domestic Galliformes | H5N8 |
| A/turkey/Rostov-on-Don/332-09/2021(H5N8) | 2020-2022 | East Europe | Domestic Galliformes | H5N8 |
| A/turkey/Rostov-on-Don/332-10/2021(H5N8) | 2020-2022 | East Europe | Domestic Galliformes | H5N8 |
| A/turkey/Rostov-on-Don/332-12/2021(H5N8) | 2020-2022 | East Europe | Domestic Galliformes | H5N8 |
| A/chicken/Miyazaki/J12T/2021(H5N8) | 2020-2022 | Japan | Domestic Galliformes | H5N8 |
| A/chicken/Miyazaki/J3T/2021(H5N8) | 2020-2022 | Japan | Domestic Galliformes | H5N8 |
| A/chicken/Miyazaki/J4T/2021(H5N8) | 2020-2022 | Japan | Domestic Galliformes | H5N8 |
| A/chicken/Miyazaki/J6T/2021(H5N8) | 2020-2022 | Japan | Domestic Galliformes | H5N8 |
| A/turkey/Germany-BB/AI01023/2021 (H5N8) | 2020-2022 | West Europe | Domestic Galliformes | H5N8 |
| A/wild goose/Netherlands/21024076-001/2021(H5N8) | 2020-2022 | West Europe | Wild Anseriformes | H5N8 |
| A/barnacle goose/Netherlands/21022611-001/2021(H5N1) | 2020-2022 | West Europe | Wild Anseriformes | H5N1 |
| A/chicken/Bulgaria/39 21VIR1454-3/2021(H5N8) | 2020-2022 | East Europe | Domestic Galliformes | H5N8 |
| A/chicken/Ibaraki/1C/2021(H5N8) | 2020-2022 | Japan | Domestic Galliformes | H5N8 |
| A/chicken/Ibaraki/1T/2021(H5N8) | 2020-2022 | Japan | Domestic Galliformes | H5N8 |
| A/chicken/Ibaraki/2T/2021(H5N8) | 2020-2022 | Japan | Domestic Galliformes | H5N8 |
| A/chicken/Ibaraki/3T/2021(H5N8) | 2020-2022 | Japan | Domestic Galliformes | H5N8 |
| A/chicken/Ibaraki/7C/2021(H5N8) | 2020-2022 | Japan | Domestic Galliformes | H5N8 |
| A/chicken/Ibaraki/8T/2021(H5N8) | 2020-2022 | Japan | Domestic Galliformes | H5N8 |
| A/common buzzard/Netherlands/21022834-002/2021(H5N1) | 2020-2022 | West Europe | Other wild species | H5N8 |
| A/duck/Guangxi/S10099/2021(H5N8) | 2020-2022 | China | Domestic Anseriformes | H5N8 |
| A/duck/Guangxi/S10263/2021(H5N8) | 2020-2022 | China | Domestic Anseriformes | H5N8 |
| A/duck/Guangxi/S11043/2021(H5N8) | 2020-2022 | China | Domestic Anseriformes | H5N8 |
| A/mute swan/Austria/21014124 21VIR1085-8/2021(H5N8) | 2020-2022 | East Europe | Wild Anseriformes | H5N8 |
| A/mute swan/Ibaraki/080203C/2021(H5N8) | 2020-2022 | Japan | Wild Anseriformes | H5N8 |
| A/mute swan/Ibaraki/080203T/2021(H5N8) | 2020-2022 | Japan | Wild Anseriformes | H5N8 |
| A/peacock/Netherlands/21022591-002/2021(H5N8) | 2020-2022 | West Europe | Other wild species | H5N8 |
| A/pheasant/Finland/1589 21VIR7689-3/2021(H5N8) | 2020-2022 | West Europe | Other wild species | H5N8 |
| A/pomeranian goose/Germany-MV/AI01100/2021(H5N8) | 2020-2022 | West Europe | Wild Anseriformes | H5N8 |
| A/tufted duck/Poland/MB061/2021(H5N5) | 2020-2022 | East Europe | Wild Anseriformes | H5N5 |
| A/yellow-legged gull/Switzerland/15-0039 21VIR3035/2021(H5N4) | 2020-2022 | West Europe | Charadriiformes | H5N4 |
| A/chicken/Czech Republic/2395/2021 (H5N8) | 2020-2022 | East Europe | Domestic Galliformes | H5N8 |
| A/chicken/Chiba/E1T/2021(H5N8) | 2020-2022 | Japan | Domestic Galliformes | H5N8 |
| A/chicken/Chiba/E2T/2021(H5N8) | 2020-2022 | Japan | Domestic Galliformes | H5N8 |
| A/chicken/Chiba/E3T/2021(H5N8) | 2020-2022 | Japan | Domestic Galliformes | H5N8 |
| A/chicken/Chiba/E4T/2021(H5N8) | 2020-2022 | Japan | Domestic Galliformes | H5N8 |
| A/chicken/Czech Republic/2502-1/2021(H5N8) | 2020-2022 | East Europe | Domestic Galliformes | H5N8 |
| A/chicken/Czech Republic/2502-2/2021(H5N8) | 2020-2022 | East Europe | Domestic Galliformes | H5N8 |
| A/mute swan/Austria/21011165 21VIR1085-2/2021(H5N8) | 2020-2022 | East Europe | Wild Anseriformes | H5N8 |
| A/mute swan/Czech Republic/2600/2021(H5N8) | 2020-2022 | East Europe | Wild Anseriformes | H5N8 |
| A/mute swan/Czech Republic/2669-1/2021 (H5N8) | 2020-2022 | East Europe | Wild Anseriformes | H5N8 |
| A/mute swan/Czech Republic/2669-2/2021 (H5N8) | 2020-2022 | East Europe | Wild Anseriformes | H5N8 |
| A/mute swan/Netherlands/21022898-002/2021(H5N8) | 2020-2022 | West Europe | Wild Anseriformes | H5N8 |
| A/pekin duck/Bulgaria/48-3 21VIR1454-7/2021(H5N8) | 2020-2022 | East Europe | Wild Anseriformes | H5N8 |
| A/turkey/Germany-BB/AI01120/2021(H5N8) | 2020-2022 | West Europe | Domestic Galliformes | H5N8 |
| A/wigeon/Latvia/23903/2021(H5N8) | 2020-2022 | East Europe | Wild Anseriformes | H5N8 |
| A/barnacle goose/Netherlands/21023501-002/2021(H5N8) | 2020-2022 | West Europe | Wild Anseriformes | H5N8 |
| A/chicken/Chiba/F1T/2021(H5N8) | 2020-2022 | Japan | Domestic Galliformes | H5N8 |
| A/chicken/Chiba/F2T/2021(H5N8) | 2020-2022 | Japan | Domestic Galliformes | H5N8 |
| A/chicken/Chiba/F3C/2021(H5N8) | 2020-2022 | Japan | Domestic Galliformes | H5N8 |
| A/chicken/Chiba/F3T/2021(H5N8) | 2020-2022 | Japan | Domestic Galliformes | H5N8 |
| A/chicken/Chiba/F4T/2021(H5N8) | 2020-2022 | Japan | Domestic Galliformes | H5N8 |
| A/chicken/Nigeria/VRD21-035B 21VIR2288-1/2021(H5N8) | 2020-2022 | Africa | Domestic Galliformes | H5N8 |
| A/chicken/Nigeria/VRD21-37 21VIR2288-2/2021(H5N1) | 2020-2022 | Africa | Domestic Galliformes | H5N1 |
| A/chicken/Chiba/G1C/2021(H5N8) | 2020-2022 | Japan | Domestic Galliformes | H5N8 |
| A/chicken/Chiba/G1T/2021(H5N8) | 2020-2022 | Japan | Domestic Galliformes | H5N8 |
| A/chicken/Chiba/G2C/2021(H5N8) | 2020-2022 | Japan | Domestic Galliformes | H5N8 |
| A/chicken/Chiba/G2T/2021(H5N8) | 2020-2022 | Japan | Domestic Galliformes | H5N8 |
| A/chicken/Miyazaki/K1T/2021(H5N8) | 2020-2022 | Japan | Domestic Galliformes | H5N8 |
| A/chicken/Miyazaki/K3T/2021(H5N8) | 2020-2022 | Japan | Domestic Galliformes | H5N8 |
| A/chicken/Miyazaki/K5T/2021(H5N8) | 2020-2022 | Japan | Domestic Galliformes | H5N8 |
| A/chicken/Miyazaki/K8T/2021(H5N8) | 2020-2022 | Japan | Domestic Galliformes | H5N8 |
| A/turkey/Germany-MV/AI01127/2021(H5N8) | 2020-2022 | West Europe | Domestic Galliformes | H5N8 |
| A/chicken/Chiba/H1C/2021(H5N8) | 2020-2022 | Japan | Domestic Galliformes | H5N8 |
| A/chicken/Chiba/H1T/2021(H5N8) | 2020-2022 | Japan | Domestic Galliformes | H5N8 |
| A/chicken/Chiba/H2C/2021(H5N8) | 2020-2022 | Japan | Domestic Galliformes | H5N8 |
| A/chicken/Chiba/H2T/2021(H5N8) | 2020-2022 | Japan | Domestic Galliformes | H5N8 |
| A/chicken/Germany-BY/AI01355/2021(H5N8) | 2020-2022 | West Europe | Domestic Galliformes | H5N8 |
| A/chicken/Bulgaria/50-1 21VIR1454-9/2021(H5N8) | 2020-2022 | East Europe | Domestic Galliformes | H5N8 |
| A/chicken/Germany-MV/AI01347/2021(H5N8) | 2020-2022 | West Europe | Domestic Galliformes | H5N8 |
| A/chicken/Tokushima/B1T/2020(H5N8) | 2020-2022 | Japan | Domestic Galliformes | H5N8 |
| A/chicken/Tokushima/B2T/2020(H5N8) | 2020-2022 | Japan | Domestic Galliformes | H5N8 |
| A/chicken/Tokushima/B3T/2020(H5N8) | 2020-2022 | Japan | Domestic Galliformes | H5N8 |
| A/chicken/Tokushima/B4T/2020(H5N8) | 2020-2022 | Japan | Domestic Galliformes | H5N8 |
| A/mallard duck/Niigata/150209C/2021(H5N8) | 2020-2022 | Africa | Wild Anseriformes | H5N8 |
| A/mallard duck/Niigata/150209T/2021(H5N8) | 2020-2022 | Africa | Wild Anseriformes | H5N8 |
| A/whooper swan/Fukushima/0701B002/2021(H5N8) | 2020-2022 | Japan | Wild Anseriformes | H5N8 |
| A/chicken/Chiba/I1C/2021(H5N8) | 2020-2022 | Japan | Domestic Galliformes | H5N8 |
| A/chicken/Chiba/I1T/2021(H5N8) | 2020-2022 | Japan | Domestic Galliformes | H5N8 |
| A/chicken/Chiba/I2T/2021(H5N8) | 2020-2022 | Japan | Domestic Galliformes | H5N8 |
| A/chicken/Chiba/I3T/2021(H5N8) | 2020-2022 | Japan | Domestic Galliformes | H5N8 |
| A/chicken/Chiba/J1C/2021(H5N8) | 2020-2022 | Japan | Domestic Galliformes | H5N8 |
| A/chicken/Chiba/J1T/2021(H5N8) | 2020-2022 | Japan | Domestic Galliformes | H5N8 |
| A/chicken/Chiba/J6C/2021(H5N8) | 2020-2022 | Japan | Domestic Galliformes | H5N8 |
| A/chicken/Chiba/J6T/2021(H5N8) | 2020-2022 | Japan | Domestic Galliformes | H5N8 |
| A/chicken/Czech Republic/29392021 (H5N8) | 2020-2022 | East Europe | Domestic Galliformes | H5N8 |
| A/chicken/Nigeria/VRD21-43 21VIR2288-4/2021(H5N8) | 2020-2022 | Africa | Domestic Galliformes | H5N8 |
| A/eastern buzzard/Toyama/160213C/2021(H5N8) | 2020-2022 | Japan | Other wild species | H5N8 |
| A/eastern buzzard/Toyama/160213T/2021(H5N8) | 2020-2022 | Japan | Other wild species | H5N8 |
| A/mute swan/Austria/21013162 21VIR1085-5/2021(H5N5) | 2020-2022 | East Europe | Wild Anseriformes | H5N5 |
| A/pheasant/Scotland/000348/2021(H5N1) | 2020-2022 | West Europe | Other wild species | H5N1 |
| A/chicken/Nigeria/VRD21-53B 21VIR2288-5/2021(H5N8) | 2020-2022 | Africa | Domestic Galliformes | H5N8 |
| A/chicken/Czech Republic/3099-1/2021 (H5N8) | 2020-2022 | East Europe | Domestic Galliformes | H5N8 |
| A/chicken/Czech Republic/3099-2/2021 (H5N8) | 2020-2022 | East Europe | Domestic Galliformes | H5N8 |
| A/chicken/Germany-MV/AI01407/2021(H5N8) | 2020-2022 | West Europe | Domestic Galliformes | H5N8 |
| A/chicken/Nigeria/VRD21-98 21VIR2288-6/2021(H5N1) | 2020-2022 | Africa | Domestic Galliformes | H5N1 |
| A/whooper swan/Niigata/150212T/2021(H5N8) | 2020-2022 | Africa | Wild Anseriformes | H5N8 |
| A/barnacle goose/Netherlands/21023498-002/2021(H5N8) | 2020-2022 | West Europe | Wild Anseriformes | H5N8 |
| A/chicken/Chiba/K11C/2021(H5N8) | 2020-2022 | Japan | Domestic Galliformes | H5N8 |
| A/chicken/Chiba/K11T/2021(H5N8) | 2020-2022 | Japan | Domestic Galliformes | H5N8 |
| A/chicken/Chiba/K5C/2021(H5N8) | 2020-2022 | Japan | Domestic Galliformes | H5N8 |
| A/chicken/Chiba/K5T/2021(H5N8) | 2020-2022 | Japan | Domestic Galliformes | H5N8 |
| A/common coot/Czech Republic/3160-2/2021 (H5N8) | 2020-2022 | East Europe | Other wild species | H5N8 |
| A/mute swan/Czech Republic/3160-1/2021 (H5N8) | 2020-2022 | East Europe | Wild Anseriformes | H5N8 |
| A/whooper swan/Tochigi/090203C/2021(H5N8) | 2020-2022 | Japan | Wild Anseriformes | H5N8 |
| A/eurasian curlew/Netherlands/21024069-002/2021(H5N4) | 2020-2022 | West Europe | Charadriiformes | H5N1 |
| A/mute swan/Poland/MB131/2021(H5N8) | 2020-2022 | East Europe | Wild Anseriformes | H5N8 |
| A/peregrine falcon/Tochigi/090205C/2021(H5N8) | 2020-2022 | Japan | Other wild species | H5N8 |
| A/peregrine falcon/Tochigi/090205T/2021(H5N8) | 2020-2022 | Japan | Other wild species | H5N8 |
| A/turkey/Germany-BB/AI01419/2021(H5N8) | 2020-2022 | West Europe | Domestic Galliformes | H5N8 |
| A/domestic duck/Germany-BB/AI01423/2021(H5N8) | 2020-2022 | West Europe | Domestic Anseriformes | H5N8 |
| A/owl/Tochigi/090204C/2021(H5N8) | 2020-2022 | Japan | Other wild species | H5N8 |
| A/owl/Tochigi/090204T/2021(H5N8) | 2020-2022 | Japan | Other wild species | H5N8 |
| A/barnacle goose/Germany-NI/AI01605/2021(H5N1) | 2020-2022 | West Europe | Wild Anseriformes | H5N1 |
| A/chicken/Italy/21VIR1151-2/2021(H5N8) | 2020-2022 | West Europe | Domestic Galliformes | H5N8 |
| A/domestic duck/Germany-BB/AI01437/2021(H5N8) | 2020-2022 | West Europe | Domestic Anseriformes | H5N8 |
| A/domestic duck/Germany-BB/AI01443/2021(H5N8) | 2020-2022 | West Europe | Domestic Anseriformes | H5N8 |
| A/domestic duck/Germany-BB/AI01444/2021(H5N8) | 2020-2022 | West Europe | Domestic Anseriformes | H5N8 |
| A/Laridae/Germany-SH/AI01498/2021(H5N4) | 2020-2022 | West Europe | Charadriiformes | H5N4 |
| A/whooper swan/Miyagi/0402B001/2021(H5N8) | 2020-2022 | Japan | Wild Anseriformes | H5N8 |
| A/chicken/Czech Republic/3531-1/2021(H5N8) | 2020-2022 | East Europe | Domestic Galliformes | H5N8 |
| A/guinea fowl/Estonia/TA2104719 21VIR7512-9/2021(H5N8) | 2020-2022 | East Europe | Other wild species | H5N8 |
| A/chicken/Netherlands/21023815-001005/2021(H5N8) | 2020-2022 | West Europe | Domestic Galliformes | H5N8 |
| A/mute swan/Czech Republic/3549/2021(H5N8) | 2020-2022 | East Europe | Wild Anseriformes | H5N8 |
| A/turkey/Germany-MV/AI01477/2021(H5N8) | 2020-2022 | West Europe | Domestic Galliformes | H5N8 |
| A/turkey/Germany-NI/AI01483/2021(H5N8) | 2020-2022 | West Europe | Domestic Galliformes | H5N8 |
| A/barnacle goose/Netherlands/21024358-001/2021(H5N8) | 2020-2022 | West Europe | Wild Anseriformes | H5N8 |
| A/common buzzard/Netherlands/21023939-001/2021(H5N8) | 2020-2022 | West Europe | Other wild species | H5N1 |
| A/european herring gull/Netherlands/21023937-002/2021(H5N4) | 2020-2022 | West Europe | Charadriiformes | H5N4 |
| A/turkey/Germany-NI/AI01488/2021(H5N8) | 2020-2022 | West Europe | Domestic Galliformes | H5N8 |
| A/barnacle goose/Netherlands/21024066-001/2021(H5N1) | 2020-2022 | West Europe | Wild Anseriformes | H5N1 |
| A/black-backed gull/Shandong/SC189/2021(H5N8) | 2020-2022 | China | Charadriiformes | H5N8 |
| A/chicken/Germany-MV/AI01587/2021 (H5N8) | 2020-2022 | West Europe | Domestic Galliformes | H5N8 |
| A/common buzzard/Netherlands/21024357-002/2021(H5N3) | 2020-2022 | West Europe | Other wild species | H5N8 |
| A/grebe/Shandong/SC184/2021(H5N8) | 2020-2022 | China | Other wild species | H5N8 |
| A/mute swan/Czech Republic/3777/2021(H5N8) | 2020-2022 | East Europe | Wild Anseriformes | H5N8 |
| A/mute swan/Poland/MB189/2021(H5N8) | 2020-2022 | East Europe | Wild Anseriformes | H5N8 |
| A/whooper swan/Shandong/SC176/2021(H5N8) | 2020-2022 | China | Wild Anseriformes | H5N8 |
| A/whooper swan/Shandong/SC185/2021(H5N8) | 2020-2022 | China | Wild Anseriformes | H5N8 |
| A/whooper swan/Shandong/SC188/2021(H5N8) | 2020-2022 | China | Wild Anseriformes | H5N8 |
| A/wild duck/Shandong/SC177/2021(H5N8) | 2020-2022 | China | Wild Anseriformes | H5N8 |
| A/chicken/Germany-NI/AI01599/2021(H5N1) | 2020-2022 | West Europe | Domestic Galliformes | H5N1 |
| A/chicken/Italy/21VIR1293-9/2021(H5N8) | 2020-2022 | West Europe | Domestic Galliformes | H5N8 |
| A/chicken/Romania/10101 21VIR2044-1/2021(H5N5) | 2020-2022 | East Europe | Domestic Galliformes | H5N5 |
| A/duck/Italy/21VIR1293-15/2021(H5N8) | 2020-2022 | West Europe | Domestic Anseriformes | H5N8 |
| A/guinea fowl/Italy/21VIR1293-20/2021(H5N8) | 2020-2022 | West Europe | Other wild species | H5N8 |
| A/mute swan/Czech Republic/4100/2021(H5N8) | 2020-2022 | East Europe | Wild Anseriformes | H5N8 |
| A/chicken/Germany-BY/AI01617/2021(H5N8) | 2020-2022 | West Europe | Domestic Galliformes | H5N8 |
| A/chicken/Miyazaki/L10T/2021(H5N8) | 2020-2022 | Japan | Domestic Galliformes | H5N8 |
| A/chicken/Miyazaki/L1T/2021(H5N8) | 2020-2022 | Japan | Domestic Galliformes | H5N8 |
| A/chicken/Miyazaki/L4T/2021(H5N8) | 2020-2022 | Japan | Domestic Galliformes | H5N8 |
| A/chicken/Miyazaki/L9T/2021(H5N8) | 2020-2022 | Japan | Domestic Galliformes | H5N8 |
| A/eastern buzzard/Toyama/160208C/2021(H5N8) | 2020-2022 | Japan | Other wild species | H5N8 |
| A/eastern buzzard/Toyama/160208T/2021(H5N8) | 2020-2022 | Japan | Other wild species | H5N8 |
| A/mute swan/Czech Republic/4099/2021(H5N5) | 2020-2022 | East Europe | Wild Anseriformes | H5N5 |
| A/chicken/Czech Republic/3893/2021(H5N8) | 2020-2022 | East Europe | Domestic Galliformes | H5N8 |
| A/chicken/Nigeria/VRD21-88 21VIR2288-8/2021(H5N8) | 2020-2022 | Africa | Domestic Galliformes | H5N8 |
| A/chicken/Germany-BE/AI01974/2021(H5N8) | 2020-2022 | West Europe | Domestic Galliformes | H5N8 |
| A/chicken/Germany-MV/AI01794/2021(H5N8) | 2020-2022 | West Europe | Domestic Galliformes | H5N8 |
| A/swan/Germany-HE/AI02335/2021(H5N8) | 2020-2022 | West Europe | Wild Anseriformes | H5N8 |
| A/swan/Lithuania/1258PG1 21VIR2606-2/2021(H5N8) | 2020-2022 | East Europe | Wild Anseriformes | H5N8 |
| A/swan/Lithuania/1298PG1 21VIR2606-3/2021(H5N8) | 2020-2022 | East Europe | Wild Anseriformes | H5N8 |
| A/chicken/Czech Republic/4092-1/2021(H5N8) | 2020-2022 | East Europe | Domestic Galliformes | H5N8 |
| A/chicken/Czech Republic/4092-2/2021(H5N8) | 2020-2022 | East Europe | Domestic Galliformes | H5N8 |
| A/turkey/Germany-NI/AI01799/2021(H5N8) | 2020-2022 | West Europe | Domestic Galliformes | H5N8 |
| A/turkey/Germany-NI/AI01805/2021(H5N8) | 2020-2022 | West Europe | Domestic Galliformes | H5N8 |
| A/Aalopochen Aegyptiaca/Belgium/2928 002/2021(H5N8) | 2020-2022 | West Europe | Wild Anseriformes | H5N8 |
| A/chicken/Germany-NI/AI01815/2021(H5N8) | 2020-2022 | West Europe | Domestic Galliformes | H5N8 |
| A/turkey/Germany-BB/AI01980/2021(H5N8) | 2020-2022 | West Europe | Domestic Galliformes | H5N8 |
| A/turkey/Germany-NI/AI02013/2021(H5N8) | 2020-2022 | West Europe | Domestic Galliformes | H5N8 |
| A/barnacle goose/Denmark/17572-1.01/2021-03-01(H5N1) | 2020-2022 | West Europe | Wild Anseriformes | H5N1 |
| A/chicken/Germany-BY/AI02060/2021 (H5N8) | 2020-2022 | West Europe | Domestic Galliformes | H5N8 |
| A/chicken/Germany-BY/AI02064/2021(H5N8) | 2020-2022 | West Europe | Domestic Galliformes | H5N8 |
| A/chicken/Germany-MV/AI01965/2021(H5N8) | 2020-2022 | West Europe | Domestic Galliformes | H5N8 |
| A/chicken/Germany-NW/AI02054/2021 (H5N8) | 2020-2022 | West Europe | Domestic Galliformes | H5N8 |
| A/chicken/Nigeria/VRD21-100 21VIR2370-423/2021(H5N8) | 2020-2022 | Africa | Domestic Galliformes | H5N8 |
| A/chicken/Nigeria/VRD21-102 21VIR2370-424/2021(H5N1) | 2020-2022 | Africa | Domestic Galliformes | H5N1 |
| A/chicken/Nigeria/VRD21-109 21VIR2370-425/2021(H5N1) | 2020-2022 | Africa | Domestic Galliformes | H5N1 |
| A/Columba palumbus/Belgium/2928 004/2021(H5N8) | 2020-2022 | West Europe | Other wild species | H5N8 |
| A/domestic duck/Germany-NW/AI02049/2021(H5N8) | 2020-2022 | West Europe | Domestic Anseriformes | H5N8 |
| A/Garrulus glandarius/Belgium/2928 003/2021(H5N8) | 2020-2022 | West Europe | Other wild species | H5N8 |
| A/mute swan/Croatia/14/2021 (H5N8) | 2020-2022 | East Europe | Wild Anseriformes | H5N8 |
| A/chicken/Germany-BY/AI02066/2021 (H5N8) | 2020-2022 | West Europe | Domestic Galliformes | H5N8 |
| A/duck/Guangdong/S1269/2021(H5N8) | 2020-2022 | China | Domestic Anseriformes | H5N8 |
| A/greater rhea/Germany-SN/AI02351/2021(H5N8) | 2020-2022 | West Europe | Other wild species | H5N8 |
| A/mute swan/Czech Republic/4270/2021(H5N8) | 2020-2022 | East Europe | Wild Anseriformes | H5N8 |
| A/mute swan/Estonia/TA2106111-2 21VIR7512-1/2021(H5N8) | 2020-2022 | East Europe | Wild Anseriformes | H5N8 |
| A/mute swan/Estonia/TA2106241 21VIR7512-2/2021(H5N8) | 2020-2022 | East Europe | Wild Anseriformes | H5N8 |
| A/mute swan/Germany-HE/AI02373/2021(H5N8) | 2020-2022 | West Europe | Wild Anseriformes | H5N8 |
| A/peregrine falcon/Sweden/SVA210309SZ0403/FB000851/K-2021(H5N4) | 2020-2022 | West Europe | Other wild species | H5N4 |
| A/turkey/Germany-NW/AI02290/2021(H5N8) | 2020-2022 | West Europe | Domestic Galliformes | H5N8 |
| A/eastern buzzard/Tochigi/090311C/2021(H5N8) | 2020-2022 | Japan | Other wild species | H5N8 |
| A/eastern buzzard/Tochigi/090311T/2021(H5N8) | 2020-2022 | Japan | Other wild species | H5N8 |
| A/mute swan/Poland/MB268/2021(H5N8) | 2020-2022 | East Europe | Wild Anseriformes | H5N8 |
| A/turkey/Germany-NI/AI02025/2021(H5N8) | 2020-2022 | West Europe | Domestic Galliformes | H5N8 |
| A/chicken/Germany-MV/AI02141/2021(H5N8) | 2020-2022 | West Europe | Domestic Galliformes | H5N8 |
| A/common buzzard/Netherlands/21024712-002/2021(H5N8) | 2020-2022 | West Europe | Other wild species | H5N3 |
| A/domestic goose/Germany-SH/AI02100/2021(H5N8) | 2020-2022 | West Europe | Domestic Anseriformes | H5N8 |
| A/domestic goose/Germany-SH/AI02102/2021(H5N8) | 2020-2022 | West Europe | Domestic Anseriformes | H5N8 |
| A/mute swan/Czech Republic/4606/2021(H5N8) | 2020-2022 | East Europe | Wild Anseriformes | H5N8 |
| A/mute swan/Czech Republic/4607-1/2021(H5N8) | 2020-2022 | East Europe | Wild Anseriformes | H5N8 |
| A/mute swan/Czech Republic/4607-2/2021(H5N8) | 2020-2022 | East Europe | Wild Anseriformes | H5N8 |
| A/mute swan/Estonia/TA2106419-1 21VIR7512-3/2021(H5N8) | 2020-2022 | East Europe | Wild Anseriformes | H5N8 |
| A/mute swan/Estonia/TA2106615 21VIR7512-4/2021(H5N8) | 2020-2022 | East Europe | Wild Anseriformes | H5N8 |
| A/turkey/Germany-BB/AI02117/2021(H5N8) | 2020-2022 | West Europe | Domestic Galliformes | H5N8 |
| A/turkey/Germany-BB/AI02318/2021(H5N8) | 2020-2022 | West Europe | Domestic Galliformes | H5N8 |
| A/turkey/Germany-NI/AI02122/2021(H5N8) | 2020-2022 | West Europe | Domestic Galliformes | H5N8 |
| A/turkey/Germany-NI/AI02128/2021(H5N8) | 2020-2022 | West Europe | Domestic Galliformes | H5N8 |
| A/chicken/Germany-SH/AI02312/2021 (H5N8) | 2020-2022 | West Europe | Domestic Galliformes | H5N8 |
| A/chicken/Germany-SN/AI02353/2021(H5N8) | 2020-2022 | West Europe | Domestic Galliformes | H5N8 |
| A/domestic goose/Germany-MV/AI02319/2021 (H5N8) | 2020-2022 | West Europe | Domestic Anseriformes | H5N8 |
| A/duck/Poland/H160 21RS1385-1/2021(H5N8) | 2020-2022 | East Europe | Domestic Anseriformes | H5N8 |
| A/peregrine falcon/Sweden/SVA210325SZ0348/FP001210/H-2021(H5N4) | 2020-2022 | West Europe | Other wild species | H5N4 |
| A/turkey/Germany-NI/AI02303/2021(H5N8) | 2020-2022 | West Europe | Domestic Galliformes | H5N8 |
| A/turkey/Germany-NI/AI02306/2021(H5N8) | 2020-2022 | West Europe | Domestic Galliformes | H5N8 |
| A/chicken/Czech Republic/4526/2021(H5N8) | 2020-2022 | East Europe | Domestic Galliformes | H5N8 |
| A/chicken/Czech Republic/4527-1/2021(H5N8) | 2020-2022 | East Europe | Domestic Galliformes | H5N8 |
| A/chicken/Germany-NI/AI02328/2021(H5N8) | 2020-2022 | West Europe | Domestic Galliformes | H5N8 |
| A/mute swan/Czech Republic/4799/2021(H5N8) | 2020-2022 | East Europe | Wild Anseriformes | H5N8 |
| A/mute swan/Poland/MB272/2021(H5N8) | 2020-2022 | East Europe | Wild Anseriformes | H5N8 |
| A/turkey/Germany-NI/AI02349/2021(H5N8) | 2020-2022 | West Europe | Domestic Galliformes | H5N8 |
| A/chicken/Germany-MV/AI02376/2021 (H5N8) | 2020-2022 | West Europe | Domestic Galliformes | H5N8 |
| A/eagle/Hungary/8569/2021 (H5N5) | 2020-2022 | East Europe | Other wild species | H5N8 |
| A/mute swan/Croatia/19/2021(H5N8) | 2020-2022 | East Europe | Wild Anseriformes | H5N8 |
| A/swan/Hungary/9638/2021 (H5N8) | 2020-2022 | East Europe | Wild Anseriformes | H5N8 |
| A/turkey/Germany-NI/AI02393/2021(H5N8) | 2020-2022 | West Europe | Domestic Galliformes | H5N8 |
| A/barnacle goose/Netherlands/21024897-001/2021(H5N8) | 2020-2022 | West Europe | Wild Anseriformes | H5N8 |
| A/chicken/Germany-NI/AI02412/2021 (H5N8) | 2020-2022 | West Europe | Domestic Galliformes | H5N8 |
| A/duck/Jiangxi/S10252/2021(H5N8) | 2020-2022 | China | Domestic Anseriformes | H5N8 |
| A/goose/Zhejiang/S1266/2021(H5N8) | 2020-2022 | China | Domestic Anseriformes | H5N8 |
| A/chicken/Czech Republic/4756/2021(H5N8) | 2020-2022 | East Europe | Domestic Galliformes | H5N8 |
| A/chicken/Germany-NI/AI02543/2021(H5N8) | 2020-2022 | West Europe | Domestic Galliformes | H5N8 |
| A/domestic goose/Germany-MV/AI02558/2021(H5N8) | 2020-2022 | West Europe | Domestic Anseriformes | H5N8 |
| A/eagle owl/Germany-ST/AI02542/2021(H5N8) | 2020-2022 | West Europe | Other wild species | H5N5 |
| A/peregrine falcon/Netherlands/21025108-001/2021(H5N4) | 2020-2022 | West Europe | Other wild species | H5N4 |
| A/turkey/Germany-BB/AI02434/2021 (H5N8) | 2020-2022 | West Europe | Domestic Galliformes | H5N8 |
| A/turkey/Germany-MV/AI02458/2021(H5N8) | 2020-2022 | West Europe | Domestic Galliformes | H5N8 |
| A/turkey/Germany-NI/AI02424/2021 (H5N8) | 2020-2022 | West Europe | Domestic Galliformes | H5N8 |
| A/turkey/Germany-NI/AI02553/2021(H5N8) | 2020-2022 | West Europe | Domestic Galliformes | H5N8 |
| A/chicken/Germany-SH/AI02562/2021 (H5N8) | 2020-2022 | West Europe | Domestic Galliformes | H5N8 |
| A/domestic duck/Germany-BB/AI02599/2021(H5N8) | 2020-2022 | West Europe | Domestic Anseriformes | H5N8 |
| A/chicken/Czech Republic/4980/2021(H5N8) | 2020-2022 | East Europe | Domestic Galliformes | H5N8 |
| A/turkey/Germany-NI/AI02615/2021(H5N8) | 2020-2022 | West Europe | Domestic Galliformes | H5N8 |
| A/chicken/Germany-NI/AI02954/2021(H5N8) | 2020-2022 | West Europe | Domestic Galliformes | H5N8 |
| A/chicken/Tochigi/2T/2021(H5N8) | 2020-2022 | Japan | Domestic Galliformes | H5N8 |
| A/chicken/Tochigi/3T/2021(H5N8) | 2020-2022 | Japan | Domestic Galliformes | H5N8 |
| A/chicken/Tochigi/4T/2021(H5N8) | 2020-2022 | Japan | Domestic Galliformes | H5N8 |
| A/chicken/Tochigi/5T/2021(H5N8) | 2020-2022 | Japan | Domestic Galliformes | H5N8 |
| A/goose/Czech Republic/5363-10/2021(H5N8) | 2020-2022 | East Europe | Domestic Anseriformes | H5N8 |
| A/turkey/Germany-NI/AI02678/2021 (H5N8) | 2020-2022 | West Europe | Domestic Galliformes | H5N8 |
| A/domestic duck/Germany-NI/AI02660/2021(H5N8) | 2020-2022 | West Europe | Domestic Anseriformes | H5N8 |
| A/turkey/Germany-NI/AI02663/2021 (H5N8) | 2020-2022 | West Europe | Domestic Galliformes | H5N8 |
| A/turkey/Germany-NI/AI02671/2021 (H5N8) | 2020-2022 | West Europe | Domestic Galliformes | H5N8 |
| A/turkey/Germany-NI/AI02674/2021 (H5N8) | 2020-2022 | West Europe | Domestic Galliformes | H5N8 |
| A/turkey/Germany-NI/AI02950/2021(H5N8) | 2020-2022 | West Europe | Domestic Galliformes | H5N8 |
| A/chicken/Germany-NI/AI02942/2021 (H5N8) | 2020-2022 | West Europe | Domestic Galliformes | H5N8 |
| A/turkey/Germany-NI/AI02933/2021 (H5N8) | 2020-2022 | West Europe | Domestic Galliformes | H5N8 |
| A/turkey/Germany-NI/AI02943/2021(H5N8) | 2020-2022 | West Europe | Domestic Galliformes | H5N8 |
| A/chicken/Germany-ST/AI02967/2021(H5N8) | 2020-2022 | West Europe | Domestic Galliformes | H5N8 |
| A/domestic goose/Germany-NI/AI02980/2021(H5N8) | 2020-2022 | West Europe | Domestic Anseriformes | H5N8 |
| A/common murre/Netherlands/21025491-002/2021(H5N1) | 2020-2022 | West Europe | Other wild species | H5N1 |
| A/domestic duck/Germany-NI/AI03088/2021 (H5N8) | 2020-2022 | West Europe | Domestic Anseriformes | H5N8 |
| A/domestic goose/Germany-NI/AI03093/2021(H5N8) | 2020-2022 | West Europe | Domestic Anseriformes | H5N8 |
| A/duck/Czech Republic/5360-1/2021(H5N8) | 2020-2022 | East Europe | Domestic Anseriformes | H5N8 |
| A/duck/Czech Republic/5360-2/2021(H5N8) | 2020-2022 | East Europe | Domestic Anseriformes | H5N8 |
| A/duck/Czech Republic/5361/2021(H5N8) | 2020-2022 | East Europe | Domestic Anseriformes | H5N8 |
| A/turkey/Germany-NI/AI02991/2021(H5N8) | 2020-2022 | West Europe | Domestic Galliformes | H5N8 |
| A/turkey/Germany-NW/AI03104/2021(H5N8) | 2020-2022 | West Europe | Domestic Galliformes | H5N8 |
| A/domestic duck/Germany-NI/AI03099/2021(H5N8) | 2020-2022 | West Europe | Domestic Anseriformes | H5N8 |
| A/duck/Czech Republic/5448/2021(H5N8) | 2020-2022 | East Europe | Domestic Anseriformes | H5N8 |
| A/duck/Czech Republic/5466/2021(H5N8) | 2020-2022 | East Europe | Domestic Anseriformes | H5N8 |
| A/duck/Czech Republic/5467/2021 (H5N8) | 2020-2022 | East Europe | Domestic Anseriformes | H5N8 |
| A/goose/Hunan/S11288/2021(H5N8) | 2020-2022 | China | Domestic Anseriformes | H5N8 |
| A/swan/Lithuania/1842PG1 21VIR2606-5/2021(H5N8) | 2020-2022 | East Europe | Wild Anseriformes | H5N8 |
| A/swan/Lithuania/2306PG1 21VIR2606-7/2021(H5N8) | 2020-2022 | East Europe | Wild Anseriformes | H5N8 |
| A/turkey/Germany-NI/AI03126/2021(H5N8) | 2020-2022 | West Europe | Domestic Galliformes | H5N8 |
| A/chicken/Germany-NW/AI03148/2021(H5N8) | 2020-2022 | West Europe | Domestic Galliformes | H5N8 |
| A/chicken/Germany-NW/AI03154/2021(H5N8) | 2020-2022 | West Europe | Domestic Galliformes | H5N8 |
| A/domestic goose/Germany-NI/AI03142/2021(H5N8) | 2020-2022 | West Europe | Domestic Anseriformes | H5N8 |
| A/domestic duck/Germany-ST/AI03183/2021(H5N8) | 2020-2022 | West Europe | Domestic Anseriformes | H5N8 |
| A/turkey/Germany-NI/AI03160/2021 (H5N8) | 2020-2022 | West Europe | Domestic Galliformes | H5N8 |
| A/barnacle goose/Netherlands/21025769-002/2021(H5N1) | 2020-2022 | West Europe | Wild Anseriformes | H5N1 |
| A/chicken/Germany-BW/AI03205/2021(H5N8) | 2020-2022 | West Europe | Domestic Galliformes | H5N8 |
| A/chicken/Germany-TH/AI03193/2021(H5N8) | 2020-2022 | West Europe | Domestic Galliformes | H5N8 |
| A/dalmatian pelican/Greece/47t 21VIR3735-3/2021(H5N8) | 2020-2022 | East Europe | Other wild species | H5N8 |
| A/chicken/Germany-TH/AI03512/2021(H5N8) | 2020-2022 | West Europe | Domestic Galliformes | H5N8 |
| A/chicken/Poland/H293 21RS1385-8/2021(H5N8) | 2020-2022 | East Europe | Domestic Galliformes | H5N8 |
| A/turkey/Germany-NI/AI03209/2021(H5N8) | 2020-2022 | West Europe | Domestic Galliformes | H5N8 |
| A/chicken/Germany-BW/AI03554/2021(H5N8) | 2020-2022 | West Europe | Domestic Galliformes | H5N8 |
| A/chicken/Germany-BY/AI03544/2021 (H5N8) | 2020-2022 | West Europe | Domestic Galliformes | H5N8 |
| A/chicken/Germany-SN/AI03496/2021(H5N8) | 2020-2022 | West Europe | Domestic Galliformes | H5N8 |
| A/chicken/Germany-SN/AI03502/2021(H5N8) | 2020-2022 | West Europe | Domestic Galliformes | H5N8 |
| A/chicken/Germany-TH/AI03513/2021(H5N8) | 2020-2022 | West Europe | Domestic Galliformes | H5N8 |
| A/duck/Czech Republic/5792-13/2021(H5N8) | 2020-2022 | East Europe | Domestic Anseriformes | H5N8 |
| A/duck/Czech Republic/5792-19/2021(H5N8) | 2020-2022 | East Europe | Domestic Anseriformes | H5N8 |
| A/goose/Jiangsu/S1385/2021(H5N8) | 2020-2022 | China | Domestic Anseriformes | H5N8 |
| A/australian brushturkey/Czech Republic/5904/2021(H5N8) | 2020-2022 | East Europe | Other wild species | H5N8 |
| A/chicken/Czech Republic/5903/2021(H5N8) | 2020-2022 | East Europe | Domestic Galliformes | H5N8 |
| A/duck/Czech Republic/6017/2021 (H5N8) | 2020-2022 | East Europe | Domestic Anseriformes | H5N8 |
| A/mute swan/Estonia/TA2108545-1 21VIR7512-5/2021(H5N8) | 2020-2022 | East Europe | Wild Anseriformes | H5N8 |
| A/turkey/Germany-MV/AI03535/2021(H5N8) | 2020-2022 | West Europe | Domestic Galliformes | H5N8 |
| A/chicken/Czech Republic/6151-1/2021(H5N8) | 2020-2022 | East Europe | Domestic Galliformes | H5N8 |
| A/chicken/Czech Republic/6151-2/2021(H5N8) | 2020-2022 | East Europe | Domestic Galliformes | H5N8 |
| A/chicken/Germany-BW/AI03634/2021(H5N8) | 2020-2022 | West Europe | Domestic Galliformes | H5N8 |
| A/European herring gull/Bulgaria/222 21VIR4270-1/2021(H5N5) | 2020-2022 | East Europe | Charadriiformes | H5N5 |
| A/grey heron/Bulgaria/223 21VIR4270-2/2021(H5N5) | 2020-2022 | East Europe | Other wild species | H5N5 |
| A/chicken/Germany-NW/AI03705/2021 (H5N8) | 2020-2022 | West Europe | Domestic Galliformes | H5N8 |
| A/chicken/Czech Republic/6527/2021(H5N8) | 2020-2022 | East Europe | Domestic Galliformes | H5N8 |
| A/chicken/Czech Republic/6532-1/2021(H5N8) | 2020-2022 | East Europe | Domestic Galliformes | H5N8 |
| A/chicken/Czech Republic/6532-2/2021(H5N8) | 2020-2022 | East Europe | Domestic Galliformes | H5N8 |
| A/peacock/Czech Republic/6529-2/2021(H5N8) | 2020-2022 | East Europe | Other wild species | H5N8 |
| A/chicken/Czech Republic/6542-1/2021(H5N8) | 2020-2022 | East Europe | Domestic Galliformes | H5N8 |
| A/chicken/Czech Republic/6542-2/2021(H5N8) | 2020-2022 | East Europe | Domestic Galliformes | H5N8 |
| A/chicken/Czech Republic/6654/2021(H5N8) | 2020-2022 | East Europe | Domestic Galliformes | H5N8 |
| A/chicken/Czech Republic/6684/2021(H5N8) | 2020-2022 | East Europe | Domestic Galliformes | H5N8 |
| A/duck/Czech Republic/6653-15/2021(H5N8) | 2020-2022 | East Europe | Domestic Anseriformes | H5N8 |
| A/duck/Czech Republic/6653-4/2021(H5N8) | 2020-2022 | East Europe | Domestic Anseriformes | H5N8 |
| A/duck/Czech Republic/6653-5/2021(H5N8) | 2020-2022 | East Europe | Domestic Anseriformes | H5N8 |
| A/duck/Czech Republic/6653-6/2021(H5N8) | 2020-2022 | East Europe | Domestic Anseriformes | H5N8 |
| A/duck/Poland/H514 21RS1385-18/2021(H5N8) | 2020-2022 | East Europe | Domestic Anseriformes | H5N8 |
| A/duck/Poland/H515 21RS1385-17/2021(H5N8) | 2020-2022 | East Europe | Domestic Anseriformes | H5N8 |
| A/peacock/Netherlands/21026542-001/2021(H5N8) | 2020-2022 | West Europe | Other wild species | H5N8 |
| A/barnacle goose/Germany-NI/AI03914/2021(H5N1) | 2020-2022 | West Europe | Wild Anseriformes | H5N1 |
| A/chicken/Czech Republic/7100/2021(H5N8) | 2020-2022 | East Europe | Domestic Galliformes | H5N8 |
| A/mute swan/Romania/11981-1 21VIR3163-5/2021(H5N5) | 2020-2022 | East Europe | Wild Anseriformes | H5N5 |
| A/mute swan/Romania/11981-2 21VIR3163-6/2021(H5N8) | 2020-2022 | East Europe | Wild Anseriformes | H5N8 |
| A/duck/Poland/H542 21RS1385-2/2021(H5N8) | 2020-2022 | East Europe | Domestic Anseriformes | H5N8 |
| A/Gallus gallus/Belgium/5107 002/2021(H5N8) | 2020-2022 | West Europe | Domestic Galliformes | H5N8 |
| A/goose/Liaoning/S1266/2021(H5N8) | 2020-2022 | China | Domestic Anseriformes | H5N8 |
| A/Turkey/Hungary/16603/2021 (H5N1) | 2020-2022 | East Europe | Domestic Galliformes | H5N1 |
| A/barnacle goose/Germany-NI/AI03980/2021(H5N1) | 2020-2022 | West Europe | Wild Anseriformes | H5N1 |
| A/barnacle goose/Netherlands/21027016-002/2021(H5N1) | 2020-2022 | West Europe | Wild Anseriformes | H5N1 |
| A/chicken/Poland/H712 21RS1385-12/2021(H5N8) | 2020-2022 | East Europe | Domestic Galliformes | H5N8 |
| A/barnacle/goose Denmark/19027-1.02/2021-04-18(H5N1) | 2020-2022 | West Europe | Wild Anseriformes | H5N1 |
| A/duck/Czech Republic/7681-10/2021(H5N8) | 2020-2022 | East Europe | Domestic Anseriformes | H5N8 |
| A/duck/Czech Republic/7681-3/2021(H5N8) | 2020-2022 | East Europe | Domestic Anseriformes | H5N8 |
| A/duck/Czech Republic/7681-5/2021(H5N8) | 2020-2022 | East Europe | Domestic Anseriformes | H5N8 |
| A/duck/Czech Republic/7681-7/2021(H5N8) | 2020-2022 | East Europe | Domestic Anseriformes | H5N8 |
| A/duck/Czech Republic/7681-8/2021(H5N8) | 2020-2022 | East Europe | Domestic Anseriformes | H5N8 |
| A/duck/Czech Republic/7682-2/2021(H5N8) | 2020-2022 | East Europe | Domestic Anseriformes | H5N8 |
| A/duck/Czech Republic/7682-5/2021(H5N8) | 2020-2022 | East Europe | Domestic Anseriformes | H5N8 |
| A/duck/Czech Republic/7682-9/2021(H5N8) | 2020-2022 | East Europe | Domestic Anseriformes | H5N8 |
| A/Anas platyrhynchos domestica/Belgium/5517/2021(H5N8) | 2020-2022 | West Europe | Wild Anseriformes | H5N8 |
| A/barnacle goose/Netherlands/21027357-002/2021(H5N1) | 2020-2022 | West Europe | Wild Anseriformes | H5N1 |
| A/chicken/Poland/H812 21RS1385-7/2021(H5N8) | 2020-2022 | East Europe | Domestic Galliformes | H5N8 |
| A/white stork/Poland/MB391/2021(H5N1) | 2020-2022 | East Europe | Other wild species | H5N1 |
| A/barnacle goose/Netherlands/21028196-002/2021(H5N1) | 2020-2022 | West Europe | Wild Anseriformes | H5N1 |
| A/duck/Henan/S1381/2021(H5N8) | 2020-2022 | China | Domestic Anseriformes | H5N8 |
| A/goose/Henan/S1315/2021(H5N8) | 2020-2022 | China | Domestic Anseriformes | H5N8 |
| A/mute swan/Poland/MB396 21RS1385-19/2021(H5N8) | 2020-2022 | East Europe | Wild Anseriformes | H5N8 |
| A/chicken/Bulgaria/274-5 21VIR4270-4/2021(H5N8) | 2020-2022 | East Europe | Domestic Galliformes | H5N8 |
| A/white-tailed eagle/Netherlands/21027616-001/2021(H5N1) | 2020-2022 | West Europe | Other wild species | H5N1 |
| A/chicken/Poland/H928 21RS1385-16/2021(H5N8) | 2020-2022 | East Europe | Domestic Galliformes | H5N8 |
| A/chicken/Bulgaria/275-4 21VIR4270-6/2021(H5N8) | 2020-2022 | East Europe | Domestic Galliformes | H5N8 |
| A/chicken/Austria/21052483 21VIR3291-1/2021(H5N8) | 2020-2022 | East Europe | Domestic Galliformes | H5N8 |
| A/duck/Hebei/S1070/2021(H5N8) | 2020-2022 | China | Domestic Anseriformes | H5N8 |
| A/mute swan/Austria/21051907 21VIR3291-6/2021(H5N8) | 2020-2022 | East Europe | Wild Anseriformes | H5N8 |
| A/mute swan/Austria/21051907 21VIR3291-7/2021(H5N8) | 2020-2022 | East Europe | Wild Anseriformes | H5N8 |
| A/turkey/Germany-NI/AI04373/2021(H5N1) | 2020-2022 | West Europe | Domestic Galliformes | H5N1 |
| A/chicken/Poland/H984 21RS1385-9/2021(H5N8) | 2020-2022 | East Europe | Domestic Galliformes | H5N8 |
| A/barnacle goose/Finland//6378 21VIR7689-7/2021(H5N1) | 2020-2022 | West Europe | Wild Anseriformes | H5N1 |
| A/barnacle goose/Finland/6247 21VIR7689-6/2021(H5N1) | 2020-2022 | West Europe | Wild Anseriformes | H5N1 |
| A/duck/Poland/H1029 21RS1385-5/2021(H5N8) | 2020-2022 | East Europe | Domestic Anseriformes | H5N8 |
| A/white stork/Poland/MB412 21RS1385-11/2021(H5N8) | 2020-2022 | East Europe | Other wild species | H5N8 |
| A/goose/Poland/H1036 21RS1385-6/2021(H5N8) | 2020-2022 | East Europe | Domestic Anseriformes | H5N8 |
| A/turkey/Germany-NI/AI04425/2021(H5N1) | 2020-2022 | West Europe | Domestic Galliformes | H5N1 |
| A/chicken/Bulgaria/297-2 21VIR4270-8/2021(H5N8) | 2020-2022 | East Europe | Domestic Galliformes | H5N8 |
| A/chicken/Bulgaria/298-1 21VIR4270-9/2021(H5N8) | 2020-2022 | East Europe | Domestic Galliformes | H5N8 |
| A/chicken/Romania/12448 21VIR3734-3/2021(H5N8) | 2020-2022 | East Europe | Domestic Galliformes | H5N8 |
| A/goose/Poland/H1044 21RS1385-4/2021(H5N8) | 2020-2022 | East Europe | Domestic Anseriformes | H5N8 |
| A/turkey/Germany-NI/AI04455/2021(H5N1) | 2020-2022 | West Europe | Domestic Galliformes | H5N1 |
| A/chicken/Poland/H1084 21RS1385-13/2021(H5N8) | 2020-2022 | East Europe | Domestic Galliformes | H5N8 |
| A/western marsh harrier/Netherlands/21028606-002/2021(H5N1) | 2020-2022 | West Europe | Other wild species | H5N1 |
| A/goose/Netherlands/21028502-002/21028502/2021(H5N1) | 2020-2022 | West Europe | Domestic Anseriformes | H5N1 |
| A/duck/Guangxi/S21194/2021(H5N8) | 2020-2022 | China | Domestic Anseriformes | H5N8 |
| A/barnacle goose/Sweden/SVA210511SZ0567/FB001840/M-2021(H5N1) | 2020-2022 | West Europe | Wild Anseriformes | H5N1 |
| A/chicken/Poland/H1124 21RS1385-3/2021(H5N8) | 2020-2022 | East Europe | Domestic Galliformes | H5N8 |
| A/goose/Guangxi/S20601/2021(H5N8) | 2020-2022 | China | Domestic Anseriformes | H5N8 |
| A/barnacle goose/Netherlands/21028534-002/2021(H5N1) | 2020-2022 | West Europe | Wild Anseriformes | H5N1 |
| A/chicken/Poland/H1161 21RS1385-10/2021(H5N8) | 2020-2022 | East Europe | Domestic Galliformes | H5N8 |
| A/turkey/Poland/H1168 21RS1385-15/2021(H5N8) | 2020-2022 | East Europe | Domestic Galliformes | H5N8 |
| A/European herring gull/Denmark/19968-1.02/2021-05-14(H5N1) | 2020-2022 | West Europe | Charadriiformes | H5N1 |
| A/turkey/Poland/H1184 21RS1385-14/2021(H5N8) | 2020-2022 | East Europe | Domestic Galliformes | H5N8 |
| A/Bar-headed Goose/Tibet/XZ181/2021(H5N8) | 2020-2022 | China | Wild Anseriformes | H5N8 |
| A/Bar-headed Goose/Tibet/XZ6/2021(H5N8) | 2020-2022 | China | Wild Anseriformes | H5N8 |
| A/Bar-headed Goose/Tibet/XZ71/2021(H5N8) | 2020-2022 | China | Wild Anseriformes | H5N8 |
| A/Bar-headed Goose/Tibet/XZ81/2021(H5N8) | 2020-2022 | China | Wild Anseriformes | H5N8 |
| A/Bar-headed Goose/Tibet/XZQ10-1/2021(H5N8) | 2020-2022 | China | Wild Anseriformes | H5N8 |
| A/Bar-headed Goose/Tibet/XZQ5-1/2021(H5N8) | 2020-2022 | China | Wild Anseriformes | H5N8 |
| A/Bar-headed Goose/Tibet/XZQ7/2021(H5N8) | 2020-2022 | China | Wild Anseriformes | H5N8 |
| A/Bar-headed Goose/Tibet/XZQ8-1/2021(H5N8) | 2020-2022 | China | Wild Anseriformes | H5N8 |
| A/Bar-headed Goose/Tibet/XZQ9-1/2021(H5N8) | 2020-2022 | China | Wild Anseriformes | H5N8 |
| A/brown-headed gull/Tibet/1-1/2021(H5N8) | 2020-2022 | China | Charadriiformes | H5N8 |
| A/chicken/Czech Republic/10251-1/2021(H5N8) | 2020-2022 | East Europe | Domestic Galliformes | H5N8 |
| A/white-tailed eagle/Estonia/TA2111864-2 21VIR7512-6/2021(H5N1) | 2020-2022 | East Europe | Other wild species | H5N1 |
| A/Bar-headed Goose/Tibet/XZQ13-1/2021(H5N8) | 2020-2022 | China | Wild Anseriformes | H5N8 |
| A/Bar-headed Goose/Tibet/XZQ17-1/2021(H5N8) | 2020-2022 | China | Wild Anseriformes | H5N8 |
| A/Bar-headed Goose/Tibet/XZQ18-1/2021(H5N8) | 2020-2022 | China | Wild Anseriformes | H5N8 |
| A/Brown-headed Gull/Tibet/XZ19/2021(H5N8) | 2020-2022 | China | Charadriiformes | H5N8 |
| A/Brown-headed Gull/Tibet/XZQ15-2/2021(H5N8) | 2020-2022 | China | Charadriiformes | H5N8 |
| A/Brown-headed Gull/Tibet/XZQ16-2/2021(H5N8) | 2020-2022 | China | Charadriiformes | H5N8 |
| A/chicken/Czech Republic/10405/2021(H5N8) | 2020-2022 | East Europe | Domestic Galliformes | H5N8 |
| A/red fox/Netherlands/21028774-002/2021(H5N1) | 2020-2022 | West Europe | Mammal | H5N1 |
| A/red fox/Netherlands/21028774-004/2021(H5N1) | 2020-2022 | West Europe | Mammal | H5N1 |
| A/chicken/Kosovo/82 21VIR5162-1/2021(H5N8) | 2020-2022 | East Europe | Domestic Galliformes | H5N8 |
| A/Bar-headed Goose/Tibet/XZ1131/2021(H5N1) | 2020-2022 | China | Wild Anseriformes | H5N1 |
| A/Bar-headed Goose/Tibet/XZ901/2021(H5N1) | 2020-2022 | China | Wild Anseriformes | H5N1 |
| A/turkey/Netherlands/21028936-001005/2021(H5N8) | 2020-2022 | West Europe | Domestic Galliformes | H5N8 |
| A/turkey/Poland/H1289 21RS1385-20/2021(H5N8) | 2020-2022 | East Europe | Domestic Galliformes | H5N8 |
| A/chicken/Kosovo/84 21VIR5162-2/2021(H5N8) | 2020-2022 | East Europe | Domestic Galliformes | H5N8 |
| A/chicken/Kosovo/90 21VIR5162-4/2021(H5N8) | 2020-2022 | East Europe | Domestic Galliformes | H5N8 |
| A/turkey/Kosovo/90 21VIR5162-5/2021(H5N8) | 2020-2022 | East Europe | Domestic Galliformes | H5N8 |
| A/White-Tailed Eagle/Sweden/SVA210528SZ0223/KN002027/AB-2021(H5N1) | 2020-2022 | West Europe | Other wild species | H5N1 |
| A/chicken/Albania/2574 21VIR5387-1/2021(H5N8) | 2020-2022 | East Europe | Domestic Galliformes | H5N8 |
| A/chicken/Czech Republic/10251-2/2021(H5N8) | 2020-2022 | East Europe | Domestic Galliformes | H5N8 |
| A/chicken/Kosovo/97 21VIR5162-15/2021(H5N8) | 2020-2022 | East Europe | Domestic Galliformes | H5N8 |
| A/chicken/Kosovo/98 21VIR5162-16/2021(H5N8) | 2020-2022 | East Europe | Domestic Galliformes | H5N8 |
| A/partridge/Kosovo/96 21VIR5162-14/2021(H5N8) | 2020-2022 | East Europe | Other wild species | H5N8 |
| A/barnacle goose/Finland/6955 21VIR7689-9/2021(H5N1) | 2020-2022 | West Europe | Wild Anseriformes | H5N1 |
| A/chicken/Kosovo/104 21VIR5162-7/2021(H5N8) | 2020-2022 | East Europe | Domestic Galliformes | H5N8 |
| A/white-tailed eagle/Finland/6984 21VIR7689-10/2021(H5N1) | 2020-2022 | West Europe | Other wild species | H5N1 |
| A/duck/Kosovo/107 21VIR5162-17/2021(H5N8) | 2020-2022 | East Europe | Domestic Anseriformes | H5N8 |
| A/gull/Estonia/TA2113284-4 21VIR7512-8/2021(H5N1) | 2020-2022 | East Europe | Charadriiformes | H5N1 |
| A/chicken/Kosovo/113 21VIR5162-19/2021(H5N8) | 2020-2022 | East Europe | Domestic Galliformes | H5N8 |
| A/chicken/Albania/3816 21VIR5387-2/2021(H5N8) | 2020-2022 | East Europe | Domestic Galliformes | H5N8 |
| A/Gallus gallus/Belgium/7578 001/2021(H5N8) | 2020-2022 | West Europe | Domestic Galliformes | H5N8 |
| A/grebe/Shaanxi/SD001/2021(H5N8) | 2020-2022 | China | Other wild species | H5N8 |
| A/chicken/chongqing/H1/2021(H5N6) | 2020-2022 | China | Domestic Galliformes | H5N6 |
| A/chicken/Kosovo/126 22VIR3124-18/2021(H5N8) | 2020-2022 | East Europe | Domestic Galliformes | H5N8 |
| A/greylag goose/Norway/V294 21VIR7634-1/2021(H5N8) | 2020-2022 | West Europe | Wild Anseriformes | H5N8 |
| A/grebe/Ningxia/SD001/2021(H5N8) | 2020-2022 | China | Other wild species | H5N8 |
| A/mute swan/Norway/FU452 21VIR7634-2/2021(H5N8) | 2020-2022 | West Europe | Wild Anseriformes | H5N8 |
| A/common eider/Norway/FU453 21VIR7634-3/2021(H5N8) | 2020-2022 | West Europe | Wild Anseriformes | H5N8 |
| A/golden eagle/Finland/9378 21VIR7689-12/2021(H5N1) | 2016-2017 | West Europe | Other wild species | H5N5 |
| A/common eider/Norway/FU458 21VIR7634-4/2021(H5N8) | 2020-2022 | West Europe | Wild Anseriformes | H5N8 |
| A/white-tailed eagle/Finland/9257 21VIR7689-11/2021(H5N8) | 2020-2022 | West Europe | Other wild species | H5N8 |
| A/Sichuan/06689/2021(H5N6) | 2020-2022 | China | Human | H5N6 |
| A/Chongqing/02/2021(H5N6) | 2020-2022 | China | Human | H5N6 |
| A/Gallus gallus/Belgium/9247/2021(H5N8) | 2020-2022 | West Europe | Domestic Galliformes | H5N8 |
| A/GX-guilin/11151/2021(H5N6) | 2020-2022 | China | Human | H5N6 |
| A/oystercatcher/Germany-NI/AI05047/2021(H5N1) | 2020-2022 | West Europe | Charadriiformes | H5N1 |
| A/great skua/Scotland/041672/2021(H5N1) | 2020-2022 | West Europe | Charadriiformes | H5N1 |
| A/swan/Germany-NI/AI05045/2021(H5N8) | 2020-2022 | West Europe | Wild Anseriformes | H5N8 |
| A/great skua/Scotland/042505/2021(H5N1) | 2020-2022 | West Europe | Charadriiformes | H5N1 |
| A/great skua/Scotland/B07779/2021(H5N1) | 2020-2022 | West Europe | Charadriiformes | H5N1 |
| A/Eurasian eagle-owl/Finland/10617 21VIR7689-15/2021(H5N1) | 2020-2022 | West Europe | Other wild species | H5N1 |
| A/European herring gull/Finland/9722 21VIR7689-13/2021(H5N1) | 2020-2022 | West Europe | Charadriiformes | H5N1 |
| A/whooper swan/Finland/9906 21VIR7689-14/2021(H5N8) | 2020-2022 | West Europe | Wild Anseriformes | H5N8 |
| A/Hunan/09285/2021(H5N6) | 2020-2022 | China | Human | H5N6 |
| A/seal/Germany-SH/AI05373/2021(H5N8) | 2020-2022 | West Europe | Mammal | H5N8 |
| A/seal/Germany-SH/AI05377/2021(H5N8) | 2020-2022 | West Europe | Mammal | H5N8 |
| A/seal/Germany-SH/AI05379/2021(H5N8) | 2020-2022 | West Europe | Mammal | H5N8 |
| A/Meleagris gallopavo/Belgium/11293 001/2021(H5N8) | 2020-2022 | West Europe | Domestic Galliformes | H5N8 |
| A/Gallus gallus/Belgium/11372 0001/2021(H5N8) | 2020-2022 | West Europe | Domestic Galliformes | H5N8 |
| A/chicken/Luxembourg/21168413/2021 (H5N8) | 2020-2022 | West Europe | Domestic Galliformes | H5N8 |
| A/Hunan/09911/2021(H5N6) | 2020-2022 | China | Human | H5N6 |
| A/Gallus gallus/Belgium/11644/2021(H5N8) | 2020-2022 | West Europe | Domestic Galliformes | H5N8 |
| A/Hunan/10117/2021(H5N6) | 2020-2022 | China | Human | H5N6 |
| A/Eagle owl/Estonia/TA2122239 21VIR10433-10/2021(H5N8) | 2020-2022 | East Europe | Other wild species | H5N8 |
| A/turkey/Tyumen/15-14V/2021(H5N1) | 2020-2022 | Russian Federation Siberia | Domestic Galliformes | H5N1 |
| A/turkey/Tyumen/15-1V/2021(H5N1) | 2020-2022 | Russian Federation Siberia | Domestic Galliformes | H5N1 |
| A/turkey/Tyumen/15-2V/2021(H5N1) | 2020-2022 | Russian Federation Siberia | Domestic Galliformes | H5N1 |
| A/turkey/Tyumen/15-3V/2021(H5N1) | 2020-2022 | Russian Federation Siberia | Domestic Galliformes | H5N1 |
| A/turkey/Tyumen/15-4V/2021(H5N1) | 2020-2022 | Russian Federation Siberia | Domestic Galliformes | H5N1 |
| A/turkey/Tyumen/15-6V/2021(H5N1) | 2020-2022 | Russian Federation Siberia | Domestic Galliformes | H5N1 |
| A/turkey/Tyumen/15-9V/2021(H5N1) | 2020-2022 | Russian Federation Siberia | Domestic Galliformes | H5N1 |
| A/common pheasant /Sweden/SVA210923SZ0341/KN000365/M-2021-H5N1(H5N1) | 2020-2022 | West Europe | Other wild species | H5N1 |
| A/common pheasant /Sweden/SVA210923SZ0341/KN000366/M-2021(H5N1) | 2020-2022 | West Europe | Other wild species | H5N1 |
| A/duck/Omsk/19-1V/2021(H5N1) | 2020-2022 | Russian Federation Siberia | Domestic Anseriformes | H5N1 |
| A/duck/Omsk/19-2V/2021(H5N1) | 2020-2022 | Russian Federation Siberia | Domestic Anseriformes | H5N1 |
| A/duck/Omsk/19-3V/2021(H5N1) | 2020-2022 | Russian Federation Siberia | Domestic Anseriformes | H5N1 |
| A/duck/Omsk/19-5V/2021(H5N1) | 2020-2022 | Russian Federation Siberia | Domestic Anseriformes | H5N1 |
| A/duck/Czech Republic/18520-2/2021(H5N1) | 2020-2022 | East Europe | Domestic Anseriformes | H5N1 |
| A/goose/Czech Republic/18520-1/2021(H5N1) | 2020-2022 | East Europe | Domestic Anseriformes | H5N1 |
| A/chicken/Kosovo/278 22VIR3124-1/2021(H5N8) | 2020-2022 | East Europe | Domestic Galliformes | H5N8 |
| A/chicken/Saratov/29-06V/2021(H5N1) | 2020-2022 | East Europe | Domestic Galliformes | H5N1 |
| A/chicken/Saratov/29-07V/2021(H5N1) | 2020-2022 | East Europe | Domestic Galliformes | H5N1 |
| A/chicken/Saratov/29-6V/2021(H5N1) | 2020-2022 | East Europe | Domestic Galliformes | H5N1 |
| A/duck/Saratov/29-02V/2021(H5N1) | 2020-2022 | East Europe | Domestic Anseriformes | H5N1 |
| A/duck/Saratov/29-03V/2021(H5N1) | 2020-2022 | East Europe | Domestic Anseriformes | H5N1 |
| A/duck/Saratov/29-04V/2021(H5N1) | 2020-2022 | East Europe | Domestic Anseriformes | H5N1 |
| A/duck/Saratov/29-08V/2021(H5N1) | 2020-2022 | East Europe | Domestic Anseriformes | H5N1 |
| A/duck/Saratov/29-11V/2021(H5N1) | 2020-2022 | East Europe | Domestic Anseriformes | H5N1 |
| A/duck/Saratov/29-2V/2021(H5N1) | 2020-2022 | East Europe | Domestic Anseriformes | H5N1 |
| A/chicken/Tyumen/27-31V/2021(H5N1) | 2020-2022 | Russian Federation Siberia | Domestic Galliformes | H5N1 |
| A/chicken/Tyumen/27-39V/2021(H5N1) | 2020-2022 | Russian Federation Siberia | Domestic Galliformes | H5N1 |
| A/chicken/Tyumen/27-42V/2021(H5N1) | 2020-2022 | Russian Federation Siberia | Domestic Galliformes | H5N1 |
| A/chicken/Kosovo/279 22VIR3124-2/2021(H5N8) | 2020-2022 | East Europe | Domestic Galliformes | H5N8 |
| A/chicken/Kosovo/280 22VIR3124-3/2021(H5N8) | 2020-2022 | East Europe | Domestic Galliformes | H5N8 |
| A/chicken/Tyumen/27-31V/2021(H5N1) | 2020-2022 | Russian Federation Siberia | Domestic Galliformes | H5N1 |
| A/chicken/Tyumen/27-39V/2021(H5N1) | 2020-2022 | Russian Federation Siberia | Domestic Galliformes | H5N1 |
| A/chicken/Tyumen/27-40V/2021(H5N1) | 2020-2022 | Russian Federation Siberia | Domestic Galliformes | H5N1 |
| A/chicken/Tyumen/27-42V/2021(H5N1) | 2020-2022 | Russian Federation Siberia | Domestic Galliformes | H5N1 |
| A/chicken/Kosovo/283 22VIR3124-4/2021(H5N8) | 2020-2022 | East Europe | Domestic Galliformes | H5N8 |
| A/chicken/Tyumen/33-45V/2021(H5N1) | 2020-2022 | Russian Federation Siberia | Domestic Galliformes | H5N1 |
| A/goose/Tyumen/33-52V/2021(H5N1) | 2020-2022 | Russian Federation Siberia | Domestic Anseriformes | H5N1 |
| A/goose/Tyumen/33-53V/2021(H5N1) | 2020-2022 | Russian Federation Siberia | Domestic Anseriformes | H5N1 |
| A/goose/Rostov-on-Don/28-1V/2021(H5N1) | 2020-2022 | East Europe | Domestic Anseriformes | H5N1 |
| A/chicken/Kosovo/284 22VIR3124-5/2021(H5N8) | 2020-2022 | East Europe | Domestic Galliformes | H5N8 |
| A/chicken/Tyumen/47-66V/2021(H5N1) | 2020-2022 | Russian Federation Siberia | Domestic Galliformes | H5N1 |
| A/goose/Chelyabinsk/34-1V/2021(H5N1) | 2020-2022 | Russian Federation Siberia | Domestic Anseriformes | H5N1 |
| A/chicken/Tyumen/47-79V/2021(H5N1) | 2020-2022 | Russian Federation Siberia | Domestic Galliformes | H5N1 |
| A/chicken/Tyumen/47-85V/2021(H5N1) | 2020-2022 | Russian Federation Siberia | Domestic Galliformes | H5N1 |
| A/chicken/Tyumen/47-88V/2021(H5N1) | 2020-2022 | Russian Federation Siberia | Domestic Galliformes | H5N1 |
| A/chicken/Tyumen/47-95V/2021(H5N1) | 2020-2022 | Russian Federation Siberia | Domestic Galliformes | H5N1 |
| A/turkey/Israel/537/2021(H5N1) | 2020-2022 | West Central Asia | Domestic Galliformes | H5N1 |
| A/Withe-tiled eagle/Estonia/TA2124126-1 21VIR10433-11/2021(H5N1) | 2020-2022 | East Europe | Other wild species | H5N1 |
| A/chicken/Saratov/102-12V/2021(H5N1) | 2020-2022 | East Europe | Domestic Galliformes | H5N1 |
| A/Eurasian wigeon/Denmark/24066-9/2021-10-13(H5N1) | 2020-2022 | West Europe | Wild Anseriformes | H5N1 |
| A/Eurasian wigeon/Germany-SH/AI05948/2021(H5N1) | 2020-2022 | West Europe | Wild Anseriformes | H5N1 |
| A/chicken/Orenburg/46-1V/2021(H5N1) | 2020-2022 | Russian Federation Siberia | Domestic Galliformes | H5N1 |
| A/chicken/Orenburg/46-2V/2021(H5N1) | 2020-2022 | Russian Federation Siberia | Domestic Galliformes | H5N1 |
| A/goose/Orenburg/46-3V/2021(H5N1) | 2020-2022 | Russian Federation Siberia | Domestic Anseriformes | H5N1 |
| A/Eurasian teal/Denmark/24115-2/2021-10-16(H5N1) | 2020-2022 | West Europe | Wild Anseriformes | H5N1 |
| A/common quail/Kosovo/288 22VIR3124-6/2021(H5N8) | 2020-2022 | East Europe | Domestic Galliformes | H5N8 |
| A/duck/Guangxi/S31116/2021(H5N6) | 2020-2022 | China | Domestic Anseriformes | H5N6 |
| A/turkey/Italy/21VIR8585-1/2021(H5N1) | 2020-2022 | West Europe | Domestic Galliformes | H5N1 |
| A/duck/Guangxi/S30428/2021(H5N6) | 2020-2022 | China | Domestic Anseriformes | H5N6 |
| A/greylag goose/Netherlands/21037497-001/2021(H5N1) | 2020-2022 | West Europe | Wild Anseriformes | H5N1 |
| A/chicken/Kosovo/290 22VIR3124-7/2021(H5N8) | 2020-2022 | East Europe | Domestic Galliformes | H5N8 |
| A/chicken/Kurgan/72-1V/2021(H5N1) | 2020-2022 | Russian Federation Siberia | Domestic Galliformes | H5N1 |
| A/chicken/Netherlands/21037233-001/2021(H5N1) | 2020-2022 | West Europe | Domestic Galliformes | H5N1 |
| A/chicken/England/053052/2021(H5N1) | 2020-2022 | West Europe | Domestic Galliformes | H5N1 |
| A/Eurasian wigeon/Denmark/24279-1/2021-10-24(H5N1) | 2020-2022 | West Europe | Wild Anseriformes | H5N1 |
| A/mute swan/England/053054/2021(H5N1) | 2020-2022 | West Europe | Wild Anseriformes | H5N1 |
| A/Mute swan/Netherlands/21037283-002/2021(H5N1) | 2020-2022 | West Europe | Wild Anseriformes | H5N1 |
| A/chicken/Netherlands/21037287-006010/2021(H5N1) | 2020-2022 | West Europe | Domestic Galliformes | H5N1 |
| A/common buzzard/Denmark/24271-1.02/2021-10-25(H5N1) | 2020-2022 | West Europe | Other wild species | H5N8 |
| A/turkey/Italy/21VIR8817-1/2021(H5N1) | 2020-2022 | West Europe | Domestic Galliformes | H5N1 |
| A/barnacle goose/Denmark/24273-1.02/2021-10-26(H5N1) | 2020-2022 | West Europe | Wild Anseriformes | H5N1 |
| A/mandarin duck/Korea/WA585/2021(H5N1) | 2020-2022 | Korea | Wild Anseriformes | H5N1 |
| A/chicken/Kirov/63-1V/2021(H5N1) | 2020-2022 | East Europe | Domestic Galliformes | H5N1 |
| A/chicken/Kirov/63-2V/2021(H5N1) | 2020-2022 | East Europe | Domestic Galliformes | H5N1 |
| A/chicken/Kirov/63-5V/2021(H5N1) | 2020-2022 | East Europe | Domestic Galliformes | H5N1 |
| A/chicken/Orenburg/73-5V/2021(H5N1) | 2020-2022 | Russian Federation Siberia | Domestic Galliformes | H5N1 |
| A/chicken/Orenburg/73-6V/2021(H5N1) | 2020-2022 | Russian Federation Siberia | Domestic Galliformes | H5N1 |
| A/chicken/Orenburg/73-7V/2021(H5N1) | 2020-2022 | Russian Federation Siberia | Domestic Galliformes | H5N1 |
| A/greylag goose/Denmark/24309-1.01/2021-10-27(H5N1) | 2020-2022 | West Europe | Wild Anseriformes | H5N1 |
| A/mallard/Italy/21VIR8919-2/2021(H5N1) | 2020-2022 | West Europe | Wild Anseriformes | H5N1 |
| A/pheasant/Wales/385129/2021(H5N1) | 2020-2022 | West Europe | Other wild species | H5N1 |
| A/chicken/Italy/IZSLT-122448 21VIR9218-1/2021(H5N1) | 2020-2022 | West Europe | Domestic Galliformes | H5N1 |
| A/Eurasian wigeon/Italy/21VIR8919-3/2021(H5N1) | 2020-2022 | West Europe | Wild Anseriformes | H5N1 |
| A/gray heron/Denmark/24326-1.02/2021-10-28(H5N1) | 2020-2022 | West Europe | Other wild species | H5N1 |
| A/common buzzard /Sweden/SVA211104SZ0320/FB004419/M-2021(H5N1) | 2020-2022 | West Europe | Other wild species | H5N1 |
| A/common pheasant /Sweden/SVA211104SZ0320/FB004417/M-2021(H5N1) | 2020-2022 | West Europe | Other wild species | H5N1 |
| A/common pheasant /Sweden/SVA211104SZ0320/FB004418/M-2021(H5N1) | 2020-2022 | West Europe | Other wild species | H5N1 |
| A/barnacle goose/Denmark/24342-1.02/2021-10-30(H5N1) | 2020-2022 | West Europe | Wild Anseriformes | H5N1 |
| A/chicken/Tyumen/81-97V/2021(H5N1) | 2020-2022 | Russian Federation Siberia | Domestic Galliformes | H5N1 |
| A/chicken/Wales/053969/2021(H5N1) | 2020-2022 | West Europe | Domestic Galliformes | H5N1 |
| A/Greylag goose/England/054503/2021(H5N1) | 2020-2022 | West Europe | Wild Anseriformes | H5N1 |
| A/turkey/Denmark/24325-25/2021-10-30(H5N1) | 2020-2022 | West Europe | Domestic Galliformes | H5N1 |
| A/turkey/Tyumen/81-96V/2021(H5N1) | 2020-2022 | Russian Federation Siberia | Domestic Galliformes | H5N1 |
| A/greylag goose/Netherlands/21037809-001/2021(H5N1) | 2020-2022 | West Europe | Wild Anseriformes | H5N1 |
| A/barnacle goose/Sweden/SVA211111SZ0376/FB004496/2021(H5N1) | 2020-2022 | West Europe | Wild Anseriformes | H5N1 |
| A/Canada goose/England/385250/2021(H5N1) | 2020-2022 | West Europe | Wild Anseriformes | H5N1 |
| A/chicken/Scotland/054477/2021(H5N1) | 2020-2022 | West Europe | Domestic Galliformes | H5N1 |
| A/domestic duck/Scotland/054469/2021(H5N1) | 2020-2022 | West Europe | Domestic Anseriformes | H5N1 |
| A/greylag goose/Denmark/24343-1.02/2021-11-01(H5N1) | 2020-2022 | West Europe | Wild Anseriformes | H5N1 |
| A/greylag goose /Sweden/SVA211103SZ0398/FB004410/M-2021(H5N1) | 2020-2022 | West Europe | Wild Anseriformes | H5N1 |
| A/guineafowl/Scotland/054471/2021(H5N1) | 2020-2022 | West Europe | Other wild species | H5N1 |
| A/mute swan/Czech Republic/21312/2021(H5N1) | 2020-2022 | East Europe | Wild Anseriformes | H5N1 |
| A/Turkey/Egypt/A2/2021(H5N8) | 2020-2022 | Africa | Domestic Galliformes | H5N8 |
| A/turkey/Poland/H1910-T3/2021(H5N1) | 2020-2022 | East Europe | Domestic Galliformes | H5N1 |
| A/turkey/Poland/H1911-N/2021(H5N1) | 2020-2022 | East Europe | Domestic Galliformes | H5N1 |
| A/barnacle goose/Sweden/SVA211102SZ0402/FB004395/M-2021(H5N1) | 2020-2022 | West Europe | Wild Anseriformes | H5N1 |
| A/chicken/Denmark/24357-11/2021-11-02(H5N1) | 2020-2022 | West Europe | Domestic Galliformes | H5N1 |
| A/duck/Yunnan/S4318/2021(H5N6) | 2020-2022 | China | Domestic Anseriformes | H5N6 |
| A/turkey/Poland/H1913-T1/2021(H5N1) | 2020-2022 | East Europe | Domestic Galliformes | H5N1 |
| A/black-necked grebe/Kalmykia/78-1V/2021(H5N5) | 2020-2022 | East Europe | Other wild species | H5N5 |
| A/chicken/Kosovo/299 22VIR3124-8/2021(H5N8) | 2020-2022 | East Europe | Domestic Galliformes | H5N8 |
| A/domestic goose/Poland/H1931-T1/2021(H5N1) | 2020-2022 | East Europe | Domestic Anseriformes | H5N1 |
| A/turkey/Italy/21VIR9143-2/2021(H5N1) | 2020-2022 | West Europe | Domestic Galliformes | H5N1 |
| A/turkey/Poland/H1924-T1/2021(H5N1) | 2020-2022 | East Europe | Domestic Galliformes | H5N1 |
| A/white-tailed eagle/Ireland/032034 22VIR1325-23/2021(H5N1) | 2020-2022 | West Europe | Other wild species | H5N1 |
| A/Branta leucopsis/Belgium/14735 0001/2021(H5N1) | 2020-2022 | West Europe | Wild Anseriformes | H5N1 |
| A/chicken/Italy/21VIR9133-20/2021(H5N1) | 2020-2022 | West Europe | Domestic Galliformes | H5N1 |
| A/chicken/Saratov/102-12V/2021(H5N1) | 2020-2022 | East Europe | Domestic Galliformes | H5N1 |
| A/chicken/Saratov/102-15V/2021(H5N1) | 2020-2022 | East Europe | Domestic Galliformes | H5N1 |
| A/bean goose/Sweden/SVA211111SZ0372/FB004482/2021(H5N1) | 2020-2022 | West Europe | Wild Anseriformes | H5N1 |
| A/chicken/Kosovo/302 22VIR3124-9/2021(H5N8) | 2020-2022 | East Europe | Domestic Galliformes | H5N8 |
| A/chicken/Kosovo/303 22VIR3124-10/2021(H5N8) | 2020-2022 | East Europe | Domestic Galliformes | H5N8 |
| A/chicken/Poland/H1940-N/2021(H5N1) | 2020-2022 | East Europe | Domestic Galliformes | H5N1 |
| A/magpie/Italy/21VIR9487-2/2021(H5N1) | 2020-2022 | West Europe | Other wild species | H5N1 |
| A/turkey/Italy/21VIR9210-1/2021(H5N1) | 2020-2022 | West Europe | Domestic Galliformes | H5N1 |
| A/turkey/England/055251/2021(H5N1) | 2020-2022 | West Europe | Domestic Galliformes | H5N1 |
| A/chicken/Netherlands/21038165-006010/2021(H5N1) | 2020-2022 | West Europe | Domestic Galliformes | H5N1 |
| A/domestic duck/Poland/H1942-N/2021(H5N1) | 2020-2022 | East Europe | Domestic Anseriformes | H5N1 |
| A/European herring gull/Sweden/SVA211116SZ0432/FB004518/M-2021(H5N1) | 2020-2022 | West Europe | Charadriiformes | H5N1 |
| A/goose/Netherlands/21038413-002/2021(H5N1) | 2020-2022 | West Europe | Domestic Anseriformes | H5N1 |
| A/greylag goose /Sweden/SVA211111SZ0376/FB004497/M-2021(H5N1) | 2020-2022 | West Europe | Wild Anseriformes | H5N1 |
| A/mute swan/Ireland/032363 22VIR1325-1/2021(H5N1) | 2020-2022 | West Europe | Wild Anseriformes | H5N1 |
| A/mute swan/Poland/MB490-L1/2021(H5N1) | 2020-2022 | East Europe | Wild Anseriformes | H5N1 |
| A/quail/Korea/H526/2021(H5N1) | 2020-2022 | Korea | Domestic Galliformes | H5N1 |
| A/Red fox/Estonia/TA2126820 21VIR10433-13/2021(H5N1) | 2020-2022 | East Europe | Mammal | H5N1 |
| A/swan/France/21P012384/2021(H5N1) | 2020-2022 | West Europe | Wild Anseriformes | H5N1 |
| A/turkey/Poland/H1944-N/2021(H5N1) | 2020-2022 | East Europe | Domestic Galliformes | H5N1 |
| A/western jackdaw/Sweden/SVA211111SZ0376/FB004483/2021(H5N1) | 2020-2022 | West Europe | Other wild species | H5N1 |
| A/chicken/Akita/7C/2021(H5N8) | 2020-2022 | Japan | Domestic Galliformes | H5N8 |
| A/common buzzard/Sweden/SVA211111SZ0376/FB004484/2021(H5N1) | 2020-2022 | West Europe | Other wild species | H5N1 |
| A/great black-backed gull/Sweden/SVA211109SZ0434/FB004445/M-2021(H5N1) | 2020-2022 | West Europe | Charadriiformes | H5N1 |
| A/mute swan/Netherlands/21038479-002/2021(H5N1) | 2020-2022 | West Europe | Wild Anseriformes | H5N1 |
| A/whooper swan/Ireland/032444 22VIR1325-2/2021(H5N1) | 2020-2022 | West Europe | Wild Anseriformes | H5N1 |
| A/Whooper swan/Scotland/056219/2021(H5N1) | 2020-2022 | West Europe | Wild Anseriformes | H5N1 |
| A/peregrine falcon/Ireland/032476 22VIR1325-3/2021(H5N1) | 2020-2022 | West Europe | Other wild species | H5N1 |
| A/turkey/England/056764/2021(H5N1) | 2020-2022 | West Europe | Domestic Galliformes | H5N1 |
| A/Cygnus olor/Romania/16381 21VIR10306/2021(H5N1) | 2020-2022 | East Europe | Wild Anseriformes | H5N1 |
| A/mute swan/England/385466/2021(H5N1) | 2020-2022 | West Europe | Wild Anseriformes | H5N1 |
| A/seagull/Italy/21VIR9432-2/2021(H5N1) | 2020-2022 | West Europe | Charadriiformes | H5N1 |
| A/turkey/Italy/21VIR9510-1/2021(H5N1) | 2020-2022 | West Europe | Domestic Galliformes | H5N1 |
| A/turkey/Italy/21VIR9512-1/2021(H5N1) | 2020-2022 | West Europe | Domestic Galliformes | H5N1 |
| A/chicken/Czech Republic/22224-2T/2021(H5N1) | 2020-2022 | East Europe | Domestic Galliformes | H5N1 |
| A/chicken/Czech Republic/22224-3K/2021(H5N1) | 2020-2022 | East Europe | Domestic Galliformes | H5N1 |
| A/chicken/Czech Republic/22224-3T/2021(H5N1) | 2020-2022 | East Europe | Domestic Galliformes | H5N1 |
| A/chicken/Czech Republic/22224-4T/2021(H5N1) | 2020-2022 | East Europe | Domestic Galliformes | H5N1 |
| A/chicken/Kagoshima/21A6T/2021(H5N1) | 2020-2022 | Japan | Domestic Galliformes | H5N1 |
| A/common buzzard/Netherlands/21038793-001/2021(H5N1) | 2020-2022 | West Europe | Other wild species | H5N8 |
| A/magpie/Ireland/032958 22VIR1325-5/2021(H5N1) | 2020-2022 | West Europe | Other wild species | H5N1 |
| A/mute swan/Croatia/100/2021(H5N1) | 2020-2022 | East Europe | Wild Anseriformes | H5N1 |
| A/mute swan/Croatia/101/2021(H5N1) | 2020-2022 | East Europe | Wild Anseriformes | H5N1 |
| A/mute swan/Czech Republic/22477-1/2021(H5N1) | 2020-2022 | East Europe | Wild Anseriformes | H5N1 |
| A/mute swan/Czech Republic/22477-2/2021(H5N1) | 2020-2022 | East Europe | Wild Anseriformes | H5N1 |
| A/whooper swan/Ireland/032960 22VIR1325-4/2021(H5N1) | 2020-2022 | West Europe | Wild Anseriformes | H5N1 |
| A/chicken/England/057314/2021(H5N1) | 2020-2022 | West Europe | Domestic Galliformes | H5N1 |
| A/chicken/Kagoshima/B3T/2021(H5N8) | 2020-2022 | Japan | Domestic Galliformes | H5N8 |
| A/chicken/Netherlands/21038675-001005/2021(H5N1) | 2020-2022 | West Europe | Domestic Galliformes | H5N1 |
| A/turkey/Italy/21VIR9520/2021(H5N1) | 2020-2022 | West Europe | Domestic Galliformes | H5N1 |
| A/chicken/Italy/21VIR9580-22/2021(H5N1) | 2020-2022 | West Europe | Domestic Galliformes | H5N1 |
| A/chicken/Italy/21VIR9691-2/2021(H5N1) | 2020-2022 | West Europe | Domestic Galliformes | H5N1 |
| A/greylag goose/Ireland/032969 22VIR1325-6/2021(H5N1) | 2020-2022 | West Europe | Wild Anseriformes | H5N1 |
| A/greylag goose/Ireland/033062 22VIR1325-7/2021(H5N1) | 2020-2022 | West Europe | Wild Anseriformes | H5N1 |
| A/hen/Bulgaria/722-1 22VIR778-1/2021(H5N1) | 2020-2022 | East Europe | Domestic Galliformes | H5N1 |
| A/mute swan/Czech Republic/22380/2021(H5N1) | 2020-2022 | East Europe | Wild Anseriformes | H5N1 |
| A/mute swan/Czech Republic/22684/2021(H5N1) | 2020-2022 | East Europe | Wild Anseriformes | H5N1 |
| A/mute swan/Ireland/033169 22VIR1325-14/2021(H5N1) | 2020-2022 | West Europe | Wild Anseriformes | H5N1 |
| A/turkey/Italy/21VIR9649-2/2021(H5N1) | 2020-2022 | West Europe | Domestic Galliformes | H5N1 |
| A/turkey/Italy/21VIR9652-2/2021(H5N1) | 2020-2022 | West Europe | Domestic Galliformes | H5N1 |
| A/white-fronted goose/Ireland/033181 22VIR1325-8/2021(H5N1) | 2020-2022 | West Europe | Wild Anseriformes | H5N1 |
| A/brent goose/Ireland/033257 22VIR1325-9/2021(H5N1) | 2020-2022 | West Europe | Wild Anseriformes | H5N1 |
| A/turkey/England/057679/2021(H5N1) | 2020-2022 | West Europe | Domestic Galliformes | H5N1 |
| A/chicken/Italy/21VIR9765-12/2021(H5N1) | 2020-2022 | West Europe | Domestic Galliformes | H5N1 |
| A/greylag goose /Sweden/SVA211118SZ0354/FB004497/I-2021(H5N1) | 2020-2022 | West Europe | Wild Anseriformes | H5N1 |
| A/herring gull/Ireland/033533 22VIR1325-10/2021(H5N1) | 2020-2022 | West Europe | Charadriiformes | H5N1 |
| A/turkey/Italy/21VIR9767-3/2021(H5N1) | 2020-2022 | West Europe | Domestic Galliformes | H5N1 |
| A/turkey/Italy/21VIR9768-8/2021(H5N1) | 2020-2022 | West Europe | Domestic Galliformes | H5N1 |
| A/domestic duck/England/058612/2021(H5N1) | 2020-2022 | West Europe | Domestic Anseriformes | H5N1 |
| A/goose/Croatia/107/2021(H5N1) | 2020-2022 | East Europe | Domestic Anseriformes | H5N1 |
| A/goose/Czech Republic/22608-1/2021(H5N1) | 2020-2022 | East Europe | Domestic Anseriformes | H5N1 |
| A/goose/Czech Republic/22608-1T/2021(H5N1) | 2020-2022 | East Europe | Domestic Anseriformes | H5N1 |
| A/goose/Czech Republic/22608-2/2021(H5N1) | 2020-2022 | East Europe | Domestic Anseriformes | H5N1 |
| A/goose/Czech Republic/22608-2T/2021(H5N1) | 2020-2022 | East Europe | Domestic Anseriformes | H5N1 |
| A/goose/Czech Republic/22608-3T/2021(H5N1) | 2020-2022 | East Europe | Domestic Anseriformes | H5N1 |
| A/turkey/Italy/21VIR9618-7/2021(H5N1) | 2020-2022 | West Europe | Domestic Galliformes | H5N1 |
| A/goose/Czech Republic/22750/2021(H5N1) | 2020-2022 | East Europe | Domestic Anseriformes | H5N1 |
| A/hooded crane/Kagoshima/KU-5T/2021 (H5N8) | 2020-2022 | Japan | Other wild species | H5N8 |
| A/mute swan/Croatia/104/2021(H5N1) | 2020-2022 | East Europe | Wild Anseriformes | H5N1 |
| A/mute swan/Ireland/033945 22VIR1325-16/2021(H5N1) | 2020-2022 | West Europe | Wild Anseriformes | H5N1 |
| A/turkey/Ireland/033674 22VIR1325-19/2021(H5N1) | 2020-2022 | West Europe | Domestic Galliformes | H5N1 |
| A/turkey/Italy/21VIR9816-1/2021(H5N1) | 2020-2022 | West Europe | Domestic Galliformes | H5N1 |
| A/western jackdaw/Netherlands/21039297-002/2021(H5N1) | 2020-2022 | West Europe | Other wild species | H5N1 |
| A/Anser albifrons/Belgium/15465 0010/2021(H5N1) | 2020-2022 | West Europe | Wild Anseriformes | H5N1 |
| A/broiler/Ireland/033734 22VIR1325-20/2021(H5N1) | 2020-2022 | West Europe | Domestic Galliformes | H5N1 |
| A/chicken/Italy/21VIR9951-25/2021(H5N1) | 2020-2022 | West Europe | Domestic Galliformes | H5N1 |
| A/gadwall/Croatia/108/2021(H5N1) | 2020-2022 | East Europe | Wild Anseriformes | H5N1 |
| A/goose/Italy/IZSLT-21VIR10273/2021(H5N1) | 2020-2022 | West Europe | Domestic Anseriformes | H5N1 |
| A/partridge/Bulgaria/745 22VIR778-3/2021(H5N1) | 2020-2022 | East Europe | Other wild species | H5N1 |
| A/Wild goose/Italy/21VIR10193/2021(H5N1) | 2020-2022 | West Europe | Wild Anseriformes | H5N1 |
| A/chicken/Czech Republic/23404/2021(H5N1) | 2020-2022 | East Europe | Domestic Galliformes | H5N1 |
| A/chicken/Czech Republic/23404-2K/2021(H5N1) | 2020-2022 | East Europe | Domestic Galliformes | H5N1 |
| A/chicken/Czech Republic/23404-2T/2021(H5N1) | 2020-2022 | East Europe | Domestic Galliformes | H5N1 |
| A/chicken/Czech Republic/23404-4K/2021(H5N1) | 2020-2022 | East Europe | Domestic Galliformes | H5N1 |
| A/chicken/Czech Republic/23404-4T/2021(H5N1) | 2020-2022 | East Europe | Domestic Galliformes | H5N1 |
| A/chicken/France/21P013076/2021(H5N1) | 2020-2022 | West Europe | Domestic Galliformes | H5N1 |
| A/chicken/Italy/21VIR10239/2021(H5N1) | 2020-2022 | West Europe | Domestic Galliformes | H5N1 |
| A/goose/France/21P013228/2021(H5N1) | 2020-2022 | West Europe | Domestic Anseriformes | H5N1 |
| A/mute swan/Netherlands/21039824-002/2021(H5N1) | 2020-2022 | West Europe | Wild Anseriformes | H5N1 |
| A/turkey/Italy/21VIR10251/2021(H5N1) | 2020-2022 | West Europe | Domestic Galliformes | H5N1 |
| A/chicken/Italy/21VIR10352/2021(H5N1) | 2020-2022 | West Europe | Domestic Galliformes | H5N1 |
| A/goose/Czech Republic/23458-1K/2021(H5N1) | 2020-2022 | East Europe | Domestic Anseriformes | H5N1 |
| A/goose/Czech Republic/23458-2T/2021(H5N1) | 2020-2022 | East Europe | Domestic Anseriformes | H5N1 |
| A/goose/Czech Republic/23458-4T/2021(H5N1) | 2020-2022 | East Europe | Domestic Anseriformes | H5N1 |
| A/goose/Czech Republic/23458-5T/2021(H5N1) | 2020-2022 | East Europe | Domestic Anseriformes | H5N1 |
| A/mute swan/Romania/16790 21VIR11355/2021(H5N1) | 2020-2022 | East Europe | Wild Anseriformes | H5N1 |
| A/white-tailed eagle/Sweden/SVA211201SZ0380/FB004721/M-2021(H5N1) | 2020-2022 | West Europe | Other wild species | H5N1 |
| A/chicken/Kursk/132-1V/2021(H5N1) | 2020-2022 | East Europe | Domestic Galliformes | H5N1 |
| A/chicken/Italy/21VIR10343/2021(H5N1) | 2020-2022 | West Europe | Domestic Galliformes | H5N1 |
| A/great egret/Czech Republic/23609/2021(H5N1) | 2020-2022 | East Europe | Other wild species | H5N1 |
| A/grey heron/Czech Republic/23608/2021(H5N1) | 2020-2022 | East Europe | Other wild species | H5N1 |
| A/grey heron/Czech Republic/23608-1K/2021(H5N1) | 2020-2022 | East Europe | Other wild species | H5N1 |
| A/Mallard/Netherlands/5/2021(H5N1) | 2016-2017 | West Europe | Wild Anseriformes | H5N8 |
| A/chicken/Czech Republic/23589-1T/2021(H5N1) | 2020-2022 | East Europe | Domestic Galliformes | H5N1 |
| A/chicken/Czech Republic/23589-3/2021(H5N1) | 2020-2022 | East Europe | Domestic Galliformes | H5N1 |
| A/chicken/Czech Republic/23589-4/2021(H5N1) | 2020-2022 | East Europe | Domestic Galliformes | H5N1 |
| A/duck/Czech Republic/23589-1T/2021(H5N1) | 2020-2022 | East Europe | Domestic Anseriformes | H5N1 |
| A/layer/Ireland/034424 22VIR1325-21/2021(H5N1) | 2020-2022 | West Europe | Domestic Galliformes | H5N1 |
| A/Mallard/Netherlands/4/2021(H5N1) | 2020-2022 | West Europe | Wild Anseriformes | H5N1 |
| A/Mallard/Netherlands/6/2021(H5N1) | 2020-2022 | West Europe | Wild Anseriformes | H5N1 |
| A/Mallard/Netherlands/7/2021(H5N1) | 2020-2022 | West Europe | Wild Anseriformes | H5N1 |
| A/Mallard/Netherlands/8/2021(H5N1) | 2020-2022 | West Europe | Wild Anseriformes | H5N1 |
| A/seagull/Italy/21VIR10481-2/2021(H5N1) | 2020-2022 | West Europe | Charadriiformes | H5N1 |
| A/turkey/Italy/21VIR10340/2021(H5N1) | 2020-2022 | West Europe | Domestic Galliformes | H5N1 |
| A/chicken/Italy/21VIR10384/2021(H5N1) | 2020-2022 | West Europe | Domestic Galliformes | H5N1 |
| A/chicken/Italy/21VIR10388/2021(H5N1) | 2020-2022 | West Europe | Domestic Galliformes | H5N1 |
| A/chicken/Italy/21VIR10389/2021(H5N1) | 2020-2022 | West Europe | Domestic Galliformes | H5N1 |
| A/Chicken/Sweden/SVA211130SZ0427/FB290424-IP-1/M-2021(H5N1) | 2020-2022 | West Europe | Domestic Galliformes | H5N1 |
| A/kestrel/Italy/21VIR10468/2021(H5N1) | 2020-2022 | West Europe | Other wild species | H5N1 |
| A/turkey/Bulgaria/755-1 22VIR778-4/2021(H5N1) | 2020-2022 | East Europe | Domestic Galliformes | H5N1 |
| A/chicken/Vietnam/HU14-LB11/2021(H5N8) | 2020-2022 | West Central Asia | Domestic Galliformes | H5N8 |
| A/cignus olor/Italy/IZSLT 21VIR10529-1/2021(H5N1) | 2020-2022 | West Europe | Wild Anseriformes | H5N1 |
| A/duck/Bulgaria/756-4 22VIR778-6/2021(H5N1) | 2020-2022 | East Europe | Domestic Anseriformes | H5N1 |
| A/duck/Hunan/S40199/2021(H5N6) | 2020-2022 | China | Domestic Anseriformes | H5N6 |
| A/duck/Hunan/S40268/2021(H5N6) | 2020-2022 | China | Domestic Anseriformes | H5N6 |
| A/duck/Italy/21VIR10447/2021(H5N1) | 2020-2022 | West Europe | Domestic Anseriformes | H5N1 |
| A/turkey/Italy/21VIR10456/2021(H5N1) | 2020-2022 | West Europe | Domestic Galliformes | H5N1 |
| A/chicken/Stavropol/146-1V/2021(H5N1) | 2020-2022 | East Europe | Domestic Galliformes | H5N1 |
| A/duck/Guizhou/S4702/2021(H5N6) | 2020-2022 | China | Domestic Anseriformes | H5N6 |
| A/hen/Bulgaria/757-6 22VIR778-7/2021(H5N1) | 2020-2022 | East Europe | Domestic Galliformes | H5N1 |
| A/turkey/Kentucky/22-004546-001/2022(H5N1) | 2020-2022 | North America | Domestic Galliformes | H5N1 |
| A/turkey/Kentucky/22-004546-002/2022(H5N1) | 2020-2022 | North America | Domestic Galliformes | H5N1 |
| A/chicken/Italy/21VIR10573-1/2021(H5N1) | 2020-2022 | West Europe | Domestic Galliformes | H5N1 |
| A/duck/Zhejiang/S4854/2021(H5N6) | 2020-2022 | China | Domestic Anseriformes | H5N6 |
| A/egret/France/21P013418/2021(H5N1) | 2020-2022 | West Europe | Other wild species | H5N1 |
| A/turkey/Lisbon/1/2021(H5N1) | 2020-2022 | West Europe | Domestic Galliformes | H5N1 |
| A/chicken/Italy/21VIR10850/2021(H5N1) | 2020-2022 | West Europe | Domestic Galliformes | H5N1 |
| A/Gallus gallus/Belgium/15977/2021(H5N1) | 2020-2022 | West Europe | Domestic Galliformes | H5N1 |
| A/turkey/Ireland/035425 22VIR1325-17/2021(H5N1) | 2020-2022 | West Europe | Domestic Galliformes | H5N1 |
| A/turkey/Italy/21VIR10851/2021(H5N1) | 2020-2022 | West Europe | Domestic Galliformes | H5N1 |
| A/Gallus gallus/Belgium/16070 003/2021(H5N1) | 2020-2022 | West Europe | Domestic Galliformes | H5N1 |
| A/crow/Ireland/035624 22VIR1325-11/2021(H5N1) | 2020-2022 | West Europe | Other wild species | H5N1 |
| A/goose/Guangdong/S4751/2021(H5N6) | 2020-2022 | China | Domestic Anseriformes | H5N6 |
| A/grey heron/Croatia/132/2021(H5N1) | 2020-2022 | East Europe | Other wild species | H5N1 |
| A/swan/Romania/16905 22VIR2749-1/2021(H5N1) | 2020-2022 | East Europe | Wild Anseriformes | H5N1 |
| A/heron/Italy/21VIR10998-1/2021(H5N1) | 2020-2022 | West Europe | Other wild species | H5N1 |
| A/duck/Ireland/036105 22VIR1325-22/2021(H5N1) | 2020-2022 | West Europe | Domestic Anseriformes | H5N1 |
| A/Turkey/Sweden/SVA211212SZ0001/FB301013-IP-2/M-2021(H5N1) | 2020-2022 | West Europe | Domestic Galliformes | H5N1 |
| A/chicken/Rostov-on-Don/159-1V/2021(H5N1) | 2020-2022 | East Europe | Domestic Galliformes | H5N1 |
| A/chicken/Rostov-on-Don/159-2V/2021(H5N1) | 2020-2022 | East Europe | Domestic Galliformes | H5N1 |
| A/chicken/Rostov-on-Don/159-3V/2021(H5N1) | 2020-2022 | East Europe | Domestic Galliformes | H5N1 |
| A/chicken/Rostov-on-Don/159-4V/2021(H5N1) | 2020-2022 | East Europe | Domestic Galliformes | H5N1 |
| A/chicken/Rostov-on-Don/159-5V/2021(H5N1) | 2020-2022 | East Europe | Domestic Galliformes | H5N1 |
| A/chicken/Rostov-on-Don/159-6V/2021(H5N1) | 2020-2022 | East Europe | Domestic Galliformes | H5N1 |
| A/chicken/Rostov-on-Don/159-7V/2021(H5N1) | 2020-2022 | East Europe | Domestic Galliformes | H5N1 |
| A/little owl/Italy/21VIR11382-1/2021(H5N1) | 2020-2022 | West Europe | Other wild species | H5N1 |
| A/owl/Italy/21VIR11057/2021(H5N1) | 2020-2022 | West Europe | Other wild species | H5N1 |
| A/turkey/Italy/21VIR11053-1/2021(H5N1) | 2020-2022 | West Europe | Domestic Galliformes | H5N1 |
| A/pelican/France/21P013720/2021(H5N1) | 2020-2022 | West Europe | Other wild species | H5N1 |
| A/seagull/Italy/21VIR11259-10/2021(H5N1) | 2020-2022 | West Europe | Charadriiformes | H5N1 |
| A/seagull/Italy/21VIR11259-12/2021(H5N1) | 2020-2022 | West Europe | Charadriiformes | H5N1 |
| A/magpie/Italy/21VIR11656/2021(H5N1) | 2020-2022 | West Europe | Other wild species | H5N1 |
| A/duck/Ireland/036646 22VIR1325-18/2021(H5N1) | 2020-2022 | West Europe | Domestic Anseriformes | H5N1 |
| A/Herring Gull/Netherlands/1/2021(H5N1) | 2020-2022 | West Europe | Charadriiformes | H5N1 |
| A/goose/Czech Republic/25322-179/2021(H5N1) | 2020-2022 | East Europe | Domestic Anseriformes | H5N1 |
| A/goose/Czech Republic/25322-205/2021(H5N1) | 2020-2022 | East Europe | Domestic Anseriformes | H5N1 |
| A/goose/Czech Republic/25322-229/2021(H5N1) | 2020-2022 | East Europe | Domestic Anseriformes | H5N1 |
| A/grey heron/Czech Republic/25338-1/2021(H5N1) | 2020-2022 | East Europe | Other wild species | H5N1 |
| A/grey heron/Czech Republic/25338-2/2021(H5N1) | 2020-2022 | East Europe | Other wild species | H5N1 |
| A/mute swan/Czech Republic/25702-2/2021(H5N1) | 2020-2022 | East Europe | Wild Anseriformes | H5N1 |
| A/turkey/Italy/21VIR11507/2021(H5N1) | 2020-2022 | West Europe | Domestic Galliformes | H5N1 |
| A/laying hen/Italy/21VIR11502/2021(H5N1) | 2020-2022 | West Europe | Domestic Galliformes | H5N1 |
| A/Muscovy duck/England/074477/2021(H5N1) | 2020-2022 | West Europe | Wild Anseriformes | H5N1 |
| A/chicken/Czech Republic/25690/2021(H5N1) | 2020-2022 | East Europe | Domestic Galliformes | H5N1 |
| A/duck/Bangladesh/19D1818/2021(H5N1) | 2020-2022 | West Central Asia | Domestic Anseriformes | H5N1 |
| A/mute swan/Ireland/037311 22VIR1325-13/2021(H5N1) | 2020-2022 | West Europe | Wild Anseriformes | H5N1 |
| A/swan/Spain/4087-1 22VIR2142-1/2021(H5N1) | 2020-2022 | West Europe | Wild Anseriformes | H5N1 |
| A/Chicken/Hangzhou/E1149/2021(H5N6) | 2020-2022 | China | Domestic Galliformes | H5N6 |
| A/goose/France/21P014207/2021(H5N1) | 2020-2022 | West Europe | Domestic Anseriformes | H5N1 |
| A/turkey/Italy/21VIR11586-2/2021(H5N1) | 2020-2022 | West Europe | Domestic Galliformes | H5N1 |
| A/turkey/Italy/21VIR11591-8/2021(H5N1) | 2020-2022 | West Europe | Domestic Galliformes | H5N1 |
| A/pheasant/Czech Republic/25827-1/2021(H5N1) | 2020-2022 | East Europe | Other wild species | H5N1 |
| A/pheasant/Czech Republic/25827-2/2021(H5N1) | 2020-2022 | East Europe | Other wild species | H5N1 |
| A/pheasant/Czech Republic/25827-3/2021(H5N1) | 2020-2022 | East Europe | Other wild species | H5N1 |
| A/England/215201407/2021(H5N1) | 2020-2022 | West Europe | Human | H5N1 |
| A/rooster/Slovenia/2039 22VIR777-1/2021(H5N1) | 2020-2022 | East Europe | Other wild species | H5N1 |
| A/swan/Slovenia/2041 22VIR777-2/2021(H5N1) | 2020-2022 | East Europe | Wild Anseriformes | H5N1 |
| A/buzzard/Italy/21VIR11899-5/2021(H5N1) | 2020-2022 | West Europe | Other wild species | H5N1 |
| A/Gallus gallus/Belgium/17100 0001/2021(H5N1) | 2020-2022 | West Europe | Domestic Galliformes | H5N1 |
| A/Mallard/Netherlands/13/2021(H5N1) | 2020-2022 | West Europe | Wild Anseriformes | H5N1 |
| A/Mallard/Netherlands/14/2021(H5N1) | 2020-2022 | West Europe | Wild Anseriformes | H5N1 |
| A/Mallard/Netherlands/15/2021(H5N1) | 2020-2022 | West Europe | Wild Anseriformes | H5N1 |
| A/mute swan/Croatia/144/2021(H5N1) | 2020-2022 | East Europe | Wild Anseriformes | H5N1 |
| A/owl/Italy/21VIR11899-1/2021(H5N1) | 2020-2022 | West Europe | Other wild species | H5N1 |
| A/swan/Slovenia/2049 22VIR777-3/2021(H5N1) | 2020-2022 | East Europe | Wild Anseriformes | H5N1 |
| A/turkey/Italy/21VIR11803-1/2021(H5N1) | 2020-2022 | West Europe | Domestic Galliformes | H5N1 |
| A/turkey/Italy/21VIR11804-1/2021(H5N1) | 2020-2022 | West Europe | Domestic Galliformes | H5N1 |
| A/Barnacle Goose/Netherlands/6/2021(H5N1) | 2020-2022 | West Europe | Wild Anseriformes | H5N1 |
| A/Barnacle Goose/Netherlands/7/2021(H5N1) | 2020-2022 | West Europe | Wild Anseriformes | H5N1 |
| A/hen/Bulgaria/854-1 22VIR778-10/2021(H5N1) | 2020-2022 | East Europe | Domestic Galliformes | H5N1 |
| A/Mallard/Netherlands/17/2021(H5N3) | 2020-2022 | West Europe | Wild Anseriformes | H5N3 |
| A/mute swan/Croatia/145/2021(H5N1) | 2020-2022 | East Europe | Wild Anseriformes | H5N1 |
| A/mute swan/Croatia/146/2021(H5N1) | 2020-2022 | East Europe | Wild Anseriformes | H5N1 |
| A/swan/Slovenia/2060 22VIR777-4/2021(H5N1) | 2020-2022 | East Europe | Wild Anseriformes | H5N1 |
| A/turkey/Italy/21VIR11887-3/2021(H5N1) | 2020-2022 | West Europe | Domestic Galliformes | H5N1 |
| A/American blue-winged teal/South Carolina/AH0195150/2021(H5N1) | 2020-2022 | North America | Wild Anseriformes | H5N1 |
| A/American wigeon/South Carolina/AH0195145/2021(H5N1) | 2020-2022 | North America | Wild Anseriformes | H5N1 |
| A/seagull/Slovenia/2075 22VIR777-7/2021(H5N1) | 2020-2022 | East Europe | Charadriiformes | H5N1 |
| A/swan/Slovenia/2072 22VIR777-5/2021(H5N1) | 2020-2022 | East Europe | Wild Anseriformes | H5N1 |
| A/swan/Slovenia/2073 22VIR777-6/2021(H5N1) | 2020-2022 | East Europe | Wild Anseriformes | H5N1 |
| A/chicken/Czech Republic/61-1/2022(H5N1) | 2020-2022 | East Europe | Domestic Galliformes | H5N1 |
| A/chicken/Czech Republic/61-2/2022(H5N1) | 2020-2022 | East Europe | Domestic Galliformes | H5N1 |
| A/chicken/Niger/22VIR1409-13/2022(H5N1) | 2020-2022 | Africa | Domestic Galliformes | H5N1 |
| A/chicken/Niger/22VIR1409-23/2022(H5N1) | 2020-2022 | Africa | Domestic Galliformes | H5N1 |
| A/chicken/Niger/22VIR1409-5/2022(H5N1) | 2020-2022 | Africa | Domestic Galliformes | H5N1 |
| A/chicken/Niger/22VIR1409-9/2022(H5N1) | 2020-2022 | Africa | Domestic Galliformes | H5N1 |
| A/duck/Spain/570-2 22VIR2142-40/2022(H5N1) | 2020-2022 | West Europe | Domestic Anseriformes | H5N1 |
| A/quail/Niger/22VIR1409-28/2022(H5N1) | 2020-2022 | Africa | Domestic Galliformes | H5N1 |
| A/quail/Niger/22VIR1409-30/2022(H5N1) | 2020-2022 | Africa | Domestic Galliformes | H5N1 |
| A/common kestel/Israel/49-2/2022(H5N1) | 2020-2022 | West Central Asia | Other wild species | H5N1 |
| A/heron/Italy/22VIR1562/2022(H5N1) | 2020-2022 | West Europe | Other wild species | H5N1 |
| A/white-tailed eagle/Hokkaido/22-RU-WTE-2/2022(H5N1) | 2020-2022 | Japan | Other wild species | H5N1 |
| A/broiler/Italy/22VIR54-1/2022(H5N1) | 2020-2022 | West Europe | Domestic Galliformes | H5N1 |
| A/chicken/Czech Republic/63/2022(H5N1) | 2020-2022 | East Europe | Domestic Galliformes | H5N1 |
| A/chicken/England/000187/2022(H5N1) | 2020-2022 | West Europe | Domestic Galliformes | H5N1 |
| A/great black-backed gull/Netherlands/22000090-002/2022(H5N1) | 2020-2022 | West Europe | Charadriiformes | H5N1 |
| A/laying hen/Italy/22VIR48-1/2022(H5N1) | 2020-2022 | West Europe | Domestic Galliformes | H5N1 |
| A/laying hen/Moldova/68-1 22VIR638-1/2022(H5N1) | 2020-2022 | East Europe | Domestic Galliformes | H5N1 |
| A/laying hen/Moldova/68-2 22VIR638-2/2022(H5N1) | 2020-2022 | East Europe | Domestic Galliformes | H5N1 |
| A/swan/Slovenia/13 22VIR777-8/2022(H5N1) | 2020-2022 | East Europe | Wild Anseriformes | H5N1 |
| A/Barnacle goose/Netherlands/1/2022(H5N1) | 2020-2022 | West Europe | Wild Anseriformes | H5N1 |
| A/chicken/Ehime/TU11-2-2425/2022(H5N1) | 2020-2022 | Japan | Domestic Galliformes | H5N1 |
| A/chicken/Ehime/TU12-2-1617/2022(H5N1) | 2020-2022 | Japan | Domestic Galliformes | H5N1 |
| A/northern goshawk/Netherlands/22000305-002/2022(H5N1) | 2020-2022 | West Europe | Other wild species | H5N1 |
| A/Phasianus colchicus/Belgium/294/2022(H5N1) | 2020-2022 | West Europe | Other wild species | H5N1 |
| A/turkey/Italy/IZSLT 22VIR366-3/2022(H5N1) | 2020-2022 | West Europe | Domestic Galliformes | H5N1 |
| A/goose/Hunan/SE284/2022(H5N1) | 2020-2022 | China | Domestic Anseriformes | H5N1 |
| A/guinea fowl/Italy/22VIR205-4/2022(H5N1) | 2020-2022 | West Europe | Other wild species | H5N1 |
| A/laying hen/Italy/22VIR203-3/2022(H5N1) | 2020-2022 | West Europe | Domestic Galliformes | H5N1 |
| A/peregrine falcon/Ireland/000191 22VIR1325-15/2022(H5N1) | 2020-2022 | West Europe | Other wild species | H5N1 |
| A/turkey/Italy/22VIR171-2/2022(H5N1) | 2020-2022 | West Europe | Domestic Galliformes | H5N1 |
| A/mute swan/Croatia/6/2022 (H5N1) | 2016-2017 | East Europe | Wild Anseriformes | H5N8 |
| A/barnacle goose/Netherlands/22000419-002/2022(H5N1) | 2020-2022 | West Europe | Wild Anseriformes | H5N1 |
| A/chicken/England/002070/2022(H5N1) | 2020-2022 | West Europe | Domestic Galliformes | H5N1 |
| A/chicken/Kosovo/22-2 22VIR3124-13/2022(H5N8) | 2020-2022 | East Europe | Domestic Galliformes | H5N8 |
| A/Great black-backed gull/1/2022(H5N1) | 2020-2022 | West Europe | Charadriiformes | H5N1 |
| A/Larus argentatus/Belgium/595 0008/2022(H5N1) | 2020-2022 | West Europe | Charadriiformes | H5N1 |
| A/red knot/Netherlands/22000409-002/2022(H5N1) | 2020-2022 | West Europe | Charadriiformes | H5N1 |
| A/buzzard/Ireland/000656 22VIR1325-12/2022(H5N1) | 2020-2022 | West Europe | Other wild species | H5N1 |
| A/goose/Spain/65-3 22VIR2142-2/2022(H5N1) | 2020-2022 | West Europe | Domestic Anseriformes | H5N1 |
| A/American wigeon/North Carolina/AH0182517/2022(H5N1) | 2020-2022 | North America | Wild Anseriformes | H5N1 |
| A/American wigeon/North Carolina/AH0182954/2022(H5N1) | 2020-2022 | North America | Wild Anseriformes | H5N1 |
| A/chicken/Italy/22VIR234-1/2022(H5N1) | 2020-2022 | West Europe | Domestic Galliformes | H5N1 |
| A/gadwall/North Carolina/AH0182894/2022(H5N1) | 2020-2022 | North America | Wild Anseriformes | H5N1 |
| A/mallard/North Carolina/AH0182886/2022(H5N1) | 2020-2022 | North America | Wild Anseriformes | H5N1 |
| A/northern pintail/North Carolina/AH0182892/2022(H5N1) | 2020-2022 | North America | Wild Anseriformes | H5N1 |
| A/northern shoveler/North Carolina/AH0182913/2022(H5N1) | 2020-2022 | North America | Wild Anseriformes | H5N1 |
| A/chicken/Israel/88/2022(H5N1) | 2016-2017 | West Central Asia | Domestic Galliformes | H5N8 |
| A/chicken/Croatia/7/2022 (H5N1) | 2016-2017 | East Europe | Domestic Galliformes | H5N8 |
| A/duck/Hubei/SE128/2022(H5N1) | 2020-2022 | China | Domestic Anseriformes | H5N1 |
| A/duck/Hubei/SE220/2022(H5N1) | 2020-2022 | China | Domestic Anseriformes | H5N1 |
| A/Fox/Netherlands/EMC2/2022(H5N1) | 2020-2022 | West Europe | Mammal | H5N1 |
| A/goose/Spain/88-3 22VIR2142-4/2022(H5N1) | 2020-2022 | West Europe | Domestic Anseriformes | H5N1 |
| A/laying hen/Italy/22VIR204-1/2022(H5N1) | 2020-2022 | West Europe | Domestic Galliformes | H5N1 |
| A/mute swan/Czech Republic/785/2022(H5N1) | 2020-2022 | East Europe | Wild Anseriformes | H5N1 |
| A/Anser anser/Spain/88-3/2022(H5N1) | 2020-2022 | West Europe | Wild Anseriformes | H5N1 |
| A/Barnacle goose/Netherlands/2/2022(H5N1) | 2020-2022 | West Europe | Wild Anseriformes | H5N1 |
| A/Barnacle goose/Netherlands/3/2022(H5N1) | 2020-2022 | West Europe | Wild Anseriformes | H5N1 |
| A/Barnacle goose/Netherlands/4/2022(H5N1) | 2020-2022 | West Europe | Wild Anseriformes | H5N1 |
| A/broiler/Italy/22VIR278-3/2022(H5N1) | 2020-2022 | West Europe | Domestic Galliformes | H5N1 |
| A/gray heron/Spain/88-2 22VIR2142-3/2022(H5N1) | 2020-2022 | West Europe | Other wild species | H5N1 |
| A/Oystercatcher/Netherlands/1/2022(H5N1) | 2020-2022 | West Europe | Charadriiformes | H5N1 |
| A/chicken/Czech Republic/913/2022(H5N1) | 2020-2022 | East Europe | Domestic Galliformes | H5N1 |
| A/duck/Czech Republic/913/2022(H5N1) | 2020-2022 | East Europe | Domestic Anseriformes | H5N1 |
| A/goose/Czech Republic/913/2022(H5N1) | 2020-2022 | East Europe | Domestic Anseriformes | H5N1 |
| A/Great white pelican/Israel/123/2022(H5N1) | 2020-2022 | West Central Asia | Other wild species | H5N1 |
| A/turkey/England/004737/2022(H5N1) | 2020-2022 | West Europe | Domestic Galliformes | H5N1 |
| A/Anser anser/Spain/141-6/2022(H5N1) | 2020-2022 | West Europe | Wild Anseriformes | H5N1 |
| A/Barnacle goose/Netherlands/5/2022(H5N1) | 2020-2022 | West Europe | Wild Anseriformes | H5N1 |
| A/Barnacle goose/Netherlands/6/2022(H5N1) | 2020-2022 | West Europe | Wild Anseriformes | H5N1 |
| A/Black-headed gull/Netherlands/1/2022(H5N1) | 2020-2022 | West Europe | Charadriiformes | H5N1 |
| A/Caspian gull/Netherlands/1/2022(H5N1) | 2020-2022 | West Europe | Charadriiformes | H5N1 |
| A/chicken/Kosovo/22-8 22VIR3124-14/2022(H5N8) | 2020-2022 | East Europe | Domestic Galliformes | H5N8 |
| A/goose/Spain/141-9 22VIR2142-6/2022(H5N1) | 2020-2022 | West Europe | Domestic Anseriformes | H5N1 |
| A/Grey heron/Netherlands/1/2022(H5N1) | 2020-2022 | West Europe | Other wild species | H5N1 |
| A/hawk/Italy/22VIR428-5/2022(H5N1) | 2020-2022 | West Europe | Other wild species | H5N1 |
| A/hawk/Italy/22VIR428-7/2022(H5N1) | 2020-2022 | West Europe | Other wild species | H5N1 |
| A/Turkey/Spain/140-24/2022(H5N1) | 2020-2022 | West Europe | Domestic Galliformes | H5N1 |
| A/turkey/Spain/140-38 22VIR2142-19/2022(H5N1) | 2020-2022 | West Europe | Domestic Galliformes | H5N1 |
| A/Canada goose/Luxembourg/22012198/2022(H5N1) | 2020-2022 | West Europe | Wild Anseriformes | H5N1 |
| A/stork/Spain/234-2 22VIR2142-7/2022(H5N1) | 2020-2022 | West Europe | Other wild species | H5N1 |
| A/Little egret/Israel/172/2022(H5N1) | 2020-2022 | West Central Asia | Other wild species | H5N1 |
| A/chicken/Kosovo/22-9 22VIR3124-15/2022(H5N8) | 2020-2022 | East Europe | Domestic Galliformes | H5N8 |
| A/turkey/Kosovo/13-2 22VIR3124-31/2022(H5N8) | 2020-2022 | East Europe | Domestic Galliformes | H5N8 |
| A/crow/Hokkaido/0101Q044/2022 (H5N1) | 2020-2022 | Japan | Other wild species | H5N1 |
| A/crow/Hokkaido/0101Q045/2022(H5N1) | 2020-2022 | Japan | Other wild species | H5N1 |
| A/white-fronted Goose/Croatia/16/2022(H5N1) | 2020-2022 | East Europe | Wild Anseriformes | H5N1 |
| A/Greylag goose/Sweden/SVA220308SZ0382/FB000559/M-2022(H5N1) | 2020-2022 | West Europe | Wild Anseriformes | H5N1 |
| A/Larus canus/Belgium/1668 0019/2022(H5N1) | 2020-2022 | West Europe | Charadriiformes | H5N1 |
| A/chicken/Netherlands/22001401-001005/2022(H5N1) | 2020-2022 | West Europe | Domestic Galliformes | H5N1 |
| A/Fox/Netherlands/EMC3/2022(H5N1) | 2020-2022 | West Europe | Mammal | H5N1 |
| A/goose/Spain/239-1 22VIR2142-8/2022(H5N1) | 2020-2022 | West Europe | Domestic Anseriformes | H5N1 |
| A/Anser anser/Belgium/1809 0002/2022(H5N1) | 2020-2022 | West Europe | Wild Anseriformes | H5N1 |
| A/chicken/Poland/H071 22VIR2515-6/2022(H5N1) | 2020-2022 | East Europe | Domestic Galliformes | H5N1 |
| A/domestic duck/England/007588/2022(H5N1) | 2020-2022 | West Europe | Domestic Anseriformes | H5N1 |
| A/Lesser scaup/MD/-LC-EESC-024/2022(H5N1) | 2020-2022 | North America | Wild Anseriformes | H5N1 |
| A/buzzard/Italy/22VIR767-1/2022(H5N1) | 2020-2022 | West Europe | Other wild species | H5N1 |
| A/buzzard/Italy/22VIR767-2/2022(H5N1) | 2020-2022 | West Europe | Other wild species | H5N1 |
| A/pigeon/Germany-NW/AI00951/2022(H5N1) | 2020-2022 | West Europe | Domestic Galliformes | H5N1 |
| A/Tachybaptus ruficollis/Belgium/1234 0008/2022(H5N1) | 2020-2022 | West Europe | Other wild species | H5N1 |
| A/northern goshawk/Sweden/SVA220210SZ0305/FP000317/O-2022(H5N1) | 2020-2022 | West Europe | Other wild species | H5N1 |
| A/red knot/Germany-SH/AI01010/2022(H5N1) | 2020-2022 | West Europe | Charadriiformes | H5N1 |
| A/Anser anser domesticus/Belgium/1668 0016/2022(H5N1) | 2020-2022 | West Europe | Wild Anseriformes | H5N1 |
| A/Fox/Netherlands/EMC1/2022(H5N1) | 2020-2022 | West Europe | Mammal | H5N1 |
| A/goose/Spain/294-2 22VIR2142-9/2022(H5N1) | 2020-2022 | West Europe | Domestic Anseriformes | H5N1 |
| A/swan/Italy/22VIR1560/2022(H5N1) | 2020-2022 | West Europe | Wild Anseriformes | H5N1 |
| A/Barnacle Goose/Netherlands/8/2022(H5N1) | 2020-2022 | West Europe | Wild Anseriformes | H5N1 |
| A/mute swan/Czech Republic/2755/2022(H5N1) | 2020-2022 | East Europe | Wild Anseriformes | H5N1 |
| A/Sanderling/Netherlands/1/2022(H5N1) | 2020-2022 | West Europe | Charadriiformes | H5N1 |
| A/swan/Germany-BW/AI00996/2022(H5N2) | 2020-2022 | West Europe | Wild Anseriformes | H5N2 |
| A/swan/Germany-BW/AI00997/2022(H5N2) | 2020-2022 | West Europe | Wild Anseriformes | H5N2 |
| A/black-headed gull/England/306270/2022(H5N1) | 2020-2022 | West Europe | Charadriiformes | H5N1 |
| A/chicken/Spain/340-37 22VIR2142-20/2022(H5N1) | 2020-2022 | West Europe | Domestic Galliformes | H5N1 |
| A/chicken/Spain/340-37 22VIR6312-37/2022(H5N1) | 2020-2022 | West Europe | Domestic Galliformes | H5N1 |
| A/Common Gull/Netherlands/1/2022(H5N1) | 2020-2022 | West Europe | Charadriiformes | H5N1 |
| A/herring gull/Italy/22VIR1710-3/2022(H5N1) | 2020-2022 | West Europe | Charadriiformes | H5N1 |
| A/seagull/Netherlands/22002274-002/2022(H5N1) | 2020-2022 | West Europe | Charadriiformes | H5N1 |
| A/swan/Romania/10324 22VIR2749-2/2022(H5N1) | 2020-2022 | East Europe | Wild Anseriformes | H5N1 |
| A/turkey/Stavropol/211-12V/2022(H5N1) | 2020-2022 | East Europe | Domestic Galliformes | H5N1 |
| A/turkey/Stavropol/211-15V/2022(H5N1) | 2020-2022 | East Europe | Domestic Galliformes | H5N1 |
| A/turkey/Stavropol/211-18V/2022(H5N1) | 2020-2022 | East Europe | Domestic Galliformes | H5N1 |
| A/turkey/Stavropol/211-1V/2022(H5N1) | 2020-2022 | East Europe | Domestic Galliformes | H5N1 |
| A/turkey/Stavropol/211-4V/2022(H5N1) | 2020-2022 | East Europe | Domestic Galliformes | H5N1 |
| A/turkey/Stavropol/211-5V/2022(H5N1) | 2020-2022 | East Europe | Domestic Galliformes | H5N1 |
| A/turkey/Stavropol/211-7V/2022(H5N1) | 2020-2022 | East Europe | Domestic Galliformes | H5N1 |
| A/turkey/Stavropol/211-9V/2022(H5N1) | 2020-2022 | East Europe | Domestic Galliformes | H5N1 |
| A/Anser anser/Spain/750-3/2022(H5N1) | 2020-2022 | West Europe | Wild Anseriformes | H5N1 |
| A/Anser anser/Spain/750-4 22VIR6312-2/2022(H5N1) | 2020-2022 | West Europe | Wild Anseriformes | H5N1 |
| A/chicken/England/011981/2022(H5N1) | 2020-2022 | West Europe | Domestic Galliformes | H5N1 |
| A/Greylag Goose/Netherlands/1/2022(H5N1) | 2020-2022 | West Europe | Wild Anseriformes | H5N1 |
| A/Phalacrocorax carbo/Belgium/1734 0002/2022(H5N1) | 2020-2022 | West Europe | Other wild species | H5N1 |
| A/Bald Eagle/BC/OTH-33-36/2022 (H5N1) | 2020-2022 | North America | Other wild species | H5N1 |
| A/black-headed gull/England/388256/2022(H5N1) | 2020-2022 | West Europe | Charadriiformes | H5N1 |
| A/crow/Hokkaido/0101Q054/2022 (H5N1) | 2020-2022 | Japan | Other wild species | H5N1 |
| A/stork/Spain/442-8 22VIR2142-12/2022(H5N1) | 2020-2022 | West Europe | Other wild species | H5N1 |
| A/stork/Spain/538-2 22VIR2142-14/2022(H5N1) | 2020-2022 | West Europe | Other wild species | H5N1 |
| A/swan/Romania/10394 22VIR2749-3/2022(H5N1) | 2020-2022 | East Europe | Wild Anseriformes | H5N1 |
| A/Anser anser/Spain/512-2/2022(H5N1) | 2020-2022 | West Europe | Wild Anseriformes | H5N1 |
| A/Barnacle goose/Luxembourg/22033922/2022(H5N1) | 2020-2022 | West Europe | Wild Anseriformes | H5N1 |
| A/chicken/Czech Republic/2968/2022(H5N1) | 2020-2022 | East Europe | Domestic Galliformes | H5N1 |
| A/goose/Spain/512-2 22VIR2142-13/2022(H5N1) | 2020-2022 | West Europe | Domestic Anseriformes | H5N1 |
| A/duck/Korea/H125/2022(H5N1) | 2018-2019 | Korea | Domestic Anseriformes | H5N6 |
| A/goose/Poland/H124 22VIR2515-5/2022(H5N1) | 2020-2022 | East Europe | Domestic Anseriformes | H5N1 |
| A/turkey/Spain/455-83 22VIR2142-23/2022(H5N1) | 2020-2022 | West Europe | Domestic Galliformes | H5N1 |
| A/Turkey/Spain/455-96/2022(H5N1) | 2020-2022 | West Europe | Domestic Galliformes | H5N1 |
| A/turkey/Spain/455-96 22VIR2142-24/2022(H5N1) | 2020-2022 | West Europe | Domestic Galliformes | H5N1 |
| A/Barnacle Goose/Netherlands/10/2022(H5N1) | 2020-2022 | West Europe | Wild Anseriformes | H5N1 |
| A/Barnacle Goose/Netherlands/9/2022(H5N1) | 2020-2022 | West Europe | Wild Anseriformes | H5N1 |
| A/chicken/Spain/452-1/2022(H5N1) | 2020-2022 | West Europe | Domestic Galliformes | H5N1 |
| A/chicken/Spain/452-1 22VIR2142-21/2022(H5N1) | 2020-2022 | West Europe | Domestic Galliformes | H5N1 |
| A/chicken/Spain/452-17 22VIR2142-22/2022(H5N1) | 2020-2022 | West Europe | Domestic Galliformes | H5N1 |
| A/crow/Hokkaido/0102F043/2022(H5N1) | 2020-2022 | Japan | Other wild species | H5N1 |
| A/crow/Hokkaido/0102F046/2022(H5N1) | 2020-2022 | Japan | Other wild species | H5N1 |
| A/turkey/Indiana/22-003707-003/2022(H5N1) | 2020-2022 | North America | Domestic Galliformes | H5N1 |
| A/chicken/England/012967/2022(H5N1) | 2020-2022 | West Europe | Domestic Galliformes | H5N1 |
| A/crow/Hokkaido/0101Q056/2022 (H5N1) | 2020-2022 | Japan | Other wild species | H5N1 |
| A/crow/Hokkaido/0102F048/2022 (H5N1) | 2020-2022 | Japan | Other wild species | H5N1 |
| A/domestic duck/England/012973/2022(H5N1) | 2020-2022 | West Europe | Domestic Anseriformes | H5N1 |
| A/duck/Italy/22VIR1294-1/2022(H5N1) | 2020-2022 | West Europe | Domestic Anseriformes | H5N1 |
| A/duck/Italy/22VIR1295-1/2022(H5N1) | 2020-2022 | West Europe | Domestic Anseriformes | H5N1 |
| A/duck/Poland/H126 22VIR2515-4/2022(H5N1) | 2020-2022 | East Europe | Domestic Anseriformes | H5N1 |
| A/turkey/Spain/489-21 22VIR2142-25/2022(H5N1) | 2020-2022 | West Europe | Domestic Galliformes | H5N1 |
| A/Turkey/Spain/489-6/2022(H5N1) | 2020-2022 | West Europe | Domestic Galliformes | H5N1 |
| A/turkey/Spain/490-22 22VIR2142-26/2022(H5N1) | 2020-2022 | West Europe | Domestic Galliformes | H5N1 |
| A/Turkey/Spain/490-24/2022(H5N1) | 2020-2022 | West Europe | Domestic Galliformes | H5N1 |
| A/turkey/Spain/490-24 22VIR2142-27/2022(H5N1) | 2020-2022 | West Europe | Domestic Galliformes | H5N1 |
| A/Barnacle Goose/Netherlands/11/2022(H5N1) | 2020-2022 | West Europe | Wild Anseriformes | H5N1 |
| A/Barnacle Goose/Netherlands/12/2022(H5N1) | 2020-2022 | West Europe | Wild Anseriformes | H5N1 |
| A/chicken/Czech Republic/3306-2/2022(H5N1) | 2020-2022 | East Europe | Domestic Galliformes | H5N1 |
| A/crow/Hokkaido/0102M086/2022(H5N1) | 2020-2022 | Japan | Other wild species | H5N1 |
| A/duck/Czech Republic/3306-1/2022(H5N1) | 2020-2022 | East Europe | Domestic Anseriformes | H5N1 |
| A/Eurasian Curlew/Netherlands/1/2022(H5N1) | 2020-2022 | West Europe | Charadriiformes | H5N1 |
| A/Fox/Netherlands/EMC5/2022(H5N1) | 2020-2022 | West Europe | Mammal | H5N1 |
| A/mute swan/Croatia/26/2022 (H5N1) | 2020-2022 | East Europe | Wild Anseriformes | H5N1 |
| A/swan/Lithuania/1220PG1 22VIR7255-1/2022(H5N1) | 2020-2022 | East Europe | Wild Anseriformes | H5N1 |
| A/swan/Romania/10455 22VIR2749-4/2022(H5N1) | 2020-2022 | East Europe | Wild Anseriformes | H5N1 |
| A/Turkey/Spain/540-26/2022(H5N1) | 2020-2022 | West Europe | Domestic Galliformes | H5N1 |
| A/turkey/Spain/540-26 22VIR2142-28/2022(H5N1) | 2020-2022 | West Europe | Domestic Galliformes | H5N1 |
| A/gray heron/Spain/602-1 22VIR2142-17/2022(H5N1) | 2020-2022 | West Europe | Other wild species | H5N1 |
| A/laying hen/Romania/10470 22VIR2749-5/2022(H5N1) | 2020-2022 | East Europe | Domestic Galliformes | H5N1 |
| A/swan/Poland/MB078 22VIR2515-7/2022(H5N1) | 2020-2022 | East Europe | Wild Anseriformes | H5N1 |
| A/white-tailed eagle/Hokkaido/20220210001/2022(H5N1) | 2020-2022 | Japan | Other wild species | H5N1 |
| A/buzzard/Germany-BB/AI01212/2022(H5N1) | 2020-2022 | West Europe | Other wild species | H5N1 |
| A/chicken/Kentucky/22-004416-001/2022(H5N1) | 2020-2022 | North America | Domestic Galliformes | H5N1 |
| A/chicken/Kentucky/22-004416-002/2022(H5N1) | 2020-2022 | North America | Domestic Galliformes | H5N1 |
| A/chicken/Kentucky/22-004416-003/2022(H5N1) | 2020-2022 | North America | Domestic Galliformes | H5N1 |
| A/chicken/Spain/562-1 22VIR2142-29/2022(H5N1) | 2020-2022 | West Europe | Domestic Galliformes | H5N1 |
| A/chicken/Spain/564-11/2022(H5N1) | 2020-2022 | West Europe | Domestic Galliformes | H5N1 |
| A/chicken/Spain/564-11 22VIR2142-31/2022(H5N1) | 2020-2022 | West Europe | Domestic Galliformes | H5N1 |
| A/chicken/Spain/564-4 22VIR2142-30/2022(H5N1) | 2020-2022 | West Europe | Domestic Galliformes | H5N1 |
| A/chicken/Virginia/22-004415-001/2022(H5N1) | 2020-2022 | North America | Domestic Galliformes | H5N1 |
| A/Mallard/Netherlands/1/2022(H5N1) | 2016-2017 | West Europe | Wild Anseriformes | H5N8 |
| A/bald eagle/Florida/W22-134-OP/2022(H5N1) | 2020-2022 | North America | Other wild species | H5N1 |
| A/turkey/Kentucky/22-004546-001/2022(H5N1) | 2020-2022 | North America | Domestic Galliformes | H5N1 |
| A/turkey/Kentucky/22-004546-002/2022(H5N1) | 2020-2022 | North America | Domestic Galliformes | H5N1 |
| A/Anser anser/Spain/638-6 22VIR6312-39/2022(H5N1) | 2020-2022 | West Europe | Wild Anseriformes | H5N1 |
| A/chicken/Spain/587-1/2022(H5N1) | 2020-2022 | West Europe | Domestic Galliformes | H5N1 |
| A/chicken/Spain/587-1 22VIR2142-33/2022(H5N1) | 2020-2022 | West Europe | Domestic Galliformes | H5N1 |
| A/chicken/Spain/622-8/2022(H5N1) | 2020-2022 | West Europe | Domestic Galliformes | H5N1 |
| A/chicken/Spain/622-8 22VIR2142-34/2022(H5N1) | 2020-2022 | West Europe | Domestic Galliformes | H5N1 |
| A/common crane/Spain/597-2 22VIR2142-15/2022(H5N1) | 2020-2022 | West Europe | Other wild species | H5N1 |
| A/crow/Hokkaido/0101Q061/2022 (H5N1) | 2020-2022 | Japan | Other wild species | H5N1 |
| A/crow/Hokkaido/0102L010/2022(H5N1) | 2020-2022 | Japan | Other wild species | H5N1 |
| A/fox/Ireland/3866 22VIR2064-1/2022(H5N1) | 2020-2022 | West Europe | Mammal | H5N1 |
| A/goose/Ireland/3869 22VIR2064-2/2022(H5N1) | 2020-2022 | West Europe | Domestic Anseriformes | H5N1 |
| A/goose/Italy/22VIR1520/2022(H5N1) | 2020-2022 | West Europe | Domestic Anseriformes | H5N1 |
| A/laying hen/Italy/22VIR1521-2/2022(H5N1) | 2020-2022 | West Europe | Domestic Galliformes | H5N1 |
| A/Mallard/Netherlands/2/2022(H5N1) | 2020-2022 | West Europe | Wild Anseriformes | H5N1 |
| A/turkey/Indiana/22-004688-002/2022(H5N1) | 2020-2022 | North America | Domestic Galliformes | H5N1 |
| A/turkey/Indiana/22-004688-003/2022(H5N1) | 2020-2022 | North America | Domestic Galliformes | H5N1 |
| A/turkey/Indiana/22-004688-006/2022(H5N1) | 2020-2022 | North America | Domestic Galliformes | H5N1 |
| A/Turkey/Spain/586-4/2022(H5N1) | 2020-2022 | West Europe | Domestic Galliformes | H5N1 |
| A/turkey/Spain/586-4 22VIR2142-32/2022(H5N1) | 2020-2022 | West Europe | Domestic Galliformes | H5N1 |
| A/chicken/Kosovo/22-50 22VIR3124-19/2022(H5N8) | 2020-2022 | East Europe | Domestic Galliformes | H5N8 |
| A/Fox/Netherlands/EMC6/2022(H5N1) | 2020-2022 | West Europe | Mammal | H5N1 |
| A/Turkey/Spain/645-1/2022(H5N1) | 2020-2022 | West Europe | Domestic Galliformes | H5N1 |
| A/turkey/Spain/645-1 22VIR2142-36/2022(H5N1) | 2020-2022 | West Europe | Domestic Galliformes | H5N1 |
| A/turkey/Spain/646-7 22VIR2142-37/2022(H5N1) | 2020-2022 | West Europe | Domestic Galliformes | H5N1 |
| A/chicken/Spain/644-8 22VIR2142-35/2022(H5N1) | 2020-2022 | West Europe | Domestic Galliformes | H5N1 |
| A/chicken/Spain/649-4/2022(H5N1) | 2020-2022 | West Europe | Domestic Galliformes | H5N1 |
| A/chicken/Spain/649-6 22VIR2142-38/2022(H5N1) | 2020-2022 | West Europe | Domestic Galliformes | H5N1 |
| A/turkey/Indiana/22-005289-001/2022(H5N1) | 2020-2022 | North America | Domestic Galliformes | H5N1 |
| A/turkey/Indiana/22-005289-002/2022(H5N1) | 2020-2022 | North America | Domestic Galliformes | H5N1 |
| A/Anser anser/Spain/638-6/2022(H5N1) | 2020-2022 | West Europe | Wild Anseriformes | H5N1 |
| A/Branta leucopsis/Belgium/2606 0009/2022(H5N1) | 2020-2022 | West Europe | Wild Anseriformes | H5N1 |
| A/chicken/Maine/22-005158-001/2022(H5N1) | 2020-2022 | North America | Domestic Galliformes | H5N1 |
| A/chicken/Spain/642-1/2022(H5N1) | 2020-2022 | West Europe | Domestic Galliformes | H5N1 |
| A/swan/Poland/MB083 22VIR2515-8/2022(H5N1) | 2020-2022 | East Europe | Wild Anseriformes | H5N1 |
| A/chicken/Italy/22VIR1694-5/2022(H5N1) | 2020-2022 | West Europe | Domestic Galliformes | H5N1 |
| A/chicken/Poland/H157 22VIR2515-3/2022(H5N1) | 2020-2022 | East Europe | Domestic Galliformes | H5N1 |
| A/turkey/Indiana/22-005328-001/2022(H5N1) | 2020-2022 | North America | Domestic Galliformes | H5N1 |
| A/turkey/Indiana/22-005328-002/2022(H5N1) | 2020-2022 | North America | Domestic Galliformes | H5N1 |
| A/Buteo buteo/Belgium/2606 0006/2022(H5N1) | 2020-2022 | West Europe | Other wild species | H5N1 |
| A/chicken/Maine/22-005443-001/2022(H5N1) | 2020-2022 | North America | Domestic Galliformes | H5N1 |
| A/turkey/England/016515/2022(H5N1) | 2020-2022 | West Europe | Domestic Galliformes | H5N1 |
| A/chicken/Delaware/22-005260-001/2022(H5N1) | 2020-2022 | North America | Domestic Galliformes | H5N1 |
| A/chicken/Delaware/22-005260-002/2022(H5N1) | 2020-2022 | North America | Domestic Galliformes | H5N1 |
| A/chicken/Delaware/22-005260-003/2022(H5N1) | 2020-2022 | North America | Domestic Galliformes | H5N1 |
| A/Ciconia ciconia/Spain/853-14 22VIR6312-42/2022(H5N1) | 2020-2022 | West Europe | Other wild species | H5N1 |
| A/turkey/Indiana/22-005696-001/2022(H5N1) | 2020-2022 | North America | Domestic Galliformes | H5N1 |
| A/turkey/Indiana/22-005696-002/2022(H5N1) | 2020-2022 | North America | Domestic Galliformes | H5N1 |
| A/turkey/Indiana/22-005696-003/2022(H5N1) | 2020-2022 | North America | Domestic Galliformes | H5N1 |
| A/Turkey/Spain/711-5/2022(H5N1) | 2020-2022 | West Europe | Domestic Galliformes | H5N1 |
| A/turkey/Spain/711-5 22VIR6312-23/2022(H5N1) | 2020-2022 | West Europe | Domestic Galliformes | H5N1 |
| A/Turkey/Spain/712-7/2022(H5N1) | 2020-2022 | West Europe | Domestic Galliformes | H5N1 |
| A/turkey/Spain/712-7 22VIR6312-24/2022(H5N1) | 2020-2022 | West Europe | Domestic Galliformes | H5N1 |
| A/bald eagle/North Carolina/W22-140/2022(H5N1) | 2020-2022 | North America | Other wild species | H5N1 |
| A/chicken/Italy/22VIR1953-3/2022(H5N1) | 2020-2022 | West Europe | Domestic Galliformes | H5N1 |
| A/chicken/Italy/22VIR1953-4/2022(H5N1) | 2020-2022 | West Europe | Domestic Galliformes | H5N1 |
| A/chicken/Michigan/22-005440-002/2022(H5N1) | 2020-2022 | North America | Domestic Galliformes | H5N1 |
| A/Ciconia ciconia/Spain/485-2 22VIR6312-1/2022(H5N1) | 2020-2022 | West Europe | Other wild species | H5N1 |
| A/duck/Guizhou/S1321/2022(H5N1) | 2020-2022 | China | Domestic Anseriformes | H5N1 |
| A/goose/Guizhou/S1541/2022(H5N1) | 2020-2022 | China | Domestic Anseriformes | H5N1 |
| A/guinea fowl/Michigan/22-005440-003/2022(H5N1) | 2020-2022 | North America | Other wild species | H5N1 |
| A/peacock/Italy/22VIR1953-2/2022(H5N1) | 2020-2022 | West Europe | Other wild species | H5N1 |
| A/pheasant/Italy/22VIR1953-1/2022(H5N1) | 2020-2022 | West Europe | Other wild species | H5N1 |
| A/pheasant/New York/22-005647-001/2022(H5N1) | 2020-2022 | North America | Other wild species | H5N1 |
| A/pheasant/New York/22-005647-002/2022(H5N1) | 2020-2022 | North America | Other wild species | H5N1 |
| A/stork/Spain/729-1 22VIR2142-18/2022(H5N1) | 2020-2022 | West Europe | Other wild species | H5N1 |
| A/swan/Romania/10656 22VIR2749-6/2022(H5N1) | 2020-2022 | East Europe | Wild Anseriformes | H5N1 |
| A/turkey/Michigan/22-005440-001/2022(H5N1) | 2020-2022 | North America | Domestic Galliformes | H5N1 |
| A/Great black-backed Gull/Netherlands/2/2022(H5N1) | 2016-2017 | West Europe | Charadriiformes | H5N8 |
| A/Anser anser/Spain/810-6 22VIR8632-16/2022(H5N1) | 2020-2022 | West Europe | Wild Anseriformes | H5N1 |
| A/Barnacle Goose/Netherlands/13/2022(H5N1) | 2020-2022 | West Europe | Wild Anseriformes | H5N1 |
| A/Barnacle Goose/Netherlands/14/2022(H5N1) | 2020-2022 | West Europe | Wild Anseriformes | H5N1 |
| A/broiler/Italy/22VIR1892-3/2022(H5N1) | 2020-2022 | West Europe | Domestic Galliformes | H5N1 |
| A/Caspian Gull/Netherlands/2/2022(H5N1) | 2020-2022 | West Europe | Charadriiformes | H5N1 |
| A/swan/Romania/10678 22VIR2749-7/2022(H5N1) | 2020-2022 | East Europe | Wild Anseriformes | H5N1 |
| A/turkey/Spain/801-6 22VIR6312-25/2022(H5N1) | 2020-2022 | West Europe | Domestic Galliformes | H5N1 |
| A/pelican/Greece/41 AL1 22VIR3126-2/2022(H5N1) | 2020-2022 | East Europe | Other wild species | H5N1 |
| A/pelican/Greece/41 AL2 22VIR3126-3/2022(H5N1) | 2020-2022 | East Europe | Other wild species | H5N1 |
| A/pelican/Greece/41-TR-313 22VIR3126-1/2022(H5N1) | 2020-2022 | East Europe | Other wild species | H5N1 |
| A/Barnacle Goose/Netherlands/15/2022(H5N1) | 2020-2022 | West Europe | Wild Anseriformes | H5N1 |
| A/chicken/Spain/806-7 22VIR6312-41/2022(H5N1) | 2020-2022 | West Europe | Domestic Galliformes | H5N1 |
| A/European Herring Gull/Netherlands/2/2022(H5N1) | 2020-2022 | West Europe | Charadriiformes | H5N1 |
| A/turkey/Spain/802-7 22VIR6312-26/2022(H5N1) | 2020-2022 | West Europe | Domestic Galliformes | H5N1 |
| A/turkey/Spain/803-5 22VIR6312-27/2022(H5N1) | 2020-2022 | West Europe | Domestic Galliformes | H5N1 |
| A/Turkey/Spain/803-7/2022(H5N1) | 2020-2022 | West Europe | Domestic Galliformes | H5N1 |
| A/turkey/Spain/805-8 22VIR6312-28/2022(H5N1) | 2020-2022 | West Europe | Domestic Galliformes | H5N1 |
| A/bald eagle/Florida/W22-153A/2022(H5N1) | 2020-2022 | North America | Other wild species | H5N1 |
| A/bald eagle/Florida/W22-153B/2022(H5N1) | 2020-2022 | North America | Other wild species | H5N1 |
| A/chicken/Connecticut/22-006118-001/2022(H5N1) | 2020-2022 | North America | Domestic Galliformes | H5N1 |
| A/chicken/Poland/H182 22VIR2515-1/2022(H5N2) | 2020-2022 | East Europe | Domestic Galliformes | H5N2 |
| A/Ciconia ciconia/Spain/971-6 22VIR6312-7/2022(H5N1) | 2020-2022 | West Europe | Other wild species | H5N1 |
| A/silkie chicken/Iowa/22-006114-001/2022(H5N1) | 2020-2022 | North America | Other wild species | H5N1 |
| A/turkey/Indiana/22-006259-001/2022(H5N1) | 2020-2022 | North America | Domestic Galliformes | H5N1 |
| A/turkey/Indiana/22-006259-002/2022(H5N1) | 2020-2022 | North America | Domestic Galliformes | H5N1 |
| A/bald eagle/Georgia/W22-202/2022(H5N1) | 2020-2022 | West Central Asia | Other wild species | H5N1 |
| A/bald eagle/South Carolina/W22-205/2022(H5N1) | 2020-2022 | North America | Other wild species | H5N1 |
| A/chicken/Albania/D381-22 22VIR3125-1/2022(H5N8) | 2020-2022 | East Europe | Domestic Galliformes | H5N8 |
| A/chicken/Kosovo/22-59 22VIR3124-20/2022(H5N8) | 2020-2022 | East Europe | Domestic Galliformes | H5N8 |
| A/Common buzzard/Scotland/020817/2022(H5N1) | 2020-2022 | West Europe | Other wild species | H5N8 |
| A/gull/Florida/W22-162/2022(H5N1) | 2020-2022 | North America | Charadriiformes | H5N1 |
| A/hooded merganser/Florida/W22-154/2022(H5N1) | 2020-2022 | North America | Wild Anseriformes | H5N1 |
| A/pelecanus crispus/Albania/D383-22 22VIR3125-2/2022(H5N1) | 2020-2022 | East Europe | Other wild species | H5N1 |
| A/turkey/Indiana/22-006261-001/2022(H5N1) | 2020-2022 | North America | Domestic Galliformes | H5N1 |
| A/turkey/Indiana/22-006261-002/2022(H5N1) | 2020-2022 | North America | Domestic Galliformes | H5N1 |
| A/Turkey/Spain/830-5/2022(H5N1) | 2020-2022 | West Europe | Domestic Galliformes | H5N1 |
| A/turkey/Spain/830-5 22VIR6312-29/2022(H5N1) | 2020-2022 | West Europe | Domestic Galliformes | H5N1 |
| A/Anser anser/Spain/863-2 22VIR6312-6/2022(H5N1) | 2020-2022 | West Europe | Wild Anseriformes | H5N1 |
| A/Ardea cinerea/Spain/863-1 22VIR6312-5/2022(H5N1) | 2020-2022 | West Europe | Other wild species | H5N1 |
| A/chicken/Missouri/22-006569-001/2022(H5N1) | 2020-2022 | North America | Domestic Galliformes | H5N1 |
| A/chicken/Missouri/22-006569-002/2022(H5N1) | 2020-2022 | North America | Domestic Galliformes | H5N1 |
| A/crow/Hokkaido/0102L015/2022 (H5N1) | 2020-2022 | Japan | Other wild species | H5N1 |
| A/duck/Poland/H188 22VIR2515-2/2022(H5N1) | 2020-2022 | East Europe | Domestic Anseriformes | H5N1 |
| A/Turkey/Spain/805-8/2022(H5N1) | 2020-2022 | West Europe | Domestic Galliformes | H5N1 |
| A/Turkey/Spain/859-8/2022(H5N1) | 2020-2022 | West Europe | Domestic Galliformes | H5N1 |
| A/turkey/Spain/859-8 22VIR6312-30/2022(H5N1) | 2020-2022 | West Europe | Domestic Galliformes | H5N1 |
| A/bald eagle/Florida/W22-189/2022(H5N1) | 2020-2022 | North America | Other wild species | H5N1 |
| A/Barnacle Goose/Netherlands/16/2022(H5N1) | 2020-2022 | West Europe | Wild Anseriformes | H5N1 |
| A/Barnacle Goose/Netherlands/17/2022(H5N1) | 2020-2022 | West Europe | Wild Anseriformes | H5N1 |
| A/Brent goose/Netherlands/1/2022(H5N1) | 2020-2022 | West Europe | Wild Anseriformes | H5N1 |
| A/chicken/Anhui/S1740/2022(H5N1) | 2020-2022 | China | Domestic Galliformes | H5N1 |
| A/chicken/Maryland/22-006578-001/2022(H5N1) | 2020-2022 | North America | Domestic Galliformes | H5N1 |
| A/chicken/Maryland/22-006578-001-original/2022(H5N1) | 2020-2022 | North America | Domestic Galliformes | H5N1 |
| A/chicken/Maryland/22-006578-002/2022(H5N1) | 2020-2022 | North America | Domestic Galliformes | H5N1 |
| A/chicken/Maryland/22-006578-002-original/2022(H5N1) | 2020-2022 | North America | Domestic Galliformes | H5N1 |
| A/chicken/Missouri/22-006639-001/2022(H5N1) | 2020-2022 | North America | Domestic Galliformes | H5N1 |
| A/chicken/Missouri/22-006639-001-original/2022(H5N1) | 2020-2022 | North America | Domestic Galliformes | H5N1 |
| A/chicken/Spain/897-7 22VIR6312-31/2022(H5N1) | 2020-2022 | West Europe | Domestic Galliformes | H5N1 |
| A/chicken/Spain/897-8/2022(H5N1) | 2020-2022 | West Europe | Domestic Galliformes | H5N1 |
| A/Barnacle Goose/Netherlands/18/2022(H5N1) | 2020-2022 | West Europe | Wild Anseriformes | H5N1 |
| A/chicken/Maryland/22-006948-001-original/2022(H5N1) | 2020-2022 | North America | Domestic Galliformes | H5N1 |
| A/chicken/Spain/899-7/2022(H5N1) | 2020-2022 | West Europe | Domestic Galliformes | H5N1 |
| A/chicken/Spain/899-7 22VIR6312-32/2022(H5N1) | 2020-2022 | West Europe | Domestic Galliformes | H5N1 |
| A/snow goose/Kansas/W22-177B/2022(H5N1) | 2020-2022 | North America | Wild Anseriformes | H5N1 |
| A/turkey/South Dakota/22-006792-001/2022(H5N1) | 2020-2022 | North America | Domestic Galliformes | H5N1 |
| A/turkey/South Dakota/22-006792-001-original/2022(H5N1) | 2020-2022 | North America | Domestic Galliformes | H5N1 |
| A/turkey/South Dakota/22-006792-002/2022(H5N1) | 2020-2022 | North America | Domestic Galliformes | H5N1 |
| A/turkey/South Dakota/22-006792-002-original/2022(H5N1) | 2020-2022 | North America | Domestic Galliformes | H5N1 |
| A/Chicken/BC/FAV-0369-OS/2022(H5N1) | 2020-2022 | North America | Domestic Galliformes | H5N1 |
| A/European polecat/Netherlands/1/2022(H5N1) | 2020-2022 | West Europe | Mammal | H5N1 |
| A/bald eagle/Kansas/W22-185/2022(H5N1) | 2020-2022 | North America | Other wild species | H5N1 |
| A/chicken/Spain/924-3/2022(H5N1) | 2020-2022 | West Europe | Domestic Galliformes | H5N1 |
| A/chicken/Spain/924-3 22VIR6312-33/2022(H5N1) | 2020-2022 | West Europe | Domestic Galliformes | H5N1 |
| A/Graylag goose/Netherlands/2/20222(H5N1) | 2020-2022 | West Europe | Wild Anseriformes | H5N1 |
| A/snow goose/Kansas/W22-174B/2022(H5N1) | 2020-2022 | North America | Wild Anseriformes | H5N1 |
| A/turkey/Iowa/22-006795-001/2022(H5N1) | 2020-2022 | North America | Domestic Galliformes | H5N1 |
| A/turkey/Iowa/22-006795-001-original/2022(H5N1) | 2020-2022 | North America | Domestic Galliformes | H5N1 |
| A/turkey/Iowa/22-006795-002/2022(H5N1) | 2020-2022 | North America | Domestic Galliformes | H5N1 |
| A/turkey/Iowa/22-006795-002-original/2022(H5N1) | 2020-2022 | North America | Domestic Galliformes | H5N1 |
| A/chicken/Delaware/22-006945-001-original/2022(H5N1) | 2020-2022 | North America | Domestic Galliformes | H5N1 |
| A/chicken/Delaware/22-006945-002-original/2022(H5N1) | 2020-2022 | North America | Domestic Galliformes | H5N1 |
| A/chicken/Maryland/22-007086-001-original/2022(H5N1) | 2020-2022 | North America | Domestic Galliformes | H5N1 |
| A/Greylag goose/Scotland/024915/2022(H5N1) | 2020-2022 | West Europe | Wild Anseriformes | H5N1 |
| A/ring-billed gull/Florida/W22-169/2022(H5N1) | 2020-2022 | North America | Charadriiformes | H5N1 |
| A/swan/Romania/10986 22VIR2749-8/2022(H5N1) | 2020-2022 | East Europe | Wild Anseriformes | H5N1 |
| A/turkey/Missouri/22-006944-002-original/2022(H5N1) | 2020-2022 | North America | Domestic Galliformes | H5N1 |
| A/bald eagle/Florida/W22-191/2022(H5N1) | 2020-2022 | North America | Other wild species | H5N1 |
| A/bald eagle/Georgia/W22-194A/2022(H5N1) | 2020-2022 | West Central Asia | Other wild species | H5N1 |
| A/bald eagle/North Carolina/W22-186/2022(H5N1) | 2020-2022 | North America | Other wild species | H5N1 |
| A/chicken/Maryland/22-007273-001-original/2022(H5N1) | 2020-2022 | North America | Domestic Galliformes | H5N1 |
| A/chicken/Maryland/22-007273-002-original/2022(H5N1) | 2020-2022 | North America | Domestic Galliformes | H5N1 |
| A/chicken/Spain/942-8/2022(H5N1) | 2020-2022 | West Europe | Domestic Galliformes | H5N1 |
| A/chicken/Spain/942-8 22VIR6312-34/2022(H5N1) | 2020-2022 | West Europe | Domestic Galliformes | H5N1 |
| A/turkey/Missouri/22-007087-001-original/2022(H5N1) | 2020-2022 | North America | Domestic Galliformes | H5N1 |
| A/turkey/Missouri/22-007087-002-original/2022(H5N1) | 2020-2022 | North America | Domestic Galliformes | H5N1 |
| A/duck/Bangladesh/19D1874/2022(H5N1) | 2020-2022 | West Central Asia | Domestic Anseriformes | H5N1 |
| A/guinea fowl/Illinois/22-007382-001-original/2022(H5N1) | 2020-2022 | North America | Other wild species | H5N1 |
| A/poultry/Kansas/22-007391-001-original/2022(H5N1) | 2020-2022 | North America | Domestic Galliformes | H5N1 |
| A/poultry/Kansas/22-007391-002-original/2022(H5N1) | 2020-2022 | North America | Domestic Galliformes | H5N1 |
| A/chicken/Iowa/22-007376-001-original/2022(H5N1) | 2020-2022 | North America | Domestic Galliformes | H5N1 |
| A/chicken/Iowa/22-007376-002-original/2022(H5N1) | 2020-2022 | North America | Domestic Galliformes | H5N1 |
| A/chicken/Maine/22-007410-002-original/2022(H5N1) | 2020-2022 | North America | Domestic Galliformes | H5N1 |
| A/Gallus gallus/Belgium/3194 0001/2022(H5N1) | 2020-2022 | West Europe | Domestic Galliformes | H5N1 |
| A/pelican/Greece/64 KI 22VIR3126-6/2022(H5N1) | 2020-2022 | East Europe | Other wild species | H5N1 |
| A/pelican/Greece/64 LI 22VIR3126-8/2022(H5N1) | 2020-2022 | East Europe | Other wild species | H5N1 |
| A/pelican/Greece/64 SP 22VIR3126-7/2022(H5N1) | 2020-2022 | East Europe | Other wild species | H5N1 |
| A/pelican/Greece/64 TR 22VIR3126-9/2022(H5N1) | 2020-2022 | East Europe | Other wild species | H5N1 |
| A/turkey/Maine/22-007410-001-original/2022(H5N1) | 2020-2022 | North America | Domestic Galliformes | H5N1 |
| A/chicken/Maine/22-007582-001-original/2022(H5N1) | 2020-2022 | North America | Domestic Galliformes | H5N1 |
| A/turkey/South Dakota/22-007534-001-original/2022(H5N1) | 2020-2022 | North America | Domestic Galliformes | H5N1 |
| A/turkey/South Dakota/22-007534-002-original/2022(H5N1) | 2020-2022 | North America | Domestic Galliformes | H5N1 |
| A/bald eagle/North Carolina/W22-229/2022(H5N1) | 2020-2022 | North America | Other wild species | H5N1 |
| A/pelican/Greece/69 CL 22VIR3126-10/2022(H5N1) | 2020-2022 | East Europe | Other wild species | H5N1 |
| A/snow goose/Kansas/W22-199B/2022(H5N1) | 2020-2022 | North America | Wild Anseriformes | H5N1 |
| A/snow goose/Kansas/W22-199C/2022(H5N1) | 2020-2022 | North America | Wild Anseriformes | H5N1 |
| A/snow goose/Kansas/W22-199D/2022(H5N1) | 2020-2022 | North America | Wild Anseriformes | H5N1 |
| A/snow goose/Kansas/W22-199E/2022(H5N1) | 2020-2022 | North America | Wild Anseriformes | H5N1 |
| A/snow goose/Kansas/W22-199F/2022(H5N1) | 2020-2022 | North America | Wild Anseriformes | H5N1 |
| A/bald eagle/Florida/W22-195/2022(H5N1) | 2020-2022 | North America | Other wild species | H5N1 |
| A/bald eagle/Kansas/W22-197/2022(H5N1) | 2020-2022 | North America | Other wild species | H5N1 |
| A/chicken/Wisconsin/22-007545-001-original/2022(H5N1) | 2020-2022 | North America | Domestic Galliformes | H5N1 |
| A/chicken/Wisconsin/22-007545-002-original/2022(H5N1) | 2020-2022 | North America | Domestic Galliformes | H5N1 |
| A/Anser anser/Spain/1035-1/2022(H5N1) | 2020-2022 | West Europe | Wild Anseriformes | H5N1 |
| A/Anser anser/Spain/1035-5 22VIR6312-8/2022(H5N1) | 2020-2022 | West Europe | Wild Anseriformes | H5N1 |
| A/bald eagle/Georgia/W22-194B/2022(H5N1) | 2020-2022 | West Central Asia | Other wild species | H5N1 |
| A/chicken/Missouri/22-007677-001-original/2022(H5N1) | 2020-2022 | North America | Domestic Galliformes | H5N1 |
| A/chicken/Missouri/22-007677-002-original/2022(H5N1) | 2020-2022 | North America | Domestic Galliformes | H5N1 |
| A/chicken/Nebraska/22-007805-001-original/2022(H5N1) | 2020-2022 | North America | Domestic Galliformes | H5N1 |
| A/crow/Hokkaido/0103L018/2022 (H5N1) | 2020-2022 | Japan | Other wild species | H5N1 |
| A/dalmatian pelican/Astrakhan/213-1V/2022(H5N1) | 2020-2022 | East Europe | Other wild species | H5N1 |
| A/dalmatian pelican/Astrakhan/213-2V/2022(H5N1) | 2020-2022 | East Europe | Other wild species | H5N1 |
| A/red-tailed hawk/Kansas/W22-198/2022(H5N1) | 2020-2022 | North America | Other wild species | H5N1 |
| A/snow goose/Kansas/W22-199A/2022(H5N1) | 2020-2022 | North America | Wild Anseriformes | H5N1 |
| A/waterfowl/Nebraska/22-007805-002-original/2022(H5N1) | 2020-2022 | North America | Other wild species | H5N1 |
| A/chicken/Italy/IZSLT22VIR2562-1/2022(H5N1) | 2020-2022 | West Europe | Domestic Galliformes | H5N1 |
| A/chicken/Maine/22-008064-002-original/2022(H5N1) | 2020-2022 | North America | Domestic Galliformes | H5N1 |
| A/Great black-backed Gull/Netherlands/3/2022(H5N1) | 2020-2022 | West Europe | Charadriiformes | H5N1 |
| A/guinea fowl/Maine/22-008064-001-original/2022(H5N1) | 2020-2022 | North America | Other wild species | H5N1 |
| A/pelican/Greece/72 CL 22VIR3126-11/2022(H5N1) | 2020-2022 | East Europe | Other wild species | H5N1 |
| A/turkey/New Hampshire/22-007886-001-original/2022(H5N1) | 2020-2022 | North America | Domestic Galliformes | H5N1 |
| A/chicken/Delaware/22-008054-002-original/2022(H5N1) | 2020-2022 | North America | Domestic Galliformes | H5N1 |
| A/chicken/Delaware/22-008054-003-original/2022(H5N1) | 2020-2022 | North America | Domestic Galliformes | H5N1 |
| A/chicken/Kansas/22-008114-002-original/2022(H5N1) | 2020-2022 | North America | Domestic Galliformes | H5N1 |
| A/chicken/Kansas/22-008259-001-original/2022(H5N1) | 2020-2022 | North America | Domestic Galliformes | H5N1 |
| A/chicken/Kansas/22-008259-002-original/2022(H5N1) | 2020-2022 | North America | Domestic Galliformes | H5N1 |
| A/chicken/Mali/S3-179 22VIR6104-5/2022(H5N1) | 2020-2022 | Africa | Domestic Galliformes | H5N1 |
| A/chicken/Mali/T1-177 22VIR6104-1/2022(H5N1) | 2020-2022 | Africa | Domestic Galliformes | H5N1 |
| A/chicken/Mali/T2-178 22VIR6104-3/2022(H5N1) | 2020-2022 | Africa | Domestic Galliformes | H5N1 |
| A/chicken/Mali/T4 180 22VIR6104-7/2022(H5N1) | 2020-2022 | Africa | Domestic Galliformes | H5N1 |
| A/chicken/Spain/1096-8 22VIR6312-35/2022(H5N1) | 2020-2022 | West Europe | Domestic Galliformes | H5N1 |
| A/duck/Kansas/22-008114-001-original/2022(H5N1) | 2020-2022 | North America | Domestic Anseriformes | H5N1 |
| A/Turkey/Spain/1097-6/2022(H5N1) | 2020-2022 | West Europe | Domestic Galliformes | H5N1 |
| A/turkey/Spain/1097-7 22VIR6312-36/2022(H5N1) | 2020-2022 | West Europe | Domestic Galliformes | H5N1 |
| A/buzzard/Scotland/043118/2022(H5N1) | 2020-2022 | West Europe | Other wild species | H5N1 |
| A/chicken/Iowa/22-008176-003-original/2022(H5N1) | 2020-2022 | North America | Domestic Galliformes | H5N1 |
| A/chicken/Maryland/22-008243-001-original/2022(H5N1) | 2020-2022 | North America | Domestic Galliformes | H5N1 |
| A/pelican/Romania/11334 22VIR4106-3/2022(H5N1) | 2020-2022 | East Europe | Other wild species | H5N1 |
| A/pelican/Romania/11335 22VIR4106-2/2022(H5N1) | 2020-2022 | East Europe | Other wild species | H5N1 |
| A/turkey/South Dakota/22-008239-001-original/2022(H5N1) | 2020-2022 | North America | Domestic Galliformes | H5N1 |
| A/turkey/South Dakota/22-008239-002-original/2022(H5N1) | 2020-2022 | North America | Domestic Galliformes | H5N1 |
| A/turkey/South Dakota/22-008242-001-original/2022(H5N1) | 2020-2022 | North America | Domestic Galliformes | H5N1 |
| A/turkey/South Dakota/22-008242-002-original/2022(H5N1) | 2020-2022 | North America | Domestic Galliformes | H5N1 |
| A/Anser anser/Spain/1124-21/2022(H5N1) | 2020-2022 | West Europe | Wild Anseriformes | H5N1 |
| A/Anser anser/Spain/1124-65 22VIR6312-9/2022(H5N1) | 2020-2022 | West Europe | Wild Anseriformes | H5N1 |
| A/chicken/Maine/22-008366-001-original/2022(H5N1) | 2020-2022 | North America | Domestic Galliformes | H5N1 |
| A/chicken/Maine/22-008367-001-original/2022(H5N1) | 2020-2022 | North America | Domestic Galliformes | H5N1 |
| A/laying hen/Romania/11343 22VIR4106-1/2022(H5N1) | 2020-2022 | East Europe | Domestic Galliformes | H5N1 |
| A/turkey/Maine/22-008367-002-original/2022(H5N1) | 2020-2022 | North America | Domestic Galliformes | H5N1 |
| A/chicken/Iowa/22-008373-001-original/2022(H5N1) | 2020-2022 | North America | Domestic Galliformes | H5N1 |
| A/chicken/Iowa/22-008373-002-original/2022(H5N1) | 2020-2022 | North America | Domestic Galliformes | H5N1 |
| A/Falco peregrinus/Belgium/4055 0002/2022(H5N1) | 2020-2022 | West Europe | Other wild species | H5N1 |
| A/chicken/Nebraska/22-008467-001-original/2022(H5N1) | 2020-2022 | North America | Domestic Galliformes | H5N1 |
| A/turkey/South Dakota/22-008479-001-original/2022(H5N1) | 2020-2022 | North America | Domestic Galliformes | H5N1 |
| A/turkey/South Dakota/22-008479-002-original/2022(H5N1) | 2020-2022 | North America | Domestic Galliformes | H5N1 |
| A/turkey/South Dakota/22-008483-001-original/2022(H5N1) | 2020-2022 | North America | Domestic Galliformes | H5N1 |
| A/turkey/South Dakota/22-008485-001-original/2022(H5N1) | 2020-2022 | North America | Domestic Galliformes | H5N1 |
| A/turkey/South Dakota/22-008485-002-original/2022(H5N1) | 2020-2022 | North America | Domestic Galliformes | H5N1 |
| A/chicken/Maine/22-008540-001-original/2022(H5N1) | 2020-2022 | North America | Domestic Galliformes | H5N1 |
| A/chicken/Michigan/22-008890-006-original/2022(H5N1) | 2020-2022 | North America | Domestic Galliformes | H5N1 |
| A/chicken/South Dakota/22-008704-001-original/2022(H5N1) | 2020-2022 | North America | Domestic Galliformes | H5N1 |
| A/chicken/South Dakota/22-008704-002-original/2022(H5N1) | 2020-2022 | North America | Domestic Galliformes | H5N1 |
| A/domestic duck/Michigan/22-008890-001-original/2022(H5N1) | 2020-2022 | North America | Domestic Anseriformes | H5N1 |
| A/domestic goose/Michigan/22-008890-004-original/2022(H5N1) | 2020-2022 | North America | Domestic Anseriformes | H5N1 |
| A/mallard/New York/22-008760-007-original/2022(H5N1) | 2020-2022 | North America | Wild Anseriformes | H5N1 |
| A/pheasant/New York/22-008760-008-original/2022(H5N1) | 2020-2022 | North America | Other wild species | H5N1 |
| A/turkey/Michigan/22-008890-007-original/2022(H5N1) | 2020-2022 | North America | Domestic Galliformes | H5N1 |
| A/turkey/South Dakota/22-008702-001-original/2022(H5N1) | 2020-2022 | North America | Domestic Galliformes | H5N1 |
| A/turkey/South Dakota/22-008702-002-original/2022(H5N1) | 2020-2022 | North America | Domestic Galliformes | H5N1 |
| A/turkey/South Dakota/22-008705-001-original/2022(H5N1) | 2020-2022 | North America | Domestic Galliformes | H5N1 |
| A/turkey/South Dakota/22-008705-002-original/2022(H5N1) | 2020-2022 | North America | Domestic Galliformes | H5N1 |
| A/chicken/Nebraska/22-009006-001-original/2022(H5N1) | 2020-2022 | North America | Domestic Galliformes | H5N1 |
| A/greylag goose/Netherlands/22005844-002/2022(H5N1) | 2020-2022 | West Europe | Wild Anseriformes | H5N1 |
| A/turkey/Iowa/22-008862-002-original/2022(H5N1) | 2020-2022 | North America | Domestic Galliformes | H5N1 |
| A/turkey/South Dakota/22-008866-001-original/2022(H5N1) | 2020-2022 | North America | Domestic Galliformes | H5N1 |
| A/turkey/South Dakota/22-008866-002-original/2022(H5N1) | 2020-2022 | North America | Domestic Galliformes | H5N1 |
| A/chicken/Nebraska/22-009190-002-original/2022(H5N1) | 2020-2022 | North America | Domestic Galliformes | H5N1 |
| A/domestic duck/Nebraska/22-009190-001-original/2022(H5N1) | 2020-2022 | North America | Domestic Anseriformes | H5N1 |
| A/sea eagle/Norway/2022-07-198 22VIR3866-2/2022(H5N5) | 2020-2022 | West Europe | Other wild species | H5N5 |
| A/turkey/South Dakota/22-009023-001-original/2022(H5N1) | 2020-2022 | North America | Domestic Galliformes | H5N1 |
| A/turkey/South Dakota/22-009023-002-original/2022(H5N1) | 2020-2022 | North America | Domestic Galliformes | H5N1 |
| A/chicken/Iowa/22-009180-001-original/2022(H5N1) | 2020-2022 | North America | Domestic Galliformes | H5N1 |
| A/chicken/Iowa/22-009180-002-original/2022(H5N1) | 2020-2022 | North America | Domestic Galliformes | H5N1 |
| A/chicken/Maine/22-009412-001-original/2022(H5N1) | 2020-2022 | North America | Domestic Galliformes | H5N1 |
| A/chicken/Minnesota/22-009181-001-original/2022(H5N1) | 2020-2022 | North America | Domestic Galliformes | H5N1 |
| A/chicken/Missouri/22-009192-002-original/2022(H5N1) | 2020-2022 | North America | Domestic Galliformes | H5N1 |
| A/chicken/South Dakota/22-009194-002-original/2022(H5N1) | 2020-2022 | North America | Domestic Galliformes | H5N1 |
| A/duck/Missouri/22-009192-003-original/2022(H5N1) | 2020-2022 | North America | Domestic Anseriformes | H5N1 |
| A/Embden goose/Minnesota/22-009181-003-original/2022(H5N1) | 2020-2022 | North America | Wild Anseriformes | H5N1 |
| A/guinea fowl/Maine/22-009412-002-original/2022(H5N1) | 2020-2022 | North America | Other wild species | H5N1 |
| A/guinea fowl/South Dakota/22-009194-003-original/2022(H5N1) | 2020-2022 | North America | Other wild species | H5N1 |
| A/pheasant/New York/22-009066-001-original/2022(H5N1) | 2020-2022 | North America | Other wild species | H5N1 |
| A/sea eagle/Norway/2022-07-196 22VIR3866-1/2022(H5N5) | 2020-2022 | West Europe | Other wild species | H5N5 |
| A/Sebastopol goose/Massachusetts/22-009371-001-original/2022(H5N1) | 2020-2022 | North America | Wild Anseriformes | H5N1 |
| A/snow goose/Kansas/W22-260/2022(H5N1) | 2020-2022 | North America | Wild Anseriformes | H5N1 |
| A/turkey/Minnesota/22-009182-001-original/2022(H5N1) | 2020-2022 | North America | Domestic Galliformes | H5N1 |
| A/turkey/Minnesota/22-009182-003-original/2022(H5N1) | 2020-2022 | North America | Domestic Galliformes | H5N1 |
| A/Anser anser/Spain/1277-10 22VIR6312-10/2022(H5N1) | 2020-2022 | West Europe | Wild Anseriformes | H5N1 |
| A/chicken/Ohio/22-009419-001-original/2022(H5N1) | 2020-2022 | North America | Domestic Galliformes | H5N1 |
| A/chicken/Wyoming/22-009599-001-original/2022(H5N1) | 2020-2022 | North America | Domestic Galliformes | H5N1 |
| A/chicken/Wyoming/22-009599-002-original/2022(H5N1) | 2020-2022 | North America | Domestic Galliformes | H5N1 |
| A/domestic duck/England/032919/2022(H5N1) | 2020-2022 | West Europe | Domestic Anseriformes | H5N1 |
| A/laying hen/Romania/11562 22VIR4106-4/2022(H5N1) | 2020-2022 | East Europe | Domestic Galliformes | H5N1 |
| A/turkey/Minnesota/22-009195-002-original/2022(H5N1) | 2020-2022 | North America | Domestic Galliformes | H5N1 |
| A/turkey/Minnesota/22-009195-003-original/2022(H5N1) | 2020-2022 | North America | Domestic Galliformes | H5N1 |
| A/turkey/South Dakota/22-009196-001-original/2022(H5N1) | 2020-2022 | North America | Domestic Galliformes | H5N1 |
| A/turkey/South Dakota/22-009196-002-original/2022(H5N1) | 2020-2022 | North America | Domestic Galliformes | H5N1 |
| A/turkey/South Dakota/22-009198-001-original/2022(H5N1) | 2020-2022 | North America | Domestic Galliformes | H5N1 |
| A/black-backed gull/Netherlands/22006192-001/2022(H5N1) | 2020-2022 | West Europe | Charadriiformes | H5N1 |
| A/turkey/Iowa/22-009227-001-original/2022(H5N1) | 2020-2022 | North America | Domestic Galliformes | H5N1 |
| A/turkey/North Carolina/22-009332-001-original/2022(H5N1) | 2020-2022 | North America | Domestic Galliformes | H5N1 |
| A/turkey/North Carolina/22-009332-002-original/2022(H5N1) | 2020-2022 | North America | Domestic Galliformes | H5N1 |
| A/turkey/North Carolina/22-009583-001-original/2022(H5N1) | 2020-2022 | North America | Domestic Galliformes | H5N1 |
| A/turkey/North Carolina/22-009583-002-original/2022(H5N1) | 2020-2022 | North America | Domestic Galliformes | H5N1 |
| A/turkey/South Dakota/22-009327-001-original/2022(H5N1) | 2020-2022 | North America | Domestic Galliformes | H5N1 |
| A/turkey/South Dakota/22-009327-002-original/2022(H5N1) | 2020-2022 | North America | Domestic Galliformes | H5N1 |
| A/turkey/South Dakota/22-009329-001-original/2022(H5N1) | 2020-2022 | North America | Domestic Galliformes | H5N1 |
| A/turkey/South Dakota/22-009329-002-original/2022(H5N1) | 2020-2022 | North America | Domestic Galliformes | H5N1 |
| A/American Buff goose/New York/22-009324-006-original/2022(H5N1) | 2020-2022 | North America | Wild Anseriformes | H5N1 |
| A/chicken/Iowa/22-009287-001-original/2022(H5N1) | 2020-2022 | North America | Domestic Galliformes | H5N1 |
| A/chicken/Iowa/22-009287-002-original/2022(H5N1) | 2020-2022 | North America | Domestic Galliformes | H5N1 |
| A/chicken/Maine/22-009605-002-original/2022(H5N1) | 2020-2022 | North America | Domestic Galliformes | H5N1 |
| A/chicken/New York/22-009324-002-original/2022(H5N1) | 2020-2022 | North America | Domestic Galliformes | H5N1 |
| A/chicken/North Dakota/22-009337-002-original/2022(H5N1) | 2020-2022 | North America | Domestic Galliformes | H5N1 |
| A/chicken/Wyoming/22-009849-001-original/2022(H5N1) | 2020-2022 | North America | Domestic Galliformes | H5N1 |
| A/domestic duck/New York/22-009324-004-original/2022(H5N1) | 2020-2022 | North America | Domestic Anseriformes | H5N1 |
| A/domestic goose/North Dakota/22-009337-001-original/2022(H5N1) | 2020-2022 | North America | Domestic Anseriformes | H5N1 |
| A/Gallus gallus/Belgium/4190 0002/2022(H5N1) | 2020-2022 | West Europe | Domestic Galliformes | H5N1 |
| A/Goose/Scotland/036879/2022(H5N1) | 2020-2022 | West Europe | Domestic Anseriformes | H5N1 |
| A/greylag goose/Netherlands/22006190-002/2022(H5N1) | 2020-2022 | West Europe | Wild Anseriformes | H5N1 |
| A/quail/New York/22-009324-009-original/2022(H5N1) | 2020-2022 | North America | Domestic Galliformes | H5N1 |
| A/turkey/Iowa/22-009286-001-original/2022(H5N1) | 2020-2022 | North America | Domestic Galliformes | H5N1 |
| A/turkey/Iowa/22-009286-002-original/2022(H5N1) | 2020-2022 | North America | Domestic Galliformes | H5N1 |
| A/turkey/Maine/22-009605-001-original/2022(H5N1) | 2020-2022 | North America | Domestic Galliformes | H5N1 |
| A/turkey/Minnesota/22-009313-001-original/2022(H5N1) | 2020-2022 | North America | Domestic Galliformes | H5N1 |
| A/turkey/Minnesota/22-009313-002-original/2022(H5N1) | 2020-2022 | North America | Domestic Galliformes | H5N1 |
| A/turkey/Minnesota/22-009314-001-original/2022(H5N1) | 2020-2022 | North America | Domestic Galliformes | H5N1 |
| A/turkey/Minnesota/22-009314-002-original/2022(H5N1) | 2020-2022 | North America | Domestic Galliformes | H5N1 |
| A/turkey/South Dakota/22-009330-001-original/2022(H5N1) | 2020-2022 | North America | Domestic Galliformes | H5N1 |
| A/turkey/South Dakota/22-009330-002-original/2022(H5N1) | 2020-2022 | North America | Domestic Galliformes | H5N1 |
| A/chicken/Illinois/22-009720-001-original/2022(H5N1) | 2020-2022 | North America | Domestic Galliformes | H5N1 |
| A/chicken/Minnesota/22-009735-001-original/2022(H5N1) | 2020-2022 | North America | Domestic Galliformes | H5N1 |
| A/chicken/Wyoming/22-009326-002-original/2022(H5N1) | 2020-2022 | North America | Domestic Galliformes | H5N1 |
| A/crow/Hokkaido/0103B065/2022(H5N1) | 2020-2022 | Japan | Other wild species | H5N1 |
| A/goose/Illinois/22-009720-003-original/2022(H5N1) | 2020-2022 | North America | Domestic Anseriformes | H5N1 |
| A/pheasant/Texas/22-009984-001-original/2022(H5N1) | 2020-2022 | North America | Other wild species | H5N1 |
| A/pheasant/Texas/22-010008-001-original/2022(H5N1) | 2020-2022 | North America | Other wild species | H5N1 |
| A/pheasant/Texas/22-010008-002-original/2022(H5N1) | 2020-2022 | North America | Other wild species | H5N1 |
| A/turkey/Iowa/22-009550-001-original/2022(H5N1) | 2020-2022 | North America | Domestic Galliformes | H5N1 |
| A/turkey/South Dakota/22-009534-001-original/2022(H5N1) | 2020-2022 | North America | Domestic Galliformes | H5N1 |
| A/bottlenose dolphin/Florida/UFTt2203/2022(H5N1) | 2020-2022 | North America | Mammal | H5N1 |
| A/chicken/North Dakota/22-009677-001-original/2022(H5N1) | 2020-2022 | North America | Domestic Galliformes | H5N1 |
| A/dolphin/Florida/22-025319-002-original/2022(H5N1) | 2020-2022 | North America | Mammal | H5N1 |
| A/turkey/Minnesota/22-009820-001-original/2022(H5N1) | 2020-2022 | North America | Domestic Galliformes | H5N1 |
| A/turkey/South Dakota/22-009688-001-original/2022(H5N1) | 2020-2022 | North America | Domestic Galliformes | H5N1 |
| A/turkey/South Dakota/22-009839-001-original/2022(H5N1) | 2020-2022 | North America | Domestic Galliformes | H5N1 |
| A/wild bird/Spain/1302-1 22VIR6312-11/2022(H5N1) | 2020-2022 | West Europe | Other wild species | H5N1 |
| A/black vulture/Florida/22-010358-001-original/2022(H5N1) | 2020-2022 | North America | Other wild species | H5N1 |
| A/chicken/Iowa/22-009819-001-original/2022(H5N1) | 2020-2022 | North America | Domestic Galliformes | H5N1 |
| A/chicken/Nebraska/22-010155-001-original/2022(H5N1) | 2020-2022 | North America | Domestic Galliformes | H5N1 |
| A/chicken/Wyoming/22-010157-001-original/2022(H5N1) | 2020-2022 | North America | Domestic Galliformes | H5N1 |
| A/chicken/Wyoming/22-010157-002-original/2022(H5N1) | 2020-2022 | North America | Domestic Galliformes | H5N1 |
| A/chicken/Wyoming/22-010378-001-original/2022(H5N1) | 2020-2022 | North America | Domestic Galliformes | H5N1 |
| A/Ezo red fox/Hokkaido/1/2022(H5N1) | 2020-2022 | Japan | Mammal | H5N1 |
| A/silkie chicken/Wisconsin/22-009934-002-original/2022(H5N1) | 2020-2022 | North America | Other wild species | H5N1 |
| A/turkey/Iowa/22-009825-005-original/2022(H5N1) | 2020-2022 | North America | Domestic Galliformes | H5N1 |
| A/turkey/Minnesota/22-009976-003-original/2022(H5N1) | 2020-2022 | North America | Domestic Galliformes | H5N1 |
| A/turkey/Missouri/22-009845-004-original/2022(H5N1) | 2020-2022 | North America | Domestic Galliformes | H5N1 |
| A/turkey/North Carolina/22-009981-001-original/2022(H5N1) | 2020-2022 | North America | Domestic Galliformes | H5N1 |
| A/turkey/North Carolina/22-009981-004-original/2022(H5N1) | 2020-2022 | North America | Domestic Galliformes | H5N1 |
| A/turkey/North Carolina/22-009982-001-original/2022(H5N1) | 2020-2022 | North America | Domestic Galliformes | H5N1 |
| A/turkey/North Carolina/22-009982-002-original/2022(H5N1) | 2020-2022 | North America | Domestic Galliformes | H5N1 |
| A/turkey/North Carolina/22-009983-007-original/2022(H5N1) | 2020-2022 | North America | Domestic Galliformes | H5N1 |
| A/turkey/North Carolina/22-009983-008-original/2022(H5N1) | 2020-2022 | North America | Domestic Galliformes | H5N1 |
| A/turkey/North Carolina/22-010144-001-original/2022(H5N1) | 2020-2022 | North America | Domestic Galliformes | H5N1 |
| A/turkey/North Carolina/22-010144-002-original/2022(H5N1) | 2020-2022 | North America | Domestic Galliformes | H5N1 |
| A/turkey/North Carolina/22-010145-001-original/2022(H5N1) | 2020-2022 | North America | Domestic Galliformes | H5N1 |
| A/turkey/North Carolina/22-010145-002-original/2022(H5N1) | 2020-2022 | North America | Domestic Galliformes | H5N1 |
| A/turkey/North Dakota/22-009840-001-original/2022(H5N1) | 2020-2022 | North America | Domestic Galliformes | H5N1 |
| A/turkey/South Dakota/22-009841-001-original/2022(H5N1) | 2020-2022 | North America | Domestic Galliformes | H5N1 |
| A/chicken/Iowa/22-009985-001-original/2022(H5N1) | 2020-2022 | North America | Domestic Galliformes | H5N1 |
| A/crow/Hokkaido/0103B073/2022 (H5N1) | 2020-2022 | Japan | Other wild species | H5N1 |
| A/tanuki/Hokkaido/1/2022(H5N1) | 2020-2022 | Japan | Mammal | H5N1 |
| A/turkey/Minnesota/22-009996-001-original/2022(H5N1) | 2020-2022 | North America | Domestic Galliformes | H5N1 |
| A/turkey/Minnesota/22-009996-002-original/2022(H5N1) | 2020-2022 | North America | Domestic Galliformes | H5N1 |
| A/turkey/North Dakota/22-009978-001-original/2022(H5N1) | 2020-2022 | North America | Domestic Galliformes | H5N1 |
| A/turkey/North Dakota/22-009978-002-original/2022(H5N1) | 2020-2022 | North America | Domestic Galliformes | H5N1 |
| A/turkey/South Dakota/22-009979-001-original/2022(H5N1) | 2020-2022 | North America | Domestic Galliformes | H5N1 |
| A/turkey/South Dakota/22-009979-002-original/2022(H5N1) | 2020-2022 | North America | Domestic Galliformes | H5N1 |
| A/turkey/South Dakota/22-009980-001-original/2022(H5N1) | 2020-2022 | North America | Domestic Galliformes | H5N1 |
| A/turkey/South Dakota/22-009980-002-original/2022(H5N1) | 2020-2022 | North America | Domestic Galliformes | H5N1 |
| A/Fox/Netherlands/EMC4/2022(H5N1) | 2020-2022 | West Europe | Mammal | H5N1 |
| A/turkey/Iowa/22-009986-001-original/2022(H5N1) | 2020-2022 | North America | Domestic Galliformes | H5N1 |
| A/turkey/Iowa/22-009986-002-original/2022(H5N1) | 2020-2022 | North America | Domestic Galliformes | H5N1 |
| A/turkey/Iowa/22-010094-001-original/2022(H5N1) | 2020-2022 | North America | Domestic Galliformes | H5N1 |
| A/turkey/Iowa/22-010094-002-original/2022(H5N1) | 2020-2022 | North America | Domestic Galliformes | H5N1 |
| A/turkey/Minnesota/22-009997-001-original/2022(H5N1) | 2020-2022 | North America | Domestic Galliformes | H5N1 |
| A/turkey/Minnesota/22-009997-002-original/2022(H5N1) | 2020-2022 | North America | Domestic Galliformes | H5N1 |
| A/turkey/Minnesota/22-009998-001-original/2022(H5N1) | 2020-2022 | North America | Domestic Galliformes | H5N1 |
| A/turkey/Minnesota/22-009999-001-original/2022(H5N1) | 2020-2022 | North America | Domestic Galliformes | H5N1 |
| A/turkey/Minnesota/22-009999-002-original/2022(H5N1) | 2020-2022 | North America | Domestic Galliformes | H5N1 |
| A/turkey/North Dakota/22-010015-001-original/2022(H5N1) | 2020-2022 | North America | Domestic Galliformes | H5N1 |
| A/turkey/North Dakota/22-010015-002-original/2022(H5N1) | 2020-2022 | North America | Domestic Galliformes | H5N1 |
| A/turkey/South Dakota/22-010013-001-original/2022(H5N1) | 2020-2022 | North America | Domestic Galliformes | H5N1 |
| A/turkey/South Dakota/22-010013-002-original/2022(H5N1) | 2020-2022 | North America | Domestic Galliformes | H5N1 |
| A/turkey/Minnesota/22-010000-001-original/2022(H5N1) | 2020-2022 | North America | Domestic Galliformes | H5N1 |
| A/turkey/Minnesota/22-010085-001-original/2022(H5N1) | 2020-2022 | North America | Domestic Galliformes | H5N1 |
| A/turkey/Minnesota/22-010085-002-original/2022(H5N1) | 2020-2022 | North America | Domestic Galliformes | H5N1 |
| A/turkey/Minnesota/22-010092-002-original/2022(H5N1) | 2020-2022 | North America | Domestic Galliformes | H5N1 |
| A/turkey/Minnesota/22-010092-003-original/2022(H5N1) | 2020-2022 | North America | Domestic Galliformes | H5N1 |
| A/Amazon parrot/Michigan/22-010848-001-original/2022(H5N1) | 2020-2022 | North America | Other wild species | H5N1 |
| A/Catalina macaw/Michigan/22-010848-002-original/2022(H5N1) | 2020-2022 | North America | Other wild species | H5N1 |
| A/chicken/New York/22-010200-002-original/2022(H5N1) | 2020-2022 | North America | Domestic Galliformes | H5N1 |
| A/chicken/New York/22-010321-001-original/2022(H5N1) | 2020-2022 | North America | Domestic Galliformes | H5N1 |
| A/chicken/New York/22-010321-002-original/2022(H5N1) | 2020-2022 | North America | Domestic Galliformes | H5N1 |
| A/chicken/North Carolina/22-010298-001-original/2022(H5N1) | 2020-2022 | North America | Domestic Galliformes | H5N1 |
| A/chicken/North Carolina/22-010298-002-original/2022(H5N1) | 2020-2022 | North America | Domestic Galliformes | H5N1 |
| A/chicken/North Carolina/22-010299-002-original/2022(H5N1) | 2020-2022 | North America | Domestic Galliformes | H5N1 |
| A/chicken/North Dakota/22-010141-001-original/2022(H5N1) | 2020-2022 | North America | Domestic Galliformes | H5N1 |
| A/domestic duck/England/041295/2022(H5N1) | 2020-2022 | West Europe | Domestic Anseriformes | H5N1 |
| A/domestic duck/New York/22-010321-003-original/2022(H5N1) | 2020-2022 | North America | Domestic Anseriformes | H5N1 |
| A/fox/New York/074441/2022(H5N1) | 2020-2022 | North America | Mammal | H5N1 |
| A/fox/New York/099488/2022(H5N1) | 2020-2022 | North America | Mammal | H5N1 |
| A/goose/Maine/22-010181-002-original/2022(H5N1) | 2020-2022 | North America | Domestic Anseriformes | H5N1 |
| A/guinea fowl/Maine/22-010181-001-original/2022(H5N1) | 2020-2022 | North America | Other wild species | H5N1 |
| A/guinea fowl/New York/22-010200-003-original/2022(H5N1) | 2020-2022 | North America | Other wild species | H5N1 |
| A/turkey/Iowa/22-010132-001-original/2022(H5N1) | 2020-2022 | North America | Domestic Galliformes | H5N1 |
| A/turkey/Iowa/22-010132-002-original/2022(H5N1) | 2020-2022 | North America | Domestic Galliformes | H5N1 |
| A/turkey/Minnesota/22-010122-001-original/2022(H5N1) | 2020-2022 | North America | Domestic Galliformes | H5N1 |
| A/turkey/Minnesota/22-010122-002-original/2022(H5N1) | 2020-2022 | North America | Domestic Galliformes | H5N1 |
| A/turkey/Minnesota/22-010123-001-original/2022(H5N1) | 2020-2022 | North America | Domestic Galliformes | H5N1 |
| A/turkey/Minnesota/22-010123-004-original/2022(H5N1) | 2020-2022 | North America | Domestic Galliformes | H5N1 |
| A/turkey/Minnesota/22-010124-001-original/2022(H5N1) | 2020-2022 | North America | Domestic Galliformes | H5N1 |
| A/turkey/Minnesota/22-010124-002-original/2022(H5N1) | 2020-2022 | North America | Domestic Galliformes | H5N1 |
| A/turkey/Minnesota/22-010125-001-original/2022(H5N1) | 2020-2022 | North America | Domestic Galliformes | H5N1 |
| A/turkey/Minnesota/22-010125-002-original/2022(H5N1) | 2020-2022 | North America | Domestic Galliformes | H5N1 |
| A/turkey/Minnesota/22-010126-001-original/2022(H5N1) | 2020-2022 | North America | Domestic Galliformes | H5N1 |
| A/turkey/Minnesota/22-010126-002-original/2022(H5N1) | 2020-2022 | North America | Domestic Galliformes | H5N1 |
| A/turkey/Minnesota/22-010127-001-original/2022(H5N1) | 2020-2022 | North America | Domestic Galliformes | H5N1 |
| A/turkey/Minnesota/22-010127-002-original/2022(H5N1) | 2020-2022 | North America | Domestic Galliformes | H5N1 |
| A/turkey/Minnesota/22-010312-001-original/2022(H5N1) | 2020-2022 | North America | Domestic Galliformes | H5N1 |
| A/turkey/Minnesota/22-010312-004-original/2022(H5N1) | 2020-2022 | North America | Domestic Galliformes | H5N1 |
| A/turkey/Minnesota/22-010654-001-original/2022(H5N1) | 2020-2022 | North America | Domestic Galliformes | H5N1 |
| A/turkey/Missouri/22-010142-001-original/2022(H5N1) | 2020-2022 | North America | Domestic Galliformes | H5N1 |
| A/turkey/Missouri/22-010296-001-original/2022(H5N1) | 2020-2022 | North America | Domestic Galliformes | H5N1 |
| A/turkey/North Carolina/22-010146-001-original/2022(H5N1) | 2020-2022 | North America | Domestic Galliformes | H5N1 |
| A/turkey/North Carolina/22-010146-002-original/2022(H5N1) | 2020-2022 | North America | Domestic Galliformes | H5N1 |
| A/turkey/South Dakota/22-010135-001-original/2022(H5N1) | 2020-2022 | North America | Domestic Galliformes | H5N1 |
| A/turkey/South Dakota/22-010135-002-original/2022(H5N1) | 2020-2022 | North America | Domestic Galliformes | H5N1 |
| A/turkey/South Dakota/22-010137-001-original/2022(H5N1) | 2020-2022 | North America | Domestic Galliformes | H5N1 |
| A/turkey/South Dakota/22-010137-002-original/2022(H5N1) | 2020-2022 | North America | Domestic Galliformes | H5N1 |
| A/turkey/South Dakota/22-010138-001-original/2022(H5N1) | 2020-2022 | North America | Domestic Galliformes | H5N1 |
| A/turkey/South Dakota/22-010138-002-original/2022(H5N1) | 2020-2022 | North America | Domestic Galliformes | H5N1 |
| A/turkey/South Dakota/22-010139-001-original/2022(H5N1) | 2020-2022 | North America | Domestic Galliformes | H5N1 |
| A/turkey/South Dakota/22-010139-002-original/2022(H5N1) | 2020-2022 | North America | Domestic Galliformes | H5N1 |
| A/turkey/South Dakota/22-010346-001-original/2022(H5N1) | 2020-2022 | North America | Domestic Galliformes | H5N1 |
| A/turkey/South Dakota/22-010346-002-original/2022(H5N1) | 2020-2022 | North America | Domestic Galliformes | H5N1 |
| A/black-backed gull/Netherlands/22006711-001/2022(H5N1) | 2020-2022 | West Europe | Charadriiformes | H5N1 |
| A/chicken/Montana/22-010445-001-original/2022(H5N1) | 2020-2022 | North America | Domestic Galliformes | H5N1 |
| A/chicken/Montana/22-010445-003-original/2022(H5N1) | 2020-2022 | North America | Domestic Galliformes | H5N1 |
| A/chicken/Wisconsin/22-010325-001-original/2022(H5N1) | 2020-2022 | North America | Domestic Galliformes | H5N1 |
| A/turkey/Minnesota/22-010463-002-original/2022(H5N1) | 2020-2022 | North America | Domestic Galliformes | H5N1 |
| A/turkey/Minnesota/22-010464-001-original/2022(H5N1) | 2020-2022 | North America | Domestic Galliformes | H5N1 |
| A/turkey/Minnesota/22-010464-002-original/2022(H5N1) | 2020-2022 | North America | Domestic Galliformes | H5N1 |
| A/turkey/South Dakota/22-010639-001-original/2022(H5N1) | 2020-2022 | North America | Domestic Galliformes | H5N1 |
| A/turkey/South Dakota/22-010639-002-original/2022(H5N1) | 2020-2022 | North America | Domestic Galliformes | H5N1 |
| A/turkey/Wisconsin/22-010325-003-original/2022(H5N1) | 2020-2022 | North America | Domestic Galliformes | H5N1 |
| A/Black-headed gull/Netherlands/2/2022(H5N1) | 2020-2022 | West Europe | Charadriiformes | H5N8 |
| A/Cascade duck/Montana/22-010454-001-original/2022(H5N1) | 2020-2022 | North America | Wild Anseriformes | H5N1 |
| A/Cascade duck/Montana/22-010454-003-original/2022(H5N1) | 2020-2022 | North America | Wild Anseriformes | H5N1 |
| A/chicken/North Dakota/22-010657-001-original/2022(H5N1) | 2020-2022 | North America | Domestic Galliformes | H5N1 |
| A/goose/North Dakota/22-010657-002-original/2022(H5N1) | 2020-2022 | North America | Domestic Anseriformes | H5N1 |
| A/turkey/Minnesota/22-010652-001-original/2022(H5N1) | 2020-2022 | North America | Domestic Galliformes | H5N1 |
| A/turkey/Minnesota/22-010652-002-original/2022(H5N1) | 2020-2022 | North America | Domestic Galliformes | H5N1 |
| A/turkey/Minnesota/22-010771-001-original/2022(H5N1) | 2020-2022 | North America | Domestic Galliformes | H5N1 |
| A/turkey/Minnesota/22-010771-003-original/2022(H5N1) | 2020-2022 | North America | Domestic Galliformes | H5N1 |
| A/turkey/North Carolina/22-010448-001-original/2022(H5N1) | 2020-2022 | North America | Domestic Galliformes | H5N1 |
| A/turkey/North Carolina/22-010448-003-original/2022(H5N1) | 2020-2022 | North America | Domestic Galliformes | H5N1 |
| A/turkey/South Dakota/22-010765-001-original/2022(H5N1) | 2020-2022 | North America | Domestic Galliformes | H5N1 |
| A/Branta canadensis/Belgium/4821 0001/2022(H5N1) | 2020-2022 | West Europe | Wild Anseriformes | H5N1 |
| A/chicken/Colorado/22-010668-001-original/2022(H5N1) | 2020-2022 | North America | Domestic Galliformes | H5N1 |
| A/duck/Michigan/22-010845-003-original/2022(H5N1) | 2020-2022 | North America | Domestic Anseriformes | H5N1 |
| A/goose/Michigan/22-010845-001-original/2022(H5N1) | 2020-2022 | North America | Domestic Anseriformes | H5N1 |
| A/goose/Michigan/22-010845-002-original/2022(H5N1) | 2020-2022 | North America | Domestic Anseriformes | H5N1 |
| A/Pekin duck/Indiana/22-010611-001-original/2022(H5N1) | 2020-2022 | North America | Wild Anseriformes | H5N1 |
| A/Pekin duck/Indiana/22-010624-001-original/2022(H5N1) | 2020-2022 | North America | Wild Anseriformes | H5N1 |
| A/Red Fox/MB/FAV-370-01/2022(H5N1) | 2020-2022 | North America | Mammal | H5N1 |
| A/turkey/Minnesota/22-010770-002-original/2022(H5N1) | 2020-2022 | North America | Domestic Galliformes | H5N1 |
| A/turkey/Minnesota/22-010770-003-original/2022(H5N1) | 2020-2022 | North America | Domestic Galliformes | H5N1 |
| A/turkey/Minnesota/22-010772-004-original/2022(H5N1) | 2020-2022 | North America | Domestic Galliformes | H5N1 |
| A/turkey/Minnesota/22-010773-001-original/2022(H5N1) | 2020-2022 | North America | Domestic Galliformes | H5N1 |
| A/lesser snow goose/North Dakota/ND-10/2022(H5N1) | 2020-2022 | North America | Wild Anseriformes | H5N1 |
| A/Rosss goose/North Dakota/N22-08/2022(H5N1) | 2020-2022 | North America | Wild Anseriformes | H5N1 |
| A/snow goose/North Dakota/N22-04/2022(H5N1) | 2020-2022 | North America | Wild Anseriformes | H5N1 |
| A/snow goose/North Dakota/N22-05/2022(H5N1) | 2020-2022 | North America | Wild Anseriformes | H5N1 |
| A/snow goose/North Dakota/N22-06/2022(H5N1) | 2020-2022 | North America | Wild Anseriformes | H5N1 |
| A/chicken/Montana/22-010923-004-original/2022(H5N1) | 2020-2022 | North America | Domestic Galliformes | H5N1 |
| A/chicken/Montana/22-010923-005-original/2022(H5N1) | 2020-2022 | North America | Domestic Galliformes | H5N1 |
| A/turkey/Kansas/22-011119-004-original/2022(H5N1) | 2020-2022 | North America | Domestic Galliformes | H5N1 |
| A/turkey/Minnesota/22-010991-001-original/2022(H5N1) | 2020-2022 | North America | Domestic Galliformes | H5N1 |
| A/turkey/Minnesota/22-010991-002-original/2022(H5N1) | 2020-2022 | North America | Domestic Galliformes | H5N1 |
| A/turkey/Minnesota/22-010992-001-original/2022(H5N1) | 2020-2022 | North America | Domestic Galliformes | H5N1 |
| A/turkey/Minnesota/22-010992-002-original/2022(H5N1) | 2020-2022 | North America | Domestic Galliformes | H5N1 |
| A/turkey/Minnesota/22-010995-001-original/2022(H5N1) | 2020-2022 | North America | Domestic Galliformes | H5N1 |
| A/turkey/Minnesota/22-010995-002-original/2022(H5N1) | 2020-2022 | North America | Domestic Galliformes | H5N1 |
| A/chicken/Minnesota/22-010993-001-original/2022(H5N1) | 2020-2022 | North America | Domestic Galliformes | H5N1 |
| A/chicken/Minnesota/22-010993-004-original/2022(H5N1) | 2020-2022 | North America | Domestic Galliformes | H5N1 |
| A/chicken/Minnesota/22-010994-001-original/2022(H5N1) | 2020-2022 | North America | Domestic Galliformes | H5N1 |
| A/chicken/Minnesota/22-010994-002-original/2022(H5N1) | 2020-2022 | North America | Domestic Galliformes | H5N1 |
| A/chicken/North Dakota/22-010977-001-original/2022(H5N1) | 2020-2022 | North America | Domestic Galliformes | H5N1 |
| A/chicken/North Dakota/22-010977-002-original/2022(H5N1) | 2020-2022 | North America | Domestic Galliformes | H5N1 |
| A/Mute swan/Wales/058560/2022(H5N1) | 2020-2022 | West Europe | Wild Anseriformes | H5N1 |
| A/turkey/Wisconsin/22-010935-002-original/2022(H5N1) | 2020-2022 | North America | Domestic Galliformes | H5N1 |
| A/chicken/Michigan/22-011121-001-original/2022(H5N1) | 2020-2022 | North America | Domestic Galliformes | H5N1 |
| A/chicken/Michigan/22-011121-003-original/2022(H5N1) | 2020-2022 | North America | Domestic Galliformes | H5N1 |
| A/chicken/Minnesota/22-010928-001-original/2022(H5N1) | 2020-2022 | North America | Domestic Galliformes | H5N1 |
| A/chicken/Nebraska/22-010905-001-original/2022(H5N1) | 2020-2022 | North America | Domestic Galliformes | H5N1 |
| A/chicken/North Carolina/22-010929-001-original/2022(H5N1) | 2020-2022 | North America | Domestic Galliformes | H5N1 |
| A/chicken/North Carolina/22-010929-002-original/2022(H5N1) | 2020-2022 | North America | Domestic Galliformes | H5N1 |
| A/chicken/North Dakota/22-011109-001-original/2022(H5N1) | 2020-2022 | North America | Domestic Galliformes | H5N1 |
| A/chicken/Wisconsin/22-011213-001-original/2022(H5N1) | 2020-2022 | North America | Domestic Galliformes | H5N1 |
| A/chicken/Wisconsin/22-011213-002-original/2022(H5N1) | 2020-2022 | North America | Domestic Galliformes | H5N1 |
| A/Common raven/Netherlands/1/2022(H5N1) | 2020-2022 | West Europe | Other wild species | H5N1 |
| A/domestic duck/Michigan/22-011121-002-original/2022(H5N1) | 2020-2022 | North America | Domestic Anseriformes | H5N1 |
| A/domestic duck/Minnesota/22-010928-004-original/2022(H5N1) | 2020-2022 | North America | Domestic Anseriformes | H5N1 |
| A/domestic goose/Michigan/22-011121-005-original/2022(H5N1) | 2020-2022 | North America | Domestic Anseriformes | H5N1 |
| A/greylag goose /Netherlands/22006859-001/2022(H5N1) | 2020-2022 | West Europe | Wild Anseriformes | H5N1 |
| A/guinea fowl/Minnesota/22-010928-003-original/2022(H5N1) | 2020-2022 | North America | Other wild species | H5N1 |
| A/turkey/Minnesota/22-010928-002-original/2022(H5N1) | 2020-2022 | North America | Domestic Galliformes | H5N1 |
| A/turkey/Minnesota/22-011140-001-original/2022(H5N1) | 2020-2022 | North America | Domestic Galliformes | H5N1 |
| A/turkey/Minnesota/22-011141-001-original/2022(H5N1) | 2020-2022 | North America | Domestic Galliformes | H5N1 |
| A/turkey/Minnesota/22-011141-002-original/2022(H5N1) | 2020-2022 | North America | Domestic Galliformes | H5N1 |
| A/turkey/Minnesota/22-011142-001-original/2022(H5N1) | 2020-2022 | North America | Domestic Galliformes | H5N1 |
| A/turkey/Minnesota/22-011142-002-original/2022(H5N1) | 2020-2022 | North America | Domestic Galliformes | H5N1 |
| A/Broiler Chicken/BC/FAV-0228/2022 (H5N1) | 2020-2022 | West Europe | Domestic Galliformes | H5N1 |
| A/Broiler chicken/BC/FAV-0228-OS/2022(H5N1) | 2020-2022 | West Europe | Domestic Galliformes | H5N1 |
| A/chicken/North Dakota/22-011286-001-original/2022(H5N1) | 2020-2022 | North America | Domestic Galliformes | H5N1 |
| A/domestic duck/Indiana/22-011343-001-original/2022(H5N1) | 2020-2022 | North America | Domestic Anseriformes | H5N1 |
| A/pheasant/South Dakota/22-011098-001-original/2022(H5N1) | 2020-2022 | North America | Other wild species | H5N1 |
| A/turkey/Minnesota/22-011337-001-original/2022(H5N1) | 2020-2022 | North America | Domestic Galliformes | H5N1 |
| A/turkey/Minnesota/22-011337-002-original/2022(H5N1) | 2020-2022 | North America | Domestic Galliformes | H5N1 |
| A/turkey/Minnesota/22-011338-001-original/2022(H5N1) | 2020-2022 | North America | Domestic Galliformes | H5N1 |
| A/turkey/Minnesota/22-011338-002-original/2022(H5N1) | 2020-2022 | North America | Domestic Galliformes | H5N1 |
| A/Branta canadensis/Belgium/5177 0003/2022(H5N1) | 2020-2022 | West Europe | Wild Anseriformes | H5N1 |
| A/Canada goose/Wyoming/22-011671-001-original/2022(H5N1) | 2020-2022 | North America | Wild Anseriformes | H5N1 |
| A/chicken/Czech Republic/8028-1/2022(H5N1) | 2020-2022 | East Europe | Domestic Galliformes | H5N1 |
| A/chicken/Czech Republic/8028-2/2022(H5N1) | 2020-2022 | East Europe | Domestic Galliformes | H5N1 |
| A/chicken/Idaho/22-011336-001-original/2022(H5N1) | 2020-2022 | North America | Domestic Galliformes | H5N1 |
| A/chicken/Michigan/22-011521-003-original/2022(H5N1) | 2020-2022 | North America | Domestic Galliformes | H5N1 |
| A/chicken/Michigan/22-011521-004-original/2022(H5N1) | 2020-2022 | North America | Domestic Galliformes | H5N1 |
| A/chicken/Michigan/22-011627-001-original/2022(H5N1) | 2020-2022 | North America | Domestic Galliformes | H5N1 |
| A/chicken/Wisconsin/22-011334-002-original/2022(H5N1) | 2020-2022 | North America | Domestic Galliformes | H5N1 |
| A/chicken/Wisconsin/22-011334-003-original/2022(H5N1) | 2020-2022 | North America | Domestic Galliformes | H5N1 |
| A/domestic duck/Michigan/22-011521-001-original/2022(H5N1) | 2020-2022 | North America | Domestic Anseriformes | H5N1 |
| A/quail/Michigan/22-011521-005-original/2022(H5N1) | 2020-2022 | North America | Domestic Galliformes | H5N1 |
| A/Song Thrush/Netherlands/1/2022(H5N1) | 2020-2022 | West Europe | Other wild species | H5N1 |
| A/turkey/Wisconsin/22-011508-001-original/2022(H5N1) | 2020-2022 | North America | Domestic Galliformes | H5N1 |
| A/turkey/Wisconsin/22-011508-002-original/2022(H5N1) | 2020-2022 | North America | Domestic Galliformes | H5N1 |
| A/Black-headed gull/Netherlands/3/2022(H5N1) | 2020-2022 | West Europe | Charadriiformes | H5N1 |
| A/Black-headed gull/Netherlands/4/2022(H5N1) | 2020-2022 | West Europe | Charadriiformes | H5N1 |
| A/Caspian Gull/Netherlands/3/2022(H5N1) | 2020-2022 | West Europe | Charadriiformes | H5N1 |
| A/chicken/Iceland/2022AI02564/2022(H5N1) | 2020-2022 | West Europe | Domestic Galliformes | H5N1 |
| A/chicken/Iceland/2022AI02565/2022(H5N1) | 2020-2022 | West Europe | Domestic Galliformes | H5N1 |
| A/chicken/Idaho/22-011347-002-original/2022(H5N1) | 2020-2022 | North America | Domestic Galliformes | H5N1 |
| A/chicken/Idaho/22-011347-004-original/2022(H5N1) | 2020-2022 | North America | Domestic Galliformes | H5N1 |
| A/chicken/Michigan/22-011526-001-original/2022(H5N1) | 2020-2022 | North America | Domestic Galliformes | H5N1 |
| A/chicken/Michigan/22-011526-003-original/2022(H5N1) | 2020-2022 | North America | Domestic Galliformes | H5N1 |
| A/chicken/Pennsylvania/22-011531-001-original/2022(H5N1) | 2020-2022 | North America | Domestic Galliformes | H5N1 |
| A/chicken/Pennsylvania/22-011531-002-original/2022(H5N1) | 2020-2022 | North America | Domestic Galliformes | H5N1 |
| A/chicken/Utah/22-011468-001-original/2022(H5N1) | 2020-2022 | North America | Domestic Galliformes | H5N1 |
| A/chicken/Utah/22-011468-002-original/2022(H5N1) | 2020-2022 | North America | Domestic Galliformes | H5N1 |
| A/Common raven/Netherlands/2/2022(H5N1) | 2020-2022 | West Europe | Other wild species | H5N1 |
| A/domestic duck/Michigan/22-011526-002-original/2022(H5N1) | 2020-2022 | North America | Domestic Anseriformes | H5N1 |
| A/European Herring Gull/Netherlands/3/2022(H5N1) | 2020-2022 | West Europe | Charadriiformes | H5N1 |
| A/Greylag goose/England/247696/2022(H5N1) | 2020-2022 | West Europe | Wild Anseriformes | H5N1 |
| A/turkey/Minnesota/22-011795-001-original/2022(H5N1) | 2020-2022 | North America | Domestic Galliformes | H5N1 |
| A/bald eagle/Virginia/W22-306/2022(H5N1) | 2020-2022 | North America | Other wild species | H5N1 |
| A/black vulture/Florida/22-012331-001-original/2022(H5N1) | 2020-2022 | North America | Other wild species | H5N1 |
| A/chicken/Colorado/22-011826-001-original/2022(H5N1) | 2020-2022 | North America | Domestic Galliformes | H5N1 |
| A/chicken/Colorado/22-011826-002-original/2022(H5N1) | 2020-2022 | North America | Domestic Galliformes | H5N1 |
| A/chicken/Colorado/22-011827-002-original/2022(H5N1) | 2020-2022 | North America | Domestic Galliformes | H5N1 |
| A/fox/New York/22-015063-001-original/2022(H5N1) | 2020-2022 | North America | Mammal | H5N1 |
| A/Indian Runner duck/Colorado/22-011827-001-original/2022(H5N1) | 2020-2022 | North America | Wild Anseriformes | H5N1 |
| A/turkey/Minnesota/22-011787-001-original/2022(H5N1) | 2020-2022 | North America | Domestic Galliformes | H5N1 |
| A/turkey/Minnesota/22-011787-002-original/2022(H5N1) | 2020-2022 | North America | Domestic Galliformes | H5N1 |
| A/turkey/Minnesota/22-011790-002-original/2022(H5N1) | 2020-2022 | North America | Domestic Galliformes | H5N1 |
| A/turkey/Minnesota/22-011791-003-original/2022(H5N1) | 2020-2022 | North America | Domestic Galliformes | H5N1 |
| A/turkey/Minnesota/22-011791-004-original/2022(H5N1) | 2020-2022 | North America | Domestic Galliformes | H5N1 |
| A/turkey/Minnesota/22-011792-001-original/2022(H5N1) | 2020-2022 | North America | Domestic Galliformes | H5N1 |
| A/turkey/Minnesota/22-011792-002-original/2022(H5N1) | 2020-2022 | North America | Domestic Galliformes | H5N1 |
| A/turkey/Minnesota/22-011793-001-original/2022(H5N1) | 2020-2022 | North America | Domestic Galliformes | H5N1 |
| A/turkey/Minnesota/22-011793-002-original/2022(H5N1) | 2020-2022 | North America | Domestic Galliformes | H5N1 |
| A/black vulture/Florida/22-012333-001-original/2022(H5N1) | 2020-2022 | North America | Other wild species | H5N1 |
| A/chicken/Hokkaido/I-1/2022(H5N1) | 2020-2022 | Japan | Domestic Galliformes | H5N1 |
| A/chicken/Pennsylvania/22-011767-001-original/2022(H5N1) | 2020-2022 | North America | Domestic Galliformes | H5N1 |
| A/emu/Hokkaido/A-2/2022(H5N1) | 2020-2022 | Japan | Other wild species | H5N1 |
| A/turkey/Minnesota/22-011794-001-original/2022(H5N1) | 2020-2022 | North America | Domestic Galliformes | H5N1 |
| A/turkey/Minnesota/22-011794-002-original/2022(H5N1) | 2020-2022 | North America | Domestic Galliformes | H5N1 |
| A/Vulpes vulpes/Belgium/8660 0016/2022(H5N1) | 2020-2022 | West Europe | Mammal | H5N1 |
| A/barnacle goose/Netherlands/22007405-004/2022(H5N1) | 2020-2022 | West Europe | Wild Anseriformes | H5N1 |
| A/duck/Indiana/22-011946-002-original/2022(H5N1) | 2020-2022 | North America | Domestic Anseriformes | H5N1 |
| A/duck/Indiana/22-011946-004-original/2022(H5N1) | 2020-2022 | North America | Domestic Anseriformes | H5N1 |
| A/turkey/Minnesota/22-011789-001-original/2022(H5N1) | 2020-2022 | North America | Domestic Galliformes | H5N1 |
| A/turkey/Minnesota/22-011789-002-original/2022(H5N1) | 2020-2022 | North America | Domestic Galliformes | H5N1 |
| A/turkey/Minnesota/22-011796-001-original/2022(H5N1) | 2020-2022 | North America | Domestic Galliformes | H5N1 |
| A/turkey/Minnesota/22-011796-002-original/2022(H5N1) | 2020-2022 | North America | Domestic Galliformes | H5N1 |
| A/turkey/Minnesota/22-012034-001-original/2022(H5N1) | 2020-2022 | North America | Domestic Galliformes | H5N1 |
| A/chicken/Idaho/22-011775-002-original/2022(H5N1) | 2020-2022 | North America | Domestic Galliformes | H5N1 |
| A/chicken/Idaho/22-012045-001-original/2022(H5N1) | 2020-2022 | North America | Domestic Galliformes | H5N1 |
| A/chicken/Idaho/22-012045-002-original/2022(H5N1) | 2020-2022 | North America | Domestic Galliformes | H5N1 |
| A/chicken/North Dakota/22-011778-001-original/2022(H5N1) | 2020-2022 | North America | Domestic Galliformes | H5N1 |
| A/chicken/Pennsylvania/22-011920-001-original/2022(H5N1) | 2020-2022 | North America | Domestic Galliformes | H5N1 |
| A/chicken/Pennsylvania/22-011921-002-original/2022(H5N1) | 2020-2022 | North America | Domestic Galliformes | H5N1 |
| A/duck/Idaho/22-012046-001-original/2022(H5N1) | 2020-2022 | North America | Domestic Anseriformes | H5N1 |
| A/goose/Idaho/22-012047-001-original/2022(H5N1) | 2020-2022 | North America | Domestic Anseriformes | H5N1 |
| A/guinea fowl/Idaho/22-011775-001-original/2022(H5N1) | 2020-2022 | North America | Other wild species | H5N1 |
| A/muscovy duck/Florida/W22-306/2022(H5N1) | 2020-2022 | North America | Wild Anseriformes | H5N1 |
| A/red-shouldered hawk/Minnesota/22-012000-004-original/2022(H5N1) | 2020-2022 | North America | Other wild species | H5N1 |
| A/turkey/Minnesota/22-012036-001-original/2022(H5N1) | 2020-2022 | North America | Domestic Galliformes | H5N1 |
| A/turkey/Minnesota/22-012036-002-original/2022(H5N1) | 2020-2022 | North America | Domestic Galliformes | H5N1 |
| A/chicken/Colorado/22-012308-001-original/2022(H5N1) | 2020-2022 | North America | Domestic Galliformes | H5N1 |
| A/chicken/Colorado/22-012308-002-original/2022(H5N1) | 2020-2022 | North America | Domestic Galliformes | H5N1 |
| A/chicken/Montana/22-012040-001-original/2022(H5N1) | 2020-2022 | North America | Domestic Galliformes | H5N1 |
| A/chicken/North Dakota/22-012100-001-original/2022(H5N1) | 2020-2022 | North America | Domestic Galliformes | H5N1 |
| A/chicken/Pennsylvania/22-012091-001-original/2022(H5N1) | 2020-2022 | North America | Domestic Galliformes | H5N1 |
| A/chicken/Pennsylvania/22-012092-003-original/2022(H5N1) | 2020-2022 | North America | Domestic Galliformes | H5N1 |
| A/chicken/Pennsylvania/22-012092-005-original/2022(H5N1) | 2020-2022 | North America | Domestic Galliformes | H5N1 |
[truncated: 82,090 more chars]
