# Supplementary figures and images for: Spatiotemporal and Species-Crossing Transmission Dynamics of Subclade 2.3.4.4b H5Nx HPAIVs
Source: Transbound Emerg Dis. 2024 Jul 10;2024:2862053. doi: 10.1155/2024/2862053 (PMC12017169; doi:10.1155/2024/2862053)

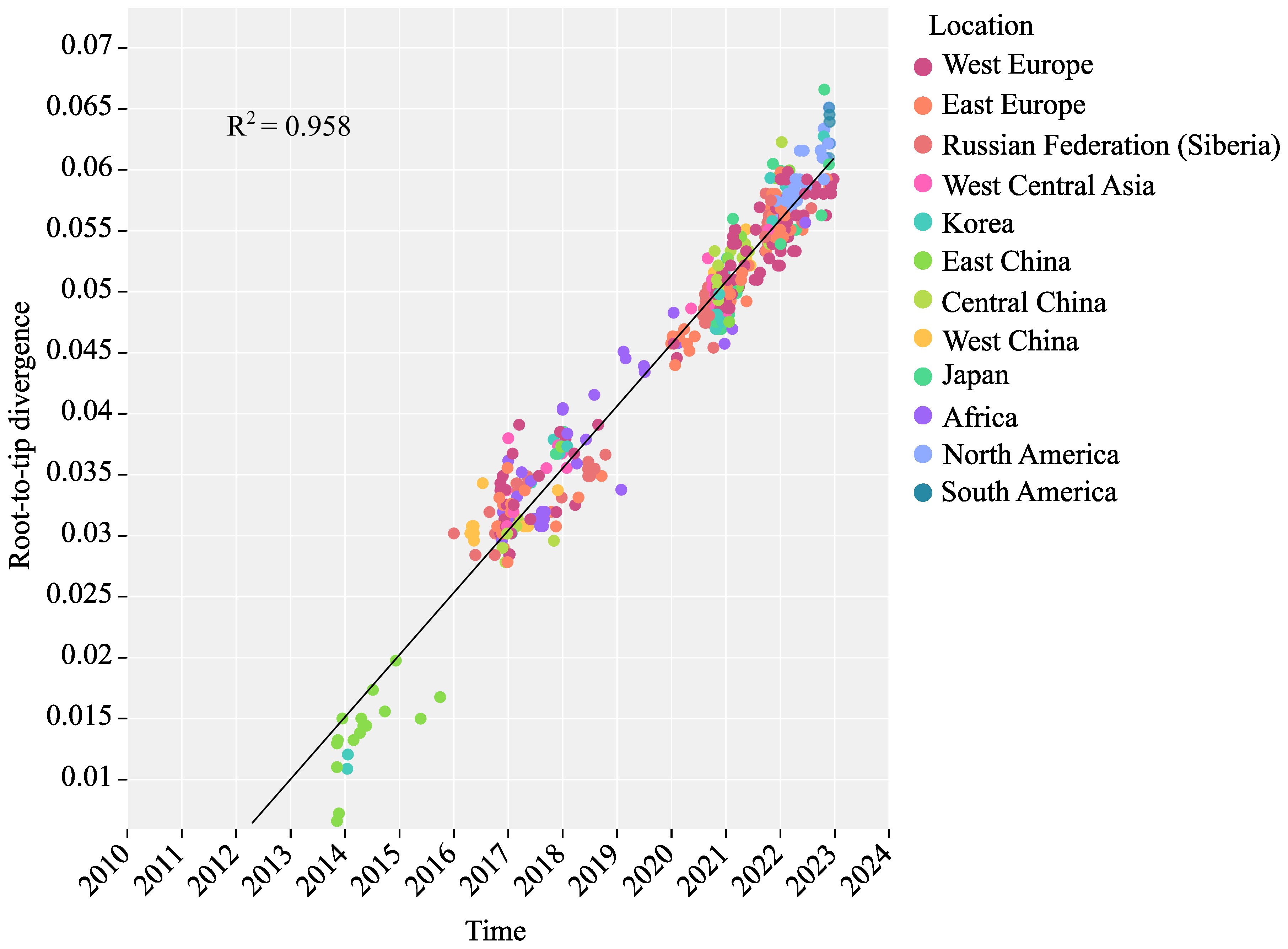

Supplement: Supplementary 2 — Figure 1: tempest of subC2344b H5Nx viruses. [file 2862053.f2.jpg]

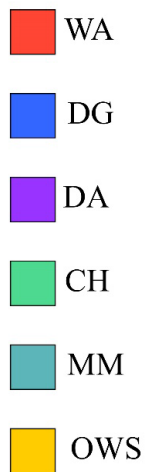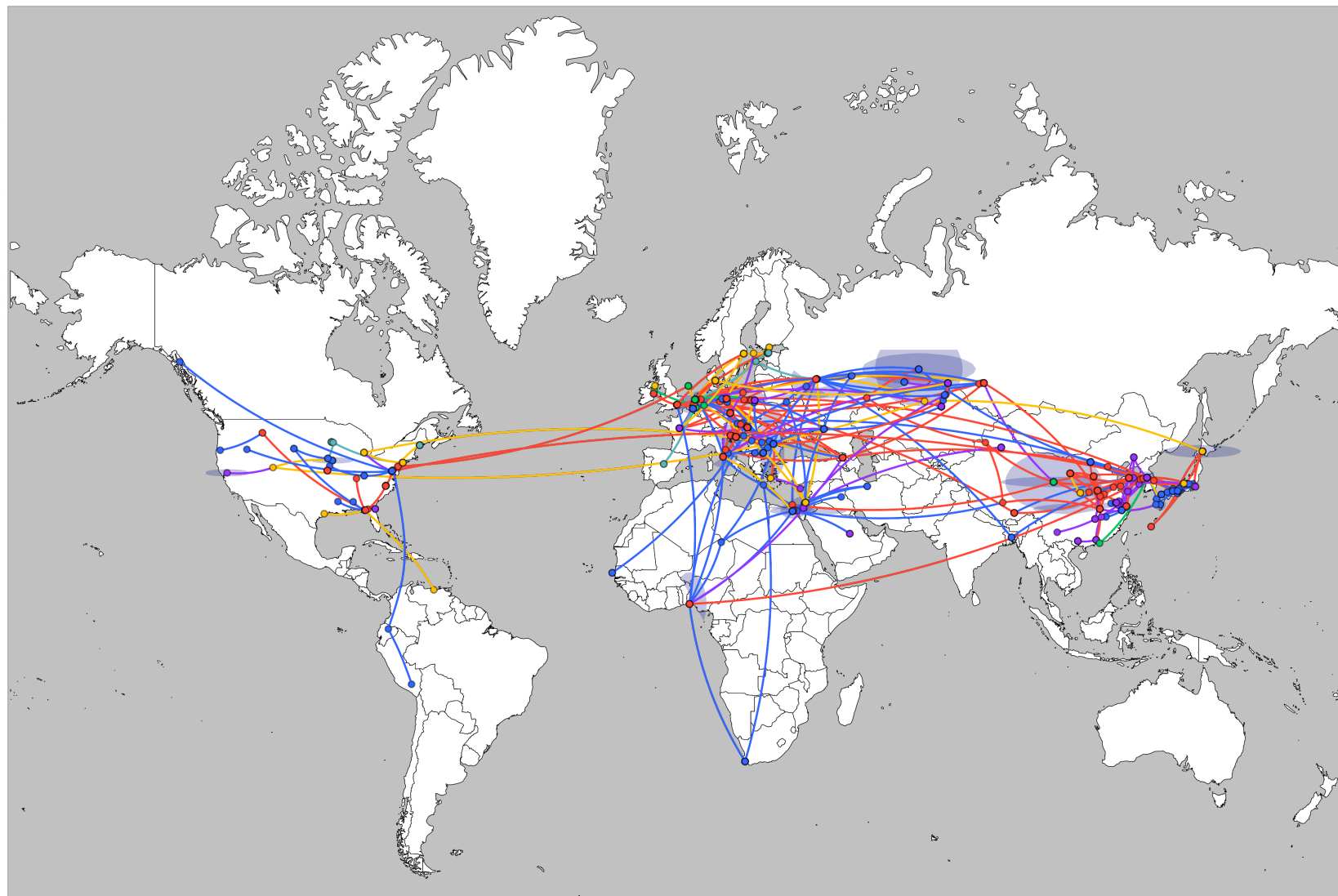

Supplement: Supplementary 11 — Figure 3: joint inference of host and region. [file 2862053.f11.pdf]
