# Supplementary material for: Spatiotemporal and Species-Crossing Transmission Dynamics of Subclade 2.3.4.4b H5Nx HPAIVs
Source: Transbound Emerg Dis. 2024 Jul 10;2024:2862053. doi: 10.1155/2024/2862053 (PMC12017169; doi:10.1155/2024/2862053)
Supplement: Supplementary 3 — Table 2: Bayes factor supports between regions. [file 2862053.f3.docx]

**Table S2.** Bayes factor supports. Bayes factor supports for individual transitions between discrete states inferred from the subC2344b H5Nx virus downsampled dataset. WEU- West Europe; EEU- East Europe; RUS- Russian Federation (Siberia); West Central Asia- WCAS; KR- Korea; ECHN- East China; WCHN- West China; CCHN- Central China; Africa- AF; Japan- JPN; North America- NA; South America-SA.

| **From** | **To** | **BF** | **PP** |
| --- | --- | --- | --- |
| KR | ECHN | 501496.00 | 1.00 |
| WEU | EEU | 501496.00 | 1.00 |
| KR | JPN | 501496.00 | 1.00 |
| NA | SA | 501496.00 | 1.00 |
| KR | CCHN | 6860.65 | 0.99 |
| WCAS | AF | 750.57 | 0.99 |
| RUS | WEU | 250.96 | 0.96 |
| RUS | WCAS | 226.50 | 0.96 |
| AF | RUS | 107.75 | 0.92 |
| RUS | KR | 94.37 | 0.91 |
| JPN | RUS | 96.16 | 0.92 |
| WCHN | CCHN | 85.28 | 0.90 |
| KR | WCHN | 65.02 | 0.88 |
| WEU | WCAS | 45.52 | 0.83 |
| WEU | AF | 42.71 | 0.82 |
| EEU | WEU | 26.77 | 0.74 |
| WEU | NA | 18.53 | 0.67 |
| ECHN | KR | 15.86 | 0.63 |
| RUS | EEU | 14.46 | 0.61 |
| RUS | WCHN | 10.92 | 0.54 |
| WCAS | CCHN | 9.36 | 0.50 |
