# Supplementary material for: Spatiotemporal and Species-Crossing Transmission Dynamics of Subclade 2.3.4.4b H5Nx HPAIVs
Source: Transbound Emerg Dis. 2024 Jul 10;2024:2862053. doi: 10.1155/2024/2862053 (PMC12017169; doi:10.1155/2024/2862053)
Supplement: Supplementary 4 — Table 3: Markov jumps for individual transitions between regions. [file 2862053.f4.docx]

**Table S3.** Markov jumps for individual transitions between discrete states.

| **Source** | **Sink** | | | | | | | | | | | |
| --- | --- | --- | --- | --- | --- | --- | --- | --- | --- | --- | --- | --- |
|  | **WEU** | **EEU** | **RUS** | **WCAS** | **KR** | **ECHN** | **CCHN** | **WCHN** | **JPN** | **AF** | **NA** | **SA** |
| **WEU** |  | 29.54 | 2.43 | 7.59 | 1.44 | 0.0040 | 0.00 | 0.02 | 0.12 | 4.86 | 0.75 | 0.00 |
| **EEU** | 6.16 |  | 0.72 | 0.01 | 0.62 | 0.00 | 0.00 | 0.01 | 0.00 | 0.28 | 0.18 | 0.00 |
| **RUS** | 32.61 | 5.76 |  | 14.90 | 7.22 | 0.01 | 0.02 | 4.57 | 0.02 | 0.52 | 0.02 | 0.00 |
| **WCAS** | 0.11 | 0.01 | 0.02 |  | 0.51 | 0.00 | 1.01 | 1.38 | 0.01 | 6.76 | 0.05 | 0.00 |
| **KR** | 0.01 | 0.00 | 0.04 | 0.02 |  | 10.42 | 8.39 | 5.87 | 11.15 | 0.62 | 0.01 | 0.00 |
| **ECHN** | 0.04 | 0.01 | 0.52 | 0.01 | 1.31 |  | 0.15 | 0.04 | 0.02 | 0.48 | 0.01 | 0.00 |
| **CCHN** | 0.00 | 0.00 | 0.03 | 0.01 | 0.01 | 0.30 |  | 1.46 | 0.00 | 0.03 | 0.00 | 0.00 |
| **WCHN** | 0.01 | 0.00 | 0.15 | 0.01 | 0.36 | 0.00 | 3.02 |  | 0.00 | 0.04 | 0.00 | 0.00 |
| **JPN** | 0.00 | 0.00 | 2.18 | 0.00 | 0.04 | 0.01 | 0.01 | 0.01 |  | 0.20 | 2.08 | 0.00 |
| **AF** | 0.17 | 0.82 | 4.30 | 0.03 | 0.01 | 0.00 | 0.01 | 0.09 | 0.00 |  | 0.02 | 0.00 |
| **NA** | 0.02 | 0.00 | 0.40 | 1.01 | 0.84 | 0.01 | 0.00 | 0.00 | 0.00 | 0.00 |  | 2.12 |
| **SA** | 0.00 | 0.00 | 0.01 | 0.02 | 0.44 | 0.01 | 0.00 | 0.00 | 0.00 | 0.00 | 0.00 |  |
