# Supplementary material for: Spatiotemporal and Species-Crossing Transmission Dynamics of Subclade 2.3.4.4b H5Nx HPAIVs
Source: Transbound Emerg Dis. 2024 Jul 10;2024:2862053. doi: 10.1155/2024/2862053 (PMC12017169; doi:10.1155/2024/2862053)
Supplement: Supplementary 5 — Table 4: migration rate for individual transitions between regions. [file 2862053.f5.docx]

**Table S4.** Migration rate for individual transitions between discrete states. Migration rate >1 is marked in bold.

| **Source** | **Sink** | | | | | | | | | | | |
| --- | --- | --- | --- | --- | --- | --- | --- | --- | --- | --- | --- | --- |
|  | **WEU** | **EEU** | **RUS** | **WCAS** | **KR** | **ECHN** | **CCHN** | **WCHN** | **JPN** | **AF** | **NA** | **SA** |
| **WEU** |  | **2.82** | 0.90 | 0.98 | 0.99 | 0.98 | 0.97 | **1.01** | 0.97 | 0.85 | 0.59 | 0.99 |
| **EEU** | 0.80 |  | **1.01** | 0.99 | 0.92 | 0.98 | **1.00** | 0.98 | 0.97 | 0.96 | 0.85 | 0.97 |
| **RUS** | **4.16** | 0.98 |  | **2.02** | **1.03** | 0.97 | 0.98 | 0.89 | 0.97 | 0.86 | 0.99 | 0.97 |
| **WCAS** | 0.98 | 0.96 | 0.95 |  | 0.94 | 0.97 | 0.73 | 0.82 | 0.99 | **1.01** | 0.97 | 0.98 |
| **KR** | 0.97 | 0.99 | 0.95 | 0.96 |  | **1.53** | **1.34** | **1.14** | **1.77** | 0.71 | **1.01** | 0.99 |
| **ECHN** | 0.96 | 0.97 | 0.73 | 0.98 | 0.63 |  | 0.95 | 0.98 | 0.95 | 0.88 | 0.97 | 0.98 |
| **CCHN** | 0.98 | 0.98 | 0.96 | 0.97 | 0.97 | 0.95 |  | 0.91 | 0.96 | 0.96 | 0.98 | 0.99 |
| **WCHN** | **1.01** | 0.97 | 0.92 | 0.98 | 0.96 | 0.98 | 0.77 |  | 0.97 | 0.98 | 0.98 | 0.98 |
| **JPN** | 0.97 | 0.97 | 0.68 | 0.97 | **1.00** | 0.99 | 0.97 | 0.99 |  | 0.88 | 0.50 | 0.98 |
| **AF** | 0.94 | 0.61 | 0.74 | 0.97 | **1.00** | **1.00** | 0.98 | 0.95 | 0.98 |  | 0.96 | 0.98 |
| **NA** | 0.99 | 0.98 | 0.87 | 0.48 | 0.52 | 0.98 | 0.99 | **1.00** | **1.01** | 0.98 |  | 0.54 |
| **SA** | 0.94 | 0.95 | 0.95 | 0.97 | 0.74 | 0.97 | 0.96 | 0.94 | 0.97 | 0.94 | 0.95 |  |
