# Supplementary material for: Spatiotemporal and Species-Crossing Transmission Dynamics of Subclade 2.3.4.4b H5Nx HPAIVs
Source: Transbound Emerg Dis. 2024 Jul 10;2024:2862053. doi: 10.1155/2024/2862053 (PMC12017169; doi:10.1155/2024/2862053)
Supplement: Supplementary 6 — Figure 2: MCC tree of eight genes of subC2344b H5Nx viruses. [file 2862053.f6.pdf]

Fig.S2A
HA

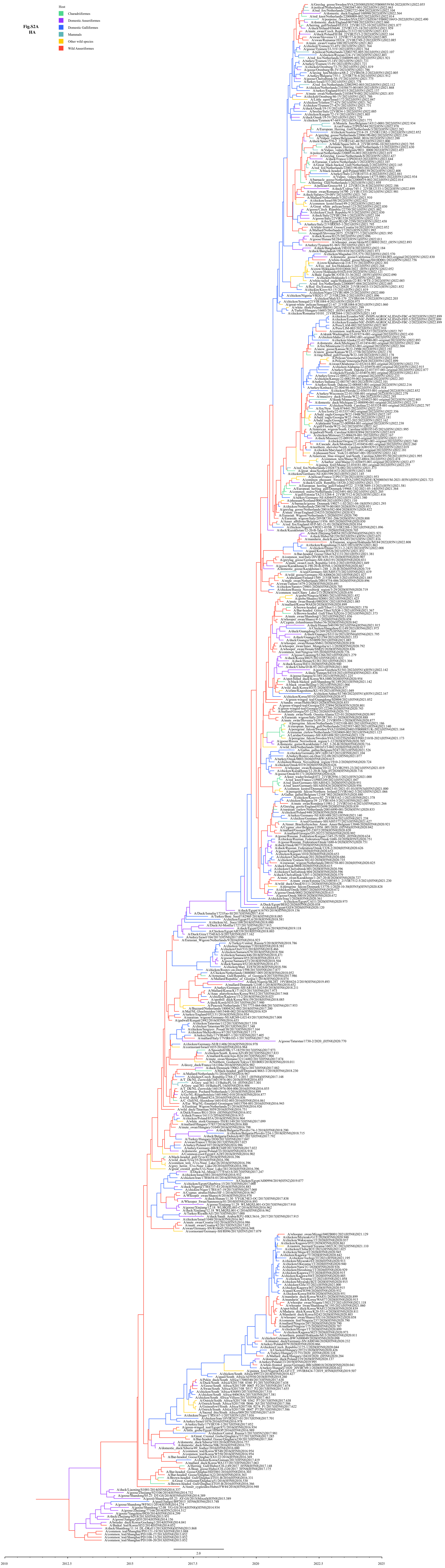

Host
Charadriiformes
Domestic Anseriformes
Domestic Galliformes
Mammals
Other wild species
Wild Anseriformes
2.0
2010
2012.5
2015
2017.5
2020
2022.5
2025

Fig.S2B
N1

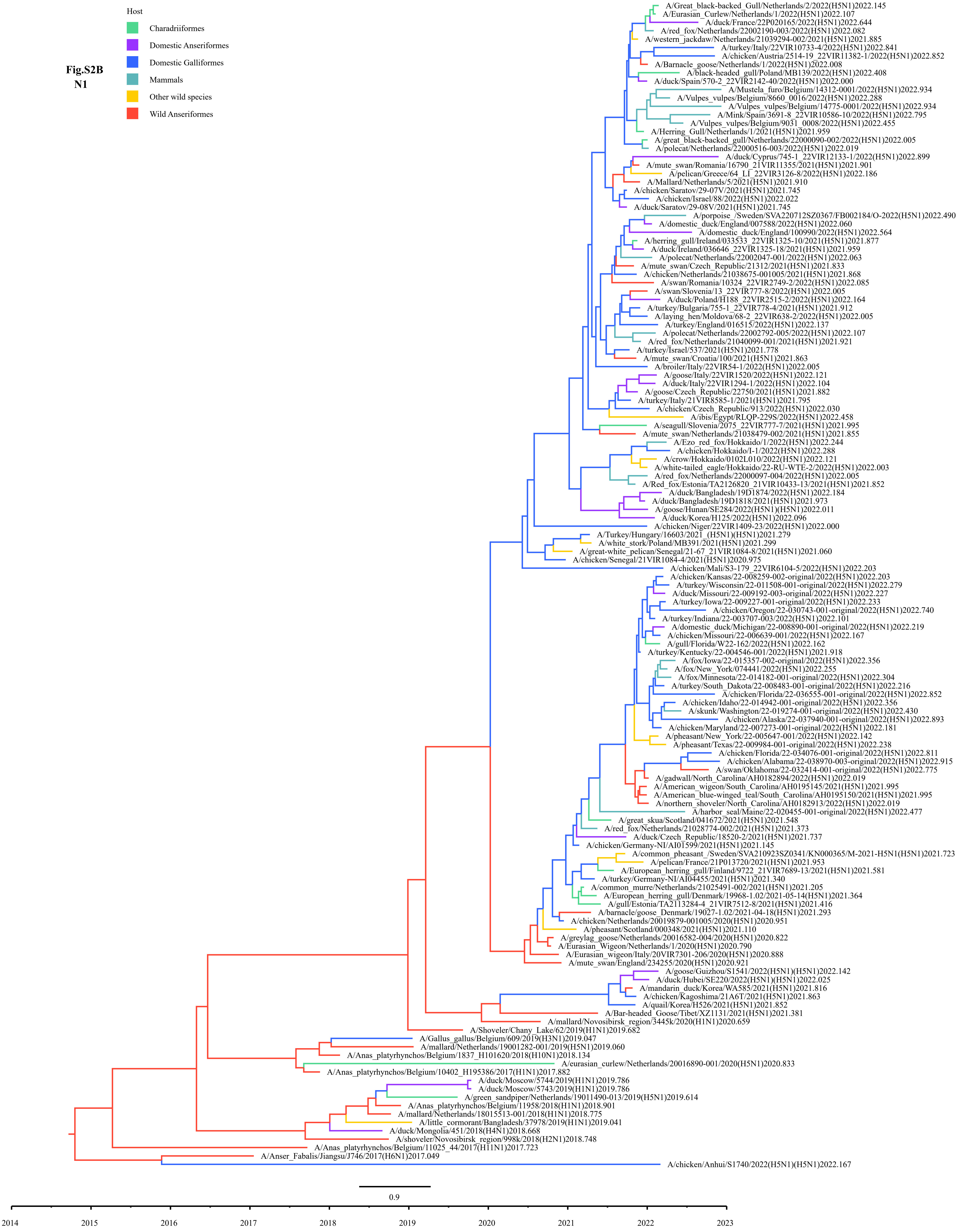
Host
Charadriiformes
Domestic Anseriformes
Domestic Galliformes
Mammals
Other wild species
Wild Anseriformes
A/Great_black-backed_Gull/Netherlands/2/2022(H5N1)2022.145
A/Eurasian_Curlew/Netherlands/1/2022(H5N1)2022.107
A/duck/France/22P020165/2022(H5N1)2022.644
A/red_fox/Netherlands/22002190-003/2022(H5N1)2022.082
A/western_jackdaw/Netherlands/21039294-002/2021(H5N1)2021.885
A/turkey/Italy/22VIR10733-4/2022(H5N1)2022.841
A/chicken/Austria/2514-19_22VIR11382-1/2022(H5N1)2022.852
A/Barnacle_goose/Netherlands/1/2022(H5N1)2022.008
A/black-headed_gull/Poland/MB139/2022(H5N1)2022.408
A/duck/Spain/570-2_22VIR2142-40/2022(H5N1)2022.000
A/Mustela_furo/Belgium/14312-0001/2022(H5N1)2022.934
A/Vulpes_vulpes/Belgium/8660_0016/2022(H5N1)2022.288
A/Vulpes_vulpes/Belgium/14775-0001/2022(H5N1)2022.934
A/Mink/Spain/3691-8_22VIR10586-10/2022(H5N1)2022.795
A/Vulpes_vulpes/Belgium/9031_0008/2022(H5N1)2022.455
A/Herring_Gull/Netherlands/1/2021(H5N1)2021.959
A/great_black-backed_gull/Netherlands/22000090-002/2022(H5N1)2022.005
A/polecat/Netherlands/22000516-003/2022(H5N1)2022.019
A/duck/Cyprus/745-1_22VIR12133-1/2022(H5N1)2022.899
A/mute_swan/Romania/16790_21VIR11355/2021(H5N1)2021.901
A/pelican/Greece/64_LI_22VIR3126-8/2022(H5N1)2022.186
A/Mallard/Netherlands/5/2021(H5N1)2021.910
A/chicken/Saratov/29-07V/2021(H5N1)2021.745
A/chicken/Israel/88/2022(H5N1)2022.022
A/duck/Saratov/29-08V/2021(H5N1)2021.745
A/porpoise_/Sweden/SVA220712SZ0367/FB002184/O-2022(H5N1)2022.490
A/domestic_duck/England/007588/2022(H5N1)2022.060
A/domestic_duck/England/100990/2022(H5N1)2022.564
A/herring_gull/Ireland/033533_22VIR1325-10/2021(H5N1)2021.877
A/duck/Ireland/036646_22VIR1325-18/2021(H5N1)2021.959
A/polecat/Netherlands/22002047-001/2022(H5N1)2022.063
A/mute_swan/Czech_Republic/21312/2021(H5N1)2021.833
A/chicken/Netherlands/21038675-001005/2021(H5N1)2021.868
A/swan/Romania/10324_22VIR2749-2/2022(H5N1)2022.085
A/swan/Slovenia/13_22VIR777-8/2022(H5N1)2022.005
A/duck/Poland/H188_22VIR2515-2/2022(H5N1)2022.164
A/turkey/Bulgaria/755-1_22VIR778-4/2021(H5N1)2021.912
A/laying_hen/Moldova/68-2_22VIR638-2/2022(H5N1)2022.005
A/turkey/England/016515/2022(H5N1)2022.137
A/polecat/Netherlands/22002792-005/2022(H5N1)2022.107
A/red_fox/Netherlands/21040099-001/2021(H5N1)2021.921
A/turkey/Israel/537/2021(H5N1)2021.778
A/mute_swan/Croatia/100/2021(H5N1)2021.863
A/broiler/Italy/22VIR54-1/2022(H5N1)2022.005
A/goose/Italy/22VIR1520/2022(H5N1)2022.121
A/duck/Italy/22VIR1294-1/2022(H5N1)2022.104
A/goose/Czech_Republic/22750/2021(H5N1)2021.882
A/turkey/Italy/21VIR8585-1/2021(H5N1)2021.795
A/chicken/Czech_Republic/913/2022(H5N1)2022.030
A/ibis/Egypt/RLQP-229S/2022(H5N1)2022.458
A/seagull/Slovenia/2075_22VIR777-7/2021(H5N1)2021.995
A/mute_swan/Netherlands/21038479-002/2021(H5N1)2021.855
A/Ezo_red_fox/Hokkaido/1/2022(H5N1)2022.244
A/chicken/Hokkaido/I-1/2022(H5N1)2022.288
A/crow/Hokkaido/0102L010/2022(H5N1)2022.121
A/white-tailed_eagle/Hokkaido/22-RU-WTE-2/2022(H5N1)2022.003
A/red_fox/Netherlands/22000097-004/2022(H5N1)2022.005
A/Red_fox/Estonia/TA2126820_21VIR10433-13/2021(H5N1)2021.852
A/duck/Bangladesh/19D1874/2022(H5N1)2022.184
A/duck/Bangladesh/19D1818/2021(H5N1)2021.973
A/goose/Hunan/SE284/2022(H5N1)(H5N1)2022.011
A/duck/Korea/H125/2022(H5N1)2022.096
A/chicken/Niger/22VIR1409-23/2022(H5N1)2022.000
A/Turkey/Hungary/16603/2021_(H5N1)(H5N1)2021.279
A/white_stork/Poland/MB391/2021(H5N1)2021.299
A/great-white_pelican/Senegal/21-67_21VIR1084-8/2021(H5N1)2021.060
A/chicken/Senegal/21VIR1084-4/2021(H5N1)2020.975
A/chicken/Mali/S3-179_22VIR6104-5/2022(H5N1)2022.203
A/chicken/Kansas/22-008259-002-original/2022(H5N1)2022.203
A/turkey/Wisconsin/22-011508-001-original/2022(H5N1)2022.279
A/duck/Missouri/22-009192-003-original/2022(H5N1)2022.227
A/turkey/Iowa/22-009227-001-original/2022(H5N1)2022.233
A/chicken/Oregon/22-030743-001-original/2022(H5N1)2022.740
A/turkey/Indiana/22-003707-003/2022(H5N1)2022.101
A/domestic_duck/Michigan/22-008890-001-original/2022(H5N1)2022.219
A/chicken/Missouri/22-006639-001/2022(H5N1)2022.167
A/gull/Florida/W22-162/2022(H5N1)2022.162
A/turkey/Kentucky/22-004546-001/2022(H5N1)2021.918
A/fox/Iowa/22-015357-002-original/2022(H5N1)2022.356
A/fox/New_York/074441/2022(H5N1)2022.255
A/fox/Minnesota/22-014182-001-original/2022(H5N1)2022.304
A/turkey/South_Dakota/22-008483-001-original/2022(H5N1)2022.216
A/chicken/Florida/22-036555-001-original/2022(H5N1)2022.852
A/chicken/Idaho/22-014942-001-original/2022(H5N1)2022.356
A/skunk/Washington/22-019274-001-original/2022(H5N1)2022.430
A/chicken/Alaska/22-037940-001-original/2022(H5N1)2022.893
A/chicken/Maryland/22-007273-001-original/2022(H5N1)2022.181
A/pheasant/New_York/22-005647-001/2022(H5N1)2022.142
A/pheasant/Texas/22-009984-001-original/2022(H5N1)2022.238
A/chicken/Florida/22-034076-001-original/2022(H5N1)2022.811
A/chicken/Alabama/22-038970-003-original/2022(H5N1)2022.915
A/swan/Oklahoma/22-032414-001-original/2022(H5N1)2022.775
A/gadwall/North_Carolina/AH0182894/2022(H5N1)2022.019
A/American_wigeon/South_Carolina/AH0195145/2021(H5N1)2021.995
A/American_blue-winged_teal/South_Carolina/AH0195150/2021(H5N1)2021.995
A/northern_shoveler/North_Carolina/AH0182913/2022(H5N1)2022.019
A/harbor_seal/Maine/22-020455-001-original/2022(H5N1)2022.477
A/great_skua/Scotland/041672/2021(H5N1)2021.548
A/red_fox/Netherlands/21028774-002/2021(H5N1)2021.373
A/duck/Czech_Republic/18520-2/2021(H5N1)2021.737
A/chicken/Germany-NI/AI01599/2021(H5N1)2021.145
A/common_pheasant_/Sweden/SVA210923SZ0341/KN000365/M-2021-H5N1(H5N1)2021.723
A/pelican/France/21P013720/2021(H5N1)2021.953
A/European_herring_gull/Finland/9722_21VIR7689-13/2021(H5N1)2021.581
A/turkey/Germany-NI/AI04455/2021(H5N1)2021.340
A/common_murre/Netherlands/21025491-002/2021(H5N1)2021.205
A/European_herring_gull/Denmark/19968-1.02/2021-05-14(H5N1)2021.364
A/gull/Estonia/TA2113284-4_21VIR7512-8/2021(H5N1)2021.416
A/barnacle/goose_Denmark/19027-1.02/2021-04-18(H5N1)2021.293
A/chicken/Netherlands/20019879-001005/2020(H5N1)2020.951
A/pheasant/Scotland/000348/2021(H5N1)2021.110
A/greylag_goose/Netherlands/20016582-004/2020(H5N1)2020.822
A/Eurasian_Wigeon/Netherlands/1/2020(H5N1)2020.790
A/Eurasian_wigeon/Italy/20VIR7301-206/2020(H5N1)2020.888
A/mute_swan/England/234255/2020(H5N1)2020.921
A/goose/Guizhou/S1541/2022(H5N1)(H5N1)2022.142
A/duck/Hubei/SE220/2022(H5N1)(H5N1)2022.025
A/mandarin_duck/Korea/WA585/2021(H5N1)2021.816
A/chicken/Kagoshima/21A6T/2021(H5N1)2021.863
A/quail/Korea/H526/2021(H5N1)2021.852
A/Bar-headed_Goose/Tibet/XZ1131/2021(H5N1)2021.381
A/mallard/Novosibirsk_region/3445k/2020(H1N1)2020.659
A/Shoveler/Chany_Lake/62/2019(H1N1)2019.682
A/Gallus_gallus/Belgium/609/2019(H3N1)2019.047
A/mallard/Netherlands/19001282-001/2019(H5N1)2019.060
A/Anas_platyrhynchos/Belgium/1837_H101620/2018(H10N1)2018.134
A/eurasian_curlew/Netherlands/20016890-001/2020(H5N1)2020.833
A/Anas_platyrhynchos/Belgium/10402_H195386/2017(H1N1)2017.882
A/duck/Moscow/5744/2019(H1N1)2019.786
A/duck/Moscow/5743/2019(H1N1)2019.786
A/green_sandpiper/Netherlands/19011490-013/2019(H5N1)2019.614
A/Anas_platyrhynchos/Belgium/11958/2018(H1N1)2018.901
A/mallard/Netherlands/18015513-001/2018(H1N1)2018.775
A/little_cormorant/Bangladesh/37978/2019(H1N1)2019.041
A/duck/Mongolia/451/2018(H4N1)2018.668
A/shoveler/Novosibirsk_region/998k/2018(H2N1)2018.748
A/Anas_platyrhynchos/Belgium/11025_44/2017(H11N1)2017.723
A/Anser_Fabalis/Jiangsu/J746/2017(H6N1)2017.049
A/chicken/Anhui/S1740/2022(H5N1)(H5N1)2022.167
0.9
2014
2015
2016
2017
2018
2019
2020
2021
2022
2023

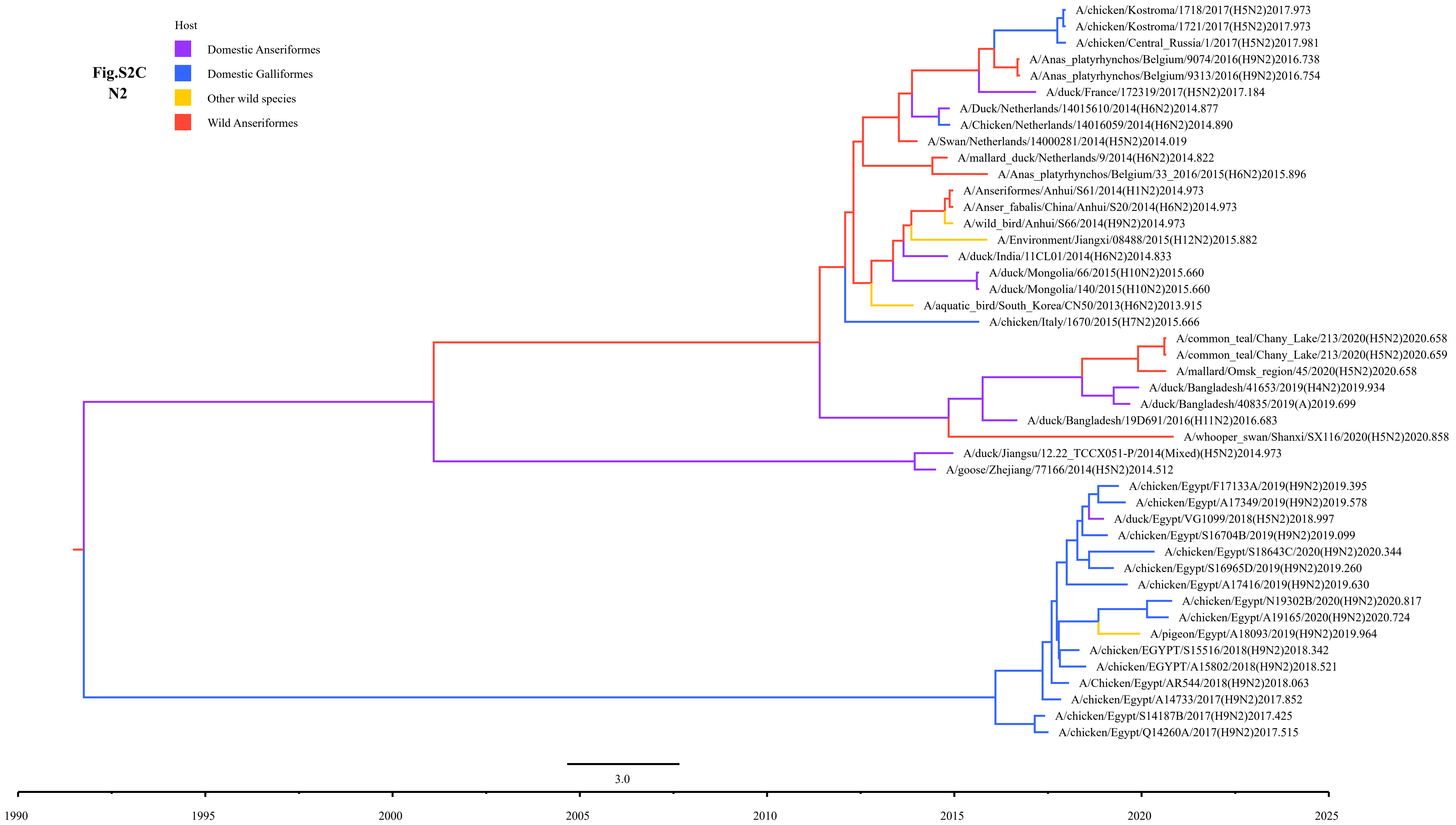
Fig.S2C
N2
Host
Domestic Anseriformes
Domestic Galliformes
Other wild species
Wild Anseriformes
A/chicken/Kostroma/1718/2017(H5N2)2017.973
A/chicken/Kostroma/1721/2017(H5N2)2017.973
A/chicken/Central_Russia/1/2017(H5N2)2017.981
A/Anas_platyrhynchos/Belgium/9074/2016(H9N2)2016.738
A/Anas_platyrhynchos/Belgium/9313/2016(H9N2)2016.754
A/duck/France/172319/2017(H5N2)2017.184
A/Duck/Netherlands/14015610/2014(H6N2)2014.877
A/Chicken/Netherlands/14016059/2014(H6N2)2014.890
A/Swan/Netherlands/14000281/2014(H5N2)2014.019
A/mallard_duck/Netherlands/9/2014(H6N2)2014.822
A/Anas_platyrhynchos/Belgium/33_2016/2015(H6N2)2015.896
A/Anseriformes/Anhui/S61/2014(H1N2)2014.973
A/Anser_fabalis/China/Anhui/S20/2014(H6N2)2014.973
A/wild_bird/Anhui/S66/2014(H9N2)2014.973
A/Environment/Jiangxi/08488/2015(H12N2)2015.882
A/duck/India/11CL01/2014(H6N2)2014.833
A/duck/Mongolia/66/2015(H10N2)2015.660
A/duck/Mongolia/140/2015(H10N2)2015.660
A/aquatic_bird/South_Korea/CN50/2013(H6N2)2013.915
A/chicken/Italy/1670/2015(H7N2)2015.666
A/common_teal/Chany_Lake/213/2020(H5N2)2020.658
A/common_teal/Chany_Lake/213/2020(H5N2)2020.659
A/mallard/Omsk_region/45/2020(H5N2)2020.658
A/duck/Bangladesh/41653/2019(H4N2)2019.934
A/duck/Bangladesh/40835/2019(A)2019.699
A/duck/Bangladesh/19D691/2016(H11N2)2016.683
A/whooper_swan/Shanxi/SX116/2020(H5N2)2020.858
A/duck/Jiangsu/12.22_TCCX051-P/2014(Mixed)(H5N2)2014.973
A/goose/Zhejiang/77166/2014(H5N2)2014.512
A/chicken/Egypt/F17133A/2019(H9N2)2019.395
A/chicken/Egypt/A17349/2019(H9N2)2019.578
A/duck/Egypt/VG1099/2018(H5N2)2018.997
A/chicken/Egypt/S16704B/2019(H9N2)2019.099
A/chicken/Egypt/S18643C/2020(H9N2)2020.344
A/chicken/Egypt/S16965D/2019(H9N2)2019.260
A/chicken/Egypt/A17416/2019(H9N2)2019.630
A/chicken/Egypt/N19302B/2020(H9N2)2020.817
A/chicken/Egypt/A19165/2020(H9N2)2020.724
A/pigeon/Egypt/A18093/2019(H9N2)2019.964
A/chicken/EGYPT/S15516/2018(H9N2)2018.342
A/chicken/EGYPT/A15802/2018(H9N2)2018.521
A/Chicken/Egypt/AR544/2018(H9N2)2018.063
A/chicken/Egypt/A14733/2017(H9N2)2017.852
A/chicken/Egypt/S14187B/2017(H9N2)2017.425
A/chicken/Egypt/Q14260A/2017(H9N2)2017.515
3.0
1990
1995
2000
2005
2010
2015
2020
2025

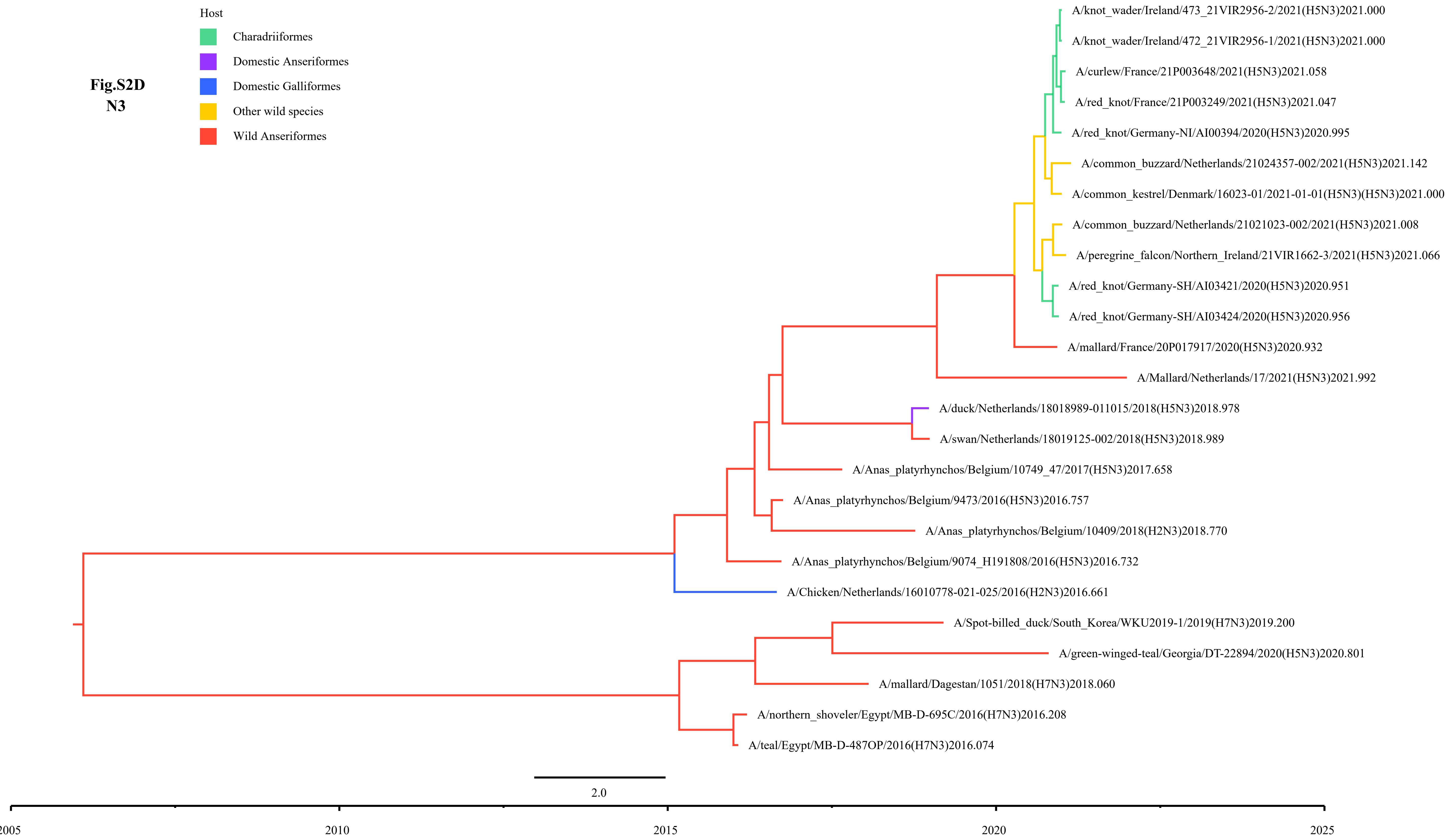

Host
Charadriiformes
Domestic Anseriformes
Domestic Galliformes
Other wild species
Wild Anseriformes
Fig.S2D
N3
A/knot_wader/Ireland/473_21VIR2956-2/2021(H5N3)2021.000
A/knot_wader/Ireland/472_21VIR2956-1/2021(H5N3)2021.000
A/curlew/France/21P003648/2021(H5N3)2021.058
A/red_knot/France/21P003249/2021(H5N3)2021.047
A/red_knot/Germany-NI/AI00394/2020(H5N3)2020.995
A/common_buzzard/Netherlands/21024357-002/2021(H5N3)2021.142
A/common_kestrel/Denmark/16023-01/2021-01-01(H5N3)(H5N3)2021.000
A/common_buzzard/Netherlands/21021023-002/2021(H5N3)2021.008
A/peregrine_falcon/Northern_Ireland/21VIR1662-3/2021(H5N3)2021.066
A/red_knot/Germany-SH/AI03421/2020(H5N3)2020.951
A/red_knot/Germany-SH/AI03424/2020(H5N3)2020.956
A/mallard/France/20P017917/2020(H5N3)2020.932
A/Mallard/Netherlands/17/2021(H5N3)2021.992
A/duck/Netherlands/18018989-011015/2018(H5N3)2018.978
A/swan/Netherlands/18019125-002/2018(H5N3)2018.989
A/Anas_platyrhynchos/Belgium/10749_47/2017(H5N3)2017.658
A/Anas_platyrhynchos/Belgium/9473/2016(H5N3)2016.757
A/Anas_platyrhynchos/Belgium/10409/2018(H2N3)2018.770
A/Anas_platyrhynchos/Belgium/9074_H191808/2016(H5N3)2016.732
A/Chicken/Netherlands/16010778-021-025/2016(H2N3)2016.661
A/Spot-billed_duck/South_Korea/WKU2019-1/2019(H7N3)2019.200
A/green-winged-teal/Georgia/DT-22894/2020(H5N3)2020.801
A/mallard/Dagestan/1051/2018(H7N3)2018.060
A/northern_shoveler/Egypt/MB-D-695C/2016(H7N3)2016.208
A/teal/Egypt/MB-D-487OP/2016(H7N3)2016.074
2.0
2005
2010
2015
2020
2025

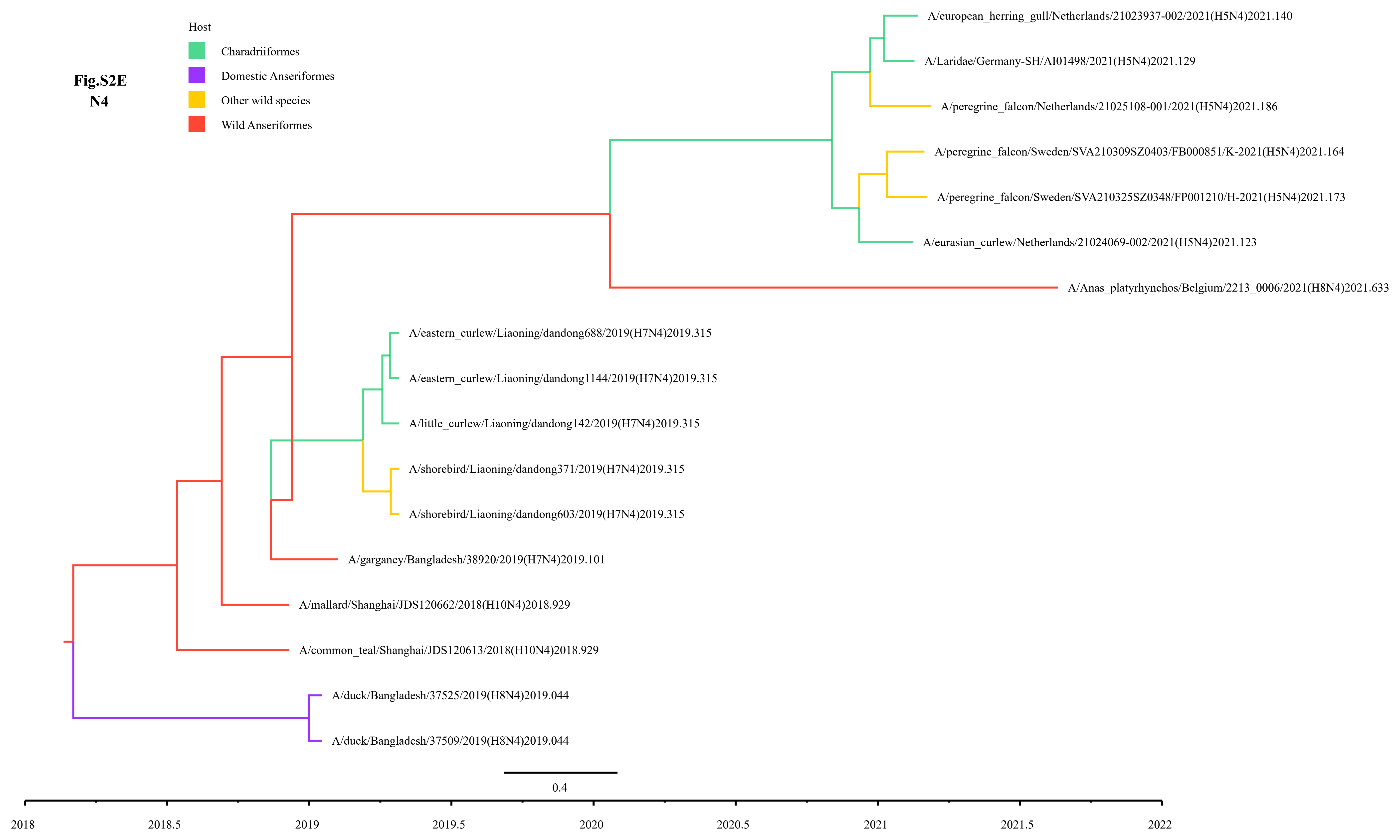
Fig.S2E
N4
Host
Charadriiformes
Domestic Anseriformes
Other wild species
Wild Anseriformes
A/european_herring_gull/Netherlands/21023937-002/2021(H5N4)2021.140
A/Laridae/Germany-SH/AI01498/2021(H5N4)2021.129
A/peregrine_falcon/Netherlands/21025108-001/2021(H5N4)2021.186
A/peregrine_falcon/Sweden/SVA210309SZ0403/FB000851/K-2021(H5N4)2021.164
A/peregrine_falcon/Sweden/SVA210325SZ0348/FP001210/H-2021(H5N4)2021.173
A/eurasian_curlew/Netherlands/21024069-002/2021(H5N4)2021.123
A/Anas_platyrhynchos/Belgium/2213_0006/2021(H8N4)2021.633
A/eastern_curlew/Liaoning/dandong688/2019(H7N4)2019.315
A/eastern_curlew/Liaoning/dandong1144/2019(H7N4)2019.315
A/little_curlew/Liaoning/dandong142/2019(H7N4)2019.315
A/shorebird/Liaoning/dandong371/2019(H7N4)2019.315
A/shorebird/Liaoning/dandong603/2019(H7N4)2019.315
A/garganey/Bangladesh/38920/2019(H7N4)2019.101
A/mallard/Shanghai/JDS120662/2018(H10N4)2018.929
A/common_teal/Shanghai/JDS120613/2018(H10N4)2018.929
A/duck/Bangladesh/37525/2019(H8N4)2019.044
A/duck/Bangladesh/37509/2019(H8N4)2019.044
0.4
2018
2018.5
2019
2019.5
2020
2020.5
2021
2021.5
2022

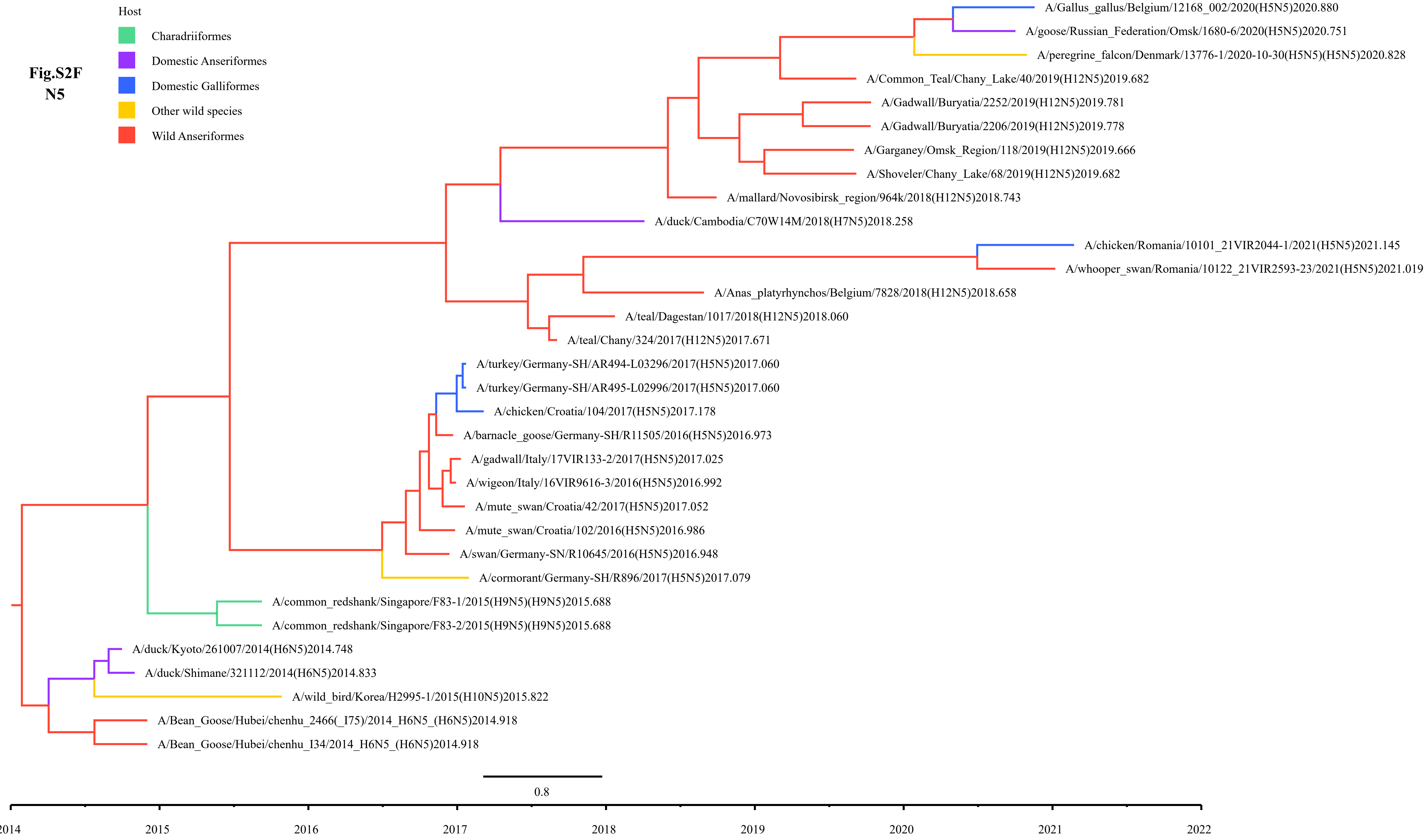
Host
Charadriiformes
Domestic Anseriformes
Domestic Galliformes
Other wild species
Wild Anseriformes
Fig.S2F
N5
A/Gallus_gallus/Belgium/12168_002/2020(H5N5)2020.880
A/goose/Russian_Federation/Omsk/1680-6/2020(H5N5)2020.751
A/peregrine_falcon/Denmark/13776-1/2020-10-30(H5N5)(H5N5)2020.828
A/Common_Teal/Chany_Lake/40/2019(H12N5)2019.682
A/Gadwall/Buryatia/2252/2019(H12N5)2019.781
A/Gadwall/Buryatia/2206/2019(H12N5)2019.778
A/Garganey/Omsk_Region/118/2019(H12N5)2019.666
A/Shoveler/Chany_Lake/68/2019(H12N5)2019.682
A/mallard/Novosibirsk_region/964k/2018(H12N5)2018.743
A/duck/Cambodia/C70W14M/2018(H7N5)2018.258
A/chicken/Romania/10101_21VIR2044-1/2021(H5N5)2021.145
A/whooper_swan/Romania/10122_21VIR2593-23/2021(H5N5)2021.019
A/Anas_platyrhynchos/Belgium/7828/2018(H12N5)2018.658
A/teal/Dagestan/1017/2018(H12N5)2018.060
A/teal/Chany/324/2017(H12N5)2017.671
A/turkey/Germany-SH/AR494-L03296/2017(H5N5)2017.060
A/turkey/Germany-SH/AR495-L02996/2017(H5N5)2017.060
A/chicken/Croatia/104/2017(H5N5)2017.178
A/barnacle_goose/Germany-SH/R11505/2016(H5N5)2016.973
A/gadwall/Italy/17VIR133-2/2017(H5N5)2017.025
A/wigeon/Italy/16VIR9616-3/2016(H5N5)2016.992
A/mute_swan/Croatia/42/2017(H5N5)2017.052
A/mute_swan/Croatia/102/2016(H5N5)2016.986
A/swan/Germany-SN/R10645/2016(H5N5)2016.948
A/cormorant/Germany-SH/R896/2017(H5N5)2017.079
A/common_redshank/Singapore/F83-1/2015(H9N5)(H9N5)2015.688
A/common_redshank/Singapore/F83-2/2015(H9N5)(H9N5)2015.688
A/duck/Kyoto/261007/2014(H6N5)2014.748
A/duck/Shimane/321112/2014(H6N5)2014.833
A/wild_bird/Korea/H2995-1/2015(H10N5)2015.822
A/Bean_Goose/Hubei/chenhu_2466(_I75)/2014_H6N5_(H6N5)2014.918
A/Bean_Goose/Hubei/chenhu_I34/2014_H6N5_(H6N5)2014.918
0.8
2014
2015
2016
2017
2018
2019
2020
2021
2022

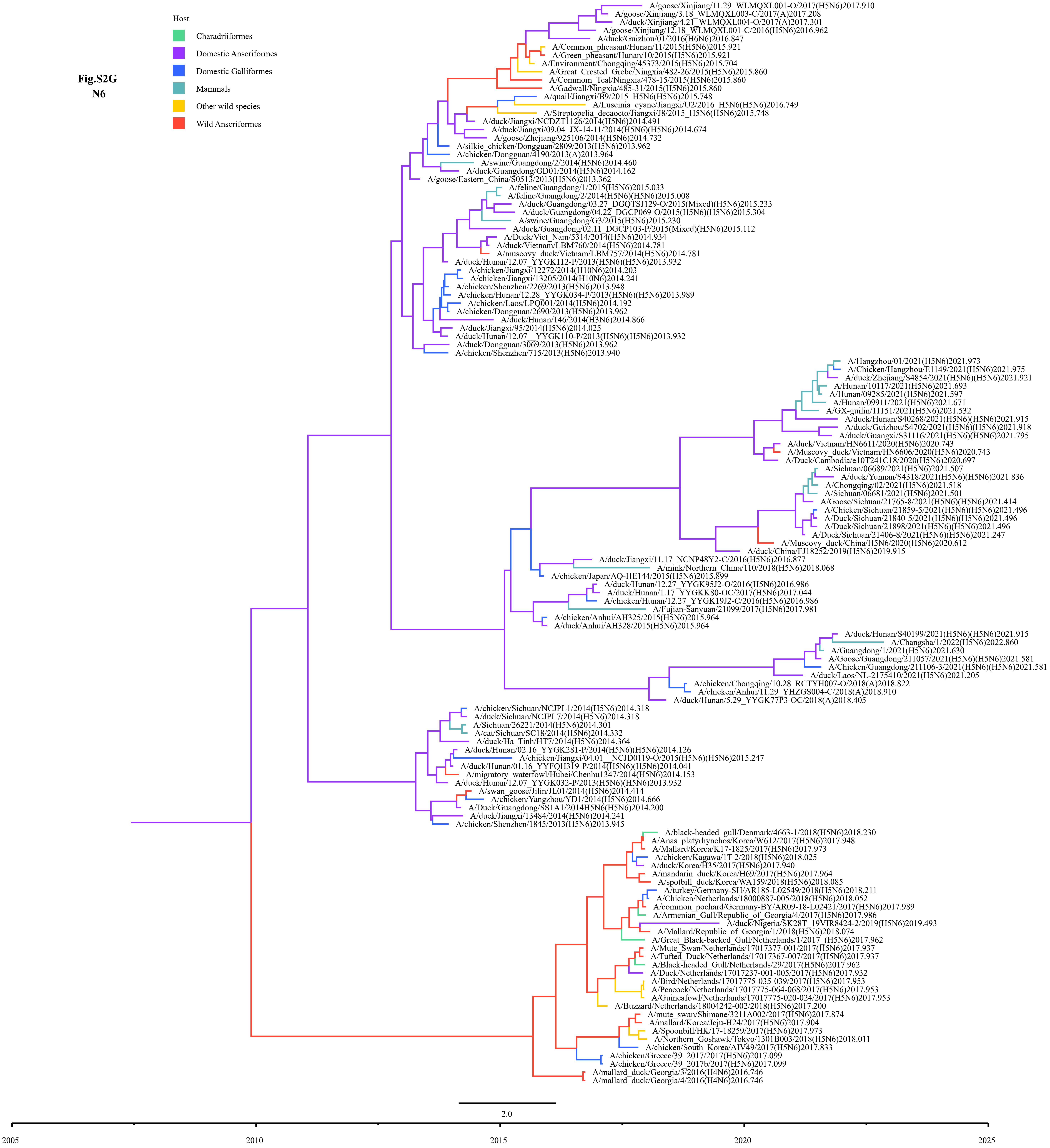
Host
Charadriiformes
Domestic Anseriformes
Domestic Galliformes
Mammals
Other wild species
Wild Anseriformes
A/goose/Xinjiang/11.29_WLMQXL001-O/2017(H5N6)2017.910
A/goose/Xinjiang/3.18_WLMQXL003-C/2017(A)2017.208
A/duck/Xinjiang/4.21_WLMQXL004-O/2017(A)2017.301
A/goose/Xinjiang/12.18_WLMQXL001-C/2016(H5N6)2016.962
A/duck/Guizhou/01/2016(H6N6)2016.847
A/Common_pheasant/Hunan/11/2015(H5N6)2015.921
A/Green_pheasant/Hunan/10/2015(H5N6)2015.921
A/Environment/Chongqing/45373/2015(H5N6)2015.704
A/Great_Crested_Grebe/Ningxia/482-26/2015(H5N6)2015.860
A/Commom_Teal/Ningxia/478-15/2015(H5N6)2015.860
A/Gadwall/Ningxia/485-31/2015(H5N6)2015.860
A/quail/Jiangxi/B9/2015_H5N6(H5N6)2015.748
A/Luscinia_cyane/Jiangxi/U2/2016_H5N6(H5N6)2016.749
A/Streptopelia_decaocto/Jiangxi/J8/2015_H5N6(H5N6)2015.748
A/duck/Jiangxi/NCDZT1126/2014(H5N6)2014.491
A/duck/Jiangxi/09.04_JX-14-11/2014(H5N6)(H5N6)2014.674
A/goose/Zhejiang/925106/2014(H5N6)2014.732
A/silkie_chicken/Dongguan/2809/2013(H5N6)2013.962
A/chicken/Dongguan/4190/2013(A)2013.964
A/swine/Guangdong/2/2014(H5N6)2014.460
A/duck/Guangdong/GD01/2014(H5N6)2014.162
A/goose/Eastern_China/S0513/2013(H5N6)2013.362
A/feline/Guangdong/1/2015(H5N6)2015.033
A/feline/Guangdong/2/2014(H5N6)(H5N6)2015.008
A/duck/Guangdong/03.27_DGQTSJ129-O/2015(Mixed)(H5N6)2015.233
A/duck/Guangdong/04.22_DGCP069-O/2015(H5N6)(H5N6)2015.304
A/swine/Guangdong/G3/2015(H5N6)2015.230
A/duck/Guangdong/02.11_DGCP103-P/2015(Mixed)(H5N6)2015.112
A/Duck/Viet_Nam/5314/2014(H5N6)2014.934
A/duck/Vietnam/LBM760/2014(H5N6)2014.781
A/muscovy_duck/Vietnam/LBM757/2014(H5N6)2014.781
A/duck/Hunan/12.07_YYGK112-P/2013(H5N6)(H5N6)2013.932
A/chicken/Jiangxi/12272/2014(H10N6)2014.203
A/chicken/Jiangxi/13205/2014(H10N6)2014.241
A/chicken/Shenzhen/2269/2013(H5N6)2013.948
A/chicken/Hunan/12.28_YYGK034-P/2013(H5N6)(H5N6)2013.989
A/chicken/Laos/LPQ001/2014(H5N6)2014.192
A/chicken/Dongguan/2690/2013(H5N6)2013.962
A/duck/Hunan/146/2014(H3N6)2014.866
A/duck/Jiangxi/95/2014(H5N6)2014.025
A/duck/Hunan/12.07__YYGK110-P/2013(H5N6)(H5N6)2013.932
A/duck/Dongguan/3069/2013(H5N6)2013.962
A/chicken/Shenzhen/715/2013(H5N6)2013.940
A/Hangzhou/01/2021(H5N6)2021.973
A/Chicken/Hangzhou/E1149/2021(H5N6)2021.975
A/duck/Zhejiang/S4854/2021(H5N6)(H5N6)2021.921
A/Hunan/10117/2021(H5N6)2021.693
A/Hunan/09285/2021(H5N6)2021.597
A/Hunan/09911/2021(H5N6)2021.671
A/GX-guilin/11151/2021(H5N6)2021.532
A/duck/Hunan/S40268/2021(H5N6)(H5N6)2021.915
A/duck/Guizhou/S4702/2021(H5N6)(H5N6)2021.918
A/duck/Guangxi/S31116/2021(H5N6)(H5N6)2021.795
A/duck/Vietnam/HN6611/2020(H5N6)2020.743
A/Muscovy_duck/Vietnam/HN6606/2020(H5N6)2020.743
A/Duck/Cambodia/e10T241C18/2020(H5N6)2020.697
A/Sichuan/06689/2021(H5N6)2021.507
A/duck/Yunnan/S4318/2021(H5N6)(H5N6)2021.836
A/Chongqing/02/2021(H5N6)2021.518
A/Sichuan/06681/2021(H5N6)2021.501
A/Goose/Sichuan/21765-8/2021(H5N6)(H5N6)2021.414
A/Chicken/Sichuan/21859-5/2021(H5N6)(H5N6)2021.496
A/Duck/Sichuan/21840-5/2021(H5N6)(H5N6)2021.496
A/Duck/Sichuan/21898/2021(H5N6)(H5N6)2021.496
A/Duck/Sichuan/21406-8/2021(H5N6)(H5N6)2021.247
A/Muscovy_duck/China/H5N6/2020(H5N6)2020.612
A/duck/China/FJ18252/2019(H5N6)2019.915
A/duck/Jiangxi/11.17_NCNP48Y2-C/2016(H5N6)2016.877
A/mink/Northern_China/110/2018(H5N6)2018.068
A/chicken/Japan/AQ-HE144/2015(H5N6)2015.899
A/duck/Hunan/12.27_YYGK95J2-O/2016(H5N6)2016.986
A/duck/Hunan/1.17_YYGKK80-OC/2017(H5N6)2017.044
A/chicken/Hunan/12.27_YYGK19J2-C/2016(H5N6)2016.986
A/Fujian-Sanyuan/21099/2017(H5N6)2017.981
A/chicken/Anhui/AH325/2015(H5N6)2015.964
A/duck/Anhui/AH328/2015(H5N6)2015.964
A/duck/Hunan/S40199/2021(H5N6)(H5N6)2021.915
A/Changsha/1/2022(H5N6)2022.860
A/Guangdong/1/2021(H5N6)2021.630
A/Goose/Guangdong/211057/2021(H5N6)(H5N6)2021.581
A/Chicken/Guangdong/211106-3/2021(H5N6)(H5N6)2021.581
A/duck/Laos/NL-2175410/2021(H5N6)2021.205
A/chicken/Chongqing/10.28_RCTYH007-O/2018(A)2018.822
A/chicken/Anhui/11.29_YHZGS004-C/2018(A)2018.910
A/duck/Hunan/5.29_YYGK77P3-OC/2018(A)2018.405
A/chicken/Sichuan/NCJPL1/2014(H5N6)2014.318
A/duck/Sichuan/NCJPL7/2014(H5N6)2014.318
A/Sichuan/26221/2014(H5N6)2014.301
A/cat/Sichuan/SC18/2014(H5N6)2014.332
A/duck/Ha_Tinh/HT7/2014(H5N6)2014.364
A/duck/Hunan/02.16_YYGK281-P/2014(H5N6)(H5N6)2014.126
A/chicken/Jiangxi/04.01__NCJD0119-O/2015(H5N6)(H5N6)2015.247
A/duck/Hunan/01.16_YYFQH319-P/2014(H5N6)(H5N6)2014.041
A/migratory_waterfowl/Hubei/Chenhu1347/2014(H5N6)2014.153
A/duck/Hunan/12.07_YYGK032-P/2013(H5N6)(H5N6)2013.932
A/swan_goose/Jilin/JL01/2014(H5N6)2014.414
A/chicken/Yangzhou/YD1/2014(H5N6)2014.666
A/Duck/Guangdong/SS1A1/2014H5N6(H5N6)2014.200
A/duck/Jiangxi/13484/2014(H5N6)2014.241
A/chicken/Shenzhen/1845/2013(H5N6)2013.945
A/black-headed_gull/Denmark/4663-1/2018(H5N6)2018.230
A/Anas_platyrhynchos/Korea/W612/2017(H5N6)2017.948
A/Mallard/Korea/K17-1825/2017(H5N6)2017.973
A/chicken/Kagawa/1T-2/2018(H5N6)2018.025
A/duck/Korea/H35/2017(H5N6)2017.940
A/mandarin_duck/Korea/H69/2017(H5N6)2017.964
A/spotbill_duck/Korea/WA159/2018(H5N6)2018.085
A/turkey/Germany-SH/AR185-L02549/2018(H5N6)2018.211
A/Chicken/Netherlands/18000887-005/2018(H5N6)2018.052
A/common_pochard/Germany-BY/AR09-18-L02421/2017(H5N6)2017.989
A/Armenian_Gull/Republic_of_Georgia/4/2017(H5N6)2017.986
A/duck/Nigeria/SK28T_19VIR8424-2/2019(H5N6)2019.493
A/Mallard/Republic_of_Georgia/1/2018(H5N6)2018.074
A/Great_Black-backed_Gull/Netherlands/1/2017_(H5N6)2017.962
A/Mute_Swan/Netherlands/17017377-001/2017(H5N6)2017.937
A/Tufted_Duck/Netherlands/17017367-007/2017(H5N6)2017.937
A/Black-headed_Gull/Netherlands/29/2017(H5N6)2017.962
A/Duck/Netherlands/17017237-001-005/2017(H5N6)2017.932
A/Bird/Netherlands/17017775-035-039/2017(H5N6)2017.953
A/Peacock/Netherlands/17017775-064-068/2017(H5N6)2017.953
A/Guineafowl/Netherlands/17017775-020-024/2017(H5N6)2017.953
A/Buzzard/Netherlands/18004242-002/2018(H5N6)2017.200
A/mute_swan/Shimane/3211A002/2017(H5N6)2017.874
A/mallard/Korea/Jeju-H24/2017(H5N6)2017.904
A/Spoonbill/HK/17-18259/2017(H5N6)2017.973
A/Northern_Goshawk/Tokyo/1301B003/2018(H5N6)2018.011
A/chicken/South_Korea/AIV49/2017(H5N6)2017.833
A/chicken/Greece/39_2017/2017(H5N6)2017.099
A/chicken/Greece/39_2017b/2017(H5N6)2017.099
A/mallard_duck/Georgia/3/2016(H4N6)2016.746
A/mallard_duck/Georgia/4/2016(H4N6)2016.746
2.0
2005
2010
2015
2020
2025

Fig.S2G
N6

Fig.S2H
N8

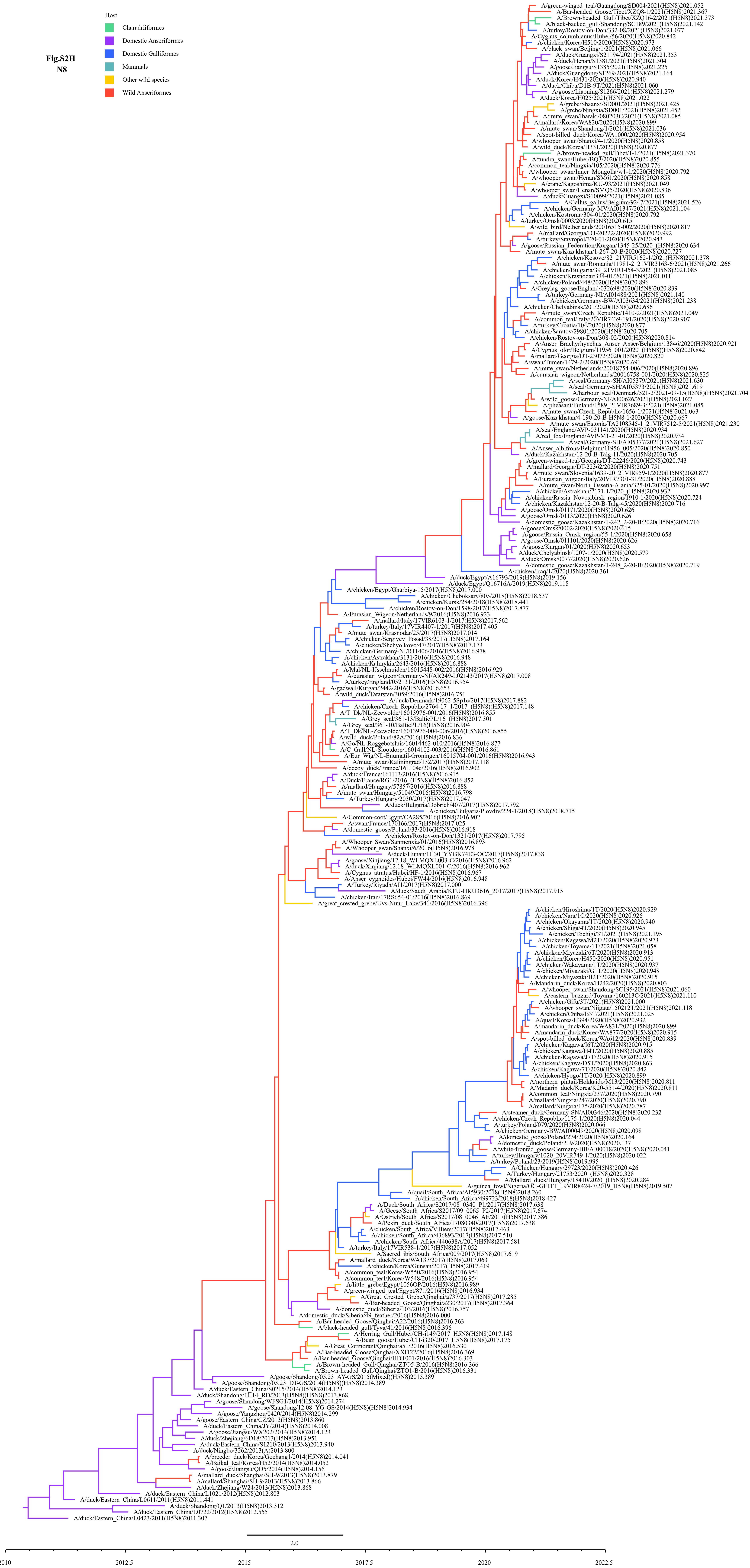
Host
Charadriiformes
Domestic Anseriformes
Domestic Galliformes
Mammals
Other wild species
Wild Anseriformes
A/green-winged_teal/Guangdong/SD004/2021(H5N8)2021.052
A/Bar-headed_Goose/Tibet/XZQ8-1/2021(H5N8)2021.367
A/Brown-headed_Gull/Tibet/XZQ16-2/2021(H5N8)2021.373
A/black-backed_gull/Shandong/SC189/2021(H5N8)2021.142
A/turkey/Rostov-on-Don/332-08/2021(H5N8)2021.077
A/Cygnus_columbianus/Hubei/56/2020(H5N8)2020.842
A/chicken/Korea/H510/2020(H5N8)2020.973
A/black_swan/Beijing/1/2021(H5N8)2021.066
A/duck/Guangxi/S21194/2021(H5N8)2021.353
A/duck/Henan/S1381/2021(H5N8)2021.304
A/goose/Jiangsu/S1385/2021(H5N8)2021.225
A/duck/Guangdong/S1269/2021(H5N8)2021.164
A/duck/Korea/H431/2020(H5N8)2020.940
A/duck/Chiba/D1B-9T/2021(H5N8)2021.060
A/goose/Liaoning/S1266/2021(H5N8)2021.279
A/duck/Korea/H025/2021(H5N8)2021.022
A/grebe/Shaanxi/SD001/2021(H5N8)2021.425
A/grebe/Ningxia/SD001/2021(H5N8)2021.452
A/mute_swan/Ibaraki/080203C/2021(H5N8)2021.085
A/mallard/Korea/WA820/2020(H5N8)2020.899
A/mute_swan/Shandong/1/2021(H5N8)2021.036
A/spot-billed_duck/Korea/WA1000/2020(H5N8)2020.954
A/whooper_swan/Shanxi/4-1/2020(H5N8)2020.858
A/wild_duck/Korea/H331/2020(H5N8)2020.877
A/brown-headed_gull/Tibet/1-1/2021(H5N8)2021.370
A/tundra_swan/Hubei/BQ3/2020(H5N8)2020.855
A/common_teal/Ningxia/105/2020(H5N8)2020.776
A/whooper_swan/Inner_Mongolia/w1-1/2020(H5N8)2020.792
A/whooper_swan/Henan/SM61/2020(H5N8)2020.858
A/crane/Kagoshima/KU-93/2021(H5N8)2021.049
A/whooper_swan/Henan/SMQ5/2020(H5N8)2020.836
A/duck/Guangxi/S10099/2021(H5N8)2021.085
A/Gallus_gallus/Belgium/9247/2021(H5N8)2021.526
A/chicken/Germany-MV/AI01347/2021(H5N8)2021.104
A/chicken/Kostroma/304-01/2020(H5N8)2020.792
A/turkey/Omsk/0003/2020(H5N8)2020.615
A/wild_bird/Netherlands/20016515-002/2020(H5N8)2020.817
A/mallard/Georgia/DT-20222/2020(H5N8)2020.992
A/turkey/Stavropol/320-01/2020(H5N8)2020.943
A/goose/Russian_Federation/Kurgan/1345-25/2020_(H5N8)2020.634
A/mute_swan/Kazakhstan/1-267-20-B/2020(H5N8)2020.727
A/chicken/Kosovo/82_21VIR5162-1/2021(H5N8)2021.378
A/mute_swan/Romania/1981-2_21VIR3163-6/2021(H5N8)2021.266
A/chicken/Bulgaria/39_21VIR1454-3/2021(H5N8)2021.085
A/chicken/Krasnodar/334-01/2021(H5N8)2021.011
A/chicken/Poland/448/2020(H5N8)2020.896
A/Greylag_goose/England/032698/2020(H5N8)2020.839
A/turkey/Germany-NI/AI01488/2021(H5N8)2021.140
A/chicken/Germany-BW/AI03634/2021(H5N8)2021.238
A/chicken/Chelyabinsk/201/2020(H5N8)2020.686
A/mute_swan/Czech_Republic/1410-2/2021(H5N8)2021.049
A/common_teal/Italy/20VIR7439-191/2020(H5N8)2020.907
A/turkey/Croatia/104/2020(H5N8)2020.877
A/chicken/Saratov/29801/2020(H5N8)2020.705
A/chicken/Rostov-on-Don/308-02/2020(H5N8)2020.814
A/Anser_Brachyrhynchus_Anser_Anser/Belgium/13846/2020(H5N8)2020.921
A/Cygnus_olor/Belgium/11956_001/2020_(H5N8)(H5N8)2020.842
A/mallard/Georgia/DT-23072/2020(H5N8)2020.820
A/swan/Tumen/1479-2/2020(H5N8)2020.691
A/mute_swan/Netherlands/20018754-006/2020(H5N8)2020.896
A/eurasian_wigeon/Netherlands/20016758-001/2020(H5N8)2020.825
A/seal/Germany-SH/AI05379/2021(H5N8)2021.630
A/seal/Germany-SH/AI05373/2021(H5N8)2021.619
A/harbour_seal/Denmark/521-2/2021-09-15(H5N8)(H5N8)2021.704
A/wild_goose/Germany-NI/AI00626/2021(H5N8)2021.027
A/pheasant/Finland/1589_21VIR7689-3/2021(H5N8)2021.085
A/mute_swan/Czech_Republic/1656-1/2021(H5N8)2021.063
A/goose/Kazakhstan/4-190-20-B-H5N8-1/2020(H5N8)2020.667
A/mute_swan/Estonia/TA2108545-1_21VIR7512-5/2021(H5N8)2021.230
A/seal/England/AVP-031141/2020(H5N8)2020.934
A/red_fox/England/AVP-M1-21-01/2020(H5N8)2020.934
A/seal/Germany-SH/AI05377/2021(H5N8)2021.627
A/Anser_albifrons/Belgium/11956_005/2020(H5N8)2020.850
A/duck/Kazakhstan/12-20-B-Talg-11/2020(H5N8)2020.705
A/green-winged-teal/Georgia/DT-22246/2020(H5N8)2020.743
A/mallard/Georgia/DT-22362/2020(H5N8)2020.751
A/mute_swan/Slovenia/1639-20_21VIR959-1/2020(H5N8)2020.877
A/Eurasian_wigeon/Italy/20VIR7301-31/2020(H5N8)2020.888
A/mute_swan/North_Ossetia-Alania/325-01/2020(H5N8)2020.997
A/chicken/Astrakhan/2171-1/2020_(H5N8)2020.932
A/chicken/Russia_Novosibirsk_region/1910-1/2020(H5N8)2020.724
A/chicken/Kazakhstan/12-20-B-Talg-45/2020(H5N8)2020.716
A/goose/Omsk/01171/2020(H5N8)2020.626
A/goose/Omsk/0113/2020(H5N8)2020.626
A/domestic_goose/Kazakhstan/1-242_2-20-B/2020(H5N8)2020.716
A/goose/Omsk/0002/2020(H5N8)2020.615
A/goose/Russia_Omsk_region/55-1/2020(H5N8)2020.658
A/goose/Omsk/011101/2020(H5N8)2020.626
A/goose/Kurgan/01/2020(H5N8)2020.653
A/duck/Chelyabinsk/1207-1/2020(H5N8)2020.579
A/duck/Omsk/0077/2020(H5N8)2020.626
A/domestic_goose/Kazakhstan/1-248_2-20-B/2020(H5N8)2020.719
A/chicken/Iraq/1/2020(H5N8)2020.361
A/duck/Egypt/A16793/2019(H5N8)2019.156
A/duck/Egypt/Q16716A/2019(H5N8)2019.118
A/chicken/Egypt/Gharbiya-15/2017(H5N8)2017.000
A/chicken/Cheboksary/805/2018(H5N8)2018.537
A/chicken/Kursk/284/2018(H5N8)2018.441
A/chicken/Rostov-on-Don/1598/2017(H5N8)2017.877
A/Eurasian_Wigeon/Netherlands/9/2016(H5N8)2016.923
A/mallard/Italy/17VIR6103-1/2017(H5N8)2017.562
A/turkey/Italy/17VIR4407-1/2017(H5N8)2017.405
A/mute_swan/Krasnodar/25/2017(H5N8)2017.014
A/chicken/Sergiyev_Posad/38/2017(H5N8)2017.164
A/chicken/Shchyolkovo/47/2017(H5N8)2017.173
A/chicken/Germany-NI/R11406/2016(H5N8)2016.978
A/chicken/Astrakhan/3131/2016(H5N8)2016.948
A/chicken/Kalmykia/2643/2016(H5N8)2016.888
A/Mal/NL-IJsselmuiden/16015448-002/2016(H5N8)2016.929
A/eurasian_wigeon/Germany-NI/AR249-L02143/2017(H5N8)2017.008
A/turkey/England/052131/2016(H5N8)2016.954
A/gadwall/Kurgan/2442/2016(H5N8)2016.653
A/wild_duck/Tatarstan/3059/2016(H5N8)2016.751
A/duck/Denmark/19062-5Sp1c/2017(H5N8)2017.882
A/chicken/Czech_Republic/2764-17_1/2017_(H5N8)(H5N8)2017.148
A/T_Dk/NL-Zeewolde/16013976-001/2016(H5N8)2016.855
A/Grey_seal/361-13/BalticPL/16_(H5N8)2017.301
A/Grey_seal/361-10/BalticPL/16(H5N8)2016.904
A/T_Dk/NL-Zeewolde/16013976-004-006/2016(H5N8)2016.855
A/wild_duck/Poland/82A/2016(H5N8)2016.836
A/Go/NL-Roggebotsluis/16014462-010/2016(H5N8)2016.877
A/C_Gull/NL-Slootdorp/16014102-003/2016(H5N8)2016.861
A/Eur_Wig/NL-Enumatil-Groningen/16015704-001/2016(H5N8)2016.943
A/mute_swan/Kaliningrad/132/2017(H5N8)2017.118
A/decoy_duck/France/161104e/2016(H5N8)2016.902
A/duck/France/161113/2016(H5N8)2016.915
A/Duck/France/RG1/2016_(H5N8)(H5N8)2016.852
A/mallard/Hungary/57857/2016(H5N8)2016.888
A/mute_swan/Hungary/51049/2016(H5N8)2016.798
A/Turkey/Hungary/2030/2017(H5N8)2017.047
A/duck/Bulgaria/Dobrich/407/2017(H5N8)2017.792
A/chicken/Bulgaria/Plovdiv/224-1/2018(H5N8)2018.715
A/Common-coot/Egypt/CA285/2016(H5N8)2016.902
A/swan/France/170166/2017(H5N8)2017.025
A/domestic_goose/Poland/33/2016(H5N8)2016.918
A/chicken/Rostov-on-Don/1321/2017(H5N8)2017.795
A/Whooper_Swan/Sanmenxia/01/2016(H5N8)2016.893
A/Whooper_swan/Shanxi/6/2016(H5N8)2016.978
A/duck/Hunan/11.30_YYGK74E3-OC/2017(H5N8)2017.838
A/goose/Xinjiang/12.18_WLMQXL003-C/2016(H5N8)2016.962
A/duck/Xinjiang/12.18_WLMQXL001-C/2016(H5N8)2016.962
A/Cygnus_atratus/Hubei/HF-1/2016(H5N8)2016.967
A/Anser_cygnoides/Hubei/FW44/2016(H5N8)2016.948
A/Turkey/Riyadh/A11/2017(H5N8)2017.000
A/duck/Saudi_Arabia/KFU-HKU3616_2017/2017(H5N8)2017.915
A/chicken/Iran/17RS654-01/2016(H5N8)2016.869
A/great_crested_grebe/Uvs-Nuur_Lake/341/2016(H5N8)2016.396
A/chicken/Hiroshima/1T/2020(H5N8)2020.929
A/chicken/Nara/1C/2020(H5N8)2020.926
A/chicken/Okayama/1T/2020(H5N8)2020.940
A/chicken/Shiga/4T/2020(H5N8)2020.945
A/chicken/Tochigi/3T/2021(H5N8)2021.195
A/chicken/Kagawa/M2T/2020(H5N8)2020.973
A/chicken/Toyama/1T/2021(H5N8)2021.058
A/chicken/Miyazaki/6T/2020(H5N8)2020.913
A/chicken/Korea/H450/2020(H5N8)2020.951
A/chicken/Wakayama/1T/2020(H5N8)2020.937
A/chicken/Miyazaki/G1T/2020(H5N8)2020.948
A/chicken/Miyazaki/B2T/2020(H5N8)2020.915
A/Mandarin_duck/Korea/H242/2020(H5N8)2020.803
A/whooper_swan/Shandong/SC195/2021(H5N8)2021.060
A/eastern_buzzard/Toyama/160213C/2021(H5N8)2021.110
A/chicken/Gifu/3T/2021(H5N8)2021.000
A/whooper_swan/Niigata/150212T/2021(H5N8)2021.118
A/chicken/Chiba/B3T/2021(H5N8)2021.025
A/quail/Korea/H394/2020(H5N8)2020.932
A/mandarin_duck/Korea/WA831/2020(H5N8)2020.899
A/mandarin_duck/Korea/WA877/2020(H5N8)2020.915
A/spot-billed_duck/Korea/WA612/2020(H5N8)2020.839
A/chicken/Kagawa/I6T/2020(H5N8)2020.915
A/chicken/Kagawa/H4T/2020(H5N8)2020.885
A/chicken/Kagawa/J7T/2020(H5N8)2020.915
A/chicken/Kagawa/D5T/2020(H5N8)2020.863
A/chicken/Kagawa/7T/2020(H5N8)2020.842
A/chicken/Hyogo/1T/2020(H5N8)2020.899
A/northern_pintail/Hokkaido/M13/2020(H5N8)2020.811
A/Madarin_duck/Korea/K20-551-4/2020(H5N8)2020.811
A/common_teal/Ningxia/237/2020(H5N8)2020.790
A/mallard/Ningxia/247/2020(H5N8)2020.790
A/mallard/Ningxia/175/2020(H5N8)2020.787
A/steamer_duck/Germany-SN/AI00346/2020(H5N8)2020.232
A/chicken/Czech_Republic/1175-1/2020(H5N8)2020.044
A/turkey/Poland/079/2020(H5N8)2020.066
A/chicken/Germany-BW/AI00049/2020(H5N8)2020.098
A/domestic_goose/Poland/274/2020(H5N8)2020.164
A/domestic_duck/Poland/219/2020(H5N8)2020.137
A/white-fronted_goose/Germany-BB/AI00018/2020(H5N8)2020.041
A/turkey/Hungary/1020_20VIR749-1/2020(H5N8)2020.022
A/turkey/Poland/23/2019(H5N8)2019.995
A/Chicken/Hungary/29723/2020(H5N8)2020.426
A/Turkey/Hungary/21753/2020_(H5N8)2020.328
A/Mallard_duck/Hungary/18410/2020_(H5N8)2020.284
A/guinea_fowl/Nigeria/OG-GF11T_19VIR8424-7/2019_(H5N8)(H5N8)2019.507
A/quail/South_Africa/A15930/2018(H5N8)2018.260
A/chicken/South_Africa/499723/2018(H5N8)2018.427
A/Duck/South_Africa/S2017/08_0340_P1/2017(H5N8)2017.638
A/Geese/South_Africa/S2017/09_0065_P2/2017(H5N8)2017.674
A/Ostrich/South_Africa/S2017/08_0046_AF/2017(H5N8)2017.586
A/Pekin_duck/South_Africa/17080340/2017(H5N8)2017.638
A/chicken/South_Africa/Villiers/2017(H5N8)2017.463
A/chicken/South_Africa/436893/2017(H5N8)2017.510
A/chicken/South_Africa/440638A/2017(H5N8)2017.581
A/turkey/Italy/17VIR538-1/2017(H5N8)2017.052
A/Sacred_ibis/South_Africa/009/2017(H5N8)2017.619
A/mallard_duck/Korea/WA137/2017(H5N8)2017.063
A/chicken/Korea/Gunsan/2017(H5N8)2017.419
A/common_teal/Korea/W550/2016(H5N8)2016.954
A/common_teal/Korea/W548/2016(H5N8)2016.954
A/little_grebe/Egypt/1056OP/2016(H5N8)2016.989
A/green-winged_teal/Egypt/871/2016(H5N8)2016.934
A/Great_Crested_Grebe/Qinghai/a737/2017(H5N8)2017.285
A/Bar-headed_Goose/Qinghai/a230/2017(H5N8)2017.364
A/domestic_duck/Siberia/103/2016(H5N8)2016.757
A/domestic_duck/Siberia/49_feather/2016(H5N8)2016.000
A/Bar-headed_Goose/Qinghai/A22/2016(H5N8)2016.363
A/black-headed_gull/Tyva/41/2016(H5N8)2016.396
A/Herring_Gull/Hubei/CH-i149/2017_H5N8(H5N8)2017.148
A/Bean_goose/Hubei/CH-i320/2017_H5N8(H5N8)2017.175
A/Great_Cormorant/Qinghai/a51/2016(H5N8)2016.530
A/Bar-headed_Goose/Qinghai/XXI122/2016(H5N8)2016.369
A/Bar-headed_Goose/Qinghai/HDT001/2016(H5N8)2016.303
A/Brown-headed_Gull/Qinghai/ZTO5-B/2016(H5N8)2016.366
A/Brown-headed_Gull/Qinghai/ZTO1-B/2016(H5N8)2016.331
A/goose/Shandong/05.23_AY-GS/2015(Mixed)(H5N8)2015.389
A/goose/Shandong/05.23_DT-GS/2014(H5N8)(H5N8)2014.389
A/duck/Eastern_China/S0215/2014(H5N8)2014.123
A/duck/Shandong/11.14_RD/2013(H5N8)(H5N8)2013.868
A/goose/Shandong/WFSG1/2014(H5N8)2014.274
A/goose/Shandong/12.08_YG-GS/2014(H5N8)(H5N8)2014.934
A/goose/Yangzhou/0420/2014(H5N8)2014.299
A/goose/Eastern_China/CZ/2013(H5N8)2013.860
A/duck/Eastern_China/JY/2014(H5N8)2014.008
A/goose/Jiangsu/WX202/2014(H5N8)2014.123
A/duck/Zhejiang/6D18/2013(H5N8)2013.951
A/duck/Eastern_China/S1210/2013(H5N8)2013.940
A/duck/Ningbo/3262/2013(A)2013.800
A/breeder_duck/Korea/Gochang1/2014(H5N8)2014.041
A/Baikal_teal/Korea/H52/2014(H5N8)2014.052
A/goose/Jiangsu/QD5/2014(H5N8)2014.156
A/mallard_duck/Shanghai/SH-9/2013(H5N8)2013.879
A/mallard/Shanghai/SH-9/2013(H5N8)2013.866
A/duck/Zhejiang/W24/2013(H5N8)2013.868
A/duck/Eastern_China/L1021/2012(H5N8)2012.803
A/duck/Eastern_China/L0611/2011(H5N8)2011.441
A/duck/Shandong/Q1/2013(H5N8)2013.312
A/duck/Eastern_China/L0722/2012(H5N8)2012.555
A/duck/Eastern_China/L0423/2011(H5N8)2011.307
2.0
2010
2012.5
2015
2017.5
2020
2022.5

Fig.S2I
PB2

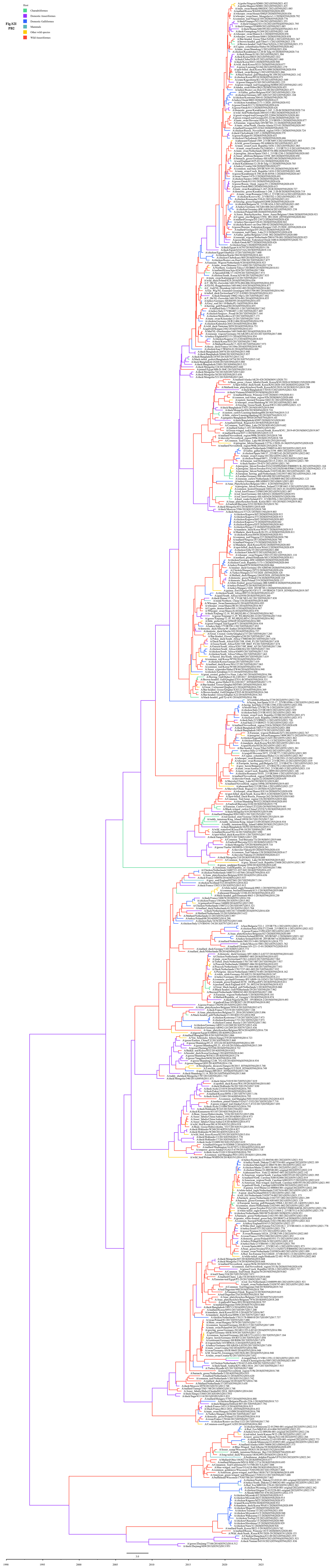

Host
Charadriiformes
Domestic Anseriformes
Domestic Galliformes
Mammals
Other wild species
Wild Anseriformes
3.0
1990
1995
2000
2005
2010
2015
2020
2025

Fig.S2J
PB1

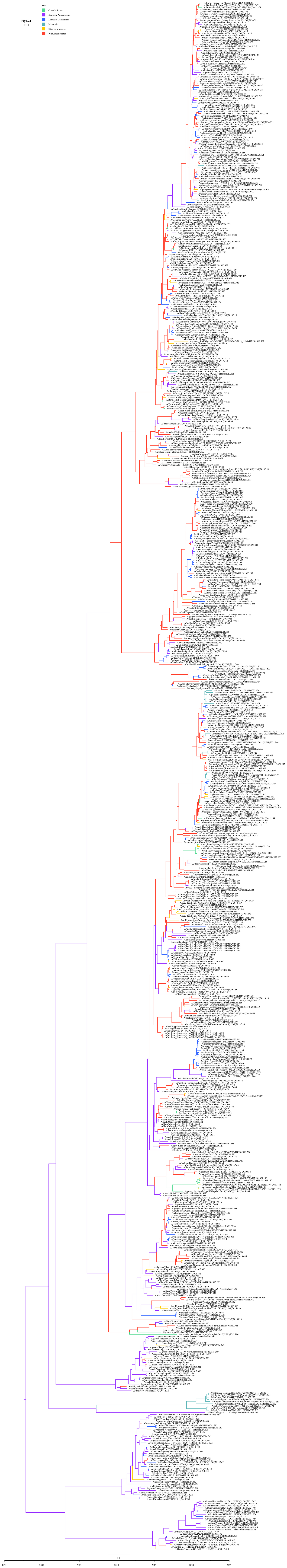
Host
Charadriiformes
Domestic Anseriformes
Domestic Galliformes
Mammals
Other wild species
Wild Anseriformes
3.0
1995
2000
2005
2010
2015
2020
2025

Fig.S2K
PA

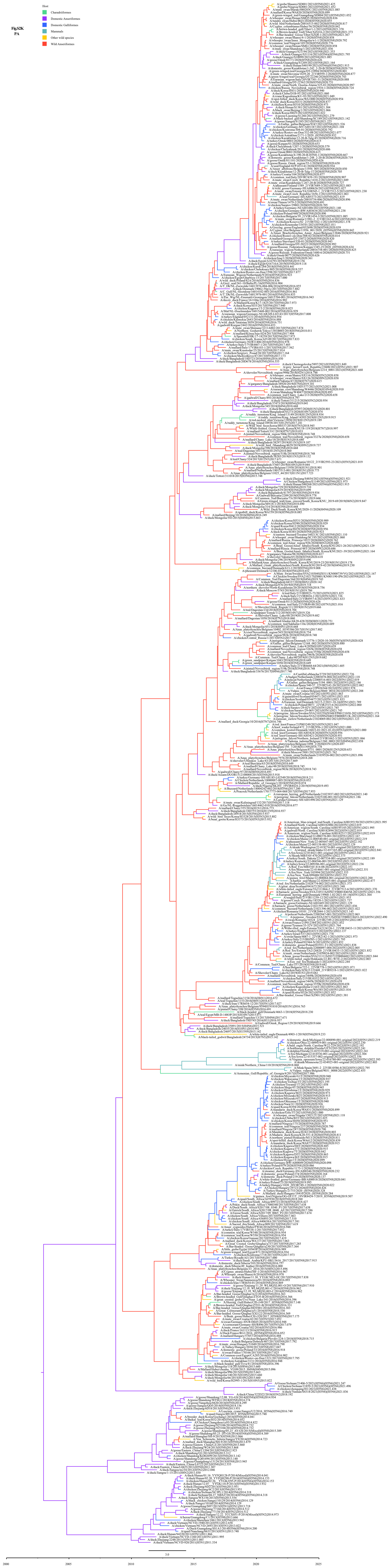
Host
Charadriiformes
Domestic Anseriformes
Domestic Galliformes
Mammals
Other wild species
Wild Anseriformes
3.0
2000
2005
2010
2015
2020
2025

Fig.S2L M

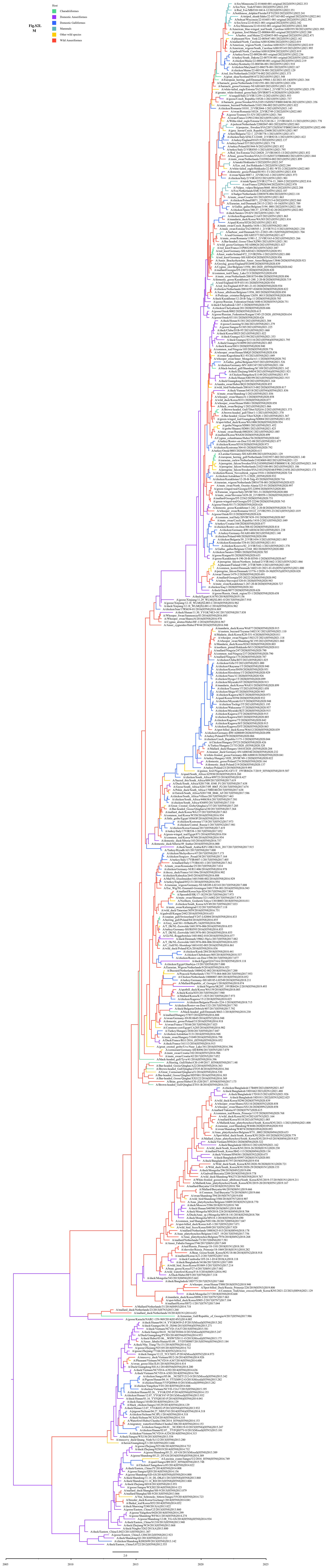

Host
Charadriiformes
Domestic Anseriformes
Domestic Galliformes
Mammals
Other wild species
Wild Anseriformes
2.0
2005
2010
2015
2020
2025

Fig.S2M
NS

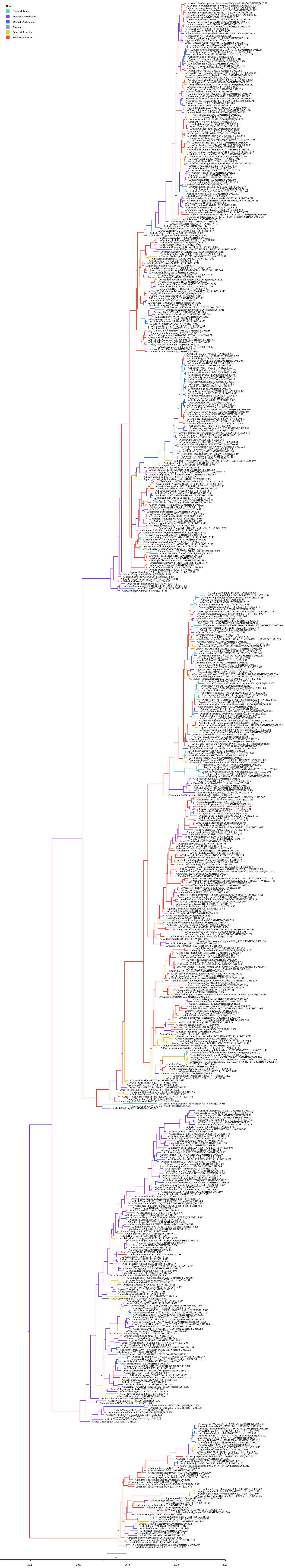
Host
Charadriiformes
Domestic Anseriformes
Domestic Galliformes
Mammals
Other wild species
Wild Anseriformes
2.0
2000
2005
2010
2015
2020
2025
