# Supplementary material for: Spatiotemporal and Species-Crossing Transmission Dynamics of Subclade 2.3.4.4b H5Nx HPAIVs
Source: Transbound Emerg Dis. 2024 Jul 10;2024:2862053. doi: 10.1155/2024/2862053 (PMC12017169; doi:10.1155/2024/2862053)
Supplement: Supplementary 7 — Table 5: Bayes factor supports between hosts. [file 2862053.f7.docx]

**Table S5.** Bayes factor supports. Bayes factor supports for individual transitions between discrete hosts. Bayes factors (BF) >3 and Posterior probability (PP) >0.5 are listed in table. Wild Anseriformes- WA; Domestic Galliformes- DG; Domestic Anseriformes- DA; Charadriiformes- CH; Other Wild Species- OWS; Mammals- MM.

| **Genes** | **Source** | **Sink** | **BF** | **PP** |
| --- | --- | --- | --- | --- |
| **H5** | WA | CH | 230552.60 | 1.00 |
|  | WA | DA | 230552.60 | 1.00 |
|  | WA | DG | 230552.60 | 1.00 |
|  | WA | MM | 230552.60 | 1.00 |
|  | WA | OWS | 230552.60 | 1.00 |
|  | DG | WA | 751.65 | 0.99 |
|  | DA | DG | 328.42 | 0.99 |
|  | MM | CH | 106.04 | 0.96 |
|  | CH | OWS | 25.34 | 0.86 |
|  | OWS | MM | 23.15 | 0.84 |
|  | DG | OWS | 12.59 | 0.75 |
|  | DA | WA | 6.29 | 0.60 |
|  | OWS | DG | 4.62 | 0.52 |
| **N1** | DG | DA | 230552.60 | 1.00 |
|  | WA | DG | 57634.96 | 0.99 |
|  | DG | WA | 13557.90 | 0.99 |
|  | DG | MM | 3655.36 | 0.99 |
|  | DG | OWS | 2708.17 | 0.99 |
|  | DG | CH | 2130.52 | 0.99 |
|  | CH | DA | 4.25 | 0.50 |
| **N2** | DG | DA | 98.13 | 0.98 |
|  | DG | OWS | 17.09 | 0.88 |
|  | DA | WA | 13.74 | 0.86 |
|  | WA | OWS | 12.54 | 0.85 |
|  | WA | DA | 9.77 | 0.81 |
|  | WA | DG | 9.03 | 0.80 |
|  | DA | DG | 3.30 | 0.59 |
| **N3** | WA | DA | 88.00 | 0.96 |
|  | OWS | CH | 7.37 | 0.69 |
|  | DG | WA | 5.69 | 0.64 |
|  | CH | OWS | 5.53 | 0.63 |
|  | WA | OWS | 3.82 | 0.54 |
|  | WA | CH | 3.67 | 0.53 |
|  | WA | DG | 3.62 | 0.53 |
| **N4** | CH | OWS | 14.02 | 0.86 |
|  | WA | CH | 8.08 | 0.78 |
|  | DA | WA | 4.29 | 0.66 |
|  | OWS | CH | 3.06 | 0.58 |

| **Genes** | **Source** | **Sink** | **BF** | **PP** |
| --- | --- | --- | --- | --- |
| **N5** | WA | DG | 151.24 | 0.98 |
|  | WA | DA | 19.41 | 0.86 |
|  | DA | OWS | 10.39 | 0.76 |
|  | WA | OWS | 6.04 | 0.65 |
|  | WA | CH | 4.09 | 0.56 |
| **N6** | DA | MM | 230552.60 | 1.00 |
|  | WA | CH | 13557.90 | 0.99 |
|  | DA | DG | 1608.02 | 0.99 |
|  | DA | WA | 1048.50 | 0.99 |
|  | WA | DG | 39.10 | 0.90 |
|  | MM | DA | 27.41 | 0.87 |
|  | WA | DA | 19.92 | 0.82 |
|  | WA | OWS | 9.88 | 0.70 |
|  | DG | DA | 6.92 | 0.62 |
|  | OWS | WA | 6.04 | 0.59 |
| **N8** | WA | DA | 230552.60 | 1.00 |
|  | WA | OWS | 230552.60 | 1.00 |
|  | WA | DG | 3542.76 | 0.99 |
|  | WA | CH | 1104.18 | 0.99 |
|  | WA | MM | 869.05 | 0.99 |
|  | DA | WA | 799.06 | 0.99 |
|  | DG | WA | 711.75 | 0.99 |
| **PB2** | DA | WA | 230552.60 | 1.00 |
|  | WA | CH | 230552.60 | 1.00 |
|  | WA | DA | 230552.60 | 1.00 |
|  | WA | DG | 230552.60 | 1.00 |
|  | WA | OWS | 230552.60 | 1.00 |
|  | DG | MM | 6982.30 | 0.99 |
|  | WA | MM | 188.83 | 0.98 |
|  | DG | WA | 69.02 | 0.94 |
|  | CH | OWS | 30.75 | 0.88 |
|  | DG | DA | 18.74 | 0.81 |
| **PB1** | DA | DG | 112078.50 | 1.00 |
|  | DA | WA | 112078.50 | 1.00 |
|  | WA | CH | 112078.50 | 1.00 |
|  | WA | DA | 112078.50 | 1.00 |
|  | WA | DG | 112078.50 | 1.00 |
|  | WA | MM | 112078.50 | 1.00 |
|  | WA | OWS | 112078.50 | 1.00 |
|  | CH | OWS | 276.64 | 0.98 |
|  | DG | DA | 75.28 | 0.95 |
|  | DG | WA | 15.26 | 0.78 |
|  | DG | OWS | 11.04 | 0.72 |
|  | DA | OWS | 9.70 | 0.69 |

| **Genes** | **Source** | **Sink** | **BF** | **PP** |
| --- | --- | --- | --- | --- |
| **PA** | WA | CH | 230552.60 | 1.00 |
|  | WA | DA | 230552.60 | 1.00 |
|  | WA | DG | 230552.60 | 1.00 |
|  | WA | MM | 230552.60 | 1.00 |
|  | WA | OWS | 230552.60 | 1.00 |
|  | DA | DG | 6063.02 | 0.99 |
|  | MM | DG | 1029.62 | 0.99 |
|  | CH | OWS | 759.16 | 0.99 |
|  | DG | WA | 540.78 | 0.99 |
|  | DG | OWS | 116.13 | 0.96 |
|  | DA | WA | 19.46 | 0.82 |
|  | DG | DA | 6.60 | 0.61 |
| **NP** | DA | DG | 134407.90 | 1.00 |
|  | DA | WA | 134407.90 | 1.00 |
|  | WA | CH | 134407.90 | 1.00 |
|  | WA | DA | 134407.90 | 1.00 |
|  | WA | DG | 134407.90 | 1.00 |
|  | WA | MM | 134407.90 | 1.00 |
|  | WA | OWS | 134407.90 | 1.00 |
|  | DG | DA | 5165.43 | 0.99 |
|  | DG | WA | 185.58 | 0.98 |
|  | CH | DG | 65.02 | 0.94 |
|  | CH | OWS | 27.17 | 0.86 |
| **M** | WA | CH | 230552.60 | 1.00 |
|  | WA | DA | 230552.60 | 1.00 |
|  | WA | DG | 230552.60 | 1.00 |
|  | WA | OWS | 230552.60 | 1.00 |
|  | WA | MM | 238.42 | 0.98 |
|  | DG | WA | 233.91 | 0.98 |
|  | DA | WA | 163.41 | 0.98 |
|  | DA | DG | 70.44 | 0.94 |
|  | OWS | CH | 4.49 | 0.51 |
| **NS** | DA | DG | 230552.60 | 1.00 |
|  | WA | CH | 230552.60 | 1.00 |
|  | WA | DA | 230552.60 | 1.00 |
|  | WA | DG | 230552.60 | 1.00 |
|  | WA | MM | 230552.60 | 1.00 |
|  | WA | OWS | 230552.60 | 1.00 |
|  | DA | WA | 32932.43 | 0.99 |
|  | DG | WA | 16464.08 | 0.99 |
|  | DA | OWS | 57.38 | 0.93 |
|  | OWS | CH | 6.70 | 0.61 |
