# Supplementary material for: Spatiotemporal and Species-Crossing Transmission Dynamics of Subclade 2.3.4.4b H5Nx HPAIVs
Source: Transbound Emerg Dis. 2024 Jul 10;2024:2862053. doi: 10.1155/2024/2862053 (PMC12017169; doi:10.1155/2024/2862053)
Supplement: Supplementary 8 — Table 6: Markov rewards for time spent at each host. [file 2862053.f8.docx]

**Table S6. Markov rewards (%).** The proportion of time that external genes and internal genes of the virus spend in different host groups. Wild Anseriformes- WA; Domestic Galliformes- DG; Domestic Anseriformes- DA; Charadriiformes- CH; Other Wild Species- OWS; Mammals- MM.

| **Gene** | **Host** | **Mean** | **Gene** | **Host** | **Mean** |
| --- | --- | --- | --- | --- | --- |
| **H5** | WA | 42.87 | **N1** | WA | 39.11 |
|  | DG | 25.64 |  | DG | 37.22 |
|  | DA | 18.68 |  | DA | 7.57 |
|  | CH | 3.01 |  | CH | 5.28 |
|  | MM | 4.56 |  | MM | 7.33 |
|  | OWS | 5.23 |  | OWS | 3.49 |
| **N2** | WA | 28.26 | **N4** | WA | 54.55 |
|  | DG | 33.27 |  | DA | 12.83 |
|  | DA | 32.27 |  | CH | 20.70 |
|  | OWS | 6.20 |  | OWS | 11.94 |
| **N3** | WA | 75.72 | **N5** | WA | 73.10 |
|  | DG | 15.39 |  | DG | 5.80 |
|  | DA | 1.75 |  | DA | 10.91 |
|  | CH | 3.53 |  | CH | 4.48 |
|  | OWS | 3.62 |  | OWS | 5.72 |
| **N6** | WA | 16.79 | **N8** | WA | 36.48 |
|  | DA | 51.14 |  | DG | 25.16 |
|  | DG | 17.32 |  | DA | 31.42 |
|  | CH | 0.66 |  | CH | 1.74 |
|  | MM | 8.42 |  | MM | 2.42 |
|  | OWS | 5.66 |  | OWS | 2.78 |
| **PB2** | WA | 47.54 | **PB1** | WA | 44.01 |
|  | DG | 13.17 |  | DG | 11.30 |
|  | DA | 31.29 |  | DA | 35.56 |
|  | CH | 3.88 |  | CH | 2.42 |
|  | MM | 0.87 |  | MM | 3.67 |
|  | OWS | 3.25 |  | OWS | 3.04 |
| **PA** | WA | 46.00 | **NP** | WA | 43.69 |
|  | DG | 13.20 |  | DG | 15.48 |
|  | DA | 28.12 |  | DA | 32.25 |
|  | CH | 3.59 |  | CH | 2.53 |
|  | MM | 6.38 |  | MM | 3.44 |
|  | OWS | 2.71 |  | OWS | 2.61 |
| **M** | WA | 41.62 | **NS** | WA | 39.84 |
|  | DG | 17.32 |  | DA | 31.69 |
|  | DA | 31.41 |  | DG | 17.11 |
|  | CH | 3.19 |  | CH | 2.72 |
|  | MM | 2.92 |  | MM | 3.57 |
|  | OWS | 3.55 |  | OWS | 5.07 |
