# Supplementary material for: Spatiotemporal and Species-Crossing Transmission Dynamics of Subclade 2.3.4.4b H5Nx HPAIVs
Source: Transbound Emerg Dis. 2024 Jul 10;2024:2862053. doi: 10.1155/2024/2862053 (PMC12017169; doi:10.1155/2024/2862053)
Supplement: Supplementary 9 — Table 7: Markov jumps for individual transitions between hosts. [file 2862053.f9.docx]

**Table S7.** Markov jumps (%) for individual transitions between discrete host groups. Wild Anseriformes- WA; Domestic Galliformes- DG; Domestic Anseriformes- DA; Charadriiformes- CH; Other Wild Species- OWS; Mammals- MM.

| **Genes** | **Source** | **Sink** | | | | | |
| --- | --- | --- | --- | --- | --- | --- | --- |
|  |  | **CH** | **DA** | **DG** | **MM** | **OWS** | **WA** |
| **HA** | CH |  | 0.13 | 0.016 | 0.0029 | 1.61 | 0.032 |
|  | DA | 0.0093 |  | 6.98 | 0.0072 | 0.0054 | 1.41 |
|  | DG | 0.015 | 0.91 |  | 0.029 | 2.29 | 7.96 |
|  | MM | 0.44 | 0.0031 | 0.0048 |  | 0.0029 | 0.016 |
|  | OWS | 0.14 | 0.06 | 0.92 | 0.64 |  | 0.20 |
|  | WA | 9.96 | 21.36 | 28.15 | 5.52 | 11.18 |  |
| **1N** | CH |  | 1.74 | 0.10 | 1.65 | 0.51 | 0.077 |
|  | DA | 0.33 |  | 0.10 | 0.45 | 0.16 | 0.063 |
|  | DG | 12.58 | 18.37 |  | 14.50 | 12.98 | 16.65 |
|  | MM | 0.91 | 0.31 | 0.18 |  | 0.064 | 0.11 |
|  | OWS | 0.23 | 0.12 | 0.37 | 0.17 |  | 0.078 |
|  | WA | 0.21 | 0.20 | 16.31 | 0.0097 | 0.45 |  |
| **2N** | DA |  |  | 7.91 |  | 4.73 | 17.92 |
|  | DG |  | 12.07 |  |  | 5.73 | 0.99 |
|  | OWS |  | 2.70 | 1.19 |  |  | 2.91 |
|  | WA |  | 19.57 | 13.11 |  | 11.17 |  |
| **3N** | CH |  | 0.21 | 0.61 |  | 21.02 | 1.01 |
|  | DA | 0.39 |  | 1.52 |  | 0.32 | 2.20 |
|  | DG | 0.22 | 0.37 |  |  | 0.22 | 14.95 |
|  |  |  |  |  |  |  |  |
|  | OWS | 15.94 | 0.21 | 0.72 |  |  | 1.19 |
|  | WA | 7.46 | 16.21 | 7.58 |  | 7.65 |  |
| **4N** | CH |  | 0.98 |  |  | 37.78 | 1.87 |
|  | DA | 1.23 |  |  |  | 0.57 | 10.47 |
|  | OWS | 11.44 | 1.09 |  |  |  | 2.61 |
|  | WA | 18.91 | 7.27 |  |  | 5.79 |  |
| **5N** | CH |  | 0.16 | 0.12 |  | 0.065 | 0.051 |
|  | DA | 0.0064 |  | 20.70 |  | 0.57 | 7.01 |
|  | DG | 0.054 | 12.49 |  |  | 9.23 | 3.35 |
|  | OWS | 0.062 | 3.34 | 0.40 |  |  | 6.51 |
|  | WA | 11.05 | 9.50 | 8.54 |  | 6.80 |  |
| **6N** | CH |  | 0.064 | 0.041 | 0.0045 | 0.011 | 0.019 |
|  | DA | 0.0015 |  | 20.03 | 15.97 | 0.048 | 10.44 |
|  | DG | 0.0071 | 12.21 |  | 0.12 | 2.96 | 0.63 |
|  | MM | 0.001 | 5.68 | 0.76 |  | 0.02 | 0.047 |
|  | OWS | 0.023 | 0.83 | 0.25 | 0.016 |  | 4.49 |
|  | WA | 5.37 | 6.37 | 6.83 | 0.015 | 6.74 |  |

| **Genes** | **Source** | **Sink** | | | | | |
| --- | --- | --- | --- | --- | --- | --- | --- |
|  |  | **CH** | **DA** | **DG** | **MM** | **OWS** | **WA** |
| **8N** | CH |  | 0.015 | 0.0096 | 0.0052 | 0.014 | 0.17 |
|  | DA | 0.033 |  | 0.51 | 0.008 | 0.0064 | 9.47 |
|  | DG | 0.0048 | 0.63 |  | 0.005 | 0.17 | 17.57 |
|  | MM | 0.0045 | 0.0086 | 0.0063 |  | 0.0047 | 0.0092 |
|  | OWS | 0.034 | 0.048 | 0.073 | 0.023 |  | 0.065 |
|  | WA | 6.21 | 21.03 | 27.49 | 3.21 | 13.16 |  |
| **PB2** | CH |  | 0.026 | 0.0015 | 0.0015 | 1.34 | 0.021 |
|  | DA | 0.35 |  | 0.063 | 0.0011 | 0.054 | 16.92 |
|  | DG | 0.0041 | 2.76 |  | 2.36 | 0.67 | 3.17 |
|  | MM | 0.0019 | 0.0048 | 0.007 |  | 0.0019 | 0.0037 |
|  | OWS | 0.29 | 0.038 | 0.13 | 0.0067 |  | 0.058 |
|  | WA | 9.19 | 19.02 | 27.63 | 2.46 | 13.40 |  |
| **PB1** | CH |  | 0.0046 | 0.00088 | 0.00068 | 1.59 | 0.0062 |
|  | DA | 0.022 |  | 8.40 | 0.0013 | 1.64 | 17.37 |
|  | DG | 0.0027 | 3.67 |  | 0.029 | 0.91 | 3.32 |
|  | MM | 0.0013 | 0.0082 | 0.16 |  | 0.0033 | 0.0036 |
|  | OWS | 0.053 | 0.044 | 0.022 | 0.051 |  | 0.11 |
|  | WA | 5.81 | 21.96 | 19.91 | 3.72 | 11.16 |  |
| **PA** | CH |  | 0.0083 | 0.0037 | 0.013 | 1.74 | 0.0072 |
|  | DA | 0.018 |  | 8.41 | 0.0035 | 0.057 | 7.99 |
|  | DG | 0.0057 | 1.89 |  | 0.038 | 3.20 | 9.80 |
|  | MM | 0.035 | 0.045 | 2.10 |  | 0.11 | 0.025 |
|  | OWS | 0.19 | 0.079 | 0.045 | 0.14 |  | 0.045 |
|  | WA | 7.49 | 24.08 | 16.54 | 4.45 | 11.46 |  |
| **NP** | CH |  | 0.051 | 0.42 | 0.0004 | 0.83 | 0.0029 |
|  | DA | 0.001 |  | 10.58 | 0.0011 | 0.13 | 13.94 |
|  | DG | 0.00075 | 4.84 |  | 0.0079 | 0.053 | 4.72 |
|  | MM | 0.00044 | 0.00095 | 0.018 |  | 0.01 | 0.0032 |
|  | OWS | 0.19 | 0.023 | 0.037 | 0.0088 |  | 0.025 |
|  | WA | 6.95 | 17.32 | 21.54 | 4.88 | 13.41 |  |
| **M** | CH |  | 0.031 | 0.0058 | 0.0085 | 0.09 | 0.037 |
|  | DA | 0.02 |  | 6.60 | 0.00093 | 0.0074 | 9.18 |
|  | DG | 0.024 | 1.42 |  | 0.054 | 0.29 | 10.08 |
|  | MM | 0.0073 | 0.0068 | 0.019 |  | 0.038 | 0.0073 |
|  | OWS | 0.60 | 0.032 | 0.095 | 0.31 |  | 0.061 |
|  | WA | 7.67 | 19.39 | 20.97 | 4.40 | 18.53 |  |
| **NS** | CH |  | 0.03 | 0.01 | 0.0015 | 0.17 | 0.049 |
|  | DA | 0.013 |  | 18.50 | 0.0021 | 3.00 | 9.67 |
|  | DG | 0.0064 | 0.40 |  | 0.058 | 0.29 | 10.17 |
|  | MM | 0.00098 | 0.0058 | 0.089 |  | 0.0032 | 0.0025 |
|  | OWS | 0.38 | 0.032 | 0.035 | 0.013 |  | 0.069 |
|  | WA | 6.46 | 21.21 | 11.40 | 3.79 | 14.15 |  |
