# Supplementary material for: Spatiotemporal and Species-Crossing Transmission Dynamics of Subclade 2.3.4.4b H5Nx HPAIVs
Source: Transbound Emerg Dis. 2024 Jul 10;2024:2862053. doi: 10.1155/2024/2862053 (PMC12017169; doi:10.1155/2024/2862053)
Supplement: Supplementary 10 — Table 8: migration rate for individual transitions between hosts. [file 2862053.f10.docx]

**Table S8.** Migration rate for individual transitions between discrete hosts. Wild Anseriformes- WA; Domestic Galliformes- DG; Domestic Anseriformes- DA; Charadriiformes- CH; Other Wild Species- OWS; Mammals- MM .

| **Genes** | **Host** | **Migration in** | **Migration out** | **Genes** | **Host** | **Migration in** | **Migration out** |
| --- | --- | --- | --- | --- | --- | --- | --- |
| **H5** | CH | 4.18 | 4.36 | **N1** | CH | 4.67 | 4.59 |
|  | DA | 5.84 | 4.81 |  | DA | 5.33 | 4.46 |
|  | DG | 6.89 | 4.58 |  | DG | 4.99 | 7.03 |
|  | OWS | 4.46 | 3.87 |  | OWS | 4.76 | 4.25 |
|  | WA | 4.63 | 8.11 |  | WA | 5.14 | 5.17 |
|  | MM | 3.86 | 4.12 |  | MM | 5.03 | 4.43 |
| **N2** | DA | 3.25 | 3.20 | **N4** | CH | 2.96 | 3.61 |
|  | DG | 2.96 | 2.46 |  | DA | 2.60 | 2.71 |
|  | OWS | 2.63 | 2.57 |  | OWS | 3.61 | 2.80 |
|  | WA | 3.03 | 3.65 |  | WA | 2.71 | 2.75 |
| **N3** | CH | 4.19 | 4.56 | **N5** | CH | 3.51 | 3.77 |
|  | DA | 3.57 | 3.75 |  | DA | 4.09 | 4.40 |
|  | DG | 3.69 | 3.94 |  | DG | 4.03 | 3.84 |
|  | OWS | 4.44 | 4.34 |  | OWS | 4.31 | 3.67 |
|  | WA | 4.02 | 3.33 |  | WA | 3.95 | 4.21 |
| **N6** | CH | 4.60 | 4.61 | **N8** | CH | 4.20 | 4.62 |
|  | DA | 5.64 | 5.20 |  | DA | 5.35 | 4.74 |
|  | DG | 5.02 | 5.48 |  | DG | 5.89 | 5.58 |
|  | MM | 4.85 | 4.82 |  | MM | 4.15 | 4.59 |
|  | OWS | 5.00 | 4.69 |  | OWS | 4.84 | 4.19 |
|  | WA | 4.76 | 5.08 |  | WA | 5.37 | 6.07 |
| **PB2** | CH | 3.94 | 4.16 | **PB1** | CH | 4.35 | 4.53 |
|  | DA | 5.23 | 5.18 |  | DA | 6.11 | 5.49 |
|  | DG | 5.62 | 4.01 |  | DG | 5.77 | 4.22 |
|  | OWS | 4.10 | 3.64 |  | OWS | 3.76 | 3.96 |
|  | WA | 5.11 | 5.87 |  | WA | 5.54 | 6.48 |
|  | MM | 2.99 | 4.14 |  | MM | 4.05 | 4.89 |
| **PA** | CH | 4.36 | 4.42 | **NP** | CH | 4.16 | 3.20 |
|  | DA | 5.89 | 5.28 |  | DA | 5.46 | 5.21 |
|  | DG | 5.31 | 5.43 |  | DG | 5.43 | 4.08 |
|  | OWS | 4.28 | 4.10 |  | OWS | 3.74 | 3.53 |
|  | WA | 5.95 | 6.44 |  | WA | 5.11 | 6.59 |
|  | MM | 4.14 | 4.27 |  | MM | 2.67 | 3.94 |
| **M** | CH | 4.33 | 4.71 | **NS** | CH | 3.83 | 4.32 |
|  | DA | 5.47 | 4.84 |  | DA | 5.30 | 4.85 |
|  | DG | 5.48 | 5.27 |  | DG | 5.73 | 4.66 |
|  | OWS | 5.44 | 4.20 |  | OWS | 4.06 | 3.73 |
|  | WA | 5.34 | 6.55 |  | WA | 4.85 | 5.46 |
|  | MM | 4.27 | 4.76 |  | MM | 3.01 | 3.75 |
